# Bioinformatics-based screening and validation of ferroptosis-related genes in sepsis and type 2 diabetes mellitus

**DOI:** 10.3389/ebm.2025.10612

**Published:** 2025-10-21

**Authors:** Heng Xiao, Zhonghua Ding, Cheng Liu, Xu He, Yanyan Tao

**Affiliations:** ^1^ Department of Emergency Medicine, First Affiliated Hospital of Bengbu Medical University, Bengbu, Anhui, China; ^2^ Department of Critical Care Medicine, First Affiliated Hospital of Bengbu Medical University, Bengbu, Anhui, China; ^3^ Department of Pharmacy, Peking University People’s Hospital, Beijing, China

**Keywords:** ferroptosis, sepsis, type 2 diabetes mellitus, drug prediction, pathway

## Abstract

Emerging clinical evidence underscores a bidirectional epidemiological linkage between sepsis and type 2 diabetes mellitus (T2DM). This study mechanistically investigates the underlying pathogenesis of this comorbidity, specifically focusing on the role of ferroptosis-related genes in its pathogenesis. A total of 1204 shared genes between sepsis and T2DM were screened using datasets from sepsis (GSE65682) and T2DM (GSE76894). GO and KEGG enrichment analyses, combined with WGCNA, were performed to identify key pathways and hub genes. Three signaling pathways—MAPK, adherens junction, and peroxisome—were significantly associated with the sepsis-T2DM interaction. Subsequent Pearson correlation analysis implicated ferroptosis as a critically involved process. Five core ferroptosis-related genes, including CDC25B, DPP7, FBXO31, PTCD3, and CNPY2, were were identified and experimentally validated using qRT-PCR. Furthermore, based on cMAP, we screened eight candidate drugs targeting these genes. Echinacea and Ibudilast were predicted to possess the greatest preclinical potential among them. This study provides a deeper insight into the shared pathogenesis of sepsis and T2DM, highlighting the pivotal role of ferroptosis in the development and progression of this comorbidity. Our findings offer preliminary insights into the sepsis-T2DM comorbidity, highlighting ferroptosis as a potential key pathological mechanism and identifying candidate targets for future therapeutic exploration.

## Impact statement

Advances in modern sequencing technologies, bioinformatics analysis have enabled researchers to explore the interrelationships between diseases and the direct links in their pathogenesis using human samples, rather than relying solely on animal or cellular models. This approach allows for the generation of more robust and convincing conclusions. In this study, we employed bioinformatics analysis combined with qRT-PCR validation to identify key genes and signaling pathways involved in sepsis and T2DM. Our findings provide new insights into the molecular mechanisms underlying ferroptosis in sepsis with T2DM and suggest potential therapeutic interventions for further exploration. This integrative approach holds promise for improving our understanding of this complex disease intersection and informing the development of targeted therapies.

## Introduction

Sepsis is a life-threatening condition characterized by a dysregulated host response to infection, leading to organ dysfunction. It remains a major global health challenge, with mortality rates exceeding 10% worldwide [1]. In 2017, sepsis incidence reached 50 million new cases, resulting in 11 million deaths and an alarming mortality rate of 22% [2]. Septic shock accounts for 8–10% of intensive care unit (ICU) fatalities, with persistently high associated mortality [3]. A recent systematic review reported a mortality rate of 27% among sepsis patients, underscoring the urgent need for improved therapeutic strategies [4]. The World Health Organization has identified sepsis and septic shock as critical public health priorities, emphasizing the necessity of enhanced prevention, diagnosis, and treatment strategies [5]. Early identification and intervention of “high-risk” sepsis patients are recognized as critical for improving clinical outcomes, underscoring the demand for novel approaches to reliably stratify patient risk and guide personalized treatment.

The global prevalence of diabetes, particularly T2DM, has been steadily increasing. According to the International Diabetes Federation (IDF), the number of people living with diabetes reached 536 million in 2021 and is projected to rise to 783 million by 2045, with approximately 80% of diabetic patients residing in middle- and low-income countries. The growth rate of diabetes is notably higher in these regions compared to high-income countries [6]. T2DM and its complications remain a leading cause of hospitalization, disability, and mortality [7, 8]. Hyperglycemia significantly increases the risk of infection, with diabetic patients exhibiting a 2-6-fold higher likelihood of developing sepsis-related complications compared to non-diabetic individuals [9–14]. Furthermore, diabetic patients demonstrate increased sepsis-associated morbidity and mortality, potentially due to impaired immune responses and delayed resolution of inflammation. Additionally, diabetic patients show higher rates of colonization by drug-resistant pathogens, including methicillin-resistant *Staphylococcus aureus* (MRSA), compared to non-diabetic patients [15]. These observations underscore the critical role of diabetes as a significant comorbidity in sepsis pathophysiology.

Ferroptosis, an iron-dependent form of regulated cell death driven by lipid peroxide accumulation, has emerged as a key mechanism in disease progression. Excessive reactive oxygen species (ROS) generation creates a pro-inflammatory microenvironment, driving pathological changes through lipid peroxidation and subsequent damage to biomolecules and cellular membranes [16]. This process triggers multiple forms of regulated cell death including ferroptosis [17, 18].In the report by Meng et al, it was demonstrated that the upregulation of HMOX1 is the cause of increased ferroptosis during the development of diabetic atherosclerosis [19]. Growing evidence indicates that ferroptosis occurs mainly under conditions such as metabolic disorders and oxidative stress and that ferroptosis in immune or other cell types can modulate the immune response, thereby contributing to the pathogenesis of both sepsis and T2DM [20].The present study was initially inspired by two considerations: firstly, while the established literature acknowledges the role of ferroptosis in sepsis and the epidemiological association between T2DM and severe infection [21], we noted a distinct lack of direct evidence focusing on ferroptosis-related genes in the specific comorbid context of human sepsis and T2DM. Secondly, our clinical observations were consistent with this documented comorbidity [22]. It was this identified gap that prompted our study to explore potential underlying mechanisms. Our findings provide preliminary evidence suggesting ferroptosis as a plausible pathway, and based on our current knowledge, we believe this may be an early contribution to this particular area of inquiry.

Advances in modern sequencing technology and bioinformatics have enabled researchers to explore the interrelationships among diseases and the direct links in their pathogenesis using human samples, rather than relying solely on animal or cellular models [23]. This approach allows for the generation of more robust and convincing conclusions. In this study, we employed bioinformatics analysis combined with qRT-PCR validation to identify key genes and signaling pathways involved in sepsis and T2DM. Based on these findings, we hypothesized that ferroptosis-related genes (FRGs) may serve as shared genetic mediators contributing to the worse outcomes of sepsis in patients with T2DM. Using integrated bioinformatics analysis of public sepsis and T2DM GEO datasets, we aimed to identify and validate candidate FRGs, explore their immune microenvironment associations, and assess their diagnostic value in sepsis complicating T2DM.

## Materials and methods

### Data download

The Series Matrix File data file of GSE76894 was downloaded from NCBI GEO public data^1^, annotated as GPL570, and the expression profile data of 103 patients were included, of which control group 84 cases and 19 cases in the disease group. Download the Series Matrix File data file of GSE65682 from NCBI GEO public database, the annotated file is GPL13667, a total of 802 patients were included in the expression profile data, of which 42 cases were in the control group and 760 cases were in the disease group (Supplementary Table S1). All datasets were preprocessed to ensure data quality and consistency, including background correction, normalization, and batch effect removal.

### Differential expression and functional enrichment analysis

Differential expression analysis was conducted using the R package “Limma”. This method identified genes showing significant differential expression between disease samples and control groups. A significance threshold of p < 0.05 was applied. The log2 fold change cutoff was set at |log2FC| > 0 following iterative adjustments to retain a robust gene set for downstream analyses. DEGs were visualized using volcano plots and heatmaps. Functional annotations for the identified genes were explored with the R package “ClusterProfiler.” Gene Ontology and Kyoto Encyclopedia of Genes and Genomes analyses assessed associated biological themes and pathways. Terms and pathways with both p-values and corrected q-values below 0.05 were deemed statistically significant.

### WGCNA analysis

A weighted gene co-expression network was constructed using the WGCNA-R package. This approach identifies gene modules with highly correlated expression patterns. The analysis transformed a weighted adjacency matrix into a topological overlap matrix (TOM) to assess network connectivity. Hierarchical clustering was then applied to the TOM matrix, generating a clustering tree structure. Distinct branches of this tree correspond to different gene modules, with each module represented by a unique color. Genes were assigned to modules based on their expression pattern similarities, effectively grouping the entire gene set into multiple co-expression modules.

### Immune cell infiltration analysis

The single-sample gene set enrichment analysis (ssGSEA) was employed to evaluate immune cell composition within the microenvironment. This method quantifies 29 distinct human immune cell phenotypes, encompassing T cells, B cells, and natural killer cells. The analysis utilized a validated custom gene set (immune.gmt), widely applied in tumor immunology. This gene set integrates core immune cell signatures from Bindea et al [24]. With critical functional features, including immune checkpoints and cytolytic activity, from Danaher et al [25]. The ssGSEA algorithm estimated relative abundances of 29 immune cell types from the gene expression profiles. Spearman correlation analysis was subsequently performed to assess relationships between gene expression and immune cell infiltration levels. For data preprocessing, probe expression values corresponding to duplicate gene symbols were averaged to ensure gene symbol uniqueness. Genes showing zero average expression across all samples were excluded from the analysis, retaining only those with detectable expression signals.

### Transcriptional regulation analysis of key genes

Transcription factor prediction was conducted using the R package “RcisTarget.” All analyses in this package are grounded in motif-based assessments. The normalized enrichment score (NES) for each motif is calculated relative to the complete motif database. Additional annotation files were generated by leveraging motif similarity and corresponding gene sequences. The analytical process first involved calculating the area under the curve for each motif and gene set pair. This calculation derived from recovery curves generated through gene-set-to-motif sequencing. Subsequently, the NES for individual motifs was determined by comparing their area under the curve (AUC) values against the distribution of all motifs within the gene set.

### miRNA network construction

MicroRNAs (miRNAs) are short non-coding RNAs that mediate post-transcriptional regulation via mRNA degradation or translational repression. This study investigated potential miRNA regulation of candidate genes. Experimentally validated miRNAs targeting the key genes were identified through the miRcode database. A comprehensive miRNA-mRNA interaction network was subsequently constructed and visualized using Cytoscape software.

### Cmap drug prediction

The Connectivity Map (CMap) resource, developed by the Broad Institute [26], provides a gene expression profiling database that captures cellular responses to chemical perturbations. This platform enables the discovery of functional relationships between small molecules, genes, and disease states. The database comprises microarray data documenting expression changes induced by 1309 small molecule compounds across five human cell lines [27]. Treatment conditions vary substantially, incorporating different concentrations and exposure durations. This study utilized disease-specific differentially expressed genes to predict potential therapeutic compounds from the CMap database.

### Collection of patients and healthy controls

The study enrolled fifteen participants comprising five sepsis patients with T2DM comorbidity, five sepsis patients without diabetes, and five healthy controls. All patient recruitment was conducted through the Department of Emergency Internal Medicine at the First Affiliated Hospital of Bengbu Medical University (Supplementary Table S2). T2DM diagnosis followed the American Diabetes Association 2021 criteria [28], requiring meeting at least one of these laboratory parameters: random plasma glucose ≥11.1 mmol/L, 2-h OGTT plasma glucose ≥11.1 mmol/L, fasting plasma glucose ≥7.0 mmol/L, or glycated hemoglobin A1C ≥ 6.5%. The study protocol received approval from the Human Ethics Committee of the First Affiliated Hospital of Bengbu Medical University. All procedures conformed to the ethical principles outlined in the Helsinki Declaration.

### Total RNA extraction and qRT-PCR

Morning fasting peripheral blood samples (5 mL) were collected in ethylene diamine tetraacetic acid (EDTA) containing tubes from all participants. Plasma separation was achieved through centrifugation. Peripheral blood mononuclear cells (PBMCs) were subsequently isolated via Ficoll gradient centrifugation. Total RNA extraction utilized RNAiso Plus reagent (Takara, Japan). Reverse transcription was performed with the 5 × PrimeScript RT Master Mix (Takara, Japan). PCR amplification employed the TB Green PCR Core Kit (Takara, Japan) on a CFX96TM real-time system (Bio-Rad, United States). The housekeeping gene Beta-actin served as an internal reference for normalization. Relative mRNA expression levels were determined using the 2^ (−ΔΔCT) method. All primer sequences appear in Supplementary Table S3. Statistical analysis of qRT-PCR data involved ANOVA implementation in GraphPad Prism (v6.0). A threshold of p < 0.05 defined statistical significance.

### Statistical analysis

All statistical analyses were performed using R language (version 4.2.2). All statistical tests were two-sided and p < 0.05 was statistically significant.

## Results

### Identification of differential expression genes and functional enrichment analysis

**FIGURE 1:**
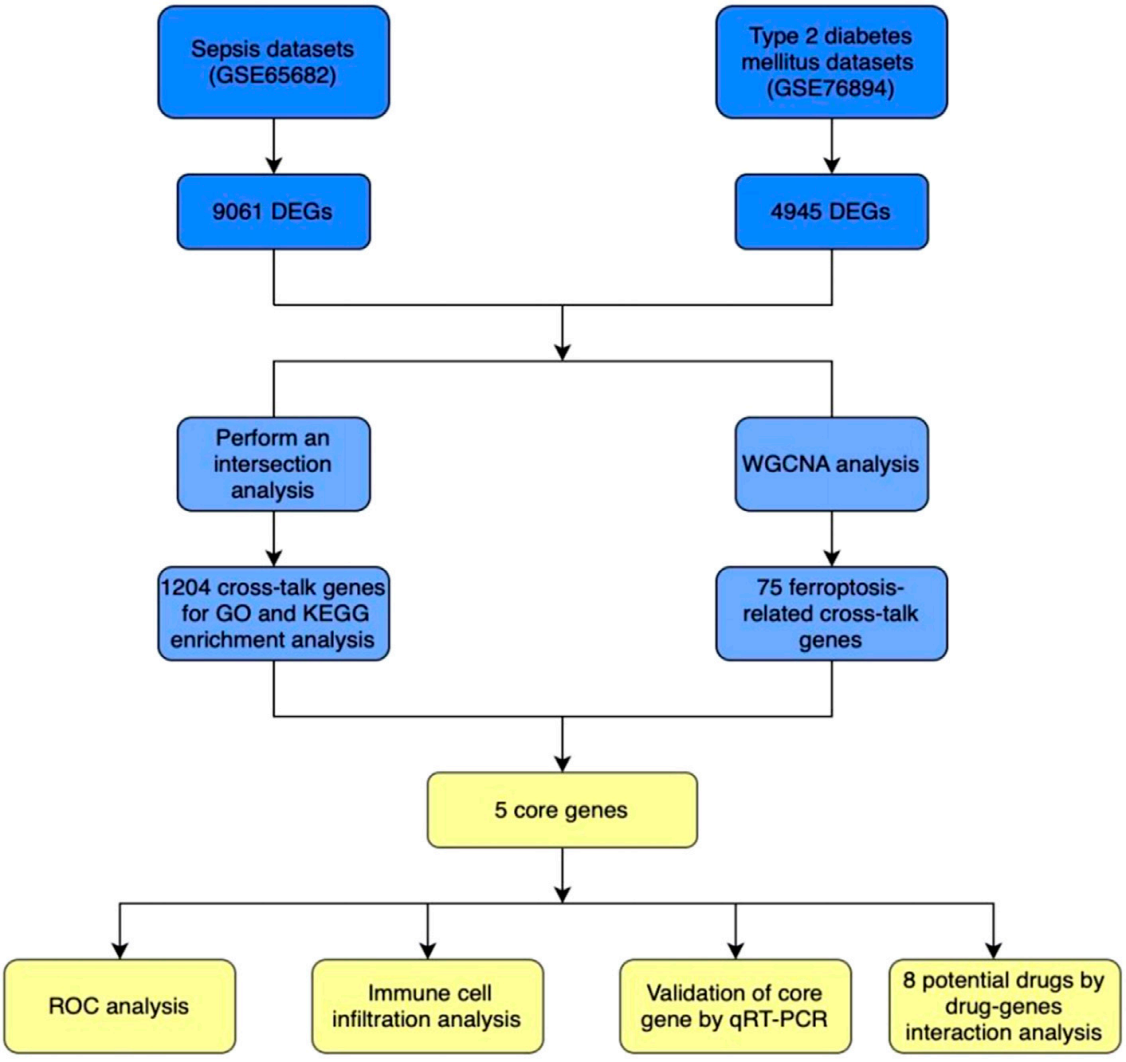
The detailed procedures of this study.

**FIGURE 2:**
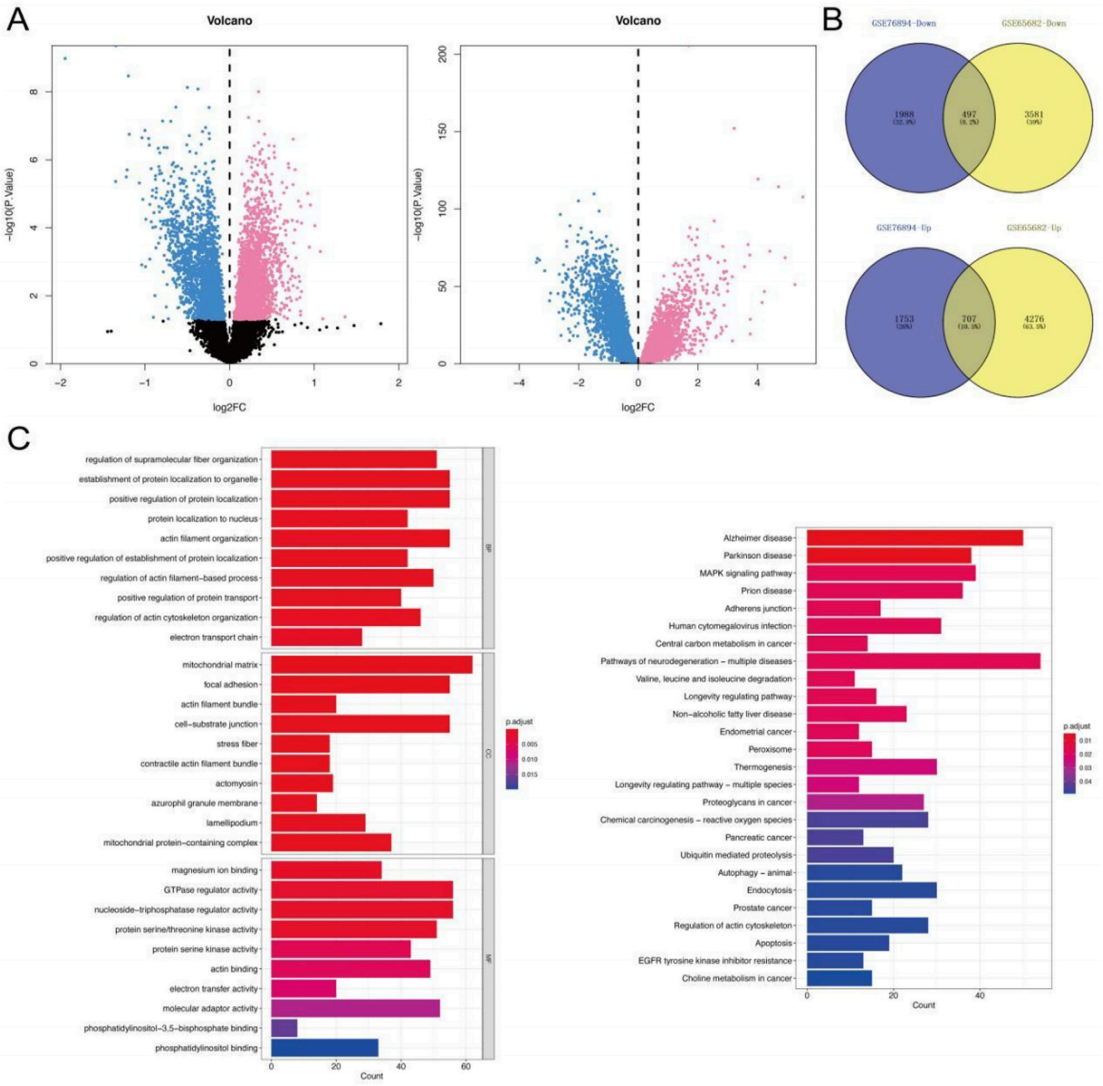
DEGs and Function enrichment analysis. **(A)** GSE76894 and GSE65682 datasets showed the Down and Up-regulated genes in Volcano map. (Up-regulated genes are marked in red and down-regulated genes in blue). **(B)** Venn diagrams of intersecting genes from two databases. **(C)** GO and KEGG enrichment analysis of DEGs. P < 0.05 is considered significant.

The overall workflow of this study is summarized in the Figure 1. Gene expression profiles were obtained from two publicly available datasets: GSE76894 (NCBI GEO) comprising 103 individuals (84 healthy controls and 19 T2DM patients), and GSE65682 (NCBI GEO) containing data from 802 sepsis patients (42 healthy controls and 760 disease cases). Differential gene expression analysis was performed using the Limma R package, with a significance threshold set at P < 0.05, ∣log2 FC∣ > 0. This analysis identified 4,945 DEGs in the T2DM dataset (2460 were upregulated, and 2485 were downregulated) and 9061 DEGs in the sepsis dataset (4983 upregulated and 4078 downregulated) (Figure 2A). Intersection analysis identified 1,204 shared DEGs (707 upregulated and 497 downregulated), highlighting genes commonly dysregulated in both conditions (Figure 2B).

Functional annotation of these intersecting genes was conducted via GO and KEGG enrichment analyses. GO terms revealed significant enrichment in processes such as regulation of supramolecular fiber organization, protein localization to organelles, and protein modification. KEGG pathway analysis highlighted involvement in key signaling pathways including the MAPK cascade, adherens junction formation, and peroxisomal β-oxidation. These findings underscore shared molecular mechanisms underlying T2DM and sepsis pathobiology (Figure 2C).

### Identification of key genes and diagnostic efficacy via WGCNA

**FIGURE 3:**
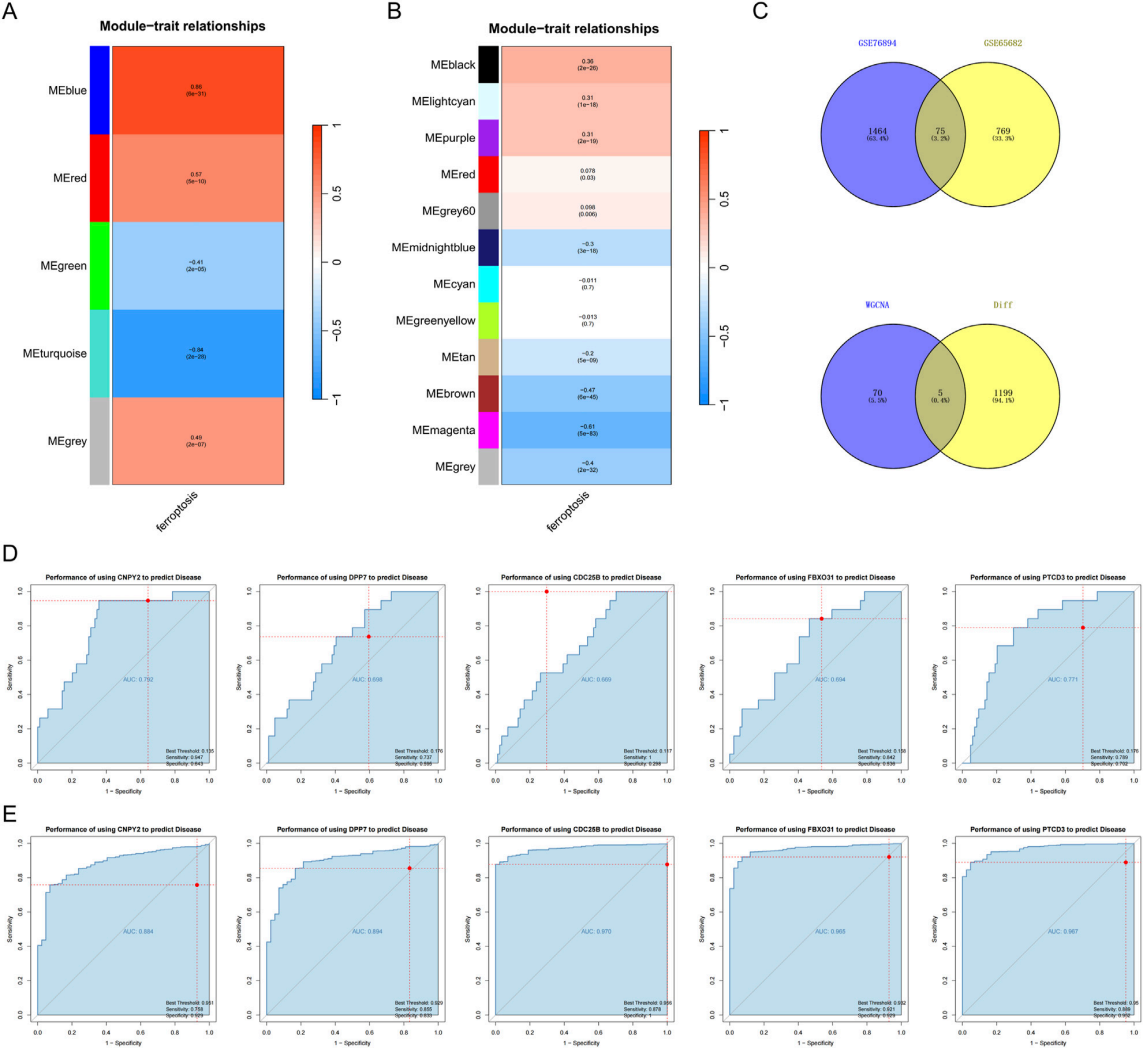
Key genes and the Diagnostic Efficacy. **(A)** Genes highly associated with T2DM in the GSE76894 database; **(B)** genes negatively associated with sepsis in the GSE65682 database; **(C)** Intersection of genes in the most relevant modules of the two database sets. **(D,E)** ROC curve analysis.

To systematically identify key genes associated with disease progression and assess their diagnostic potential, we conducted a comprehensive analysis using WGCNA on two independent datasets (GSE76894 and GSE65682). This approach enabled us to uncover shared molecular mechanisms underlying T2DM and sepsis. In the GSE76894 dataset, we determined an optimal soft-threshold power (β) of 17 to ensure the formation of a scale-free network topology, a hallmark of biological networks. Hierarchical clustering of the topological overlap matrix (TOM) revealed five distinct gene modules (Figure 3A). Notably, the blue module exhibited the highest correlation with T2DM (correlation coefficient = 0.86, p = 6e-31), suggesting its relevance to disease pathogenesis. In the GSE65682 dataset, a β value of 19 was selected to preserve network scale-free properties. This analysis yielded twelve gene modules (Figure 3B), with the magenta module showing the strongest negative correlation with sepsis (correlation coefficient = −0.61, p = 5e-83). We have supplemented the version of the R package used in this study in the Supplementary Table S4.

To identify overlapping genes between the two datasets, we performed an intersection analysis of the most disease-associated modules, resulting in 75 shared genes (Figure 3C). Further refinement by intersecting these genes with previously identified DEGs yielded five candidate genes: CDC25B, DPP7, FBXO31, PTCD3, and CNPY2 (Figure 3C), These genes emerged as promising biomarkers for both T2DM and sepsis, warranting further investigation into their functional roles.

To evaluate their diagnostic utility, we performed receiver operating characteristic (ROC) curve analysis. In the GSE65682 sepsis dataset, these genes showed high discriminative ability, with AUC values reaching 0.970 (CDC25B), 0.965 (FBXO31), and 0.967 (PTCD3) (Figures 3D,E). The specific AUC and NES thresholds have been provided in the Supplementary Table S5. These results suggest a strong discriminative ability of these genes for sepsis within this specific dataset. However, these results were obtained from a single dataset and require validation in independent cohorts before any clinical application can be considered.

### Deciphering the immune microenvironment and key gene correlations in T2DM

**FIGURE 4:**
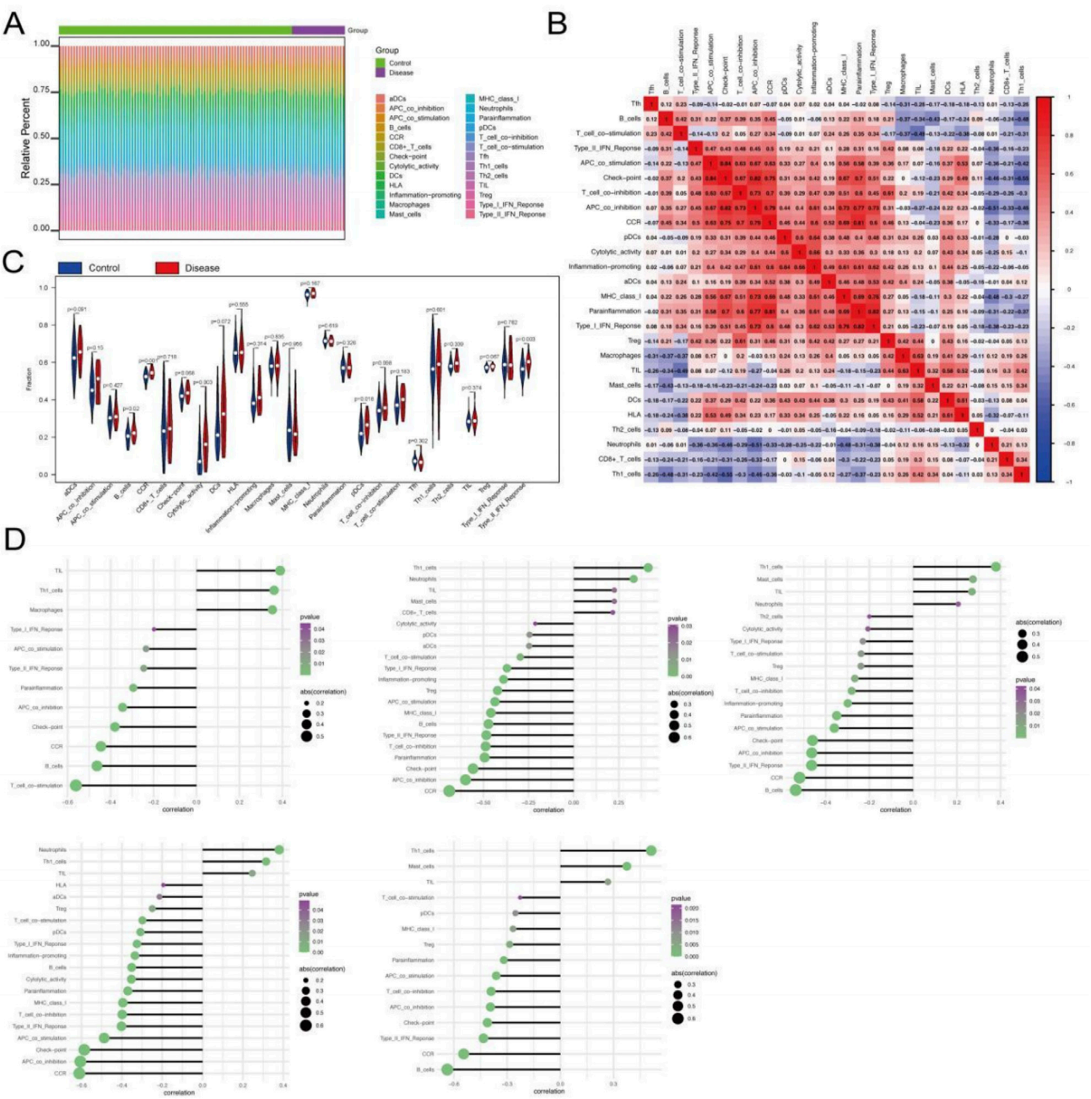
Relationship between genes and immune infiltration. **(A–C)** Immune cell infiltration differences between T2DM patients and controls. **(D)** Correlation between key genes and immune cell types.

The immune microenvironment, a dynamic interplay of immune cells, extracellular matrix components, growth factors, inflammatory mediators, and distinct physicochemical properties, is pivotal for shaping disease progression, diagnostic outcomes, and therapeutic responses. To investigate the role of key genes in T2DM progression, we conducted a comprehensive analysis of their associations with immune infiltration using the GSE76894 dataset. Quantitative assessment of immune cell composition across patients revealed significant differences in immune cell distribution between T2DM patients and healthy controls (Figures 4A,B). Specifically, T2DM patients exhibited elevated levels of B cells, chemokine receptor activity (CCR), cytolytic activity, plasmacytoid dendritic cells (pDCs), and Type II interferon response compared to controls (Figure 4C), indicating an active yet potentially dysregulated immune landscape.

We further examined correlations between the key genes and immune features. CDC25B was positively correlated with tumor-infiltrating lymphocytes (TILs), Th1 cells, and macrophages, while negatively correlated with T-cell co-stimulation, B cells, and CCR (Figure 4D). DPP7 was positively correlated with Th1 cells, neutrophils, and TILs, and negatively correlated with CCR, APC co-inhibition, and checkpoint proteins (Figure 4D). Similarly, FBXO31 demonstrated significant positive correlations with Th1_cells, Mast_cells, TIL, and significantly negatively correlated with B_cells, CCR, Type_II_IFN_Reponse (Figure 4D). PTCD3 was significantly positively correlated with Neutrophils, Th1_cells, TIL, and significantly negatively correlated with CCR, APC_co_inhibition, Check -point (Figure 4D). CNPY2 was significantly positively correlated with Th1_cells, Mast_cells, TIL, and significantly negatively correlated with B_cells, CCR, Type_II_IFN_Reponse (Figure 4D). Together, these results suggest that the key genes play multifaceted roles in shaping immune responses in T2DM, potentially contributing to immune dysregulation and disease pathogenesis.

### Immune microenvironment and key gene interactions in sepsis

**FIGURE 5:**
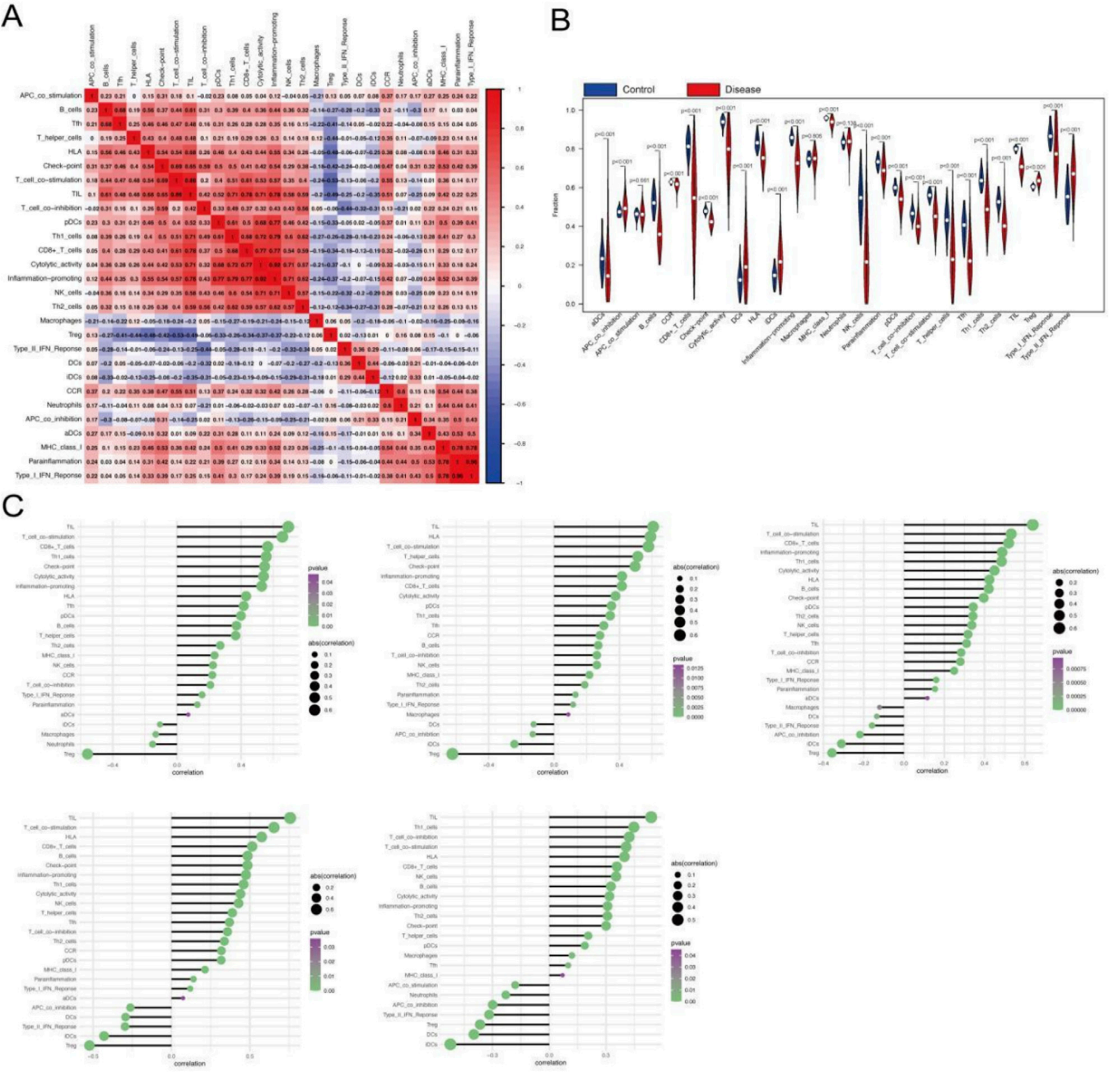
Relationship between genes and immune infiltration in the GSE65682 dataset. **(A,B)** Differential expression of immune cell infiltration between disease and healthy controls. **(C)** Correlation between key genes and immune cell types.

To investigate the role of key genes in modulating immune responses during sepsis, we analyed the GSE65682 dataset. Quantification of immune cell composition and examination of immune cell interactions revealed distinct immunological signatures in sepsis (Figure 5A). Compared to healthy controls, sepsis patients exhibited significantly elevated levels of Tregs (regulatory T cells), Type II interferon response, and immature dendritic cells (iDCs) (Figure 5B), suggesting a unique immune landscape in sepsis, characterized by augmented regulatory and inflammatory pathways, potentially reflecting the complex interplay between immune activation and systemic inflammation.

We further explored the relationships between the key genes and immune cell infiltration. CDC25B expression showed positive correlations with TILs, T-cell co-stimulation, and CD8^+^ T cells, while negatively correlated with Tregs, neutrophils, and macrophages (Figure 5C). Similarly, DPP7 was positively correlated with TILs, HLA (human leukocyte antigen), and T-cell co-stimulation, while negatively correlated with Tregs, iDCs, and APC co-inhibition (Figure 5C). Related patterns were observed for FBXO31, PTCD3, and CNPY2, which also demonstrated significant correlations with multiple immune cell subsets (Figure 5C), underscoring their collective involvement in sepsis immunopathology. These findings suggest that the identified key genes may help shape the immune microenvironment in sepsis, potentially influencing disease progression and immune regulation.

### Immunoregulatory roles of key genes in both T2DM and sepsis

**FIGURE 6:**
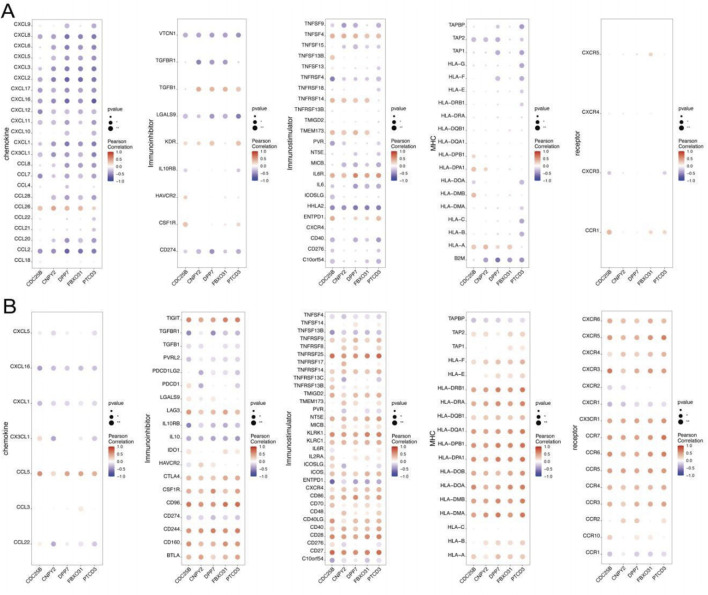
**(A,B)** Correlation between key genes and various immunomodulators in the TISIDB database.

To investigate the immunoregulatory roles of the identified key genes in T2DM and sepsis, we conducted an integrative analysis using the TISIDB database to examine their correlations with immunomodulatory factors—including immunosuppressants, immunostimulators, chemokines, and receptors. Results demonstrated that these genes exhibit intricate associations with immune cell infiltration levels and are likely involved in shaping the immune landscape of both diseases (Figures 6A,B). While the observed correlations between key genes and immune cell populations provide intriguing insights into potential immune mechanisms, we acknowledge that these findings are derived from computational deconvolution algorithms which have inherent limitations. The correlations may reflect both biological relationships and methodological constraints. However, the consistency of patterns across multiple genes and the alignment with known biology of these diseases (e.g., the elevated levels of Tregs and Type II interferon response in sepsis) suggest that at least some of these associations are biologically meaningful. Future studies with single-cell RNA sequencing or flow cytometry validation are needed to confirm these findings and distinguish true biological signals from potential computational artifacts. The elevated levels of regulatory T cells and type II interferon responses in sepsis patients reported in relevant literature can provide certain reference basis [29]. Further validation using single-cell RNA sequencing or flow cytometry will be essential to confirm these relationships and distinguish true immune regulatory mechanisms from analytical noise.

### Regulatory mechanisms underlying key gene expression

**FIGURE 7:**
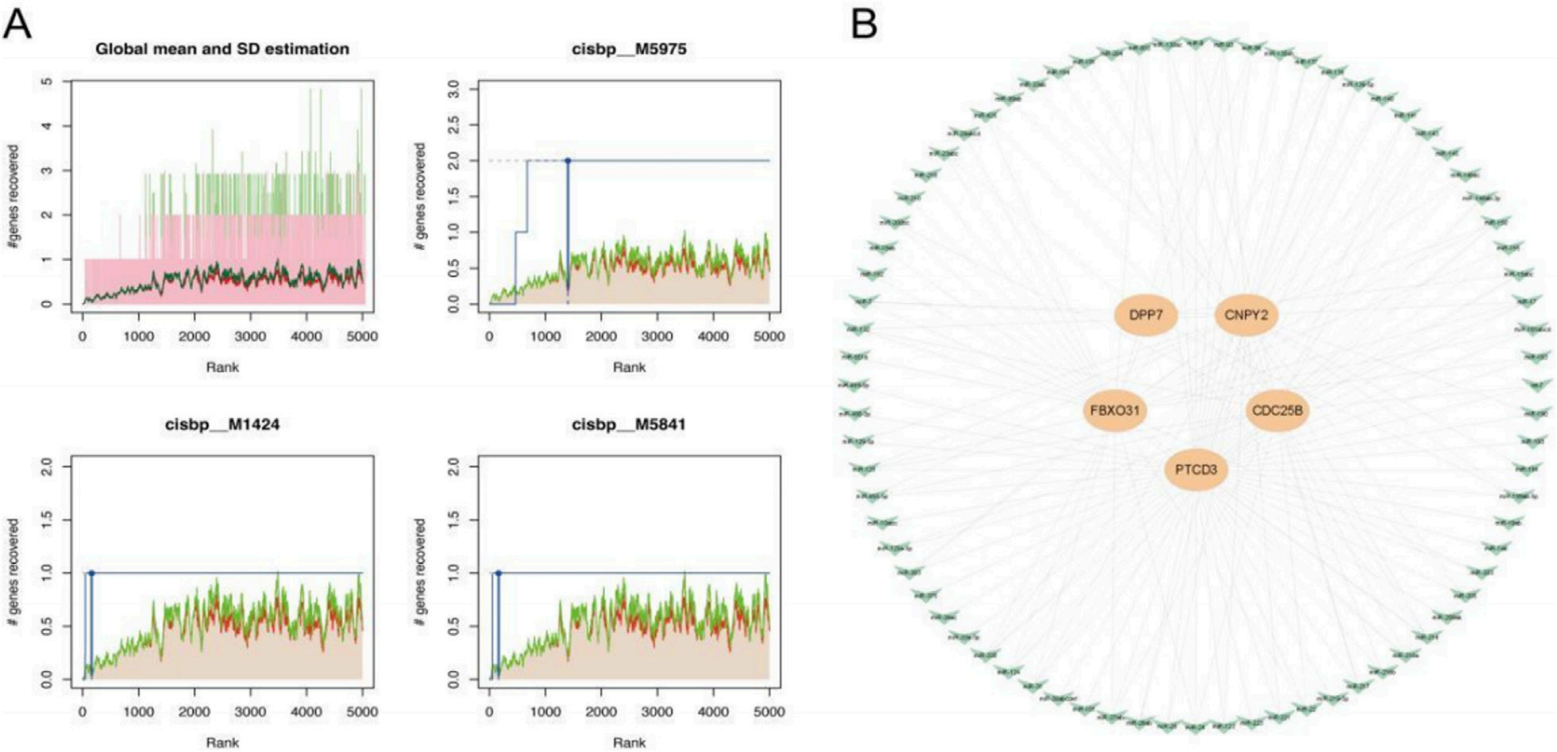
**(A)** TF enrichment analysis based on cumulative recovery curve and motif annotation; **(B)** miRNA-mRNA interaction.

We next investigated the potential regulatory mechanisms of the five key genes through transcription factor (TF) and miRNA analyses. TF enrichment analysis revealed cisbp_M5975 as the top motif (NES = 8.73), suggesting a primary role in transcriptional regulation (Figure 7A). Concurrently, miRcode-based screening identified 76 miRNAs forming 156 regulatory pairs with the key genes, visualized as a complex network in Figure 7B. These findings uncover transcriptional and post-transcriptional regulatory layers, highlighting potential intervention points for T2DM and sepsis.

### Analysis of disease-associated genes and drug prediction in type 2 diabetes and sepsis

**FIGURE 8:**
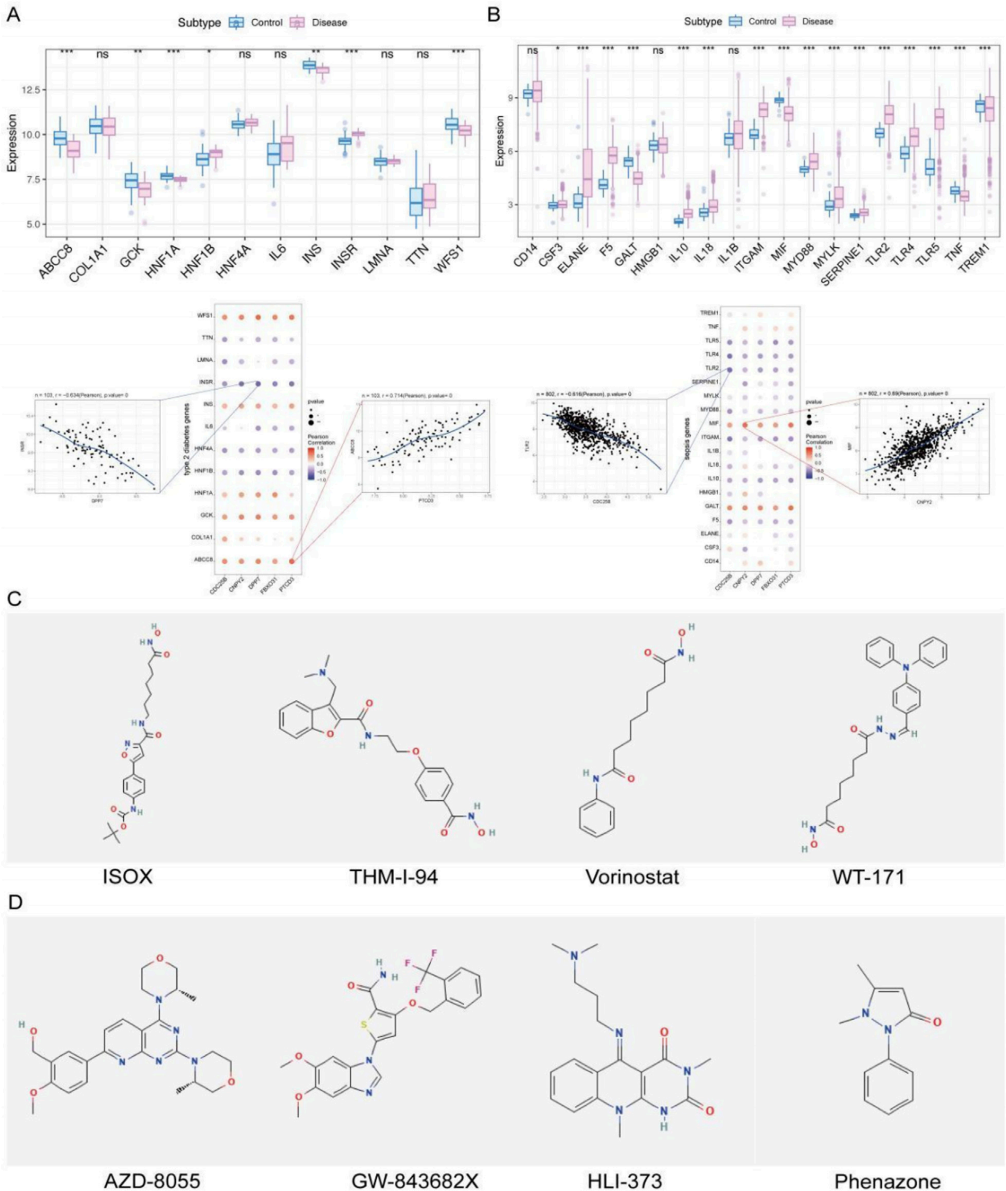
**(A,B)** Expression patterns of disease-related genes in T2DM and sepsis, along with correlations between key genes and disease-related genes. **(C,D)** Drug repurposing predictions using the CMap database for T2DM and sepsis, showing candidate drugs with negative correlations to disease-specific expression profiles.

We further explored the expression patterns of disease-associated genes by retrieving T2DM-related genes from the GeneCards database^2^. Analysis of top-ranked T2DM genes revealed significant expression differences between control and disease groups for genes including ABCC8, GCK, HNF1A, HNF1B, INS, INSR, and WFS1 (Figure 8A). Notably, PTCD3 showed a strong positive correlation with ABCC8 (Pearson r = 0.714), while DPP7 was negatively correlated with INSR (Pearson r = −0.634) (Figure 8A). These findings suggest potential co-regulatory mechanisms involving these genes in T2DM pathogenesis.

Similarly, sepsis-related genes obtained from GeneCards were analyzed across patient groups. Genes such as CSF3, ELANE, F5, and GALT exhibited significant differential expression between sepsis patients and controls (Figure 8B). Correlation analysis showed that CNPY2 was positively correlated with MIF (Pearson r = 0.69), while CDC25B was negatively correlated with TLR2 (Pearson r = −0.616) (Figure 8B). These correlations suggest that the key genes may be involved in shared molecular pathways influencing both T2DM and sepsis pathogenesis.

To identify potential therapeutic interventions, we used the CMap database to predict drugs targeting the DEGs in T2DM and sepsis. CMap drug prediction was performed using differentially expressed genes derived from the expression profiles of the two diseases. Genes were selected based on log2FC, with the top 150 up-regulated genes and the top 150 down-regulated genes being used for the analysis. For T2DM, whose perturbational gene expression profiles were negatively correlated with the disease signature, including ISOX, THM-I-94, Vorinostat, and WT-171 (Figure 8C)—corresponding to the top four compounds with the lowest connectivity scores (denoted as “Score”) in the Supplement2. Similarly, for sepsis, analysis highlighted AZD-8055, GW-843682X, HLI-373, and Phenazone as potential therapeutics, corresponding to the top compounds in the Supplement3 and showing strong negative correlations with the sepsis expression profile (Figure 8D), suggesting their potential to attenuate the septic condition.

### Validation of core gene expression by qRT-PCR

**FIGURE 9:**
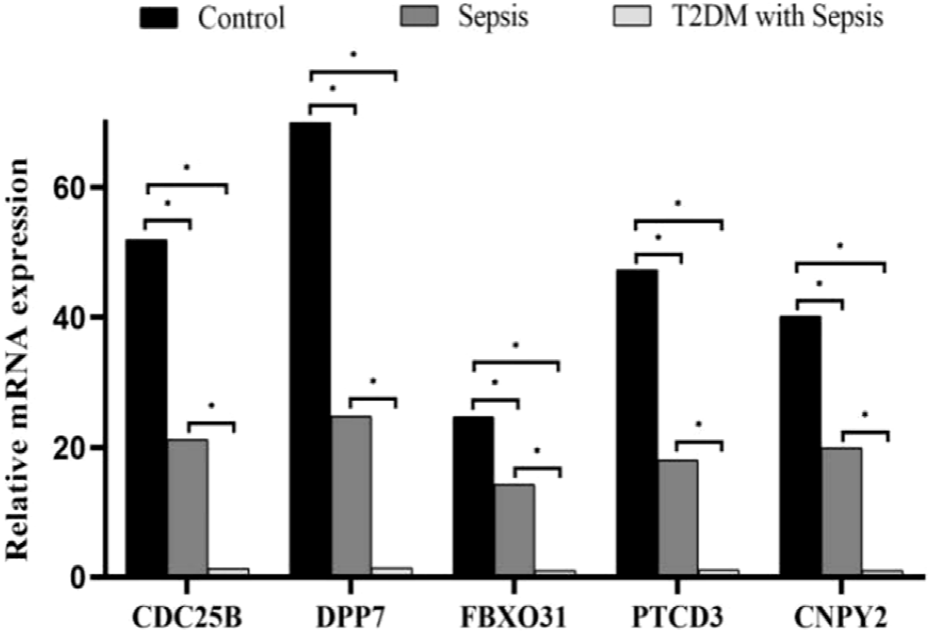
qRT-PCR validation of core gene expression levels across different groups. Relative expression levels of CDC25B, DPP7, FBXO31, PTCD3 and CNPY25 were compared between control, sepsis, and sepsis with T2DM groups. Data were analyzed using one-way ANOVA, with P < 0.05 indicating statistical significance (*P < 0.05).

To experimentally validate our findings, we quantified the expression levels of the shared genes across different sample groups using qRT-PCR (Figure 9). The results demonstrated a significant reduction in the mRNA levels of these genes in the sepsis group compared to the control group (P < 0.05). Notably, the expression was further decreased in patients with sepsis complicated by T2DM (P < 0.05).

## Discussion

### The burden of sepsis and its association with T2DM

Sepsis accounts for nearly 20% of global annual deaths, with more than 20 deaths occurring per minute due to sepsis-related complications [30]. It remains one of the most severe acute complications in critically ill patients, particularly those with infections [31]. Beyond its acute effects, sepsis is associated with poor long-term outcomes, including high hospitalization costs, prolonged recovery periods, and significant health burdens, which severely impacts patients’ quality of life. This highlights the critical need for early prevention and treatment of sepsis.

Patients with diabetes mellitus face a sixfold higher risk of sepsis compared to non-diabetic individuals [32]. Notably, over 20% of sepsis patients also have diabetes mellitus [33]. High blood glucose levels and an increase in the coefficient of variation of blood glucose are significantly associated with mortality from sepsis in the ICU, and the impact on death increases with the severity of sepsis [34].T2DM, which constitutes more than 90% of diabetes cases, is primarily characterized by insulin resistance and β-cell dysfunction, with the latter playing a central role in disease progression. β-cell dysfunction occurs more frequently in critically ill patients because they need to overcome the prevalent insulin-resistant state. Moreover, in patients with severe illness, more than four organ failures or death, β-cell dysfunction often occurs from the very beginning [35].Understanding the molecular and immune mechanisms linking sepsis and T2DM is critical for developing targeted therapies and improving outcomes for patients with these conditions.

### Immune dysregulation in sepsis and T2DM

The high morbidity and mortality rates associated with sepsis, particularly among diabetic patients, highlight the importance of understanding the underlying immune dysregulation. Our findings indicate that T2DM may elevates the risk of sepsis, consistent with previous studies indicating that diabetic patients are 2–6 times more likely to develop sepsis than non-diabetic individuals. In T2DM, chronic hyperglycemia contributes to immune dysfunction, increasing susceptibility to infections such as sepsis. Our analysis of immune infiltration in T2DM revealed elevated levels of B cells, chemokine receptor activity (CCR), and Type II interferon response, suggesting an enhanced yet dysregulated immune response.

Sepsis itself is characterized by a complex immune response, often involving both hyperinflammation and immunosuppression. In our analysis of the GSE65682 dataset, we observed increased levels of Tregs, Type II interferon response, and iDCs, reflecting the dual nature of immune dysregulation in sepsis. These findings align with previous studies demonstrating that sepsis could lead to both immune hyperactivity (resulting in tissue damage) and immune suppression (increasing susceptibility to secondary infections).

### Key genes involved in sepsis and T2DM pathogenesis

Differential gene expression and co-expression network analyses identified five key genes—CDC25B, DPP7, FBXO31, PTCD3, and CNPY2—that play critical roles in immune response and cellular regulation in sepsis and T2DM.

CDC25B, a key member of the cell division cycle 25 family, is a phosphoprotein essential for cell cycle regulation, particularly the G2/M transition. It was significantly correlated with immune cell populations such as tumor-infiltrating lymphocytes, Th1 cells, and macrophages in sepsis patients, suggesting a role beyond proliferation to immune regulation. Polypyrimidine tract-binding protein 1 (PTBP1), an RNA-binding protein expressed throughout B-cell development, regulates CDC25B mRNA abundance and splicing, further implicating CDC25B in B-cell development and immune function [36]. Our findings suggest that CDC25B may serve as a central mediator of immune cell differentiation and function in sepsis.

DPP7 is a serine protease, also known as quiescent cellular proline dipeptidase (QPP, DPP2, DPPII), which is a proline-cleaved aminopeptidase, a dipeptidyl peptidase capable of removing the N-terminal dipeptide, and edits proteins that are soluble proteins [37]. Knockdown of DPP7 increased apoptosis, and complete knockout is embryonically lethal in mice. DPP7 has been implicated in immune responses and lymphocyte apoptosis [38, 39]. In our study, DPP7 expression positively correlated with immune activation markers (e.g., T-cell co-stimulation, HLA expression) and negatively with immunosuppressive factors (e.g., Tregs, APC co-inhibition), suggesting a dual role in immune regulation during sepsis and T2DM.

FBXO31, an E3 ubiquitin ligase, is well-known for its role in the DNA damage response where it mediates Cyclin D1 degradation to halt the cell cycle. Notably, a groundbreaking recent study [40] has redefined its function, revealing that FBXO31 also serves as a crucial surveillance mechanism for oxidative protein damage by targeting C-terminal amidated proteins for degradation. Given the central role of oxidative stress in sepsis pathogenesis, we hypothesize that FBXO31 may contribute to immune regulation during sepsis by maintaining proteostasis in immune cells. Specifically, we propose that FBXO31 helps clear oxidatively damaged proteins, thereby preserving cellular function and mitigating immune dysfunction. This proposed mechanism, however, remains a testable hypothesis requiring further experimental validation.

PTCD3, a mitochondrial ribosomal protein, was associated with both immunosuppressive and immunostimulatory factors in sepsis, linking mitochondrial dysfunction to immune dysregulation [41]. Given that mitochondrial dysfunction is a hallmark of ferroptosis, the association between PTCD3 and immune cell regulation may provide a novel mechanism by which ferroptosis contributes to immune suppression and organ dysfunction in sepsis patients.

### Ferroptosis as a central mechanism in sepsis and T2DM

Ferroptosis, a form of iron-dependent regulated cell death driven by lipid peroxidation, has emerged as a critical mechanism in the progression of sepsis and T2DM. Iron metabolism is closely linked to β-cell function, participating in insulin secretion, proliferation, differentiation, and glucose metabolism. Dysregulated iron metabolism and ROS accumulation contribute to β-cell loss via ferroptosis. In the context of sepsis, ferroptosis is associated with pathogen-induced inflammatory responses, further connecting iron dysregulation to immune-mediated cell death [42, 43]. Evidence from animal models supports its role: AMPK activation reduced ferroptosis in the hippocampus of mice with diabetes, improving cognitive ability [44]. And nobiletin, a plant-based polymethoxy flavone, can regulate the composition of the intestinal microbiota in septic mice [45]. By modulating the intestinal microbiota, it can alleviate ferroptosis in liver injury caused by sepsis. Our findings further support that ferroptosis may play a key role in the progress of both conditions, particularly in the context of chronic inflammation and oxidative stress. Key genes identified were enriched in ferroptosis-related pathways such as peroxisome signaling, which regulates oxidative stress.

The interplay among iron metabolism, ROS accumulation, and immune dysfunction underscores the importance of ferroptosis in sepsis, especially in diabetic patients. Chronic hyperglycemia predisposes individuals to oxidative stress, exacerbating inflammation and promoting ferroptosis, likely contributing to the increased mortality observed in diabetic sepsis patients.

### Implications for therapeutic interventions

Using the CMap database, we identified several potential therapeutic targeting dysregulated pathways in sepsis and T2DM. Compounds such as ISOX and Vorinostat—which has been reported to exhibit efficacy in anti-tumor and anti-epileptic contexts. Notably, Vorinostat, as a histone deacetylase inhibitor, is crucial for the regulation of ferroptosis [46]. THM-I-94 were identified as potential modulators of T2DM-associated gene dysregulation, while drugs like AZD-8055 and HLI-373 showed strong negative correlations with the sepsis gene expression profile, suggesting their potential to attenuate or reverse disease progression in sepsis patients.

In addition, studies have shown that Echinacea extract can be used to improve the immune system and treat respiratory symptoms caused by bacterial infections [47].Ibudilast can act as an inhibitor and bind to phosphodiesterase 4 (PDE4), a new target for inflammatory diseases, to achieve the effect of inhibiting inflammatory responses [48]. The identification of these compounds offers promising avenues for therapeutic intervention.

This study offers important insights into the molecular mechanisms linking sepsis and T2DM, but several limitations should be acknowledged. First, our findings are derived exclusively from publicly available datasets, which may introduce batch effects, platform-specific biases, or demographic limitations that affect generalizability. Second, the immune infiltration estimates were generated through computational deconvolution methods (e.g., CIBERSORT), which infer rather than directly measure immune cell abundances and should be interpreted with caution. Functional experiments are required to confirm these observations. Additionally, while computational methods provided valuable estimates of potential therapeutic drugs, more precise experimental approaches and clinical testing are needed. Finally, the emerging role of ferroptosis in these conditions also requires further exploration. The complex interplay between sepsis, T2DM, and other comorbidities complicates the interpretation of results, highlighting the need for deeper mechanistic studies and validation in diverse patient populations.

To address these limitations mechanistically, we propose a multi-phase experimental framework for future investigation. *In vitro*, we will measure established ferroptosis markers in blood or tissue samples from septic patients with and without T2DM, correlating these with hub gene expression and clinical parameters to validate the pathophysiological relevance of our findings. Then primary human immune cells will be cultured under high-glucose conditions and subjected to genetic perturbation (e.g., siRNA-mediated knockdown or overexpression) of key hub genes, combined with pharmacological modulation of ferroptosis (including ferroptosis inhibitors (e.g., ferrostatin-1, liproxstatin-1)). These experiments will directly assess effects on ferroptotic markers—such as lipid peroxidation, GPX4 activity, and ACSL4 expression—along with functional immune readouts. *In vivo*, the cecal ligation and puncture model will be employed in diabetic (e.g., db/db) mice, integrating tissue-specific knockout approaches with treatment using candidate compounds to evaluate outcomes including survival, organ injury, and immune status. Finally, the intricate interplay among sepsis, T2DM, and associated comorbidities underscores the critical need for such in-depth mechanistic exploration.

## Conclusion

In conclusion, our study indicates the potential importance of key genes including CDC25B, DPP7, FBXO31, and PTCD3 in the shared pathogenesis of sepsis and T2DM. These genes may play a key role in immune regulation, cell cycle control, and ferroptosis, suggesting their promise as candidate therapeutic targets. Through our analysis of the potential molecular connections between these conditions, we provide a conceptual foundation for advancing precision medicine strategies aimed at improving outcomes in patients with sepsis, particularly those with comorbid diabetes. Future research should prioritize experimental validation of the identified candidate targets and further elucidate the functional role of ferroptosis in the progression of sepsis and T2DM.

## Data Availability

The data utilized in this study were sourced from the NCBI GEO database, accessible at https://www.ncbi.nlm.nih.gov/geo/info/datasets.html.

## Footnotes

1
https://www.ncbi.nlm.nih.gov/geo/info/datasets.html

2
https://www.genecards.org/
