# Supplementary material for: Bioinformatics-based screening and validation of ferroptosis-related genes in sepsis and type 2 diabetes mellitus
Source: Exp Biol Med (Maywood). 2025 Oct 21;250:10612. doi: 10.3389/ebm.2025.10612 (PMC12583109; doi:10.3389/ebm.2025.10612)
Supplement: Supplementary file 1 [file Table3.doc]

Rank Score Type ID Name Description

8541 -94.72 cp BRD-K46937689 phenazone Cyclooxygenase inhibitor

8534 -91.54 cp BRD-K17349619 HLI-373 MDM inhibitor

8531 -90.8 cp BRD-K69932463 AZD-8055 MTOR inhibitor

8528 -89.49 cp BRD-K90382497 GW-843682X PLK inhibitor

8521 -87.76 cp BRD-K64451768 GANT-58 GLI antagonist

8519 -87.52 cp BRD-K67445247 flurofamide Urease inhibitor

8515 -85.88 cp BRD-K83837640 JNJ-26854165 HDAC inhibitor

8513 -85.8 cp BRD-A74980173 gatifloxacin Bacterial DNA gyrase inhibitor

8512 -85.6 cp BRD-A53952395 prilocaine Local anesthetic

8511 -85.31 cp BRD-A39230911 chlorphensin Muscle relaxant

8487 -82.43 cp BRD-K62965247 tipifarnib-P2 farnesyltransferase inhibitor

8485 -82.29 cp BRD-K67566344 KU-0063794 MTOR inhibitor

8477 -80.72 cp BRD-K02407574 parbendazole Tubulin inhibitor

8471 -78.54 cp BRD-A59303141 quinethazone Thiazide diuretic

8468 -77.65 cp BRD-K51751936 alfadolone GABA receptor agonist

8466 -77.28 cp BRD-K23192422 lestaurtinib FLT3 inhibitor

8463 -77.13 cp BRD-A82096673 KU-C103428N CDC inhibitor

8459 -76.91 cp BRD-K19554809 MK-212 Serotonin receptor agonist

8457 -76.63 cp BRD-K69688083 mestinon Cholinesterase inhibitor

8450 -75.11 cp BRD-K51967704 BIIB021 HSP inhibitor

8446 -74.39 cp BRD-K70577657 H-9 PKA inhibitor

8445 -74.33 cp BRD-K55301415 abiraterone "17,20 lyase inhibitor"

8442 -73.73 cp BRD-A78322124 dobutamine Adrenergic receptor agonist

8441 -73.41 cp BRD-A12016240 LY-278584 Serotonin receptor antagonist

8423 -69.49 cp BRD-K96527333 dehydroisoandosterone GABA receptor modulator

8416 -68.74 cp BRD-K02953697 naringin Cytochrome P450 inhibitor

8410 -67.51 cp BRD-A74904029 EI-231 Casein kinase inhibitor

8403 -66.67 cp BRD-A14798026 mestranol Estrogen receptor agonist

8401 -66.4 cp BRD-K77793136 hydroxyfasudil Rho associated kinase inhibitor

8398 -65.91 cp BRD-K88560311 rucaparib PARP inhibitor

8393 -65.52 cp BRD-K54665485 R-59022 Diacylglycerol kinase inhibitor

8382 -63.85 cp BRD-A48261811 argatroban Thrombin inhibitor

8370 -62.41 cp BRD-A47513740 calyculin Protein phosphatase inhibitor

8359 -60.52 cp BRD-A81233518 glycopyrrolate Acetylcholine receptor antagonist

8348 -59.26 cp BRD-A62057054 MDL-11939 Serotonin receptor antagonist

8345 -58.87 cp BRD-K41731458 triclosan Enoyl-[acyl-carrier-protein] reductase [NADH] inhibitor

8344 -58.86 cp BRD-K32977963 eugenol Androgen receptor antagonist

8342 -58.65 cp BRD-K52751261 TAK-715 p38 MAPK inhibitor

8336 -57.57 cp BRD-K48935217 epothilone Microtubule inhibitor

8337 -57.57 cp BRD-K35483542 alitretinoin Retinoid receptor agonist

8335 -57.52 cp BRD-K63195589 tipifarnib Farnesyltransferase inhibitor

8334 -57.35 cp BRD-A78377521 monastrol Kinesin-like spindle protein inhibitor

8327 -56.32 cp BRD-K40530731 hyoscyamine Acetylcholine receptor antagonist

8324 -56.19 cp BRD-K98763141 niflumic-acid Cyclooxygenase inhibitor

8321 -55.99 cp BRD-K79124250 ioxaglic-acid Radiopaque medium

8318 -55.86 cp BRD-A75517195 thiazolopyrimidine CDC inhibitor

8317 -55.79 cp BRD-A41301928 bongkrek-acid "Mitochondrial ADP, ATP translocase inhibitor"

8314 -55.58 cp BRD-K22009844 phenprobamate Muscle relaxant

8313 -55.47 cp BRD-K61177364 NBI-27914 CRF receptor antagonist

8312 -55.44 cp BRD-K32330832 VER-155008 HSP inhibitor

8311 -55.39 cp BRD-K78084463 W-12 Calmodulin antagonist

8308 -54.95 cp BRD-K24526313 levcromakalim Potassium channel activator

8307 -54.87 cp BRD-K18742343 H-8 PKA inhibitor

8306 -54.86 cp BRD-A74907996 equol Estrogen receptor agonist

8300 -54.15 cp BRD-K01493881 apigenin Casein kinase inhibitor

8299 -53.99 cp BRD-K72034655 peucedanin Apoptosis stimulant

8296 -53.69 cp BRD-K50388907 fenofibrate PPAR receptor agonist

8288 -53.14 cp BRD-K15108141 gemcitabine Ribonucleotide reductase inhibitor

8284 -52.93 cp BRD-A76093993 cromakalim Potassium channel activator

8282 -52.58 cp BRD-K20285085 fostamatinib SYK inhibitor

8278 -52.35 cp BRD-A01317026 "7,8-dihydro-L-biopterin" Dihydroneopterin aldolase inhibitor

8279 -52.35 cp BRD-K80639402 SB-258585 Serotonin receptor antagonist

8270 -51.11 cp BRD-A76279427 myriocin Serine palmitoyltransferase inhibitor

8271 -51.11 cp BRD-K20714604 RS-56812 Serotonin receptor partial agonist

8266 -50.85 cp BRD-A62035778 scopolamine Acetylcholine receptor antagonist

8265 -50.69 cp BRD-K10852020 tolcapone Catechol O methyltransferase inhibitor

8259 -50.47 cp BRD-K26573499 DMAB-anabaseine Adrenergic receptor agonist

8251 -50.13 cp BRD-A53576514 orphenadrine Acetylcholine receptor antagonist

8247 -50.01 cp BRD-K84266862 BRL-50481 Phosphodiesterase inhibitor

8246 -49.68 cp BRD-K08589866 linsitinib IGF-1 inhibitor

8245 -49.62 cp BRD-A67981824 cefotaxime Bacterial cell wall synthesis inhibitor

8243 -49.59 cp BRD-K84085265 CG-930 JNK inhibitor

8236 -49.18 cp BRD-K19796430 erismodegib Smoothened receptor antagonist

8235 -49.08 cp BRD-K97354755 SU-6656 SRC inhibitor

8234 -49.05 cp BRD-K14920963 erythrosine Coloring agent

8227 -48.64 cp BRD-K26801045 pipamperone Dopamine receptor antagonist

8224 -48.45 cp BRD-K82746043 navitoclax BCL inhibitor

8221 -48.25 cp BRD-K68620903 dydrogesterone Progesterone receptor agonist

8219 -48.18 cp BRD-K82823076 RO-15-4513 GABA benzodiazepine site receptor inverse agonist

8217 -48.05 cp BRD-M40783228 mesna Antioxidant

8214 -47.92 cp BRD-K45435259 SCH-23390 Dopamine receptor antagonist

8212 -47.62 cp BRD-A03623303 metoprolol Adrenergic receptor antagonist

8210 -47.59 cp BRD-K40990712 hexamethyleneamiloride Sodium/hydrogen antiport inhibitor

8205 -47.16 cp BRD-K51941867 LM-1685 Cyclooxygenase inhibitor

8198 -46.83 cp BRD-A84702196 penicillin Bacterial cell wall synthesis inhibitor

8195 -46.71 cp BRD-A15034104 bergenin Interleukin inhibitor

8185 -46.03 cp BRD-K87990216 piretanide Glucocorticoid receptor agonist

8183 -45.82 cp BRD-A48237631 mitomycin-c DNA alkylating agent

8180 -45.51 cp BRD-K94176593 TWS-119 Glycogen synthase kinase inhibitor

8177 -45.29 cp BRD-K91733562 secoisolariciresinol Antioxidant

8176 -45.23 cp BRD-A85025557 NCS-382 GABA receptor antagonist

8173 -45.15 cp BRD-A81795050 U-18666A Oxidosqualene cyclase inhibitor

8172 -45.12 cp BRD-K60298136 ITE Aryl hydrocarbon receptor agonist

8171 -45.06 cp BRD-K56596464 QX-314 Sodium channel blocker

8169 -45.03 cp BRD-K20152659 gamma-homolinolenic-acid Cholesterol inhibitor

8168 -44.96 cp BRD-U44700465 HG-5-88-01 Protein kinase inhibitor

8165 -44.32 cp BRD-K32536677 AGK-2 SIRT inhibitor

8161 -43.96 cp BRD-K26373640 IKK-2-inhibitor IKK inhibitor

8142 -42.3 cp BRD-K49456190 prima-1-met thioredoxin inhibitor

8141 -42.27 cp BRD-K50135270 GBR-12935 Dopamine uptake inhibitor

8138 -42.2 cp BRD-K27871032 lysergol Ergoline alkaloid

8139 -42.2 cp BRD-K52512893 SC-19220 Prostanoid receptor antagonist

8134 -42.06 cp BRD-A65145453 ATPA Glutamate receptor agonist

8130 -41.92 cp BRD-K19533706 tranilast Angiogenesis inhibitor

8127 -41.83 cp BRD-K14329163 BAY-K8644 Calcium channel activator

8120 -41.13 cp BRD-A15297126 fluocinonide Glucocorticoid receptor agonist

8113 -40.5 cp BRD-K13571841 pepstatin Aspartic protease inhibitor

8109 -40.23 cp BRD-K08132273 tyrphostin EGFR inhibitor

8108 -40.18 cp BRD-K08206212 entecavir Reverse transcriptase inhibitor

8105 -40.14 cp BRD-K13211965 L-741742 Dopamine receptor antagonist

8096 -39.77 cp BRD-K13356952 methazolamide Carbonic anhydrase inhibitor

8095 -39.66 cp BRD-K29733039 deforolimus MTOR inhibitor

8092 -39.32 cp BRD-A09161221 nomilin HSP inhibitor

8090 -39.09 cp BRD-K14696368 "9-methyl-5H-6-thia-4,5-diaza-chrysene-6,6-dioxide" NFkB pathway inhibitor

8091 -39.09 cp BRD-K63913457 eicosatrienoic-acid Vasodilator

8089 -39.06 cp BRD-A26845397 isamoltan Adrenergic receptor antagonist

8084 -38.79 cp BRD-A99571536 dubinidine Anti-epileptic

8082 -38.65 cp BRD-K36395411 SB-206553 Serotonin receptor antagonist

8081 -38.59 cp BRD-A23072235 pheniramine Histamine receptor antagonist

8078 -38.53 cp BRD-K54416256 methimazole Antithyroid

8077 -38.37 cp BRD-K30677119 PP-30 RAF inhibitor

8076 -38.3 cp BRD-K34098590 tienilic-acid Sodium/potassium/chloride transporter inhibitor

8066 -37.71 cp BRD-U01690642 acetyl-geranyl-cysteine Isoprenylated protein methylation inhibitor

8065 -37.67 cp BRD-K46862739 metyrapone Cytochrome P450 inhibitor

8061 -37.2 cp BRD-K70490179 rimcazole Sigma receptor antagonist

8059 -37.13 cp BRD-K37312348 kenpaullone CDK inhibitor

8056 -37.06 cp BRD-A53077924 tianeptine Selective serotonin reuptake enhancer (SSRE)

8053 -36.68 cp BRD-A23359898 sibutramine Serotonin reuptake inhibitor

8050 -36.4 cp BRD-K08109516 L-701324 Glutamate receptor antagonist

8048 -36.22 cp BRD-A09062839 amylocaine Local anesthetic

8046 -36.05 cp BRD-K40213712 SAL-1 Adenosine receptor antagonist

8043 -35.77 cp BRD-K86204871 terconazole Sterol demethylase inhibitor

8042 -35.74 cp BRD-A80574334 oxalomalic-acid Isocitrate dehydrogenase inhibitor

8038 -35.4 cp BRD-A39172021 ampiroxicam Cyclooxygenase inhibitor

8031 -35.17 cp BRD-K01648091 LE-300 Dopamine receptor antagonist

8029 -35.11 cp BRD-K68392338 ZK-93426 Benzodiazepine receptor antagonist

8026 -34.9 cp BRD-K77625572 etomoxir Carnitine palmitoyltransferase inhibitor

8022 -34.81 cp BRD-K39391626 ethylestrenol Progesterone receptor agonist

8020 -34.69 cp BRD-K99063460 didanosine Nucleoside reverse transcriptase inhibitor

8017 -34.59 cp BRD-K76617868 fasudil Rho associated kinase inhibitor

8014 -34.55 cp BRD-A65597028 RX-821002 Adrenergic receptor antagonist

8010 -34.4 cp BRD-K96319534 phentermine Dopamine uptake inhibitor

8008 -34.23 cp BRD-K89348303 ramipril ACE inhibitor

8006 -34.21 cp BRD-K15868788 SDZ-205-557 Serotonin receptor antagonist

8001 -33.66 cp BRD-A01295252 trans-7-hydroxy-pipat Dopamine receptor ligand

8000 -33.64 cp BRD-A80928489 1-monopalmitin P-glycoprotein inhibitor

7999 -33.56 cp BRD-A50157456 terbutaline Adrenergic receptor agonist

7997 -33.48 cp BRD-K33860217 CP-94253 Serotonin receptor agonist

7991 -33.3 cp BRD-K07691486 roscovitine CDK inhibitor

7984 -32.99 cp BRD-K87932577 CDK1-5-inhibitor CDK inhibitor

7980 -32.77 cp BRD-M45964048 verteporfin Photosensitizing agent

7975 -32.46 cp BRD-K30197592 5-methoxytryptamine Serotonin receptor agonist

7970 -32.24 cp BRD-K31491153 1-phenylbiguanide Serotonin receptor agonist

7967 -32.08 cp BRD-K53523901 arctigenin MEK inhibitor

7963 -32.02 cp BRD-K46212057 voriconazole Cytochrome P450 inhibitor

7964 -32.02 cp BRD-K18316707 O-1918 Cannabinoid receptor antagonist

7959 -31.71 cp BRD-K01902415 pirinixic-acid PPAR receptor agonist

7953 -31.31 cp BRD-K55430733 WAY-629 Serotonin receptor agonist

7951 -31.14 cp BRD-A59215453 lobelanidine Acetylcholine receptor antagonist

7948 -31.06 cp BRD-K03319035 maprotiline Norepinephrine reuptake inhibitor

7943 -30.86 cp BRD-K44353683 nateglinide Insulin secretagogue

7941 -30.78 cp BRD-K05434375 HA-1004 Calcium channel blocker

7936 -30.62 cp BRD-K70881766 solanine Acetylcholinesterase inhibitor

7933 -30.39 cp BRD-M16762496 PIK-75 DNA protein kinase inhibitor

7924 -29.68 cp BRD-K08115555 tyrphostin-AG-1288 TNF production inhibitor

7925 -29.68 cp BRD-K87991767 umbelliferone Cyclooxygenase inhibitor

7918 -29.46 cp BRD-K93433262 alfacalcidol Vitamin D receptor agonist

7916 -29.17 cp BRD-K49519144 LY-2140023 Glutamate receptor agonist

7913 -29.01 cp BRD-A55393291 testosterone Androgen receptor agonist

7912 -28.98 cp BRD-K51223576 AG-99 Tyrosine kinase inhibitor

7908 -28.78 cp BRD-K50938287 sumatriptan Serotonin receptor agonist

7900 -28.33 cp BRD-A43082555 loxoprofen Cyclooxygenase inhibitor

7895 -28.16 cp BRD-K65146499 nabumetone Cyclooxygenase inhibitor

7890 -27.94 cp BRD-K07888107 depudecin HDAC inhibitor

7876 -27.23 cp BRD-K92492521 LY-255283 Leukotriene receptor antagonist

7875 -27.2 cp BRD-A45499626 UBP-302 Glutamate receptor antagonist

7874 -27.17 cp BRD-K46469693 SCH-442416 Adenosine receptor antagonist

7863 -26.82 cp BRD-A80960055 celastrol Anti-inflammatory

7862 -26.81 cp BRD-K47780086 penciclovir DNA directed DNA polymerase inhibitor

7860 -26.79 cp BRD-K92817986 BJM-CSC-19 MEK inhibitor

7859 -26.77 cp BRD-A99833829 bethanechol Acetylcholine receptor agonist

7856 -26.49 cp BRD-A83855350 naltrexone Opioid receptor antagonist

7855 -26.39 cp BRD-K77286328 reversine Aurora kinase inhibitor

7851 -26.23 cp BRD-K69032158 diprotin-a Dipeptidyl peptidase inhibitor

7847 -26.1 cp BRD-A99177642 deltaline Acetylcholine receptor antagonist

7846 -26.04 cp BRD-K07212038 selinidin Mast cell stabilizer

7843 -25.97 cp BRD-A45664787 iloprost Platelet aggregation inhibitor

7839 -25.88 cp BRD-K25906698 immepip Histamine receptor agonist

7835 -25.83 cp BRD-K33396764 alpha-linolenic-acid Omega 3 fatty acid stimulant

7827 -25.41 cp BRD-K18059238 gamma-linolenic-acid Cyclooxygenase inhibitor

7821 -25.21 cp BRD-K96670504 lonidamine Glucokinase inhibitor

7820 -25.16 cp BRD-A99411506 esculin Antioxidant

7819 -25.15 cp BRD-K08973992 linoleic-acid Oxidative stress inducer

7818 -25.04 cp BRD-K08924299 palonosetron Serotonin receptor antagonist

7815 -24.96 cp BRD-K08556791 ethoprop Acetylcholinesterase inhibitor

7812 -24.58 cp BRD-A43940795 tetrahydropalmatine Serotonin release inhibitor

7810 -24.52 cp BRD-K53913732 SB-408124 Orexin receptor antagonist

7807 -24.43 cp BRD-K92446736 zatebradine HCN channel blocker

7804 -24.23 cp BRD-K78278890 NM-PP1 Mutant kinase inhibitor

7802 -24.1 cp BRD-K18855837 varenicline Acetylcholine receptor agonist

7801 -24.07 cp BRD-K80672993 M2-PK-activator -666

7800 -24.03 cp BRD-K03384561 roquinimex Angiogenesis inhibitor

7797 -23.96 cp BRD-K40992116 parachlorophenol Anti-infective

7795 -23.94 cp BRD-K38055836 etamivan Respiratory stimulant

7792 -23.92 cp BRD-K60160658 tiagabine GABA uptake inhibitor

7790 -23.49 cp BRD-A16934955 nalbuphine Opioid receptor agonist

7786 -23.41 cp BRD-A61392169 eliprodil Glutamate receptor antagonist

7781 -23.23 cp BRD-K45117373 Y-26763 Potassium channel activator

7779 -23.2 cp BRD-K28806945 L-750667 Dopamine receptor antagonist

7777 -23.12 cp BRD-K00656370 6-aminochrysene Transferase inhibitor

7776 -23.11 cp BRD-K50018155 RS-67506 Serotonin receptor partial agonist

7765 -22.63 cp BRD-K96271548 coumaric-acid Antioxidant

7764 -22.62 cp BRD-A02176148 tubaic-acid Mitochondrial complex I inhibitor

7762 -22.52 cp BRD-A25067867 benzatropine Acetylcholine receptor antagonist

7754 -22.34 cp BRD-K73838513 cinacalcet Calcium channel activator

7752 -22.29 cp BRD-A77118605 BML-ST330 Phospholipase inhibitor

7751 -22.27 cp BRD-K70883034 nimetazepam GABA receptor agonist

7750 -22.25 cp BRD-A47598013 citalopram Serotonin reuptake inhibitor

7749 -22.18 cp BRD-K82147103 lofepramine Norepinephrine reuptake inhibitor

7743 -21.95 cp BRD-A24543851 nornicotine Acetylcholine receptor agonist

7737 -21.81 cp BRD-A34751532 homosalate HSP inducer

7731 -21.38 cp BRD-A37837077 cyclazosin Adrenergic receptor antagonist

7729 -21.3 cp BRD-K18250272 propoxycaine Local anesthetic

7718 -20.84 cp BRD-K94294671 OSI-027 MTOR inhibitor

7714 -20.74 cp BRD-A62182663 YK-4279 Apoptosis stimulant

7715 -20.74 cp BRD-K61323504 SB-225002 CC chemokine receptor antagonist

7713 -20.72 cp BRD-K65639003 icariin Phosphodiesterase inhibitor

7708 -20.53 cp BRD-K19309090 SR-95639A Acetylcholine receptor agonist

7706 -20.42 cp BRD-K00675675 CL-82198 Metalloproteinase inhibitor

7705 -20.37 cp BRD-K12762134 XAV-939 Tankyrase inhibitor

7701 -20.27 cp BRD-K13664374 dichloroacetic-acid Pyruvate dehydrogenase kinase inhibitor

7702 -20.27 cp BRD-A08877921 cephalotaxine Protein synthesis inhibitor

7695 -20.15 cp BRD-K02867583 minaprine Serotonin reuptake inhibitor

7693 -20.14 cp BRD-K69650333 idarubicin Topoisomerase inhibitor

7694 -20.14 cp BRD-K25186396 tangeritin Cell cycle inhibitor

7692 -20.1 cp BRD-A65076780 dihydroergocristine Adrenergic receptor antagonist

7691 -20.07 cp BRD-K54316499 tolterodine Acetylcholine receptor antagonist

7689 -20.06 cp BRD-K66412701 pazufloxacin Topoisomerase inhibitor

7686 -19.88 cp BRD-K48722833 iloperidone Dopamine receptor antagonist

7683 -19.79 cp BRD-K37130656 rivaroxaban Coagulation inhibitor

7682 -19.69 cp BRD-A87387433 cefpodoxime Bacterial cell wall synthesis inhibitor

7681 -19.68 cp BRD-K45861246 azaperone Dopamine receptor antagonist

7679 -19.6 cp BRD-K59273480 propentofylline Adenosine reuptake inhibitor

7676 -19.5 cp BRD-K50422030 clomethiazole GABA receptor antagonist

7674 -19.46 cp BRD-K49890030 gavestinel Glutamate receptor antagonist

7673 -19.45 cp BRD-K60174629 z-prolyl-prolinal Prolyl endopeptidase inhibitor

7670 -19.2 cp BRD-A96799240 4-hydroxyretinoic-acid Retinoid receptor binder

7668 -19.16 cp BRD-K66615216 moxifloxacin Bacterial DNA gyrase inhibitor

7666 -19.1 cp BRD-K85925969 zalcitabine Nucleoside reverse transcriptase inhibitor

7664 -18.97 cp BRD-K32830106 guanfacine Adrenergic receptor agonist

7661 -18.88 cp BRD-K27184429 levocetirizine Histamine receptor antagonist

7659 -18.85 cp BRD-A35912562 pregnenolone Glutamate receptor modulator

7657 -18.84 cp BRD-K59419204 AM-281 Cannabinoid receptor antagonist

7656 -18.79 cp BRD-A64297288 amlodipine Calcium channel blocker

7649 -18.52 cp BRD-K76805682 SB-415286 Glycogen synthase kinase inhibitor

7645 -18.48 cp BRD-K43468059 byssochlamic-acid Mycotoxin

7646 -18.48 cp BRD-K09991945 GSK-3-inhibitor-II PKC inhibitor

7633 -18.08 cp BRD-K67277431 picotamide Thromboxane receptor antagonist

7632 -18.04 cp BRD-K60184833 tyrphostin-46 Tyrosine kinase inhibitor

7628 -17.88 cp BRD-K11158509 tyrphostin-B44 EGFR inhibitor

7625 -17.84 cp BRD-K47539947 tetradecylthioacetic-acid Lipid peroxidase inhibitor

7626 -17.84 cp BRD-K25905511 buddleoflavonoloside Acetylcholinesterase inhibitor

7619 -17.68 cp BRD-K28863208 PNU-282987 Cholinergic receptor agonist

7616 -17.56 cp BRD-K06388322 pramipexole Dopamine receptor agonist

7615 -17.53 cp BRD-K92015269 GBR-12783 Dopamine uptake inhibitor

7613 -17.49 cp BRD-K96354014 nifedipine Calcium channel blocker

7610 -17.46 cp BRD-K14888893 minoxidil KATP activator

7606 -17.33 cp BRD-K49865102 PD-0325901 MEK inhibitor

7604 -17.29 cp BRD-U33728988 QL-X-138 MTOR inhibitor

7603 -17.25 cp BRD-K91442916 CAM-9-026 Membrane metalloendopeptidase inhibitor

7596 -17.05 cp BRD-K32412559 morphothebaine Adrenergic receptor antagonist

7595 -16.99 cp BRD-K12102668 nialamide Monoamine oxidase inhibitor

7594 -16.95 cp BRD-A94543220 bifonazole Sterol demethylase inhibitor

7587 -16.71 cp BRD-K31542390 mycophenolic-acid Dehydrogenase inhibitor

7584 -16.61 cp BRD-K35687265 ON-01910 PLK inhibitor

7583 -16.6 cp BRD-A97739905 ketoprofen Cyclooxygenase inhibitor

7581 -16.51 cp BRD-K82164249 andarine Androgen receptor modulator

7580 -16.49 cp BRD-K24132293 piperlongumine Glutathione transferase inhibitor

7579 -16.46 cp BRD-K08640512 RS-100329 Adrenergic receptor antagonist

7578 -16.43 cp BRD-A64933752 CV-1808 Adenosine receptor agonist

7575 -16.3 cp BRD-A24228527 ofloxacin Bacterial DNA gyrase inhibitor

7574 -16.29 cp BRD-K44084986 Y-27632 Rho associated kinase inhibitor

7571 -16.21 cp BRD-K22031190 diflunisal Prostanoid receptor antagonist

7566 -16.15 cp BRD-K10042277 desmethylclozapine Acetylcholine receptor agonist

7561 -16.02 cp BRD-K95655893 MAZ-51 VEGFR inhibitor

7562 -16.02 cp BRD-A90799790 isradipine Calcium channel blocker

7558 -15.99 cp BRD-K91900765 VX-745 p38 MAPK inhibitor

7556 -15.88 cp BRD-A38747044 KU-14R Imidazoline receptor ligand

7553 -15.79 cp BRD-K36864847 BD-1047 Adrenergic receptor antagonist

7547 -15.61 cp BRD-K66766661 17-beta-estradiol Estrogen receptor agonist

7545 -15.58 cp BRD-K57546357 prunetin Breast cancer resistance protein inhibitor

7541 -15.52 cp BRD-A65282128 cefazolin Bacterial cell wall synthesis inhibitor

7540 -15.5 cp BRD-K89687904 PKCbeta-inhibitor PKC inhibitor

7539 -15.46 cp BRD-A49225603 alimemazine Histamine receptor agonist

7538 -15.44 cp BRD-K60923938 veratridine Sodium channel activator

7536 -15.41 cp BRD-K51671335 sulpiride Dopamine receptor antagonist

7535 -15.39 cp BRD-K48869804 icilin TRPV agonist

7534 -15.35 cp BRD-K46742498 alosetron Serotonin receptor antagonist

7532 -15.28 cp BRD-K30990140 FR-122047 Cyclooxygenase inhibitor

7530 -15.11 cp BRD-K09635314 M-3M3FBS phospholipase activator

7529 -15.06 cp BRD-K05737787 isoeugenol Nitric oxide production inhibitor

7528 -15.02 cp BRD-A99449986 MT-21 Caspase activator

7524 -14.95 cp BRD-K50384076 "7,4'-dihydroxyflavone" Opioid receptor antagonist

7525 -14.95 cp BRD-K54790157 trioxsalen DNA synthesis inhibitor

7521 -14.9 cp BRD-K77171813 proxyfan Histamine receptor modulator

7518 -14.87 cp BRD-K49668410 clarithromycin Bacterial 50S ribosomal subunit inhibitor

7516 -14.83 cp BRD-A75552914 isoxicam Cyclooxygenase inhibitor

7513 -14.75 cp BRD-K17378184 prestwick-559 Dopamine receptor agonist

7512 -14.74 cp BRD-A18620900 estriol Estrogen receptor agonist

7511 -14.73 cp BRD-K80325895 eicosadienoic-acid -666

7507 -14.56 cp BRD-A85280935 quinpirole Dopamine receptor agonist

7504 -14.48 cp BRD-A41941932 vitexin Antioxidant

7505 -14.48 cp BRD-K32828673 chelidonine Tubulin inhibitor

7499 -14.42 cp BRD-A18917088 estradiol Contraceptive agent

7498 -14.36 cp BRD-K93880783 stavudine DNA directed DNA polymerase inhibitor

7495 -14.35 cp BRD-A51829654 BRL-15572 Serotonin receptor antagonist

7491 -14.34 cp BRD-A61470182 n-formylmethionylalanine macrophage activator

7492 -14.34 cp BRD-K70301876 escitalopram Selective serotonin reuptake inhibitor (SSRI)

7490 -14.33 cp BRD-K00317371 RITA MDM inhibitor

7488 -14.25 cp BRD-A31312900 montelukast Leukotriene receptor antagonist

7485 -14.22 cp BRD-K15601958 SEW-2871 Lysophospholipid receptor agonist

7484 -14.19 cp BRD-A74500471 ethambutol Bacterial cell wall synthesis inhibitor

7482 -14.14 cp BRD-A36066264 estradiol-benzoate Estrogen receptor agonist

7476 -13.99 cp BRD-K66019333 oxantel Anthelmintic

7472 -13.91 cp BRD-A91452556 estradiol-cypionate Estrogen receptor agonist

7467 -13.85 cp BRD-A94793051 gestrinone Progesterone receptor antagonist

7465 -13.83 cp BRD-A31800922 procyclidine Acetylcholine receptor antagonist

7464 -13.78 cp BRD-K46766488 S-14506 Serotonin receptor agonist

7463 -13.77 cp BRD-K77008974 WYE-354 MTOR inhibitor

7460 -13.75 cp BRD-K68095457 palmitoylethanolamide Cannabinoid receptor agonist

7450 -13.54 cp BRD-K27141178 SB-203186 Serotonin receptor antagonist

7448 -13.52 cp BRD-K63550407 erythromycin NFkB pathway inhibitor

7444 -13.39 cp BRD-K87048468 RS-102221 Serotonin receptor antagonist

7442 -13.32 cp BRD-K52911425 GDC-0941 PI3K inhibitor

7435 -13.27 cp BRD-A00267231 hemado Adenosine receptor agonist

7437 -13.27 cp BRD-K07881437 danusertib Aurora kinase inhibitor

7434 -13.26 cp BRD-A24122750 saclofen GABA receptor antagonist

7431 -13.21 cp BRD-A32349859 methyl-angolensate Apoptosis inhibitor

7427 -13.19 cp BRD-A44780397 mifepristone Glucocorticoid receptor antagonist

7428 -13.19 cp BRD-K81209512 AG-494 EGFR inhibitor

7429 -13.19 cp BRD-A29901043 KIN001-127 ITK inhibitor

7426 -13.18 cp BRD-A77349281 RK-682 Tyrosine phosphatase inhibitor

7424 -13.13 cp BRD-K92991072 PAC-1 Caspase activator

7421 -13.08 cp BRD-K24538644 KUC104502N -666

7420 -13.07 cp BRD-K08703257 3-amino-benzamide PARP inhibitor

7419 -13.06 cp BRD-K81855038 roxatidine Histamine receptor antagonist

7417 -12.94 cp BRD-K74195153 irsogladine Phosphodiesterase inhibitor

7413 -12.89 cp BRD-K00206590 P-1075 ATP channel activator

7409 -12.78 cp BRD-K94841585 emodic-acid Laxative

7407 -12.74 cp BRD-A87479750 tenidap Cyclooxygenase inhibitor

7405 -12.72 cp BRD-K83794624 pirarubicin Topoisomerase inhibitor

7404 -12.68 cp BRD-K47598052 PP-1 SRC inhibitor

7398 -12.47 cp BRD-K08619574 thioproperazine Dopamine receptor antagonist

7395 -12.45 cp BRD-K97061094 azacyclonol Histamine receptor antagonist

7388 -12.36 cp BRD-A88774919 doxycycline Bacterial 30S ribosomal subunit inhibitor

7384 -12.24 cp BRD-A33447119 oxfendazole Anthelmintic

7383 -12.22 cp BRD-K98157055 SIB-1757 Glutamate receptor antagonist

7382 -12.21 cp BRD-K94832621 Y-134 Estrogen receptor antagonist

7379 -12.15 cp BRD-A68631409 evodiamine ATPase inhibitor

7374 -12.09 cp BRD-K67352070 TC-2559 Acetylcholine receptor agonist

7372 -12.02 cp BRD-K58772419 AZD-6482 PI3K inhibitor

7368 -11.88 cp BRD-K17896185 FIT Opioid receptor agonist

7365 -11.8 cp BRD-K37846922 "3,3'-diindolylmethane" CHK inhibitor

7361 -11.77 cp BRD-K85030058 benactyzine Acetylcholine receptor antagonist

7363 -11.77 cp BRD-K97752965 nicorandil Nitric oxide donor

7358 -11.63 cp BRD-K88568253 iproniazid Monoamine oxidase inhibitor

7353 -11.53 cp BRD-A00100033 nifurtimox DNA inhibitor

7351 -11.47 cp BRD-K64785675 TG100-115 -666

7344 -11.27 cp BRD-K18799075 BAY-59-3074 Cannabinoid receptor partial agonist

7342 -11.23 cp BRD-A18043272 phensuximide Succinimide antiepileptic

7341 -11.22 cp BRD-K77998258 ganglioside SRC activator

7336 -11.11 cp BRD-A96485169 EBPC Aldose reductase inhibitor

7335 -11.1 cp BRD-K95763993 trapidil PDGFR receptor inhibitor

7332 -11 cp BRD-K94379058 BML-190 Cannabinoid receptor inverse agonist

7330 -10.97 cp BRD-K89274813 DMP-543 Acetylcholine release stimulant

7331 -10.97 cp BRD-A36217750 sulfinpyrazone Uricosuric blocker

7328 -10.92 cp BRD-K02404261 caffeine Adenosine receptor antagonist

7327 -10.89 cp BRD-A26032986 zaldaride Calmodulin antagonist

7323 -10.79 cp BRD-A79981887 midodrine Adrenergic receptor agonist

7321 -10.75 cp BRD-K73319509 PF-04217903 c-Met inhibitor

7320 -10.74 cp BRD-K40175214 torin-1 MTOR inhibitor

7317 -10.66 cp BRD-A14208071 oxyphenonium Cholinergic receptor antagonist

7316 -10.65 cp BRD-K37561857 zardaverine Phosphodiesterase inhibitor

7313 -10.57 cp BRD-K51677086 erythromycin NFkB pathway inhibitor

7312 -10.56 cp BRD-K15933101 ropinirole Dopamine receptor agonist

7311 -10.55 cp BRD-K22878149 SB-205607 Delta 1 opioid receptor agonist

7310 -10.54 cp BRD-K61192372 capecitabine DNA synthesis inhibitor

7307 -10.5 cp BRD-K09638361 SA-63133 -666

7302 -10.42 cp BRD-K88871508 lisuride Dopamine receptor agonist

7300 -10.34 cp BRD-K12184916 dactolisib MTOR inhibitor

7294 -10.2 cp BRD-A06352508 SB-218078 CHK inhibitor

7288 -10.1 cp BRD-K46142322 RS-67333 Serotonin receptor partial agonist

7290 -10.1 cp BRD-U88459701 atorvastatin HMGCR inhibitor

7282 -10.02 cp BRD-K42452249 EO-1428 p38 MAPK inhibitor

7279 -9.97 cp BRD-K31633810 DAU-5884 Acetylcholine receptor antagonist

7278 -9.94 cp BRD-K36529613 PU-H71 HSP inhibitor

7277 -9.93 cp BRD-K84639753 safinamide Dopamine uptake inhibitor

7270 -9.75 cp BRD-A69636825 diltiazem Calcium channel blocker

7266 -9.6 cp BRD-A26711594 nicardipine Calcium channel blocker

7263 -9.55 cp BRD-K19462402 buflomedil Adrenergic receptor antagonist

7261 -9.52 cp BRD-A33084410 5'-guanidinonaltrindole Opioid receptor antagonist

7259 -9.45 cp BRD-K71823332 epothilone-a Microtubule stabilizing agent

7257 -9.43 cp BRD-K70241288 L-692585 Growth hormone releasing peptide ligand agonist

7256 -9.41 cp BRD-K57886322 fluocinonide Glucocorticoid receptor agonist

7255 -9.39 cp BRD-K89162000 tandutinib FLT3 inhibitor

7251 -9.35 cp BRD-A12560204 nitrendipine Calcium channel blocker

7246 -9.23 cp BRD-A14574269 UB-165 Acetylcholine receptor agonist

7245 -9.21 cp BRD-K49372556 mofezolac Cyclooxygenase inhibitor

7239 -9.14 cp BRD-K15519488 CS-110266 Dopamine receptor agonist

7240 -9.14 cp BRD-K59633790 VU-0420363-1 SARS coronavirus 3C-like protease inhibitor

7230 -8.91 cp BRD-K01192156 tyrphostin-AG-112 Protein tyrosine kinase inhibitor

7227 -8.87 cp BRD-K50891186 GR-103691 Dopamine receptor antagonist

7226 -8.86 cp BRD-A54596827 solifenacin Acetylcholine receptor antagonist

7223 -8.83 cp BRD-A39255369 DCPIB Chloride channel blocker

7216 -8.75 cp BRD-K43796186 benzyl-quinazolin-4-yl-amine EGFR inhibitor

7211 -8.67 cp BRD-K63068307 ZSTK-474 PI3K inhibitor

7206 -8.6 cp BRD-K57304726 PRE-084 Sigma receptor agonist

7201 -8.55 cp BRD-K18779551 bifemelane Acetylcholine release stimulant

7199 -8.53 cp BRD-A32161980 carbetocin Oxytocin receptor agonist

7198 -8.51 cp BRD-K39621635 artemether Antimalarial

7194 -8.45 cp BRD-K13390322 AT-7519 CDK inhibitor

7193 -8.44 cp BRD-K06147391 telenzepine Acetylcholine receptor antagonist

7187 -8.34 cp BRD-A94669766 naringenin Aromatase inhibitor

7186 -8.32 cp BRD-K10705233 GW-405833 Cannabinoid receptor agonist

7185 -8.3 cp BRD-K29555132 arachidonamide Cannabinoid receptor agonist

7182 -8.28 cp BRD-A14985772 ascorbyl-palmitate antioxidant

7177 -8.17 cp BRD-K76775527 nimesulide Cyclooxygenase inhibitor

7175 -8.15 cp BRD-K86958018 olvanil TRPV agonist

7176 -8.15 cp BRD-K34608650 BRD-K34608650 Cannabinoid receptor agonist

7174 -8.14 cp BRD-K73196317 urapidil Adrenergic receptor antagonist

7172 -8.13 cp BRD-K05181084 NGB-2904 Dopamine receptor antagonist

7169 -8 cp BRD-K14767410 SC-560 Cyclooxygenase inhibitor

7168 -7.99 cp BRD-K61401890 deguelin NADH-ubiquinone oxidoreductase (Complex I) inhibitor

7166 -7.98 cp BRD-K27351809 nomegestrol Progesterone receptor agonist

7165 -7.97 cp BRD-K67043667 altretamine DNA synthesis inhibitor

7162 -7.9 cp BRD-K85871428 SC-68376 p38 MAPK inhibitor

7160 -7.88 cp BRD-K65786282 CGP-7930 GABA receptor modulator

7161 -7.88 cp BRD-A02367930 ethinyl-estradiol DNA directed DNA polymerase stimulant

7158 -7.87 cp BRD-K69690935 curcumin Cyclooxygenase inhibitor

7152 -7.78 cp BRD-K70281171 U-99194 Dopamine receptor antagonist

7151 -7.77 cp BRD-A55756846 H-7 PKA inhibitor

7148 -7.72 cp BRD-K68174511 torin-2 MTOR inhibitor

7149 -7.72 cp BRD-K54095730 CMPD-1 p38 MAPK inhibitor

7144 -7.68 cp BRD-A15435692 BMY-14802 Sigma receptor antagonist

7143 -7.66 cp BRD-A97479839 piperidolate Acetylcholine receptor antagonist

7141 -7.64 cp BRD-K47631482 bromhexine Mucolytic agent

7140 -7.61 cp BRD-K79404599 enzastaurin PKC inhibitor

7137 -7.53 cp BRD-K00662280 CL-218872 GABA receptor agonist

7133 -7.48 cp BRD-K45068323 W-13 Calmodulin antagonist

7132 -7.47 cp BRD-K59773493 benzohydroxamic-acid Antifungal

7130 -7.44 cp BRD-K06895174 cisapride Serotonin receptor agonist

7126 -7.38 cp BRD-K09186807 KIN001-244 Phosphoinositide dependent kinase inhibitor

7123 -7.3 cp BRD-A22713669 BVT-948 Tyrosine phosphatase inhibitor

7120 -7.29 cp BRD-K05350981 oligomycin-c ATPase inhibitor

7121 -7.29 cp BRD-K20141153 atomoxetine Norepinephrine transporter inhibitor

7118 -7.28 cp BRD-A15493168 tetracycline Bacterial 30S ribosomal subunit inhibitor

7115 -7.23 cp BRD-K28667793 pyrazinamide Fatty acid synthase inhibitor

7116 -7.23 cp BRD-A55369275 CGP-54626 GABA receptor antagonist

7108 -7.19 cp BRD-U68942961 JW-7-24-1 LCK Inhibitor

7103 -7.15 cp BRD-K15842202 eudesmic-acid -666

7102 -7.14 cp BRD-K44899736 RO-16-6941 Monoamine oxidase inhibitor

7100 -7.12 cp BRD-K52394958 GR-159897 Tachykinin antagonist

7101 -7.12 cp BRD-K15164005 apoptosis-activator-II Carboxylesterase inhibitor

7097 -7.1 cp BRD-K51313569 palbociclib CDK inhibitor

7096 -7.08 cp BRD-K81783531 VX-222 HCV inhibitor

7093 -7.07 cp BRD-K41996876 tyrphostin-1 EGFR inhibitor

7092 -7.06 cp BRD-K72264770 QW-BI-011 Histone lysine methyltransferase inhibitor

7089 -7.05 cp BRD-K90574421 ipsapirone Serotonin receptor agonist

7085 -7 cp BRD-A28856712 tetryzoline Adrenergic receptor agonist

7082 -6.98 cp BRD-K50495309 SRC-kinase-inhibitor-I SRC inhibitor

7083 -6.98 cp BRD-K04548931 pidorubicine Topoisomerase inhibitor

7084 -6.98 cp BRD-K63641886 cefuroxime Bacterial cell wall synthesis inhibitor

7080 -6.94 cp BRD-A10969569 ambelline Plant alkaloid

7079 -6.91 cp BRD-A07000685 hydrocortisone Glucocorticoid receptor agonist

7076 -6.88 cp BRD-A63667919 methylergometrine Dopamine receptor antagonist

7075 -6.87 cp BRD-A36471396 biperiden Acetylcholine receptor antagonist

7070 -6.81 cp BRD-A78391468 prednisolone Glucocorticoid receptor agonist

7072 -6.81 cp BRD-K09778810 FGIN-1-27 Inositol monophosphatase inhibitor

7068 -6.8 cp BRD-K26015241 ODQ Guanylyl cyclase inhibitor

7064 -6.75 cp BRD-A64228451 terreic-acid BTK inhibitor

7059 -6.7 cp BRD-K82577285 dipropyl-dopamine Dopamine receptor agonist

7058 -6.69 cp BRD-A47494775 dipivefrine Adrenergic receptor agonist

7056 -6.66 cp BRD-K32247306 primidone GABA receptor antagonist

7050 -6.62 cp BRD-A07395371 esmolol Adrenergic receptor antagonist

7051 -6.62 cp BRD-A66927094 nemonapride Dopamine receptor antagonist

7052 -6.62 cp BRD-A54487287 cortisone Glucocorticoid receptor agonist

7048 -6.61 cp BRD-K81376179 TCS-359 FLT3 inhibitor

7047 -6.59 cp BRD-K68402494 ML-9 Myosin light chain kinase inhibitor

7045 -6.55 cp BRD-K50398167 meclofenamic-acid Cyclooxygenase inhibitor

7042 -6.54 cp BRD-K55454768 TAS-301 Calcium-calmodulin dependent protein kinase inhibitor

7036 -6.48 cp BRD-K17674993 diflorasone Corticosteroid agonist

7037 -6.48 cp BRD-K98426715 tubacin HDAC inhibitor

7032 -6.45 cp BRD-A16754160 ampicillin Bacterial cell wall synthesis inhibitor

7031 -6.44 cp BRD-K00312224 PPT Estrogen receptor agonist

7029 -6.42 cp BRD-K53979406 ALX-5407 Glycine transporter inhibitor

7028 -6.38 cp BRD-K93658967 aloisine CDK inhibitor

7027 -6.37 cp BRD-K02992638 lamivudine Nucleoside reverse transcriptase inhibitor

7024 -6.34 cp BRD-K08219523 5-nonyloxytryptamine Serotonin receptor agonist

7022 -6.31 cp BRD-A05352148 ipratropium Acetylcholine receptor antagonist

7023 -6.31 cp BRD-K17823458 danoprevir HCV inhibitor

7014 -6.21 cp BRD-A11135865 nor-binaltorphimine Opioid receptor antagonist

7015 -6.21 cp BRD-K67080878 milrinone Phosphodiesterase inhibitor

7012 -6.18 cp BRD-K25741894 skimmianine Acetylcholinesterase inhibitor

7011 -6.17 cp BRD-K45479396 BP-554 Serotonin receptor agonist

7001 -6.1 cp BRD-K45446451 JZL-184 Monoacylglucerol lipase inhibitor

6994 -6.02 cp BRD-A71157293 fursultiamine Vitamin B

6986 -5.92 cp BRD-K92428153 mycophenolate-mofetil Dehydrogenase inhibitor

6985 -5.91 cp BRD-K07736136 VX-702 p38 MAPK inhibitor

6983 -5.88 cp BRD-A84174393 meloxicam Cyclooxygenase inhibitor

6981 -5.87 cp BRD-K13800121 parecoxib Cyclooxygenase inhibitor

6976 -5.79 cp BRD-A07440155 labetalol Adrenergic receptor antagonist

6972 -5.76 cp BRD-K75641298 metoclopramide Dopamine receptor antagonist

6971 -5.72 cp BRD-K55424922 anpirtoline Serotonin receptor agonist

6967 -5.65 cp BRD-A44551378 LFM-A12 EGFR inhibitor

6963 -5.64 cp BRD-A61793559 metolazone Carbonic anhydrase inhibitor

6964 -5.64 cp BRD-K52219182 BRD-K52219182 Phosphodiesterase inhibitor

6962 -5.63 cp BRD-K09416995 lovastatin HMGCR inhibitor

6960 -5.61 cp BRD-K71289571 zafirlukast Leukotriene receptor antagonist

6949 -5.5 cp BRD-A62071884 siguazodan Phosphodiesterase inhibitor

6944 -5.45 cp BRD-K15891719 tenofovir Reverse transcriptase inhibitor

6942 -5.44 cp BRD-K76133116 benzydamine Membrane integrity inhibitor

6943 -5.44 cp BRD-K43290182 Ro-04-6790 Serotonin receptor antagonist

6938 -5.38 cp BRD-K27499107 carbacyclin IP receptor activator

6937 -5.37 cp BRD-K91904471 SD-169 p38 MAPK inhibitor

6935 -5.36 cp BRD-A84389091 L-655708 GABA receptor inverse agonist

6930 -5.33 cp BRD-A43974575 tranylcypromine Monoamine oxidase inhibitor

6927 -5.32 cp BRD-A39522003 OMDM-2 FAAH inhibitor

6928 -5.32 cp BRD-A28422058 L-689560 Glutamate receptor antagonist

6921 -5.28 cp BRD-U44618005 WH-4023 SRC inhibitor

6922 -5.28 cp BRD-K05977355 fluconazole Sterol demethylase inhibitor

6923 -5.28 cp BRD-K95237249 probenecid Uricosuric blocker

6918 -5.25 cp BRD-K79877282 PF-543 Sphingosine kinase inhibitor

6917 -5.22 cp BRD-U66370498 androstanol CAR antagonist

6913 -5.17 cp BRD-K78373679 RO-3306 CDK inhibitor

6911 -5.16 cp BRD-K32645441 dipropyl-5ct Serotonin receptor agonist

6908 -5.14 cp BRD-K23204545 busulfan DNA inhibitor

6894 -5.04 cp BRD-K74501079 azithromycin Bacterial 50S ribosomal subunit inhibitor

6890 -4.95 cp BRD-A25576662 streptozotocin DNA alkylating agent

6886 -4.93 cp BRD-U97083655 teicoplanin Bacterial cell wall synthesis inhibitor

6887 -4.93 cp BRD-K15834839 lobendazole Anthelmintic

6885 -4.91 cp BRD-K59522102 piperine Monoamine oxidase inhibitor

6883 -4.9 cp BRD-K79437791 acetyl-farnesyl-cysteine Inhibitor of methylation of endogenous isoprenylated proteins

6881 -4.89 cp BRD-K76534306 enrofloxacin Bacterial DNA gyrase inhibitor

6880 -4.88 cp BRD-K49027941 PSB-1115 Adenosine receptor antagonist

6879 -4.86 cp BRD-K28453807 nitrocaramiphen Cholinergic receptor antagonist

6877 -4.85 cp BRD-K08890269 CO-102862 Sodium channel blocker

6869 -4.82 cp BRD-A71657825 2-(biphenyl-4-ylsulfonamido)pentanedioic-acid Matrix metalloprotease inhibitor

6870 -4.82 cp BRD-A66199457 asiaticoside Antineoplastic

6866 -4.79 cp BRD-K31283835 tofacitinib JAK inhibitor

6862 -4.74 cp BRD-A39390670 rabeprazole ATPase inhibitor

6858 -4.69 cp BRD-K30649484 mafenide Carbonic anhydrase inhibitor

6854 -4.68 cp BRD-A66563878 medetomidine Adrenergic receptor agonist

6851 -4.64 cp BRD-K59184148 SB-216763 Glycogen synthase kinase inhibitor

6843 -4.58 cp BRD-K34820100 tebuthiuron Photosynthesis inhibitor

6844 -4.58 cp BRD-K17294426 clebopride Dopamine receptor antagonist

6842 -4.56 cp BRD-K51066026 aminoindazole Ionophore

6841 -4.55 cp BRD-K55991774 BAS-09104376 HIV integrase inhibitor

6837 -4.5 cp BRD-K87024524 phenelzine Monoamine oxidase inhibitor

6838 -4.5 cp BRD-K38251852 paxilline Potassium channel blocker

6830 -4.46 cp BRD-A35989968 megestrol Progesterone receptor agonist

6833 -4.46 cp BRD-K48427617 U-0124 MEK inhibitor

6828 -4.45 cp BRD-K23913458 coumarin Vitamin K antagonist

6826 -4.44 cp BRD-A52588987 SKF-83566 Dopamine receptor antagonist

6812 -4.33 cp BRD-K93176058 AC-55649 Retinoid receptor agonist

6810 -4.31 cp BRD-A92630576 trimebutine Opioid receptor agonist

6807 -4.3 cp BRD-K02275692 cefotiam Bacterial cell wall synthesis inhibitor

6802 -4.25 cp BRD-K92870997 pterostilbene Cyclooxygenase inhibitor

6798 -4.23 cp BRD-K64606589 apicidin HDAC inhibitor

6795 -4.21 cp BRD-K90333595 phentolamine Adrenergic receptor antagonist

6791 -4.18 cp BRD-K83302049 protopine Histamine receptor antagonist

6784 -4.14 cp BRD-A22143024 estropipate Estrogen receptor agonist

6785 -4.14 cp BRD-K93080877 Ala-Ala-Phe-CMK Tripeptidyl peptidase inhibitor

6783 -4.12 cp BRD-A68039575 liquiritigenin Aromatase inhibitor

6780 -4.1 cp BRD-K50660797 epicatechin Bacterial DNA gyrase inhibitor

6774 -4.05 cp BRD-A67748489 K3644 Kinesin-like spindle protein inhibitor

6777 -4.05 cp BRD-K46424862 hymecromone Monoamine oxidase inhibitor

6773 -4.03 cp BRD-K32584078 BML-257 AKT inhibitor

6771 -4.02 cp BRD-K33864865 LY-225910 CCK receptor antagonist

6772 -4.02 cp BRD-A11813248 AM-92016 Potassium channel blocker

6770 -4.01 cp BRD-K89708791 rifaximin RNA synthesis inhibitor

6769 -3.99 cp BRD-K70487031 flupentixol Dopamine receptor antagonist

6767 -3.98 cp BRD-A51182606 chloramphenicol Protein synthesis inhibitor

6760 -3.94 cp BRD-A06726973 dibutyrylcyclic-gmp cGMP analog

6761 -3.94 cp BRD-K17110974 aristolochic-acid Phospholipase inhibitor

6756 -3.91 cp BRD-K59456551 methotrexate Dihydrofolate reductase inhibitor

6757 -3.91 cp BRD-A13946108 sulindac Cyclooxygenase inhibitor

6751 -3.88 cp BRD-K86434416 selegiline Monoamine oxidase inhibitor

6749 -3.86 cp BRD-A09925278 etilefrine Adrenergic receptor agonist

6723 -3.74 cp BRD-K96720755 relcovaptan Vasopressin receptor antagonist

6726 -3.74 cp BRD-A92585442 RU-28318 Cytochrome P450 inhibitor

6722 -3.73 cp BRD-K71003802 hippeastrine Plant alkaloid

6720 -3.72 cp BRD-K52930707 rescinnamine ACE inhibitor

6707 -3.66 cp BRD-K20287671 SU-4312 PDGFR receptor inhibitor

6712 -3.66 cp BRD-K67637637 olopatadine Histamine receptor antagonist

6713 -3.66 cp BRD-K39823328 VU-0366037-2 Glutamate receptor modulator

6701 -3.63 cp BRD-K10860596 granisetron Serotonin receptor antagonist

6702 -3.63 cp BRD-K61993165 niacin NAD precursor with lipid lowering effects

6703 -3.63 cp BRD-A14395271 mesoridazine Dopamine receptor antagonist

6705 -3.63 cp BRD-A24514565 warfarin Vitamin K antagonist

6690 -3.59 cp BRD-K13927029 retinol Retinoid receptor ligand

6691 -3.59 cp BRD-K97509413 coumestrol Estrogen receptor agonist

6692 -3.59 cp BRD-K51318897 fenbendazole Tubulin inhibitor

6693 -3.59 cp BRD-A32836748 leu-enkephalin Opioid receptor agonist

6694 -3.59 cp BRD-K86600316 RS-79948 Adrenergic receptor antagonist

6686 -3.56 cp BRD-K92726801 hydrastinine Haemostatic agent

6682 -3.55 cp BRD-K95402279 geranylgeraniol Farnesyltransferase inhibitor

6678 -3.52 cp BRD-K97564742 mepyramine Histamine receptor antagonist

6675 -3.47 cp BRD-K34533029 tyrphostin-AG-494 EGFR inhibitor

6666 -3.42 cp BRD-A26002865 verrucarin-a Protein synthesis inhibitor

6667 -3.42 cp BRD-K23623876 decafluorobutane Contrast agent

6665 -3.38 cp BRD-K87142802 veliparib PARP inhibitor

6661 -3.36 cp BRD-K14200658 syrosingopine Vesicular monoamine transporter inhibitor

6657 -3.34 cp BRD-K49328571 dasatinib BCR-ABL kinase inhibitor

6658 -3.34 cp BRD-K20995441 U-54494A Opioid receptor agonist

6656 -3.33 cp BRD-A20239487 atenolol Adrenergic receptor antagonist

6652 -3.31 cp BRD-A15010982 HU-211 Glutamate receptor antagonist

6653 -3.31 cp BRD-K59597909 phenothiazine Dopamine receptor antagonist

6646 -3.28 cp BRD-K82688027 RG-13022 PDGFR receptor inhibitor

6648 -3.28 cp BRD-K90885812 propantheline Acetylcholine receptor antagonist

6645 -3.27 cp BRD-K07996107 harpagoside Acetylcholinesterase inhibitor

6633 -3.24 cp BRD-A97730597 hexylcaine Sodium channel blocker

6634 -3.24 cp BRD-K66296774 fluvastatin HMGCR inhibitor

6625 -3.19 cp BRD-K11107424 tiotidine Histamine receptor antagonist

6626 -3.19 cp BRD-K23566484 nilutamide Androgen receptor antagonist

6620 -3.17 cp BRD-K18618618 cimetidine Histamine receptor antagonist

6621 -3.17 cp BRD-K68191783 ALW-II-38-3 Ephrin inhibitor

6614 -3.14 cp BRD-K37720887 SB-525334 TGF beta receptor inhibitor

6615 -3.14 cp BRD-K56403959 ZK-756326 CC chemokine receptor ligand

6605 -3.08 cp BRD-K92758126 gibberellic-acid NFkB pathway inhibitor

6602 -3.07 cp BRD-K64310881 MW-STK33-3B Potassium channel activator

6599 -3.06 cp BRD-K67100011 pivmecillinam Bacterial cell wall synthesis inhibitor

6592 -3.05 cp BRD-K43068349 AMG-9810 TRPV antagonist

6590 -3.04 cp BRD-A17428743 BW-723C86 Serotonin receptor agonist

6586 -3.03 cp BRD-K90733503 cefalexin Bacterial cell wall synthesis inhibitor

6587 -3.03 cp BRD-K43860855 iobenguane Antineoplastic

6588 -3.03 cp BRD-A37492983 iocetamic-acid Radiopaque medium

6589 -3.03 cp BRD-A29260609 acebutolol Adrenergic receptor antagonist

6585 -3.02 cp BRD-K52397688 amperozide Dopamine receptor antagonist

6582 -3.01 cp BRD-A62021152 WAY-161503 Serotonin receptor agonist

6580 -3 cp BRD-K40965114 cyanopindolol Adrenergic receptor antagonist

6581 -3 cp BRD-A44008656 doxylamine Histamine receptor antagonist

6577 -2.97 cp BRD-A63836183 PD-123319 Angiotensin receptor antagonist

6576 -2.96 cp BRD-K12260308 xanthoxyline Antifungal

6571 -2.93 cp BRD-K96144918 mead-acid KPL-1 tumor suppressor

6567 -2.92 cp BRD-K06854232 AM-580 Retinoid receptor agonist

6560 -2.89 cp BRD-K46435977 valaciclovir DNA polymerase inhibitor

6561 -2.89 cp BRD-K14536225 piceid Glucosidase inhibitor

6564 -2.89 cp BRD-K53561341 KIN001-220 Aurora kinase inhibitor

6556 -2.87 cp BRD-K10176267 L-701252 Glutamate receptor antagonist

6555 -2.85 cp BRD-K24656285 farnesol FXR agonist

6551 -2.84 cp BRD-A64977602 mirtazapine Adrenergic receptor antagonist

6550 -2.82 cp BRD-K56343971 vemurafenib RAF inhibitor

6543 -2.78 cp BRD-K05181463 L-741626 Dopamine receptor antagonist

6544 -2.78 cp BRD-A41250306 cyclopenthiazide Thiazide diuretic

6545 -2.78 cp BRD-K95992530 Cyclo-[Arg-Gly-Asp-D-Phe-Val] integrin antagonist

6542 -2.77 cp BRD-K28075147 biochanin-a Estrogen receptor agonist

6529 -2.72 cp BRD-K47323024 methapyrilene Histamine receptor antagonist

6528 -2.71 cp BRD-K62056274 quipazine Serotonin receptor agonist

6515 -2.68 cp BRD-K67506692 tyrphostin-AG-126 ERK1 and ERK2 phosphorylation inhibitor

6516 -2.68 cp BRD-K89046952 ciclacillin Bacterial cell wall synthesis inhibitor

6510 -2.65 cp BRD-A07780951 orciprenaline Adrenergic receptor agonist

6506 -2.64 cp BRD-K55034111 pefloxacin Bacterial DNA gyrase inhibitor

6507 -2.64 cp BRD-A59174698 ritodrine Adrenergic receptor agonist

6508 -2.64 cp BRD-K23922020 arecaidine Acetylcholine receptor agonist

6498 -2.62 cp BRD-A39052811 mosapride Serotonin receptor agonist

6491 -2.61 cp BRD-K88358234 xaliproden Serotonin receptor agonist

6492 -2.61 cp BRD-A39747742 estradiol-valerate Estrogen receptor agonist

6493 -2.61 cp BRD-A68929948 DAPT-GSI-IX Gamma secretase inhibitor

6497 -2.61 cp BRD-K48168960 propylthiouracil Thyroid peroxidase inhibitor

6489 -2.6 cp BRD-A01145011 zebularine DNA methyltransferase inhibitor

6478 -2.57 cp BRD-K20197062 SA-94315 Caspase inhibitor

6479 -2.57 cp BRD-A65615053 zacopride Serotonin receptor antagonist

6481 -2.57 cp BRD-K61250553 loperamide Opioid receptor agonist

6468 -2.55 cp BRD-A88254928 salbutamol Adrenergic receptor agonist

6464 -2.54 cp BRD-K28143534 cyproheptadine Histamine receptor antagonist

6465 -2.54 cp BRD-K77677632 SB-200646 Serotonin receptor antagonist

6466 -2.54 cp BRD-A41722204 sulmazole Adenosine receptor antagonist

6467 -2.54 cp BRD-A16665823 butoconazole Bacterial cell wall synthesis inhibitor

6455 -2.48 cp BRD-K66206289 lobeline Acetylcholine receptor antagonist

6447 -2.47 cp BRD-A27489425 rolitetracycline Bacterial 30S ribosomal subunit inhibitor

6448 -2.47 cp BRD-K12906962 dichlorobenzamil Sodium/calcium exchange inhibitor

6449 -2.47 cp BRD-K71430621 clobenpropit Histamine receptor antagonist

6454 -2.47 cp BRD-K68507560 dicycloverine Acetylcholine receptor antagonist

6445 -2.46 cp BRD-A22684332 procaterol Adrenergic receptor agonist

6446 -2.46 cp BRD-K82731415 olomoucine CDK inhibitor

6436 -2.43 cp BRD-K88551539 CAY-10585 HIF modulator

6437 -2.43 cp BRD-K01663662 diphenidol Acetylcholine receptor agonist

6433 -2.4 cp BRD-K19111024 clofibric-acid PPAR receptor agonist

6430 -2.38 cp BRD-K00234327 RU-24969 Serotonin receptor agonist

6426 -2.36 cp BRD-K81128206 edrophonium Acetylcholinesterase inhibitor

6427 -2.36 cp BRD-K56001384 antimycin-a ATP synthase inhibitor

6418 -2.33 cp BRD-K69556541 ryanodine Calcium channel blocker

6419 -2.33 cp BRD-A28746609 paclitaxel Tubulin inhibitor

6408 -2.3 cp BRD-K13642330 cosmosiin Cytochrome P450 inhibitor

6409 -2.3 cp BRD-K91699951 benzonatate Local anesthetic

6405 -2.29 cp BRD-A52172093 VU-0413807-2 Calcium channel blocker

6406 -2.29 cp BRD-K54759182 dosulepin Norepinephrine reuptake inhibitor

6407 -2.29 cp BRD-K37848908 ceforanide Penicillin binding protein inhibitor

6402 -2.27 cp BRD-K32107296 temozolomide DNA alkylating agent

6393 -2.26 cp BRD-K18787491 U-0126 MEK inhibitor

6394 -2.26 cp BRD-K54094468 remoxipride Dopamine receptor antagonist

6390 -2.24 cp BRD-K63343048 orlistat Lipase inhibitor

6392 -2.24 cp BRD-K06080977 eicosatetraynoic-acid Cyclooxygenase inhibitor

6387 -2.22 cp BRD-K26521938 dinoprostone Prostanoid receptor agonist

6370 -2.18 cp BRD-K84955386 hyperoside Glucosidase inhibitor

6371 -2.18 cp BRD-K47761761 PD-168077 Dopamine receptor agonist

6372 -2.18 cp BRD-K26134695 calpeptin Calpain inhibitor

6373 -2.18 cp BRD-A43974499 reboxetine Adrenergic receptor antagonist

6374 -2.18 cp BRD-K37792168 denbufylline Phosphodiesterase inhibitor

6377 -2.18 cp BRD-K35531059 molsidomine Guanylyl cyclase activator

6360 -2.15 cp BRD-K59331372 SB-366791 TRPV antagonist

6361 -2.15 cp BRD-K46018455 bezafibrate PPAR receptor agonist

6348 -2.11 cp BRD-K70511574 sunitinib PLK inhibitor

6347 -2.08 cp BRD-K46056750 AZD-7762 CHK inhibitor

6338 -2.05 cp BRD-A72483914 spiroxatrine Serotonin receptor antagonist

6333 -2.04 cp BRD-A41304429 practolol Adrenergic receptor antagonist

6323 -2.03 cp BRD-A04706586 bucladesine Adenosine receptor agonist

6311 -1.98 cp BRD-K30097969 pitavastatin HMGCR inhibitor

6302 -1.97 cp BRD-K48923948 BMS-641988 Androgen receptor antagonist

6303 -1.97 cp BRD-K76274772 MAPP-D-erythro Ceramidase inhibitor

6304 -1.97 cp BRD-A64125466 dehydrocholic-acid choleretic agent

6305 -1.97 cp BRD-A24817035 laudanosine Central nervous system agent

6309 -1.97 cp BRD-K28912512 nicotinamide Protein synthesis stimulant

6310 -1.97 cp BRD-A36074203 remacemide Glutamate receptor antagonist

6293 -1.94 cp BRD-M72442222 vicriviroc CC chemokine receptor antagonist

6294 -1.94 cp BRD-K67013324 luzindole Melatonin receptor antagonist

6298 -1.94 cp BRD-K93918653 quizartinib FLT3 inhibitor

6291 -1.93 cp BRD-K28115081 apafant Platelet activating factor receptor antagonist

6284 -1.9 cp BRD-A39415247 norethisterone Progesterone receptor agonist

6285 -1.9 cp BRD-A94709349 metaxalone Muscle relaxant

6286 -1.9 cp BRD-K72541103 JAK3-inhibitor-I JAK inhibitor

6271 -1.87 cp BRD-A81402010 KU-C103443N CDC inhibitor

6272 -1.87 cp BRD-A81866333 CGS-21680 Adenosine receptor agonist

6273 -1.87 cp BRD-K04976539 aminogenistein SRC inhibitor

6270 -1.86 cp BRD-K66956375 oleoylethanolamide Cannabinoid receptor agonist

6259 -1.84 cp BRD-K12516989 zaprinast Phosphodiesterase inhibitor

6257 -1.83 cp BRD-A19661776 mianserin Serotonin receptor antagonist

6258 -1.83 cp BRD-K93332168 isocarboxazid Monoamine oxidase inhibitor

6253 -1.81 cp BRD-K50324045 avrainvillamide-analog-6 nucleophosmin inhibitor

6247 -1.8 cp BRD-K93331255 lypressin Vasopressin receptor agonist

6248 -1.8 cp BRD-K43880410 pregnenolone glutamate receptor modulator

6249 -1.8 cp BRD-K67868012 PI-103 MTOR inhibitor

6250 -1.8 cp BRD-K95885906 quercetagetin PIM inhibitor

6251 -1.8 cp BRD-K70778732 trazodone Adrenergic receptor antagonist

6252 -1.8 cp BRD-K92778217 mefenamic-acid Cyclooxygenase inhibitor

6234 -1.76 cp BRD-K75532464 FTI-276 Farnesyltransferase inhibitor

6235 -1.76 cp BRD-K69195780 NAN-190 Serotonin receptor agonist

6236 -1.76 cp BRD-A39969961 eplerenone Cytochrome P450 inhibitor

6237 -1.76 cp BRD-A89672324 CGP-55845 GABA receptor antagonist

6238 -1.76 cp BRD-K08502430 angiogenesis-inhibitor Angiogenesis inhibitor

6225 -1.74 cp BRD-K83023055 GR-135531 Melatonin receptor agonist

6226 -1.74 cp BRD-A80017228 bendroflumethiazide Sodium/potassium/chloride transporter inhibitor

6231 -1.74 cp BRD-A29644307 nomifensine Dopamine uptake inhibitor

6218 -1.73 cp BRD-K13514097 everolimus MTOR inhibitor

6219 -1.73 cp BRD-K04412738 tramadol Norepinephrine reuptake inhibitor

6220 -1.73 cp BRD-K38449220 seneciphylline Cytochrome P450 inhibitor

6221 -1.73 cp BRD-K53959060 indirubin CDK inhibitor

6222 -1.73 cp BRD-K45033733 famciclovir DNA polymerase inhibitor

6223 -1.73 cp BRD-A31521121 methocarbamol Muscle relaxant

6212 -1.69 cp BRD-K97799481 theophylline Adenosine receptor antagonist

6213 -1.69 cp BRD-A56987319 SQ-22536 Adenylyl cyclase inhibitor

6214 -1.69 cp BRD-K89210380 biotin Vitamin B

6204 -1.66 cp BRD-K60476892 YC-1 Guanylyl cyclase activator

6202 -1.64 cp BRD-K89375097 pirenzepine Acetylcholine receptor antagonist

6194 -1.62 cp BRD-K96471533 nitazoxanide Pyruvate ferredoxin oxidoreductase inhibitor

6195 -1.62 cp BRD-A68281735 REV-5901 Leukotriene receptor antagonist

6196 -1.62 cp BRD-K79930101 GW-583340 EGFR inhibitor

6192 -1.6 cp BRD-A48809242 IB-MECA Adenosine receptor agonist

6185 -1.59 cp BRD-K31471398 dihydrexidine Dopamine receptor agonist

6186 -1.59 cp BRD-K82143716 flucytosine Antifungal

6187 -1.59 cp BRD-K36965586 m-chlorophenylbiguanide Serotonin receptor agonist

6188 -1.59 cp BRD-K88849294 lobaric-acid Tyrosine phosphatase inhibitor

6190 -1.59 cp BRD-K93461745 buspirone Serotonin receptor agonist

6191 -1.59 cp BRD-A42571354 cetirizine Histamine receptor antagonist

6183 -1.58 cp BRD-K18895904 olanzapine Dopamine receptor antagonist

6184 -1.58 cp BRD-K63533170 AM-630 Cannabinoid receptor antagonist

6174 -1.55 cp BRD-K32795028 1-benzylimidazole Thromboxane synthase inhibitor

6175 -1.55 cp BRD-K11717138 benzbromarone Chloride channel blocker

6176 -1.55 cp BRD-K99174507 cardiogenol-c Cardiomyogenesis inducer

6172 -1.54 cp BRD-K01253243 SB-590885 RAF inhibitor

6158 -1.52 cp BRD-K23369905 oxiconazole Bacterial cell wall synthesis inhibitor

6159 -1.52 cp BRD-K87696786 LY-456236 Glutamate receptor antagonist

6160 -1.52 cp BRD-K27737647 H-89 PKA inhibitor

6161 -1.52 cp BRD-K53570330 carbofuran Cholinesterase inhibitor

6162 -1.52 cp BRD-A30205217 ethotoin Hydantoin antiepileptic

6157 -1.51 cp BRD-K39569857 avrainvillamide-analog-3 nucleophosmin inhibitor

6149 -1.48 cp BRD-K26674531 GR-235 Estrogen receptor agonist

6150 -1.48 cp BRD-K06467078 corynanthine Adrenergic receptor antagonist

6152 -1.48 cp BRD-K79145749 dibenzepin Norepinephrine reuptake inhibitor

6142 -1.44 cp BRD-A61856038 tremulacin Lipoxygenase inhibitor

6143 -1.44 cp BRD-K99818283 PIK-90 PI3K inhibitor

6144 -1.44 cp BRD-K68065987 MK-2206 AKT inhibitor

6145 -1.44 cp BRD-K73999723 telmisartan Angiotensin receptor antagonist

6137 -1.41 cp BRD-K57011718 UK-356618 Metalloproteinase inhibitor

6138 -1.41 cp BRD-K68332390 ponalrestat Aldose reductase inhibitor

6139 -1.41 cp BRD-K73824630 skatole Thrombin inhibitor

6140 -1.41 cp BRD-A91699651 chloroquine Antimalarial

6131 -1.38 cp BRD-K54330070 SB-202190 p38 MAPK inhibitor

6117 -1.37 cp BRD-K99616396 motesanib KIT inhibitor

6118 -1.37 cp BRD-K26863634 BIX-01338 Histone lysine methyltransferase inhibitor

6119 -1.37 cp BRD-K67831364 ZM-323881 VEGFR inhibitor

6120 -1.37 cp BRD-K69837166 trap-101 Nociceptin/orphanin FQ (NOP) receptor antagonist

6121 -1.37 cp BRD-K12244279 MEK1-2-inhibitor MEK inhibitor

6122 -1.37 cp BRD-A75455249 kavain Calcium channel modulator

6123 -1.37 cp BRD-K81709173 halcinonide Glucocorticoid receptor agonist

6124 -1.37 cp BRD-K40901640 cinanserin Serotonin receptor antagonist

6125 -1.37 cp BRD-A10977446 carvedilol Adrenergic receptor antagonist

6129 -1.37 cp BRD-K28183345 proguanil Dihydrofolate reductase inhibitor

6130 -1.37 cp BRD-K76205745 losartan Angiotensin receptor antagonist

6111 -1.34 cp BRD-K94649603 taxifolin Opioid receptor antagonist

6115 -1.34 cp BRD-K53414658 tivozanib VEGFR inhibitor

6108 -1.33 cp BRD-K49519092 immethridine Histamine receptor agonist

6100 -1.3 cp BRD-A95696066 nisoxetine Norepinephrine reuptake inhibitor

6101 -1.3 cp BRD-K10065684 dantron Laxative

6102 -1.3 cp BRD-A18202423 CPCCOEt Glutamate receptor antagonist

6103 -1.3 cp BRD-A65280694 molindone Dopamine receptor antagonist

6093 -1.27 cp BRD-K61691971 avrainvillamide-analog-1 nucleophosmin inhibitor

6094 -1.27 cp BRD-K82255054 propofol GABA receptor agonist

6095 -1.27 cp BRD-K99107520 felbamate Glutamate receptor antagonist

6096 -1.27 cp BRD-K29668683 BD-1063 Sigma receptor antagonist

6097 -1.27 cp BRD-K43736954 cortisone Glucocorticoid receptor agonist

6098 -1.27 cp BRD-A80213327 NSC-23766 Ras GTPase inhibitor

6089 -1.26 cp BRD-K25224017 pirenperone Serotonin receptor antagonist

6090 -1.26 cp BRD-K30020243 aliskiren Antihypertensive

6074 -1.23 cp BRD-A55594068 vinblastine Microtubule inhibitor

6075 -1.23 cp BRD-K18574842 nafcillin Bacterial cell wall synthesis inhibitor

6076 -1.23 cp BRD-K28578425 cilostamide Phosphodiesterase inhibitor

6078 -1.23 cp BRD-K93280214 gabazine GABA receptor antagonist

6079 -1.23 cp BRD-K02130563 panobinostat HDAC inhibitor

6073 -1.21 cp BRD-K85013741 auraptene Nitric oxide production inhibitor

6065 -1.2 cp BRD-K49481516 galantamine Acetylcholinesterase inhibitor

6066 -1.2 cp BRD-K61314889 IWR-1-ENDO PARP inhibitor

6067 -1.2 cp BRD-K36324071 NF-449 Purinergic receptor antagonist

6068 -1.2 cp BRD-A17453586 MDL-72832 Serotonin receptor agonist

6069 -1.2 cp BRD-K28168037 fenretinide Apoptosis stimulant

6057 -1.17 cp BRD-K95309561 dienestrol Estrogen receptor agonist

6050 -1.16 cp BRD-A19248578 latrunculin-b Actin polymerization inhibitor

6051 -1.16 cp BRD-K19525698 ozagrel Thromboxane synthase inhibitor

6052 -1.16 cp BRD-K99595596 salsolinol Monoamine oxidase inhibitor

6055 -1.16 cp BRD-A97701745 pindolol Adrenergic receptor antagonist

6056 -1.16 cp BRD-K50859149 sulfafurazole Bacterial antifolate

6049 -1.14 cp BRD-K94441233 mevastatin HMGCR inhibitor

6043 -1.13 cp BRD-K60623809 SU-11652 Tyrosine kinase inhibitor

6044 -1.13 cp BRD-K10136726 tosyllysyl-chloromethyl-ketone Chymotrypsin inhibitor

6048 -1.13 cp BRD-K85119730 tolbutamide ATP channel blocker

6030 -1.09 cp BRD-K71035033 masitinib KIT inhibitor

6031 -1.09 cp BRD-K62996583 lidoflazine Calcium channel blocker

6032 -1.09 cp BRD-A13650332 flucloxacillin Bacterial cell wall synthesis inhibitor

6033 -1.09 cp BRD-A73581086 ergometrine Adrenergic receptor agonist

6034 -1.09 cp BRD-A75402480 desoxycorticosterone Mineralocorticoid receptor agonist

6019 -1.06 cp BRD-A63546914 RO-04-5595 Glutamate receptor antagonist

6020 -1.06 cp BRD-K32755366 reserpic-acid Norepinephrine transporter inhibitor

6021 -1.06 cp BRD-K48367671 febuxostat Xanthine oxidase inhibitor

6025 -1.06 cp BRD-K68264559 brimonidine Adrenergic receptor agonist

6026 -1.06 cp BRD-K47983010 BX-795 IKK inhibitor

6027 -1.06 cp BRD-K11742128 triprolidine Histamine receptor antagonist

6015 -1.04 cp BRD-K68103045 CGS-20625 Benzodiazepine receptor agonist

6013 -1.03 cp BRD-K28307902 flutamide Androgen receptor antagonist

6006 -1.02 cp BRD-K64857848 XMD-885 Leucine rich repeat kinase inhibitor

6007 -1.02 cp BRD-K26160755 RS-45041-190 Imidazoline receptor agonist

6008 -1.02 cp BRD-K92678294 irilin-a Isoflavone

6009 -1.02 cp BRD-K40887525 ritanserin Serotonin receptor antagonist

6011 -1.02 cp BRD-K29458283 chlorambucil DNA inhibitor

6012 -1.02 cp BRD-A93255169 thalidomide TNF production inhibitor

6004 -1 cp BRD-K82109576 vincristine Tubulin inhibitor

5995 -0.99 cp BRD-K82091397 SB-239063 p38 MAPK inhibitor

5996 -0.99 cp BRD-A52193669 withaferin-a IKK inhibitor

5997 -0.99 cp BRD-K01436366 XMD-1150 Leucine rich repeat kinase inhibitor

5998 -0.99 cp BRD-K39462424 dexchlorpheniramine Histamine receptor antagonist

5999 -0.99 cp BRD-A62025033 temsirolimus MTOR inhibitor

6000 -0.99 cp BRD-K01567962 pyrazolanthrone JNK inhibitor

6001 -0.99 cp BRD-K02590140 O-2050 Cannabinoid receptor antagonist

6002 -0.99 cp BRD-K46384212 o-3M3FBS phospholipase activator

6003 -0.99 cp BRD-K25394294 oxaprozin Cyclooxygenase inhibitor

5994 -0.98 cp BRD-K67578145 GDC-0879 RAF inhibitor

5980 -0.95 cp BRD-K85503079 perospirone Dopamine receptor antagonist

5981 -0.95 cp BRD-K88677950 PD-198306 MAP kinase inhibitor

5982 -0.95 cp BRD-A50928468 norgestrel Progesterone receptor agonist

5983 -0.95 cp BRD-K38903228 hesperidin Flavanone glycoside

5985 -0.95 cp BRD-A70083328 secnidazole Acetylcholinesterase inhibitor

5979 -0.94 cp BRD-K14965640 ibuprofen Cyclooxygenase inhibitor

5969 -0.92 cp BRD-K94270326 ecopipam Dopamine receptor antagonist

5970 -0.92 cp BRD-K71860425 CDK2-5-inhibitor CDK inhibitor

5971 -0.92 cp BRD-K34014345 naproxol Anti-inflammatory

5965 -0.88 cp BRD-A26384407 chlortalidone Carbonic anhydrase inhibitor

5952 -0.85 cp BRD-K76568384 PHTPP Estrogen receptor antagonist

5953 -0.85 cp BRD-K52721684 PCO-400 Potassium channel activator

5954 -0.85 cp BRD-K90789829 nefazodone Adrenergic inhibitor

5955 -0.85 cp BRD-K21936341 oxotremorine Acetylcholine receptor agonist

5956 -0.85 cp BRD-K53737926 amitriptyline Norepinephrine inhibitor

5957 -0.85 cp BRD-K36862742 hydroflumethiazide Sodium/potassium/chloride transporter inhibitor

5950 -0.84 cp BRD-K96740444 itopride Dopamine receptor antagonist

5951 -0.84 cp BRD-K23583188 lavendustin-a EGFR inhibitor

5944 -0.82 cp BRD-A93659613 GR-89696 Opioid receptor agonist

5937 -0.81 cp BRD-K66896231 BRD-K66896231 Acetylcholinesterase inhibitor

5938 -0.81 cp BRD-K93095519 SJ-172550 MDM inhibitor

5939 -0.81 cp BRD-A47633927 NPC-15199 ICAM1 antagonist

5941 -0.81 cp BRD-K14993104 bemegride Chemoreceptor agonist

5942 -0.81 cp BRD-K72903603 zidovudine Reverse transcriptase inhibitor

5943 -0.81 cp BRD-K66788707 fludarabine DNA synthesis inhibitor

5930 -0.78 cp BRD-K04196797 oxcarbazepine Sodium channel blocker

5931 -0.78 cp BRD-A68888262 azelastine Histamine receptor antagonist

5932 -0.78 cp BRD-A97674275 ranolazine Sodium channel blocker

5933 -0.78 cp BRD-K67439147 SIB-1893 Glutamate receptor antagonist

5934 -0.78 cp BRD-K52075715 oxibendazole Tubulin inhibitor

5927 -0.76 cp BRD-A31195449 TCB2 Serotonin receptor agonist

5916 -0.74 cp BRD-K10466330 AVA Nucleophosmin inhibitor

5920 -0.74 cp BRD-A16444946 acarbose Glucosidase inhibitor

5921 -0.74 cp BRD-K31792052 pifithrin Interleukin receptor antagonist

5922 -0.74 cp BRD-K44067360 flufenamic-acid Chloride channel blocker

5923 -0.74 cp BRD-K94080537 diethyltoluamide DEET activator of fly antenna ionotropic receptor IR40a

5924 -0.74 cp BRD-K15616905 CCCP Mitochondrial oxidative phosphorylation uncoupler

5925 -0.74 cp BRD-A54236247 racephedrine Adrenergic receptor agonist

5926 -0.74 cp BRD-A50764878 MDL-73005EF Serotonin receptor antagonist

5912 -0.71 cp BRD-K29178788 dictamnine Furoquinoline alkaloid

5913 -0.71 cp BRD-K09668667 benzo(a)pyrene Carcinogen

5906 -0.7 cp BRD-K64994968 progesterone Progesterone receptor agonist

5907 -0.7 cp BRD-K06159959 CCMQ Inhibitor of the binding of homoquinolinic acid to non-NMDA sensitive sites

5908 -0.7 cp BRD-A56012032 thiorphan Membrane metalloendopeptidase inhibitor

5909 -0.7 cp BRD-K54771420 glycocholic-acid Cholesterol inhibitor

5910 -0.7 cp BRD-K80527266 triacsin-c Adrenergic receptor antagonist

5893 -0.67 cp BRD-A24381660 zeranol Estrogen receptor agonist

5894 -0.67 cp BRD-K60511616 pravastatin HMGCR inhibitor

5895 -0.67 cp BRD-K42221274 NNC-711 GAT inhibitor

5896 -0.67 cp BRD-A78877355 nefopam Cyclooxygenase inhibitor

5897 -0.67 cp BRD-A95939040 sertaconazole Sterol demethylase inhibitor

5898 -0.67 cp BRD-A36267905 buphenine Adrenergic receptor agonist

5899 -0.67 cp BRD-K73109821 diazoxide Potassium channel activator

5900 -0.67 cp BRD-K09907482 PRL-3-inhibitor-I Tyrosine phosphatase inhibitor

5878 -0.63 cp BRD-K68437527 EMF-bca1-60 caspase inhibitor

5879 -0.63 cp BRD-K73589491 nizatidine Histamine receptor antagonist

5880 -0.63 cp BRD-A35511923 L-803087 Somatostatin receptor agonist

5881 -0.63 cp BRD-K71103788 duloxetine Serotonin reuptake inhibitor

5884 -0.63 cp BRD-K63430059 methoxsalen DNA synthesis inhibitor

5885 -0.63 cp BRD-A17655518 ibuprofen Cyclooxygenase inhibitor

5886 -0.63 cp BRD-K79602928 metformin Insulin sensitizer

5887 -0.63 cp BRD-K02637541 celecoxib Cyclooxygenase inhibitor

5888 -0.63 cp BRD-K80738081 resveratrol Cytochrome P450 inhibitor

5868 -0.6 cp BRD-K20338176 cefaclor Bacterial cell wall synthesis inhibitor

5869 -0.6 cp BRD-K16406336 methylene-blue Guanylyl cyclase inhibitor

5870 -0.6 cp BRD-K34581968 BMS-536924 IGF-1 inhibitor

5871 -0.6 cp BRD-K16664969 GTP-14564 FLT3 inhibitor

5872 -0.6 cp BRD-K34170797 fexaramine FXR agonist

5873 -0.6 cp BRD-K78294846 osthol Calcium channel blocker

5875 -0.6 cp BRD-K36927236 glibenclamide Sulfonylurea

5876 -0.6 cp BRD-K42095107 daidzein Estrogen receptor agonist

5858 -0.56 cp BRD-K49671696 ketanserin Serotonin receptor antagonist

5865 -0.56 cp BRD-A09056319 alfuzosin Adrenergic receptor antagonist

5866 -0.56 cp BRD-K88789588 letrozole Aromatase inhibitor

5855 -0.55 cp BRD-K74765201 tomelukast Leukotriene receptor antagonist

5848 -0.53 cp BRD-K86887724 dofetilide Potassium channel blocker

5849 -0.53 cp BRD-K63874012 thioperamide Histamine receptor antagonist

5850 -0.53 cp BRD-K93201660 ML-7 Myosin light chain kinase inhibitor

5851 -0.53 cp BRD-K27450477 EHNA Adenosine deaminase inhibitor

5852 -0.53 cp BRD-K55468218 spiperone Dopamine receptor antagonist

5853 -0.53 cp BRD-K39746403 erythromycin NFkB pathway inhibitor

5854 -0.53 cp BRD-A87606379 nadolol Adrenergic receptor antagonist

5846 -0.51 cp BRD-K50866992 tropisetron Serotonin receptor antagonist

5835 -0.49 cp BRD-K50938786 ropivacaine Sodium channel blocker

5836 -0.49 cp BRD-K60219430 serdemetan MDM inhibitor

5837 -0.49 cp BRD-K23383398 T-0901317 LXR agonist

5838 -0.49 cp BRD-K09397065 SR-57227A Serotonin receptor agonist

5839 -0.49 cp BRD-K73391359 quinisocaine Local anesthetic

5840 -0.49 cp BRD-K81729199 AQ-RA741 Acetylcholine receptor antagonist

5841 -0.49 cp BRD-K37206356 rhamnetin HDAC inhibitor

5842 -0.49 cp BRD-K21548250 moracizine Sodium channel blocker

5826 -0.46 cp BRD-K68997413 PF-3845 FAAH inhibitor

5827 -0.46 cp BRD-K96402602 farnesylthiotriazole PPMTase inhibitor

5828 -0.46 cp BRD-K47717570 NBQX Glutamate receptor antagonist

5830 -0.46 cp BRD-K47207162 zimelidine Serotonin reuptake inhibitor

5805 -0.42 cp BRD-A34817987 itraconazole Cytochrome P450 inhibitor

5806 -0.42 cp BRD-K54708045 nTZDpa PPAR receptor agonist

5807 -0.42 cp BRD-K23335153 AMN-082 Glutamate receptor modulator

5808 -0.42 cp BRD-K53220666 trimetozine Sedative

5809 -0.42 cp BRD-K46678324 RHO-kinase-inhibitor-II Rho associated kinase inhibitor

5810 -0.42 cp BRD-A97104540 fenoterol Adrenergic receptor agonist

5811 -0.42 cp BRD-K63945320 dihydrosamidin Phospholipase inhibitor

5812 -0.42 cp BRD-K14221570 benzopurpurin-4b HIV entry inhibitor

5813 -0.42 cp BRD-A15131297 benazepril ACE inhibitor

5816 -0.42 cp BRD-K37865504 LY-2183240 FAAH inhibitor

5817 -0.42 cp BRD-A98299281 velnacrine cholinesterase inhibitor

5791 -0.39 cp BRD-K28029915 dolasetron Serotonin receptor antagonist

5792 -0.39 cp BRD-K52172416 anastrozole Aromatase inhibitor

5793 -0.39 cp BRD-K02113016 olaparib PARP inhibitor

5794 -0.39 cp BRD-K95676198 JAK3-inhibitor-V JAK inhibitor

5795 -0.39 cp BRD-K75958195 pizotifen Serotonin receptor antagonist

5796 -0.39 cp BRD-K19227686 phenolphthalein Indicator dye

5797 -0.39 cp BRD-K29582677 flunarizine Calcium channel blocker

5798 -0.39 cp BRD-A04553218 chlorphenamine Histamine receptor antagonist

5799 -0.39 cp BRD-A46393198 tetramisole Immunostimulant

5800 -0.39 cp BRD-A29734509 disopyramide Sodium channel blocker

5801 -0.39 cp BRD-K54256913 MK-1775 WEE1 kinase inhibitor

5802 -0.39 cp BRD-K89732114 trifluoperazine Dopamine receptor antagonist

5803 -0.39 cp BRD-A70407468 PSB-36 Adenosine receptor antagonist

5785 -0.36 cp BRD-A02990301 lofexidine Adrenergic receptor agonist

5781 -0.35 cp BRD-K17953061 staurosporine PKC inhibitor

5782 -0.35 cp BRD-A70649075 sulconazole Sterol demethylase inhibitor

5783 -0.35 cp BRD-K40624912 ZM-39923 JAK inhibitor

5784 -0.35 cp BRD-K67783091 haloperidol Dopamine receptor antagonist

5780 -0.34 cp BRD-A45498368 WYE-125132 MTOR inhibitor

5768 -0.32 cp BRD-K56957086 dacinostat HDAC inhibitor

5769 -0.32 cp BRD-K86191271 cytosporone-b NUR77 receptor agonist

5770 -0.32 cp BRD-K06208435 YS-035 Calcium channel blocker

5771 -0.32 cp BRD-A41833852 naloxone Opioid receptor antagonist

5772 -0.32 cp BRD-K47936004 piribedil Dopamine receptor agonist

5773 -0.32 cp BRD-A89175223 bisoprolol Adrenergic receptor antagonist

5774 -0.32 cp BRD-A68723818 brompheniramine Histamine receptor antagonist

5775 -0.32 cp BRD-A50311610 meclozine CAR agonist

5776 -0.32 cp BRD-K75089421 procainamide Sodium channel blocker

5777 -0.32 cp BRD-K37289225 clozapine Dopamine receptor antagonist

5778 -0.32 cp BRD-A66435872 HTMT Histamine receptor agonist

5757 -0.28 cp BRD-K48932581 cetraxate Mucus protecting agent

5758 -0.28 cp BRD-K55591206 epigallocatechin Nitric oxide synthase inhibitor

5759 -0.28 cp BRD-K32696739 noreleagnine Monoamine oxidase inhibitor

5760 -0.28 cp BRD-A30815329 felodipine Calcium channel blocker

5761 -0.28 cp BRD-K82561139 ricinine Casein kinase inhibitor

5762 -0.28 cp BRD-A62525898 prednisone Glucocorticoid receptor agonist

5763 -0.28 cp BRD-K62929068 6-benzylaminopurine Purinergic receptor activator

5766 -0.28 cp BRD-K60762818 desipramine Tricyclic antidepressant

5747 -0.25 cp BRD-A26334849 propafenone Antiarrhythmic

5748 -0.25 cp BRD-A81177136 KN-62 Calcium-calmodulin dependent protein kinase inhibitor

5749 -0.25 cp BRD-K04046242 equilin Estrogen receptor agonist

5750 -0.25 cp BRD-K69600043 thiethylperazine Dopamine receptor antagonist

5751 -0.25 cp BRD-A40639672 ketorolac Cyclooxygenase inhibitor

5752 -0.25 cp BRD-K57222227 indometacin Cyclooxygenase inhibitor

5753 -0.25 cp BRD-A58048407 nimodipine Calcium channel blocker

5754 -0.25 cp BRD-K79116891 proxymetacaine Sodium channel blocker

5735 -0.21 cp BRD-K41051431 mecillinam Bacterial cell wall synthesis inhibitor

5736 -0.21 cp BRD-K76908866 CP-724714 EGFR inhibitor

5737 -0.21 cp BRD-K00603606 ticlopidine Purinergic receptor antagonist

5738 -0.21 cp BRD-A78942461 ICI-118551 Adrenergic receptor antagonist

5739 -0.21 cp BRD-K06926592 tretinoin Retinoid receptor agonist

5740 -0.21 cp BRD-K47869605 podophyllotoxin Microtubule inhibitor

5741 -0.21 cp BRD-K79254416 decitabine DNA methyltransferase inhibitor

5734 -0.2 cp BRD-K04170657 psoromic-acid Ras GTPase inhibitor

5725 -0.18 cp BRD-K00615600 AG-14361 PARP inhibitor

5726 -0.18 cp BRD-A10303790 talampicillin Bacterial cell wall synthesis inhibitor

5727 -0.18 cp BRD-K33818169 GW-3965 LXR agonist

5728 -0.18 cp BRD-A55416093 colforsin Adenylyl cyclase activator

5730 -0.18 cp BRD-K29950728 clomifene Estrogen receptor antagonist

5724 -0.15 cp BRD-K21733600 rofecoxib Cyclooxygenase inhibitor

5717 -0.14 cp BRD-K15567136 papaverine Phosphodiesterase inhibitor

5718 -0.14 cp BRD-K80315159 DPPE Histamine receptor antagonist

5719 -0.14 cp BRD-K51662849 ilomastat Matrix metalloprotease inhibitor

5720 -0.14 cp BRD-K08252256 diclofenac Cyclooxygenase inhibitor

5721 -0.14 cp BRD-K70327191 benzoxiquine Anti-infective

5723 -0.14 cp BRD-A08187463 racecadotril Enkephalinase inhibitor

5706 -0.11 cp BRD-K53972329 ruxolitinib JAK inhibitor

5707 -0.11 cp BRD-K57569181 pentoxifylline Phosphodiesterase inhibitor

5708 -0.11 cp BRD-K64614248 salicin Anti-inflammatory

5709 -0.11 cp BRD-K10974103 diloxanide Protein synthesis inhibitor

5710 -0.11 cp BRD-K56429665 calcipotriol Vitamin D receptor agonist

5711 -0.11 cp BRD-K97810537 beclometasone Glucocorticoid receptor agonist

5702 -0.08 cp BRD-K62959606 sphingosine Ceramidase inhibitor

5693 -0.07 cp BRD-A49172652 lansoprazole ATPase inhibitor

5694 -0.07 cp BRD-K77771411 moxonidine Imidazoline receptor agonist

5695 -0.07 cp BRD-A16332958 modafinil Adrenergic receptor agonist

5696 -0.07 cp BRD-K34776109 glimepiride Insulin secretagogue

5697 -0.07 cp BRD-K91290917 amodiaquine Histamine receptor agonist

5698 -0.07 cp BRD-K32821942 azathioprine Dehydrogenase inhibitor

5699 -0.07 cp BRD-A09472452 flecainide Sodium channel blocker

5700 -0.07 cp BRD-K92723993 imatinib BCR-ABL kinase inhibitor

5687 -0.04 cp BRD-K92760278 riboflavin Vitamin B

5688 -0.04 cp BRD-A64092382 mexiletine Sodium channel blocker

5677 -0.03 cp BRD-K11540476 EMF-BCA1-64 Caspase inhibitor

5678 -0.03 cp BRD-K06221026 DUP-697 Cyclooxygenase inhibitor

5679 -0.03 cp BRD-A33168282 sotalol Adrenergic receptor antagonist

5680 -0.03 cp BRD-K03842655 penitrem-a Potassium channel blocker

5681 -0.03 cp BRD-K39915878 loxapine Dopamine receptor antagonist

5682 -0.03 cp BRD-K28936863 ketotifen Histamine receptor agonist

5684 -0.03 cp BRD-A29485665 bicalutamide Androgen receptor antagonist

5685 -0.03 cp BRD-A31159102 fluoxetine Selective serotonin reuptake inhibitor (SSRI)

5686 -0.03 cp BRD-A91008255 bepridil Calcium channel blocker

5668 -0.02 cp BRD-K15916496 clotrimazole Cytochrome P450 inhibitor

5669 -0.02 cp BRD-A22380646 pantoprazole ATPase inhibitor

5670 -0.02 cp BRD-K19352500 prochlorperazine Dopamine receptor antagonist

5671 -0.02 cp BRD-K49111258 prazosin Adrenergic receptor antagonist

5672 -0.02 cp BRD-K78126613 menadione Mitochondrial DNA polymerase inhibitor

5665 -0.01 cp BRD-K86301799 dipyridamole Phosphodiesterase inhibitor

3269 0 cp BRD-K86873305 piperacillin Bacterial cell wall synthesis inhibitor

3270 0 cp BRD-K82216340 medroxyprogesterone progesterone receptor agonist

3271 0 cp BRD-K90864987 cobalt(II)-chloride HSP inducer

3272 0 cp BRD-K67977190 eprosartan Angiotensin receptor antagonist

3273 0 cp BRD-K56450366 NSC-94258 Antineoplastic

3274 0 cp BRD-K14643723 4-(2-Amino-ethyl)-benzenesulfonamide carbonic anhydrase inhibitor

3275 0 cp BRD-K06750613 GSK-1059615 PI3K inhibitor

3276 0 cp BRD-K04466929 Merck60 HDAC inhibitor

3277 0 cp BRD-A83892713 rifampicin RNA polymerase inhibitor

3278 0 cp BRD-A62890442 3-methyl-GABA GABA aminotransferase activator

3279 0 cp BRD-A29731977 17-hydroxyprogesterone-caproate progesterone receptor agonist

3280 0 cp BRD-K36627727 tamibarotene Retinoid receptor agonist

3281 0 cp BRD-K54472332 elvitegravir HIV integrase inhibitor

3282 0 cp BRD-K89014967 AS-703026 MEK inhibitor

3283 0 cp BRD-K91696562 orantinib FGFR inhibitor

3284 0 cp BRD-K07265709 razoxane Chelating agent

3285 0 cp BRD-K17306061 aprepitant Tachykinin antagonist

3286 0 cp BRD-A41692738 TGX-221 PI3K inhibitor

3287 0 cp BRD-K16485616 mocetinostat HDAC inhibitor

3288 0 cp BRD-K02965346 SU-11274 Hepatocyte growth factor receptor inhibitor

3289 0 cp BRD-K99749624 linifanib PDGFR receptor inhibitor

3290 0 cp BRD-K00337317 NU-7441 DNA dependent protein kinase inhibitor

3291 0 cp BRD-K64800655 PHA-793887 CDK inhibitor

3292 0 cp BRD-K99545815 PF-562271 Focal adhesion kinase inhibitor

3293 0 cp BRD-K86465814 HO-013 PPAR receptor agonist

3294 0 cp BRD-A39646320 HC-toxin HDAC inhibitor

3295 0 cp BRD-K98684188 GSK-0660 PPAR receptor antagonist

3296 0 cp BRD-K75181824 acetyl-geranygeranyl-cysteine Inhibitor of methyl esterification of geranylgeranylated proteins

3297 0 cp BRD-A18497530 5-iodotubercidin Adenosine kinase inhibitor

3298 0 cp BRD-K99291625 SB-203580 p38 MAPK inhibitor

3299 0 cp BRD-K56064827 EI-273 PKC inhibitor

3300 0 cp BRD-K32710582 EI-247 IGF-1 inhibitor

3301 0 cp BRD-K80725632 lavendustin-c EGFR inhibitor

3302 0 cp BRD-K47943470 tyrphostin-51 EGFR inhibitor

3303 0 cp BRD-K35128472 2-aminopurine Serine/threonine kinase inhibitor

3304 0 cp BRD-K30743633 TCPOBOP CAR agonist

3305 0 cp BRD-K81062487 taurocholic-acid Bile acid

3306 0 cp BRD-K18135438 chenodeoxycholic-acid 11-beta-HSD1 inhibitor

3307 0 cp BRD-K41170226 deoxycholic-acid G protein-coupled receptor agonist

3308 0 cp BRD-K39944607 ochratoxin-a Phenylalanyl tRNA synthetase inhibitor

3309 0 cp BRD-K04923131 GSK-3-inhibitor-IX Glycogen synthase kinase inhibitor

3310 0 cp BRD-A11702965 chromomycin-a3 DNA binding agent

3311 0 cp BRD-K63915849 AS-604850 PI3K inhibitor

3312 0 cp BRD-U51951544 ZG-10 JNK inhibitor

3313 0 cp BRD-K83972459 JWE-035 Aurora kinase inhibitor

3314 0 cp BRD-K38615104 A-443644 AKT inhibitor

3315 0 cp BRD-A60245366 AS-601245 JNK inhibitor

3316 0 cp BRD-A68589262 troxipide Glucosamine synthetase stimulant

3317 0 cp BRD-K68202742 trichostatin-a HDAC inhibitor

3318 0 cp BRD-K29653726 topiramate Carbonic anhydrase inhibitor

3319 0 cp BRD-K89125793 tinidazole Antiprotozoal

3320 0 cp BRD-A72441487 stiripentol GABA uptake inhibitor

3321 0 cp BRD-K30563334 rifabutin Protein synthesis inhibitor

3322 0 cp BRD-A56245458 reichstein Androgen receptor antagonist

3323 0 cp BRD-K25310650 ormetoprim Bacterial antifolate

3324 0 cp BRD-K83144676 olmesartan Angiotensin antagonist

3325 0 cp BRD-K57930253 nitrazepam Benzodiazepine receptor agonist

3326 0 cp BRD-K88679075 methandriol Androgenic steroid

3327 0 cp BRD-A03216249 mepivacaine Potassium channel blocker

3328 0 cp BRD-K62858456 lomerizine Calcium channel blocker

3329 0 cp BRD-K22227508 targinine Nitric oxide synthase inhibitor

3330 0 cp BRD-K37516142 idebenone Calcium channel modulator

3331 0 cp BRD-K59570838 homoveratrylamine Dopamine analog

3332 0 cp BRD-K33312228 halometasone Glucocorticoid receptor agonist

3333 0 cp BRD-K00673382 famotidine Histamine receptor antagonist

3334 0 cp BRD-K99447003 enalaprilat ACE inhibitor

3335 0 cp BRD-K63265447 docetaxel Tubulin inhibitor

3336 0 cp BRD-K90976994 dehydrocholic-acid Bile acid

3337 0 cp BRD-K86595100 chlordiazepoxide Benzodiazepine receptor agonist

3338 0 cp BRD-K52735702 cefdinir Bacterial cell wall synthesis inhibitor

3339 0 cp BRD-A59985574 topotecan Topoisomerase inhibitor

3340 0 cp BRD-K24681473 YM-155 Survivin inhibitor

3341 0 cp BRD-K05926469 lenalidomide Antineoplastic

3342 0 cp BRD-M07438658 lapatinib EGFR inhibitor

3343 0 cp BRD-K21971034 OM-137 Aurora kinase inhibitor

3344 0 cp BRD-A77050075 heraclenol Vitamin K antagonist

3345 0 cp BRD-K79090631 CGP-60474 CDK inhibitor

3346 0 cp BRD-K15402119 huperzine-a Acetylcholinesterase inhibitor

3347 0 cp BRD-K14550461 doxercalciferol Vitamin D receptor agonist

3348 0 cp BRD-A47706533 L-BSO Glutathione transferase inhibitor

3349 0 cp BRD-A74771556 nikkomycin Chitin inhibitor

3350 0 cp BRD-A61858259 CAY-10415 Insulin sensitizer

3351 0 cp BRD-A00520476 otenzepad Acetylcholine receptor antagonist

3352 0 cp BRD-K56745457 azauridine Antiviral

3353 0 cp BRD-K09485525 GANT-61 GLI antagonist

3354 0 cp BRD-K40619305 larixinic-acid Compound that interacts with metal centers

3355 0 cp BRD-A67438293 treprostinil Prostacyclin analog

3356 0 cp BRD-K54987996 CAY-10578 Casein kinase inhibitor

3357 0 cp BRD-U82589721 HG-5-113-01 Protein kinase inhibitor

3358 0 cp BRD-U37049823 HG-6-64-01 RAF inhibitor

3359 0 cp BRD-K74761218 WT-171 HDAC inhibitor

3360 0 cp BRD-K65910366 KUC103904N -666

3361 0 cp BRD-K48115423 2-(4-methoxybenzylthio)-6-methylpyrimidin-4-ol Matrix metalloprotease inhibitor

3362 0 cp BRD-K22828899 TUL-XXI039 Serine/threonine kinase inhibitor

3363 0 cp BRD-K13810148 givinostat HDAC inhibitor

3364 0 cp BRD-K11663430 pyroxamide HDAC inhibitor

3365 0 cp BRD-K28346421 rifapentine RNA polymerase inhibitor

3366 0 cp BRD-K43149758 myricetin Androgen receptor agonist

3367 0 cp BRD-K36153907 KU-C103885 Cystic fibrosis transmembrane conductance regulator inhibitor

3368 0 cp BRD-A73680854 PT-630 Dipeptidyl peptidase inhibitor

3369 0 cp BRD-K69840642 ISOX HDAC inhibitor

3370 0 cp BRD-K01779529 fluoropyruvate Pyruvate dehydrogenase kinase inhibitor

3371 0 cp BRD-K02950022 BMS-299897 Gamma secretase inhibitor

3372 0 cp BRD-K99696746 fatostatin SREBP inhibitor

3373 0 cp BRD-K28907958 CD-437 Retinoid receptor agonist

3374 0 cp BRD-A36630025 SN-38 Topoisomerase inhibitor

3375 0 cp BRD-K43797669 genistein Tyrosine kinase inhibitor

3376 0 cp BRD-A65550283 ginsenoside Steroid hormone receptor agonist

3377 0 cp BRD-A31801025 formestane Aromatase inhibitor

3378 0 cp BRD-K33882852 ZK-93423 Benzodiazepine receptor agonist

3379 0 cp BRD-K11373525 ZD-7155 Angiotensin receptor antagonist

3380 0 cp BRD-K64157027 ZD-2079 Adrenergic receptor agonist

3381 0 cp BRD-K90259198 W-7 Calmodulin antagonist

3382 0 cp BRD-A84389633 "tropanyl-3,5-dimethylbenzoate" Serotonin receptor antagonist

3383 0 cp BRD-K00959089 thenoyltrifluoroacetone Chelating agent

3384 0 cp BRD-K45988865 tetramethylsilane Internal standard for NMR spectroscopy

3385 0 cp BRD-A33833419 TER-14687 Inhibitor of translocation of PKCq in T cells

3386 0 cp BRD-K51805276 temefos Cholinesterase inhibitor

3387 0 cp BRD-K22503835 scriptaid HDAC inhibitor

3388 0 cp BRD-A10715913 sulpiride Dopamine receptor antagonist

3389 0 cp BRD-K63504947 semaxanib VEGFR inhibitor

3390 0 cp BRD-K72895815 SSR-69071 Leukocyte elastase inhibitor

3391 0 cp BRD-K35430135 SR-59230A Adrenergic receptor antagonist

3392 0 cp BRD-A09828896 SKF-81297 Dopamine receptor agonist

3393 0 cp BRD-A31007383 SDZ-WAG-994 Adenosine receptor agonist

3394 0 cp BRD-K04414442 SB-222200 Tachykinin antagonist

3395 0 cp BRD-K83637872 SANT-1 Smoothened receptor antagonist

3396 0 cp BRD-K06426971 ryuvidine Histone lysine methyltransferase inhibitor

3397 0 cp BRD-A49370193 RO-60-0175 Serotonin receptor agonist

3398 0 cp BRD-K80778372 RO-19-4605 GABA benzodiazepine site receptor inverse agonist

3399 0 cp BRD-K00486786 RO-08-2750 NGF binding inhibitor

3400 0 cp BRD-K21853356 RG-14620 EGFR inhibitor

3401 0 cp BRD-A19053259 pseudopelletierine Anthelmintic

3402 0 cp BRD-A37817666 picrotoxin GABA receptor antagonist

3403 0 cp BRD-K11399644 phenformin AMPK activator

3404 0 cp BRD-K88429204 pyrimethamine Dihydrofolate reductase inhibitor

3405 0 cp BRD-K16977723 PP-3 EGFR inhibitor

3406 0 cp BRD-K94689771 pinocembrin CYP1B1 inhibitor

3407 0 cp BRD-A43882281 pinacidil ATP channel activator

3408 0 cp BRD-K10843433 phenylbutazone Cyclooxygenase inhibitor

3409 0 cp BRD-A89337244 PD-102807 Acetylcholine receptor antagonist

3410 0 cp BRD-K16195444 oxymetazoline Adrenergic receptor agonist

3411 0 cp BRD-A10355991 norketamine Glutamate receptor antagonist

3412 0 cp BRD-K74141488 naftifine Fungal squalene epoxidase inhibitor

3413 0 cp BRD-K85015012 NNC-05-2090 GAT inhibitor

3414 0 cp BRD-A35338386 NECA Adenosine receptor agonist

3415 0 cp BRD-A23683907 NAS-181 Serotonin receptor antagonist

3416 0 cp BRD-K60060639 methyllidocaine antiarrhythmic medication

3417 0 cp BRD-K34441861 moexipril ACE inhibitor

3418 0 cp BRD-A32949107 MRS-1845 Calcium channel blocker

3419 0 cp BRD-K53878242 MMPX Phosphodiesterase inhibitor

3420 0 cp BRD-K95202259 ML-3163 p38 MAPK inhibitor

3421 0 cp BRD-K60230970 MG-132 Proteasome inhibitor

3422 0 cp BRD-K09764130 mead-ethanolamide Cannabinoid receptor agonist

3423 0 cp BRD-A85587465 bemesetron Serotonin receptor antagonist

3424 0 cp BRD-K13261168 LY-16350 Dopamine receptor agonist

3425 0 cp BRD-K64044582 linoleamide ACAT inhibitor

3426 0 cp BRD-K15791587 L-733060 Tachykinin antagonist

3427 0 cp BRD-A96882008 L-732138 Tachykinin antagonist

3428 0 cp BRD-K40656405 L-165041 PPAR receptor agonist

3429 0 cp BRD-A42553870 L-152804 Neuropeptide receptor antagonist

3430 0 cp BRD-K05464208 JX-401 p38 MAPK inhibitor

3431 0 cp BRD-K37080523 isoreserpine Vesicular monoamine transporter inhibitor

3432 0 cp BRD-A54029483 IRL-2500 Endothelin receptor antagonist

3433 0 cp BRD-K01815685 indole aryl hydrocarbon receptor agonist

3434 0 cp BRD-K07117950 imperatorin CDK inhibitor

3435 0 cp BRD-K66782112 BRD-K66782112 Histamine receptor antagonist

3436 0 cp BRD-K76674262 homoharringtonine Protein synthesis inhibitor

3437 0 cp BRD-K08554278 bisbenzimide DNA binding agent

3438 0 cp BRD-K07325606 hispidin PKC inhibitor

3439 0 cp BRD-K81209159 herniarin Acetylcholinesterase inhibitor

3440 0 cp BRD-K82983861 GW-0742 PPAR receptor agonist

3441 0 cp BRD-K67860401 AR-A014418 Glycogen synthase kinase inhibitor

3442 0 cp BRD-K40578143 GR-79236 Adenosine receptor agonist

3443 0 cp BRD-K02283807 GR-32191 Thromboxane receptor antagonist

3444 0 cp BRD-K12120659 GR-144053 Integrin antagonist

3445 0 cp BRD-K11634954 GBR-13069 Dopamine uptake inhibitor

3446 0 cp BRD-K87049188 fusaric-acid Dopamine beta hydroxylase inhibitor

3447 0 cp BRD-M00539986 formoterol Adrenergic receptor agonist

3448 0 cp BRD-A49734948 foliosidine Plant alkaloid

3449 0 cp BRD-K98769987 flumazenil Benzodiazepine receptor antagonist

3450 0 cp BRD-A50684349 fenoldopam Dopamine receptor agonist

3451 0 cp BRD-K66944906 fraxidin Carbonic anhydrase inhibitor

3452 0 cp BRD-K47693913 evoxine Furoquinoline alkaloid

3453 0 cp BRD-K81839095 estrone Estrogen receptor agonist

3454 0 cp BRD-K86727142 embelin HCV inhibitor

3455 0 cp BRD-K46068882 eugenitol Bacterial quorum sensing inhibitor

3456 0 cp BRD-K19360254 ergocornine Dopamine receptor agonist

3457 0 cp BRD-K11927976 ER-27319 Mediator release inhibitor

3458 0 cp BRD-K88759641 EMD-66684 Angiotensin receptor antagonist

3459 0 cp BRD-K18619710 digoxigenin Steroid

3460 0 cp BRD-A88282067 delcorine Antiarrhythmic

3461 0 cp BRD-K33459542 ditolylguanidine Sigma receptor agonist

3462 0 cp BRD-K94920105 DR-2313 PARP inhibitor

3463 0 cp BRD-A27143604 DPN Estrogen receptor agonist

3464 0 cp BRD-K85266041 DNQX Glutamate receptor antagonist

3465 0 cp BRD-K06014311 DH-97 Melatonin receptor antagonist

3466 0 cp BRD-K32526544 DCEBIO Potassium channel activator

3467 0 cp BRD-K74430258 "1,2-dichlorobenzene" Hepatotoxicant

3468 0 cp BRD-K26997899 SA-792574 Microtubule inhibitor

3469 0 cp BRD-K54142781 cirazoline Adrenergic receptor agonist

3470 0 cp BRD-A49358627 ciprofibrate PPAR receptor agonist

3471 0 cp BRD-K72816382 cinalukast Leukotriene receptor antagonist

3472 0 cp BRD-A90311807 cilastatin Dehydropeptidase inhibitor

3473 0 cp BRD-A30437061 camptothecin Topoisomerase inhibitor

3474 0 cp BRD-K67680372 CI-966 GAT inhibitor

3475 0 cp BRD-A04668240 CGP-52432 GABA receptor antagonist

3476 0 cp BRD-A31575449 CGP-20712 Adrenergic receptor antagonist

3477 0 cp BRD-K10870738 CDC Lipoxygenase inhibitor

3478 0 cp BRD-K03600606 catechin Beta secretase inhibitor

3479 0 cp BRD-A17411484 carprofen Cyclooxygenase inhibitor

3480 0 cp BRD-A98702003 carbenoxolone 11-beta-HSD1 inhibitor

3481 0 cp BRD-A58157837 butabindide Tripeptidyl peptidase inhibitor

3482 0 cp BRD-K07507905 BRL-37344 Adrenergic receptor agonist

3483 0 cp BRD-A38913120 BH3I-1 BCL inhibitor

3484 0 cp BRD-K75615183 talipexole Adrenergic receptor agonist

3485 0 cp BRD-K91370081 anisomycin DNA synthesis inhibitor

3486 0 cp BRD-K88611939 aniracetam Glutamate receptor agonist

3487 0 cp BRD-K36638830 anabasine Acetylcholine receptor agonist

3488 0 cp BRD-K97181089 amiloride Sodium channel blocker

3489 0 cp BRD-K78280988 anandamide Cannabinoid receptor agonist

3490 0 cp BRD-K70330367 amantadine Glutamate receptor antagonist

3491 0 cp BRD-K33204703 AG-370 PDGFR receptor inhibitor

3492 0 cp BRD-K87919739 tyrphostin-AG-825 Receptor tyrosine protein kinase inhibitor

3493 0 cp BRD-K80348542 cephaeline Protein synthesis inhibitor

3494 0 cp BRD-K05906022 limonin HIV protease inhibitor

3495 0 cp BRD-A95445494 maackiain Sodium/glucose cotransporter inhibitor

3496 0 cp BRD-K06712146 YM-90709 IL5 inhibitor

3497 0 cp BRD-A22844106 tenoxicam Cyclooxygenase inhibitor

3498 0 cp BRD-A77299732 salubrinal Eukaryotic translation initiation factor inhibitor

3499 0 cp BRD-K58299615 RO-90-7501 Beta amyloid inhibitor

3500 0 cp BRD-K51541829 RO-25-6981 Ionotropic glutamate receptor antagonist

3501 0 cp BRD-K42142750 retrorsine Antimitotic

3502 0 cp BRD-A71765365 mepireserpate Catecholamine depleting sympatholytic

3503 0 cp BRD-A34255068 rolipram Phosphodiesterase inhibitor

3504 0 cp BRD-K54411430 robustic-acid cAMP inhibitor

3505 0 cp BRD-K13725475 rhodomyrtoxin Cytotoxic agent

3506 0 cp BRD-K46137903 prednicarbate Phospholipase activator

3507 0 cp BRD-K46317332 proadifen Nitric oxide synthase inhibitor

3508 0 cp BRD-A01643550 prednisolone Glucocorticoid receptor agonist

3509 0 cp BRD-K21350491 phenamil TRPV antagonist

3510 0 cp BRD-A48257147 PHCCC Glutamate receptor agonist

3511 0 cp BRD-A45333398 periplocymarin Apoptosis stimulant

3512 0 cp BRD-K92731339 perindopril ACE inhibitor

3513 0 cp BRD-K31987754 oleylethanolamide Cannabinoid receptor agonist

3514 0 cp BRD-K59637651 NSC-119889 Protein synthesis inhibitor

3515 0 cp BRD-A31204924 mitotane Antineoplastic

3516 0 cp BRD-K35941380 methysergide Serotonin receptor antagonist

3517 0 cp BRD-K62792802 LY-83583 Guanylyl cyclase inhibitor

3518 0 cp BRD-K27305650 LY-294002 MTOR inhibitor

3519 0 cp BRD-A85472596 L-670596 Prostanoid receptor antagonist

3520 0 cp BRD-K39733634 L-161982 Prostanoid receptor antagonist

3521 0 cp BRD-K47150025 KI-8751 VEGFR inhibitor

3522 0 cp BRD-A10903566 imiloxan Adrenergic receptor antagonist

3523 0 cp BRD-K29673530 hypericin Tyrosine kinase inhibitor

3524 0 cp BRD-A65767837 hydrocortisone Glucocorticoid receptor agonist

3525 0 cp BRD-K96263742 GW-7647 PPAR receptor agonist

3526 0 cp BRD-A15415227 GW-1929 PPAR receptor agonist

3527 0 cp BRD-A90515964 guaifenesin Expectorant

3528 0 cp BRD-A29082194 gitoxigenin ATPase inhibitor

3529 0 cp BRD-A38749782 fludrocortisone Glucocorticoid receptor agonist

3530 0 cp BRD-K48722258 dilazep Adenosine reuptake inhibitor

3531 0 cp BRD-K38305202 domperidone Dopamine receptor antagonist

3532 0 cp BRD-K13819402 desoxypeganine Acetylcholinesterase inhibitor

3533 0 cp BRD-A17819071 gedunin HSP inhibitor

3534 0 cp BRD-K30240666 clemastine Histamine receptor antagonist

3535 0 cp BRD-K41707108 ceramide Phosphoenolpyruvate carboxylase activator

3536 0 cp BRD-K56509348 BMS-182874 Endothelin receptor antagonist

3537 0 cp BRD-K04877770 FTase-inhibitor-B581 Farnesyltransferase inhibitor

3538 0 cp BRD-K56558538 ambroxol Sodium channel blocker

3539 0 cp BRD-K43405658 tyrphostin-AG-527 Protein tyrosine kinase inhibitor

3540 0 cp BRD-A07824748 flavanone 11-beta-HSD1 inhibitor

5589 0 cp BRD-K04887706 AKT-inhibitor-1-2 AKT inhibitor

5590 0 cp BRD-K36740062 GSK-1070916 Aurora kinase inhibitor

5591 0 cp BRD-K49669041 BX-912 Pyruvate dehydrogenase kinase inhibitor

5592 0 cp BRD-K64890080 BI-2536 PLK inhibitor

5593 0 cp BRD-K77908580 entinostat HDAC inhibitor

5594 0 cp BRD-K88742110 BRD-K88742110 HDAC inhibitor

5595 0 cp BRD-K50417881 eticlopride Dopamine receptor antagonist

5596 0 cp BRD-K53857191 risperidone Dopamine receptor antagonist

5597 0 cp BRD-A16311756 profenamine Butyrylcholinesterase inhibitor

5598 0 cp BRD-K21450440 benzthiazide Carbonic anhydrase inhibitor

5599 0 cp BRD-K66876909 linezolid Bacterial 50S ribosomal subunit inhibitor

5600 0 cp BRD-K36616567 doxepin Histamine receptor antagonist

5601 0 cp BRD-A42759514 ornidazole Antiprotozoal

5602 0 cp BRD-K12513978 fenbufen Cyclooxygenase inhibitor

5603 0 cp BRD-K55044200 amoxicillin Penicillin binding protein inhibitor

5604 0 cp BRD-A84481105 thioridazine Dopamine receptor antagonist

5605 0 cp BRD-A55815733 phylloquinone Vitamin K

5606 0 cp BRD-K15262564 mupirocin Isoleucyl-tRNA synthetase inhibitor

5607 0 cp BRD-K82381502 acetylcholine Acetylcholine receptor agonist

5608 0 cp BRD-A80638690 floxuridine DNA synthesis inhibitor

5609 0 cp BRD-A16478930 amcinonide Glucocorticoid receptor agonist

5610 0 cp BRD-K02123250 JNJ-38877605 Tyrosine kinase inhibitor

5611 0 cp BRD-K06335600 tizanidine Adrenergic receptor agonist

5612 0 cp BRD-A75479906 rimantadine Antiviral

5613 0 cp BRD-K70976396 cefoxitin Bacterial cell wall synthesis inhibitor

5614 0 cp BRD-K74763371 bosentan Endothelin receptor antagonist

5615 0 cp BRD-K52522949 NCH-51 HDAC inhibitor

5616 0 cp BRD-A51382177 fosinopril ACE inhibitor

5617 0 cp BRD-A04308630 genipin Choleretic agent

5618 0 cp BRD-K67102207 phenylbutyrate HDAC inhibitor

5619 0 cp BRD-K11433652 aspirin Cyclooxygenase inhibitor

5620 0 cp BRD-A13188892 doxazosin Adrenergic receptor antagonist

5621 0 cp BRD-A87848830 bimatoprost Prostanoid receptor agonist

5622 0 cp BRD-K11905747 spectinomycin Bacterial 30S ribosomal subunit inhibitor

5623 0 cp BRD-A79237180 ascorbic-acid Antioxidant

5624 0 cp BRD-A67605442 tetrahydrobiopterin Nitric oxide stimulant

5625 0 cp BRD-K53790871 triamcinolone Glucocorticoid receptor agonist

5626 0 cp BRD-K10016611 pyridine-2-aldoxime Acetylcholinesterase inhibitor

5627 0 cp BRD-K11801786 trimidox Ribonucleotide reductase inhibitor

5628 0 cp BRD-K21520694 sulfacetamide PABA antagonist

5629 0 cp BRD-K55930204 phenytoin Hydantoin antiepileptic

5630 0 cp BRD-A23290232 westcort Glucocorticoid receptor agonist

5631 0 cp BRD-K91601245 mercaptopurine Immunosuppressant

5632 0 cp BRD-K22662435 ganciclovir DNA polymerase inhibitor

5633 0 cp BRD-K62363391 dapsone Bacterial antifolate

5634 0 cp BRD-K20920669 cromoglicic-acid Immunosuppressant

5635 0 cp BRD-K24994810 androstenol GABA receptor modulator

5636 0 cp BRD-K32836707 CAY-10577 Casein kinase inhibitor

5637 0 cp BRD-K89839824 raltitrexed Thymidylate synthase inhibitor

5638 0 cp BRD-K17743125 belinostat HDAC inhibitor

5639 0 cp BRD-A02333338 cyclopamine Smoothened receptor antagonist

5640 0 cp BRD-K74133369 oligomycin-a ATP synthase inhibitor

5641 0 cp BRD-K19416115 sitagliptin Dipeptidyl peptidase inhibitor

5642 0 cp BRD-K31553034 zibotentan Endothelin receptor antagonist

5643 0 cp BRD-A46179541 doxapram Potassium channel blocker

5644 0 cp BRD-K05673000 dicloxacillin Bacterial cell wall synthesis inhibitor

5645 0 cp BRD-K27721098 clopidogrel Purinergic receptor antagonist

5646 0 cp BRD-A69512159 carbidopa Aromatic L-amino acid decarboxylase inhibitor

5647 0 cp BRD-K68756823 FR-180204 -666

5648 0 cp BRD-K09499853 KU-0060648 DNA dependent protein kinase inhibitor

5649 0 cp BRD-K81418486 vorinostat HDAC inhibitor

5650 0 cp BRD-K35723520 darinaparsin Apoptosis stimulant

5651 0 cp BRD-K11853856 PJ-34 PARP inhibitor

5652 0 cp BRD-K48692744 NU-1025 PARP inhibitor

5653 0 cp BRD-K46211610 tolazoline Adrenergic receptor antagonist

5654 0 cp BRD-K26979635 NS-3694 Glutamate receptor antagonist

5655 0 cp BRD-A16700644 isoxsuprine Adrenergic receptor agonist

5656 0 cp BRD-A52326238 isogedunin HSP inhibitor

5657 0 cp BRD-A63998256 helveticoside ATPase inhibitor

5658 0 cp BRD-A09539288 homatropine Acetylcholine receptor antagonist

5659 0 cp BRD-A73859745 glycodeoxycholic-acid Apoptosis stimulant

2699 0.01 cp BRD-K57179821 crotamiton Antipruritic

2700 0.01 cp BRD-K13032584 procarbazine Monoamine oxidase inhibitor

2683 0.02 cp BRD-K68867920 quetiapine Dopamine receptor antagonist

2684 0.02 cp BRD-A49160188 donepezil Acetylcholinesterase inhibitor

2685 0.02 cp BRD-K62200014 anagrelide Phosphodiesterase inhibitor

2686 0.02 cp BRD-K17561142 amiodarone Potassium channel blocker

2687 0.02 cp BRD-K25311561 KU-55933 ATM kinase inhibitor

2688 0.02 cp BRD-A10523515 GSK-429286A Rho associated kinase inhibitor

2689 0.02 cp BRD-A48430263 pioglitazone Insulin sensitizer

2690 0.02 cp BRD-A75769826 SDM25N Opioid receptor antagonist

2673 0.03 cp BRD-K66874953 pifithrin-alpha TP53 inhibitor

2674 0.03 cp BRD-K32501161 vanoxerine Dopamine uptake inhibitor

2675 0.03 cp BRD-K08547377 irinotecan Topoisomerase inhibitor

2676 0.03 cp BRD-K67017579 cilostazol Phosphodiesterase inhibitor

2677 0.03 cp BRD-K05395900 nicotine Acetylcholine receptor agonist

2666 0.04 cp BRD-A02710418 meptazinol Opioid receptor agonist

2667 0.04 cp BRD-K18909381 CGS-12066B Serotonin receptor agonist

2668 0.04 cp BRD-A54880345 etomidate GABA receptor modulator

2660 0.07 cp BRD-K81528515 nilotinib ABL inhibitor

2661 0.07 cp BRD-K66353228 zoxazolamine Myorelaxant

2662 0.07 cp BRD-K63675182 triflupromazine Dopamine receptor antagonist

2644 0.11 cp BRD-A01787639 naftopidil Adrenergic receptor antagonist

2645 0.11 cp BRD-K64245000 GW-4064 FXR agonist

2646 0.11 cp BRD-A93424738 dexamethasone Glucocorticoid receptor agonist

2647 0.11 cp BRD-K39987650 bisacodyl Laxative

2648 0.11 cp BRD-A27554692 altrenogest Progestogen hormone

2650 0.11 cp BRD-K55127134 fluphenazine Dopamine receptor antagonist

2651 0.11 cp BRD-K79131256 albendazole Anthelmintic

2652 0.11 cp BRD-A35588707 teniposide Topoisomerase inhibitor

2653 0.11 cp BRD-K38436528 imipramine Norepinephrine reuptake inhibitor

2631 0.14 cp BRD-K28849549 mesalazine Cyclooxygenase inhibitor

2632 0.14 cp BRD-K08417745 SID-26681509 Cathepsin inhibitor

2633 0.14 cp BRD-K13049116 BMS-754807 IGF-1 inhibitor

2634 0.14 cp BRD-K01095011 finasteride 5-alpha reductase inhibitor

2635 0.14 cp BRD-K82036761 sertraline Serotonin receptor antagonist

2636 0.14 cp BRD-A57382968 piroxicam Cyclooxygenase inhibitor

2637 0.14 cp BRD-A92177080 betamethasone Glucocorticoid receptor agonist

2640 0.14 cp BRD-K65417056 meprylcaine Local anesthetic

2641 0.14 cp BRD-K81473089 tacrine Acetylcholinesterase inhibitor

2616 0.18 cp BRD-K78431006 crizotinib ALK inhibitor

2617 0.18 cp BRD-K18816859 L-694247 Serotonin receptor agonist

2618 0.18 cp BRD-A11678676 wortmannin PI3K inhibitor

2619 0.18 cp BRD-K54210043 NS-1619 Calcium channel activator

2620 0.18 cp BRD-K19136521 indirubin CDK inhibitor

2621 0.18 cp BRD-A22769835 homochlorcyclizine Antihistamine

2622 0.18 cp BRD-K96084870 DMBI PDGFR receptor inhibitor

2623 0.18 cp BRD-K42500029 CGP-57380 MAP kinase inhibitor

2624 0.18 cp BRD-K20482099 rutin Antioxidant

2625 0.18 cp BRD-K59369769 tozasertib Aurora kinase inhibitor

2626 0.18 cp BRD-A45889380 mepacrine Cytokine production inhibitor

2603 0.21 cp BRD-K72420232 WZ-4002 EGFR inhibitor

2604 0.21 cp BRD-A41451487 PK-11195 Benzodiazepine receptor antagonist

2605 0.21 cp BRD-K21667562 AM-404 Cyclooxygenase inhibitor

2606 0.21 cp BRD-K84663978 trequinsin Phosphodiesterase inhibitor

2607 0.21 cp BRD-K94512704 spiramide Dopamine receptor antagonist

2608 0.21 cp BRD-K03670461 tyrphostin-AG-82 EGFR inhibitor

2609 0.21 cp BRD-A67799922 phenoxybenzamine Adrenergic receptor antagonist

2610 0.21 cp BRD-K92000912 AM-251 Cannabinoid receptor antagonist

2611 0.21 cp BRD-A09533288 verapamil Calcium channel blocker

2594 0.25 cp BRD-K47278471 diphenhydramine Histamine receptor antagonist

2595 0.25 cp BRD-K74112339 acetohydroxamic-acid Urease inhibitor

2596 0.25 cp BRD-A63043573 cabergoline Dopamine receptor agonist

2597 0.25 cp BRD-K77695569 tiabendazole Angiogenesis inhibitor

2592 0.26 cp BRD-A44133049 azasetron Serotonin receptor antagonist

2583 0.28 cp BRD-K09963420 saquinavir HIV protease inhibitor

2584 0.28 cp BRD-K90524085 MY-5445 Phosphodiesterase inhibitor

2585 0.28 cp BRD-K59574735 ubenimex Leukotriene inhibitor

2586 0.28 cp BRD-K30480208 torasemide Electrolyte reabsorption inhibitor

2587 0.28 cp BRD-A72988804 tiaprofenic-acid Cyclooxygenase inhibitor

2588 0.28 cp BRD-K35498378 alrestatin Aldose reductase inhibitor

2589 0.28 cp BRD-K37991163 paroxetine Selective serotonin reuptake inhibitor (SSRI)

2590 0.28 cp BRD-K85090592 pilocarpine Acetylcholine receptor agonist

2582 0.29 cp BRD-A29426959 carbinoxamine Histamine receptor antagonist

2579 0.32 cp BRD-K29359156 ebselen H+/K+-ATPase inhibitor

2580 0.32 cp BRD-K11163873 phenanthridone PARP inhibitor

2581 0.32 cp BRD-K47659338 EMD-386088 Serotonin receptor agonist

2569 0.35 cp BRD-K21283037 riluzole Glutamate inhibitor

2570 0.35 cp BRD-K73397362 purmorphamine Smoothened receptor agonist

2571 0.35 cp BRD-A71262238 nafadotride Dopamine receptor antagonist

2572 0.35 cp BRD-K71499074 diclofenamide Carbonic anhydrase inhibitor

2573 0.35 cp BRD-A16694057 bisphenol-a PPAR receptor antagonist

2565 0.38 cp BRD-K44442813 pidotimod Interferon receptor agonist

2557 0.39 cp BRD-K82562631 tolmetin Cyclooxygenase inhibitor

2558 0.39 cp BRD-K85603128 resorcinol Phosphodiesterase inhibitor

2559 0.39 cp BRD-K13544237 r(-)-propylnorapomorphine Dopamine receptor agonist

2560 0.39 cp BRD-A54490543 pirlindole Monoamine oxidase inhibitor

2561 0.39 cp BRD-A92537424 danazol Estrogen receptor antagonist

2563 0.39 cp BRD-K07237224 moclobemide Monoamine oxidase inhibitor

2564 0.39 cp BRD-K98530306 clonidine Adrenergic receptor agonist

2548 0.42 cp BRD-K63923597 barasertib Aurora kinase inhibitor

2549 0.42 cp BRD-K04853698 LDN-193189 Serine/threonine kinase inhibitor

2550 0.42 cp BRD-K89402695 L-655240 Thromboxane receptor antagonist

2551 0.42 cp BRD-K45252063 clofibrate PPAR receptor agonist

2553 0.42 cp BRD-A83937277 mephenytoin Hydantoin antiepileptic

2554 0.42 cp BRD-A09722536 cyclophosphamide DNA alkylating agent

2555 0.42 cp BRD-K03816923 rottlerin MAP kinase inhibitor

2545 0.43 cp BRD-K45296539 ZD-7114 Adrenergic receptor agonist

2542 0.45 cp BRD-K47328134 lysylphenylalanyl-tyrosine Heparin activation inhibitor

2528 0.46 cp BRD-A13084692 troglitazone Insulin sensitizer

2529 0.46 cp BRD-K34154330 tracazolate GABA receptor modulator

2530 0.46 cp BRD-A11605036 thiocolchicoside GABA receptor antagonist

2531 0.46 cp BRD-K26429091 J-104129 Acetylcholine receptor antagonist

2532 0.46 cp BRD-K87158025 benzamil Sodium channel blocker

2533 0.46 cp BRD-K40919711 BAPTA-AM Potassium channel blocker

2534 0.46 cp BRD-A67862938 naftidrofuryl Adrenergic receptor antagonist

2535 0.46 cp BRD-K60038276 irbesartan Angiotensin receptor antagonist

2536 0.46 cp BRD-A05186015 bupropion Dopamine uptake inhibitor

2539 0.46 cp BRD-A60197193 amisulpride Dopamine receptor antagonist

2540 0.46 cp BRD-K89997465 chlorpromazine Dopamine receptor antagonist

2541 0.46 cp BRD-K12994359 valdecoxib Cyclooxygenase inhibitor

2524 0.49 cp BRD-K59332007 linopirdine Potassium channel blocker

2525 0.49 cp BRD-A93206962 L-755507 Adrenergic receptor agonist

2513 0.53 cp BRD-K99411983 lumicolchicine "Colchicine isomer, non-binder of microtubules"

2514 0.53 cp BRD-K79366068 PSB-069 NTPDase inhibitor

2515 0.53 cp BRD-K50311478 tosyl-phenylalanyl-chloromethyl-ketone Chymotrypsin inhibitor

2516 0.53 cp BRD-K49049886 CGS-15943 Adenosine receptor antagonist

2517 0.53 cp BRD-K22193694 dioxybenzone Topical sunscreen agent

2518 0.53 cp BRD-K77641333 naphazoline Adrenergic receptor agonist

2519 0.53 cp BRD-K38197229 bumetanide Solute carrier family member inhibitor

2508 0.56 cp BRD-A81772229 simvastatin HMGCR inhibitor

2509 0.56 cp BRD-A78295502 hydroquinine Antiarrhythmic

2506 0.57 cp BRD-K98490050 amsacrine Topoisomerase inhibitor

2496 0.6 cp BRD-K02227374 milnacipran Serotonin reuptake inhibitor

2497 0.6 cp BRD-K88304388 dextrorphan Glutamate receptor antagonist

2498 0.6 cp BRD-A02481876 importazole Importin-beta transport receptor inhibitor

2499 0.6 cp BRD-K40902647 vincamine Adrenergic receptor antagonist

2500 0.6 cp BRD-K68873215 phosphodiesterase-V-inhibitor-II Phosphodiesterase inhibitor

2501 0.6 cp BRD-K06198550 isorotenone Mitochondrial complex I inhibitor

2502 0.6 cp BRD-K51476772 ST-638 Tyrosine kinase inhibitor

2503 0.6 cp BRD-K52313696 tacedinaline HDAC inhibitor

2504 0.6 cp BRD-K63630713 etacrynic-acid Sodium/potassium/chloride transporter inhibitor

2494 0.62 cp BRD-A98378129 talniflumate Cyclooxygenase inhibitor

2487 0.63 cp BRD-K74733595 APHA-compound-8 HDAC inhibitor

2488 0.63 cp BRD-K10961822 latanoprost Prostanoid receptor agonist

2489 0.63 cp BRD-K96134740 kitasamycin Protein synthesis inhibitor

2490 0.63 cp BRD-A60294240 tribenoside Anti-inflammatory

2491 0.63 cp BRD-A72758037 asiatic-acid Apoptosis stimulant

2475 0.67 cp BRD-K94887716 TFMPP Serotonin receptor agonist

2476 0.67 cp BRD-K02526760 QS-11 ARFGAP inhibitor

2477 0.67 cp BRD-K14765469 vesamicol Acetylcholinesterase inhibitor

2478 0.67 cp BRD-K29173907 isoflupredone Glucocorticoid receptor agonist

2479 0.67 cp BRD-K31611373 fluprostenol Prostanoid receptor agonist

2480 0.67 cp BRD-K49945136 GR-113808 Serotonin receptor antagonist

2481 0.67 cp BRD-A41519720 ezetimibe Niemann-Pick C1-like 1 protein antagonist

2482 0.67 cp BRD-K86509404 iso-olomoucine CDK inhibitor

2464 0.7 cp BRD-K09471561 levofloxacin Bacterial DNA gyrase inhibitor

2465 0.7 cp BRD-K98493452 honokiol AKT inhibitor

2466 0.7 cp BRD-A21723284 naltriben Opioid receptor antagonist

2467 0.7 cp BRD-K93460210 lamotrigine Serotonin receptor antagonist

2468 0.7 cp BRD-A51820102 econazole Bacterial cell wall synthesis inhibitor

2469 0.7 cp BRD-A92161634 scopoline Acetylcholine receptor antagonist

2470 0.7 cp BRD-K00627859 tubastatin-a HDAC inhibitor

2457 0.74 cp BRD-A84189516 baccatin-III Paclitaxel precursor

2461 0.74 cp BRD-K77925998 quipazine Serotonin receptor agonist

2453 0.76 cp BRD-A10420615 cyclopiazonic-acid ATPase inhibitor

2449 0.78 cp BRD-K18523449 mestanolone Androgenic steroid

2450 0.78 cp BRD-A70449690 forskolin Adenylyl cyclase activator

2451 0.78 cp BRD-K06593056 LE-135 Retinoid receptor agonist

2433 0.81 cp BRD-K41260949 valproic-acid HDAC inhibitor

2434 0.81 cp BRD-K63165456 norcyclobenzaprine Adrenergic receptor agonist

2435 0.81 cp BRD-K06753942 nobiletin MEK inhibitor

2436 0.81 cp BRD-A91866971 SQ-29548 Thromboxane receptor antagonist

2437 0.81 cp BRD-A02713983 dihydrodeoxygedunin Growth factor receptor activator

2438 0.81 cp BRD-K93188295 ARC-239 Adrenergic receptor antagonist

2439 0.81 cp BRD-K90027355 spironolactone Mineralocorticoid receptor antagonist

2440 0.81 cp BRD-A86044036 flurbiprofen Cyclooxygenase inhibitor

2432 0.82 cp BRD-K64341947 CFM-1571 Guanylate cyclase activator

2419 0.85 cp BRD-K00532621 midazolam Benzodiazepine receptor agonist

2420 0.85 cp BRD-A31227688 kynuramine Aryl hydrocarbon receptor activator

2421 0.85 cp BRD-K85383046 IAA-94 Chloride channel blocker

2422 0.85 cp BRD-K71534238 GW-9508 Free fatty acid receptor agonist

2423 0.85 cp BRD-A98431941 ephedrine Adrenergic receptor agonist

2424 0.85 cp BRD-K26657438 imiquimod TLR agonist

2425 0.85 cp BRD-A45543382 metrizamide Radiopaque medium

2412 0.88 cp BRD-K16277217 piperacetazine Dopamine receptor antagonist

2413 0.88 cp BRD-K83597974 pargyline Monoamine oxidase inhibitor

2414 0.88 cp BRD-K14282469 LY-165163 Serotonin receptor antagonist

2405 0.89 cp BRD-K40742111 baeomycesic-acid Lipoxygenase inhibitor

2408 0.89 cp BRD-K15563106 phloretin Sodium/glucose cotransporter inhibitor

2401 0.92 cp BRD-M41783010 acamprosate Glutamate receptor antagonist

2402 0.92 cp BRD-K61217870 n-(3-acetamidophenyl)-3-chlorobenzamide Glutamate receptor antagonist

2403 0.92 cp BRD-K57033106 tripelennamine Histamine receptor antagonist

2404 0.92 cp BRD-K89055274 alverine Muscle relaxant

2394 0.95 cp BRD-A43930669 L-368899 Oxytocin receptor antagonist

2397 0.95 cp BRD-K16554956 PTB1 AMPK activator

2391 0.97 cp BRD-K16336526 capsaicin TRPV agonist

2383 0.99 cp BRD-K10670311 sulfasalazine Antirheumatic

2384 0.99 cp BRD-K04146668 GW-441756 Growth factor receptor inhibitor

2385 0.99 cp BRD-K07220430 cinnarizine Calcium channel blocker

2386 0.99 cp BRD-A17535965 gelsemine Acetylcholine receptor antagonist

2387 0.99 cp BRD-K45330754 diethylstilbestrol Estrogen receptor agonist

2389 0.99 cp BRD-K29313308 HDAC3-selective HDAC inhibitor

2390 0.99 cp BRD-K16478699 PLX-4720 RAF inhibitor

2372 1.02 cp BRD-K51485625 ritonavir HIV protease inhibitor

2373 1.02 cp BRD-A42423104 benproperine Antitussive

2374 1.02 cp BRD-K29905972 axitinib PDGFR receptor inhibitor

2375 1.02 cp BRD-K53263234 CITCO CAR agonist

2377 1.02 cp BRD-K20655524 mefexamide Psychoactive drug

2378 1.02 cp BRD-K55748775 SCH-28080 ATPase inhibitor

2369 1.05 cp BRD-K81916719 triclabendazole Microtubule inhibitor

2364 1.06 cp BRD-A47829399 artesunate DNA synthesis inhibitor

2365 1.06 cp BRD-K67298865 SB-431542 TGF beta receptor inhibitor

2366 1.06 cp BRD-A41145729 methoprene-acid Retinoid receptor agonist

2367 1.06 cp BRD-K75699339 rizatriptan Serotonin receptor agonist

2368 1.06 cp BRD-K47639036 flavoxate Acetylcholine receptor antagonist

2356 1.09 cp BRD-K20986251 lithocholic-acid FXR antagonist

2357 1.09 cp BRD-K48029790 OBAA Phospholipase inhibitor

2358 1.09 cp BRD-A87719232 naproxen Cyclooxygenase inhibitor

2359 1.09 cp BRD-K95851186 CGP-13501 GABA receptor modulator

2360 1.09 cp BRD-K02581333 protein-tyrosine-phosphatase-inhibitor-IV Tyrosine phosphatase inhibitor

2348 1.13 cp BRD-A84134924 pancuronium Acetylcholine receptor antagonist

2349 1.13 cp BRD-A49046702 SKF-89976A GABA uptake inhibitor

2350 1.13 cp BRD-K43330982 JTE-013 Lysophospholipid receptor antagonist

2351 1.13 cp BRD-K24240364 GYKI-52466 Glutamate receptor antagonist

2352 1.13 cp BRD-A34706053 CGP-12177 Adrenergic receptor agonist

2342 1.16 cp BRD-K99498722 NPI-2358 Tubulin inhibitor

2343 1.16 cp BRD-K95901403 XL-147 PI3K inhibitor

2346 1.16 cp BRD-K76723084 isotretinoin Retinoid receptor agonist

2347 1.16 cp BRD-A79672927 tropicamide Acetylcholine receptor antagonist

2333 1.2 cp BRD-A70731303 avrainvillamide-analog-5 nucleophosmin inhibitor

2335 1.2 cp BRD-K18905250 ST-91 Adrenergic receptor agonist

2336 1.2 cp BRD-K04111260 raclopride Dopamine receptor antagonist

2327 1.22 cp BRD-K80451230 zamifenacin Acetylcholine receptor antagonist

2319 1.23 cp BRD-A26097136 bulleyaconitine-a Non-opiod analgesic

2320 1.23 cp BRD-K12932420 YM-976 Phosphodiesterase inhibitor

2321 1.23 cp BRD-K68341547 W-9 Calmodulin antagonist

2322 1.23 cp BRD-A95096829 PNU-96415E Dopamine receptor antagonist

2323 1.23 cp BRD-A03816571 CP-55940 Cannabinoid receptor agonist

2324 1.23 cp BRD-K80396088 gliquidone Sulfonylurea

2325 1.23 cp BRD-A21858158 praziquantel Anthelmintic

2308 1.27 cp BRD-K62982419 cilomilast Phosphodiesterase inhibitor

2309 1.27 cp BRD-A79465854 auranofin NFkB pathway inhibitor

2310 1.27 cp BRD-K13646352 midostaurin FLT3 inhibitor

2311 1.27 cp BRD-K91623615 ABT-751 Tubulin inhibitor

2301 1.3 cp BRD-K04833372 GSK-1904529A IGF-1 inhibitor

2302 1.3 cp BRD-K72024482 MRS-1754 Adenosine receptor antagonist

2303 1.3 cp BRD-K43245338 MDL-28170 Calpain inhibitor

2304 1.3 cp BRD-K62353524 DY-131 Estrogen receptor agonist

2297 1.31 cp BRD-K97330509 SRC-kinase-inhibitor-II SRC inhibitor

2298 1.31 cp BRD-K26548821 quinpirole Dopamine receptor agonist

2289 1.34 cp BRD-A02180903 betamethasone Glucocorticoid receptor agonist

2292 1.34 cp BRD-K78959463 FPL-64176 Calcium channel activator

2287 1.36 cp BRD-K41337261 ZM-306416 ABL inhibitor

2280 1.37 cp BRD-K37814297 acepromazine Dopamine receptor antagonist

2281 1.37 cp BRD-K64402243 ivachtin Caspase inhibitor

2282 1.37 cp BRD-K65503129 HSP90-inhibitor HSP inhibitor

2283 1.37 cp BRD-K31054881 BMY-7378 Adrenergic receptor antagonist

2284 1.37 cp BRD-K17008822 BD-1008 Sigma receptor antagonist

2285 1.37 cp BRD-A72066420 mifobate PPAR receptor antagonist

2277 1.38 cp BRD-A98283014 calmidazolium Calcium channel blocker

2278 1.38 cp BRD-K63516691 T-0156 Phosphodiesterase inhibitor

2270 1.39 cp BRD-A60070924 alpha-estradiol Estrogen receptor agonist

2271 1.39 cp BRD-K63792901 arecaidine Acetylcholine receptor agonist

2267 1.4 cp BRD-K04430056 7-nitroindazole nitric oxide synthase inhibitor

2257 1.41 cp BRD-K64610608 EMF-bca1-57 caspase inhibitor

2258 1.41 cp BRD-A56359832 zileuton Leukotriene inhibitor

2259 1.41 cp BRD-K28761384 zuclopenthixol Dopamine receptor antagonist

2260 1.41 cp BRD-A48720949 testosterone androgen receptor agonist

2261 1.41 cp BRD-A45140972 meclocycline Bacterial 30S ribosomal subunit inhibitor

2262 1.41 cp BRD-K68190965 GR-46611 Serotonin receptor agonist

2263 1.41 cp BRD-K44849676 capsazepine TRPV agonist

2264 1.41 cp BRD-K04210847 tamoxifen Estrogen receptor antagonist

2265 1.41 cp BRD-K06980535 promazine Dopamine receptor antagonist

2266 1.41 cp BRD-K64746805 MBCQ Phosphodiesterase inhibitor

2255 1.42 cp BRD-K83988098 alvespimycin HSP inhibitor

2243 1.44 cp BRD-K99964838 bosutinib ABL inhibitor

2244 1.44 cp BRD-K83508485 FK-888 Tachykinin antagonist

2245 1.44 cp BRD-A83431637 resmethrin Cytochrome P450 inhibitor

2236 1.45 cp BRD-K44094599 tacrolimus Calcineurin inhibitor

2235 1.46 cp BRD-A77722753 hydralazine Vasodilator

2229 1.48 cp BRD-K13566078 BMS-345541 IKK inhibitor

2230 1.48 cp BRD-K16233984 eriochrome-black-t Azo dye

2231 1.48 cp BRD-K23875128 RHO-kinase-inhibitor-III[rockout] Rho associated kinase inhibitor

2221 1.52 cp BRD-K11696279 BU-239 Imidazoline receptor agonist

2222 1.52 cp BRD-A92826379 lupanine Sodium channel blocker

2223 1.52 cp BRD-A19195498 trimipramine Norepinephrine reuptake inhibitor

2210 1.55 cp BRD-K04010869 prostaglandin-a1 HSP inducer

2211 1.55 cp BRD-A07875874 cilnidipine Calcium channel blocker

2201 1.59 cp BRD-K13087974 "4,5-dianilinophthalimide" EGFR inhibitor

2202 1.59 cp BRD-A28318179 aminomethyltransferase Nitric oxide synthase inhibitor

2203 1.59 cp BRD-A54845972 dihydroergotamine Serotonin receptor agonist

2205 1.59 cp BRD-K41410256 balsalazide Cyclooxygenase inhibitor

2199 1.61 cp BRD-K70358946 aripiprazole Serotonin receptor agonist

2196 1.62 cp BRD-K62012036 acitretin Retinoid receptor agonist

2197 1.62 cp BRD-K52662033 lidocaine Histamine receptor agonist

2198 1.62 cp BRD-K76304753 phenazopyridine Local anesthetic

2188 1.63 cp BRD-A55946879 BW-B70C Lipoxygenase inhibitor

2185 1.66 cp BRD-K64835161 BRD-K64835161 -666

2186 1.66 cp BRD-A51714012 venlafaxine Adrenergic inhibitor

2179 1.69 cp BRD-K14441456 tyrphostin-AG-556 EGFR inhibitor

2168 1.73 cp BRD-K34508425 KUC103898N -666

2169 1.73 cp BRD-K91336023 mesulergine Dopamine receptor agonist

2170 1.73 cp BRD-K02265150 amoxapine Norepinephrine reuptake inhibitor

2171 1.73 cp BRD-K50128260 sildenafil Phosphodiesterase inhibitor

2164 1.75 cp BRD-M47937986 cefatrizine Bacterial cell wall synthesis inhibitor

2159 1.76 cp BRD-K68246049 TTNPB Retinoid receptor agonist

2163 1.76 cp BRD-K61269089 daphnetin Protein kinase inhibitor

2154 1.77 cp BRD-K85133207 HDAC1-selective HDAC inhibitor

2139 1.83 cp BRD-A24191444 ifenprodil Adrenergic receptor antagonist

2140 1.83 cp BRD-A79903587 tegafur Thymidylate synthase inhibitor

2141 1.83 cp BRD-K92138166 mammea-a other antibiotic

2142 1.83 cp BRD-A00993607 alprenolol Adrenergic receptor antagonist

2127 1.87 cp BRD-U08759356 EI-346-erlotinib-analog EGFR inhibitor

2128 1.87 cp BRD-K04993501 cefixime Bacterial cell wall synthesis inhibitor

2129 1.87 cp BRD-K06792661 narciclasine Coflilin signaling pathway activator

2130 1.87 cp BRD-K53318339 vinpocetine Phosphodiesterase inhibitor

2131 1.87 cp BRD-A30655177 LFM-A13 BTK inhibitor

2132 1.87 cp BRD-K09497549 kawain Calcium channel modulator

2125 1.89 cp BRD-A95869247 indapamide Thiazide diuretic

2119 1.9 cp BRD-A71009679 KUC103420N -666

2123 1.9 cp BRD-K87226815 cycloserine Bacterial cell wall synthesis inhibitor

2115 1.91 cp BRD-A64227845 SKF-77434 Dopamine receptor agonist

2108 1.97 cp BRD-K77947974 fluspirilene Dopamine receptor antagonist

2111 1.97 cp BRD-K93034159 cladribine Adenosine deaminase inhibitor

2106 1.98 cp BRD-K28360340 TW-37 BCL inhibitor

2096 2.01 cp BRD-K67174588 toremifene Estrogen receptor antagonist

2097 2.01 cp BRD-K65331431 retinyl vitamin analog

2098 2.01 cp BRD-K08996725 zolantidine Histamine receptor antagonist

2099 2.01 cp BRD-K81876028 CP-93129 Serotonin receptor agonist

2101 2.01 cp BRD-K68407802 KIN001-055 EGFR inhibitor

2102 2.01 cp BRD-K21680192 mitoxantrone Topoisomerase inhibitor

2093 2.03 cp BRD-A92670106 tocainide Sodium channel blocker

2083 2.04 cp BRD-K00184207 GR-206 Aryl hydrocarbon receptor ligand

2084 2.04 cp BRD-A25569250 KI-16425 Lysophosphatidic acid receptor antagonist

2085 2.04 cp BRD-K18757346 U-46619 Thromboxane receptor agonist

2086 2.04 cp BRD-A22707317 SB-205384 GABA receptor modulator

2087 2.04 cp BRD-K08998509 fananserin Dopamine receptor antagonist

2088 2.04 cp BRD-K55677650 CO-101244 Ionotropic glutamate receptor antagonist

2075 2.08 cp BRD-K41567364 SB-334867 Orexin receptor antagonist

2076 2.08 cp BRD-K84709232 caffeic-acid Lipoxygenase inhibitor

2078 2.08 cp BRD-K92049597 triamterene Sodium channel blocker

2062 2.11 cp BRD-K92413528 thiazolidinecarboxylic-acid Reducing agent

2063 2.11 cp BRD-K31699485 DMEOB glutamate receptor modulator

2064 2.11 cp BRD-A93000692 ciglitazone PPAR receptor agonist

2065 2.11 cp BRD-K60770992 pergolide Dopamine receptor agonist

2066 2.11 cp BRD-K53545112 CNQX Glutamate receptor antagonist

2055 2.15 cp BRD-K78692225 leflunomide Dihydroorotate dehydrogenase inhibitor

2056 2.15 cp BRD-K59256312 gabexate Serine protease inhibitor

2052 2.17 cp BRD-K16621777 enobosarm Androgen receptor modulator

2046 2.18 cp BRD-K97365803 PI-828 PI3K inhibitor

2047 2.18 cp BRD-K04546108 JAK3-inhibitor-VI JAK inhibitor

2050 2.18 cp BRD-K50387473 XMD-892 MAP kinase inhibitor

2032 2.22 cp BRD-K68558722 deracoxib Cyclooxygenase inhibitor

2033 2.22 cp BRD-K72093121 vidarabine Antiviral

2028 2.25 cp BRD-K18678457 ZD-7288 HCN channel blocker

2026 2.26 cp BRD-K42098891 protriptyline Tricyclic antidepressant

2025 2.27 cp BRD-K57080016 selumetinib MEK inhibitor

2015 2.29 cp BRD-K49810818 sorafenib FLT3 inhibitor

2016 2.29 cp BRD-K35629949 SR-27897 CCK receptor antagonist

2017 2.29 cp BRD-K15196155 IBC-293 Hydroxycarboxylic acid receptor agonist

2018 2.29 cp BRD-A06390036 hydroquinidine Antiarrhythmic

2019 2.29 cp BRD-A02189320 met-leu-phe -666

2021 2.29 cp BRD-K47679368 bromfenac Cyclooxygenase inhibitor

2004 2.33 cp BRD-K09255212 clioquinol Chelating agent

2005 2.33 cp BRD-K37194137 III606050 Cytochrome P450 inhibitor

1991 2.38 cp BRD-K70505054 ranitidine Histamine receptor antagonist

1992 2.38 cp BRD-K09635134 l-erythro-MAPP negative control for D-erythro-MAPP

1988 2.4 cp BRD-K33453211 levocabastine Histamine receptor antagonist

1983 2.43 cp BRD-A58564983 selamectin Nematocide

1976 2.47 cp BRD-K84996949 sinensetin Cyclooxygenase inhibitor

1977 2.47 cp BRD-A51393488 noscapine Bradykinin receptor antagonist

1970 2.5 cp BRD-K16444452 ibudilast Leukotriene receptor antagonist

1971 2.5 cp BRD-K52989797 clomipramine Serotonin transporter inhibitor (SERT)

1968 2.51 cp BRD-K41445866 alfaxalone Chloride channel agonist

1963 2.53 cp BRD-K43236057 piceid ICAM1 inhibitor

1964 2.53 cp BRD-K85242180 beta-CCP "Indoleamine 2,3-dioxygenase inhibitor"

1961 2.54 cp BRD-K06234293 LY-364947 TGF beta receptor inhibitor

1962 2.54 cp BRD-K04185004 oxybuprocaine Local anesthetic

1955 2.56 cp BRD-K74212935 ergocryptine Dopamine agonist

1953 2.57 cp BRD-K49404994 levetiracetam Calcium channel blocker

1954 2.57 cp BRD-K12867552 THM-I-94 HDAC inhibitor

1947 2.58 cp BRD-K17796732 JWH-015 Cannabinoid receptor agonist

1941 2.6 cp BRD-K07762753 aminopurvalanol-a Tyrosine kinase inhibitor

1938 2.61 cp BRD-A51410489 yohimbine Adrenergic receptor antagonist

1939 2.61 cp BRD-K76064317 tyrphostin-AG-1296 FLT3 inhibitor

1934 2.64 cp BRD-K30189597 SYK-inhibitor SYK inhibitor

1929 2.67 cp BRD-K97158071 droperidol Dopamine receptor antagonist

1926 2.68 cp BRD-A17448384 beclometasone Glucocorticoid receptor agonist

1927 2.68 cp BRD-A80641450 FR-139317 Endothelin receptor antagonist

1920 2.71 cp BRD-K26664453 cytochalasin-b Microtubule inhibitor

1916 2.72 cp BRD-K44876623 zolpidem Benzodiazepine receptor agonist

1913 2.73 cp BRD-A13807286 HA-14-1 BCL inhibitor

1906 2.75 cp BRD-A73605923 mocimycin Protein synthesis inhibitor

1907 2.75 cp BRD-K15025317 BAY-11-7821 NFkB pathway inhibitor

1908 2.75 cp BRD-K93645900 tadalafil Phosphodiesterase inhibitor

1904 2.77 cp BRD-K15935639 z-leu3-VS Proteasome inhibitor

1902 2.78 cp BRD-A33711280 metixene Acetylcholine receptor antagonist

1894 2.82 cp BRD-A22256192 terazosin Adrenergic receptor antagonist

1895 2.82 cp BRD-K14807180 SB-221284 Serotonin receptor antagonist

1889 2.84 cp BRD-K83063356 RS-102895 CCR antagonist

1888 2.85 cp BRD-K85985071 ellipticine Topoisomerase inhibitor

1885 2.87 cp BRD-K18194590 mephentermine Adrenergic receptor agonist

1879 2.89 cp BRD-K78633253 EXO-1 ARF inhibitor

1880 2.89 cp BRD-K99946902 hexylresorcinol Local anesthetic

1875 2.93 cp BRD-K33211335 dextromethorphan Glutamate receptor antagonist

1870 2.96 cp BRD-A38898897 GW-311616 Leukocyte elastase inhibitor

1871 2.96 cp BRD-K16508793 diazepam Benzodiazepine receptor agonist

1872 2.96 cp BRD-K05396879 15-delta-prostaglandin-j2 PPAR receptor agonist

1853 3.07 cp BRD-K24859147 KIN001-242 Protein kinase inhibitor

1854 3.07 cp BRD-K73290745 ICI-199441 Opioid receptor agonist

1849 3.09 cp BRD-K42748308 XE-991 Potassium channel blocker

1845 3.1 cp BRD-A20131130 "2',5'-dideoxyadenosine" Adenylyl cyclase inhibitor

1847 3.1 cp BRD-K67261995 adipiodone Contrast agent

1848 3.1 cp BRD-K11129031 gemfibrozil Lipoprotein lipase activator

1844 3.11 cp BRD-A18763547 BAX-channel-blocker Cytochrome C release inhibitor

1836 3.14 cp BRD-K24576554 AT-9283 JAK inhibitor

1837 3.14 cp BRD-K64670467 JNJ-16259685 Glutamate receptor antagonist

1832 3.17 cp BRD-A36318220 necrostatin-1 RIPK inhibitor

1823 3.21 cp BRD-K52080565 rilmenidine Imidazoline receptor agonist

1824 3.21 cp BRD-K50836978 purvalanol-a CDK inhibitor

1819 3.25 cp BRD-A56675431 altizide Thiazide diuretic

1813 3.27 cp BRD-A19736161 ondansetron Serotonin receptor antagonist

1811 3.29 cp BRD-K40645748 mefloquine Adenosine receptor antagonist

1807 3.3 cp BRD-A37347161 BRL-52537 Opioid receptor agonist

1805 3.31 cp BRD-K62221994 T-98475 Gonadotropin releasing factor hormone receptor antagonist

1802 3.32 cp BRD-K09631521 thiotepa Cytochrome P450 inhibitor

1795 3.35 cp BRD-A32164164 methyllycaconitine Acetylcholine receptor antagonist

1790 3.37 cp BRD-A55913614 primaquine Antimalarial

1788 3.38 cp BRD-A06784547 MRS-1334 Adenosine receptor antagonist

1777 3.45 cp BRD-K70401845 erlotinib EGFR inhibitor

1778 3.45 cp BRD-K39339537 epirizole Cyclooxygenase inhibitor

1780 3.45 cp BRD-K43887077 dopamine Dopamine receptor agonist

1766 3.56 cp BRD-A29289453 PCA-4248 Platelet activating factor receptor antagonist

1767 3.56 cp BRD-K09537769 NU-7026 DNA dependent protein kinase inhibitor

1768 3.56 cp BRD-K41713976 E-4031 Potassium channel blocker

1765 3.57 cp BRD-K71799949 carbamazepine Carboxamide antiepileptic

1763 3.59 cp BRD-U94846492 quinine Hemozoin biocrystallization inhibitor

1756 3.63 cp BRD-K32906660 bis-tyrphostin EGFR inhibitor

1757 3.63 cp BRD-A43671941 oxprenolol Adrenergic receptor antagonist

1752 3.67 cp BRD-K19540840 saracatinib SRC inhibitor

1747 3.72 cp BRD-A35108200 dexamethasone Glucocorticoid receptor agonist

1743 3.75 cp BRD-K77987382 mebendazole Tubulin inhibitor

1735 3.77 cp BRD-K37447567 hydrocotarnine Opioid receptor antagonist

1734 3.78 cp BRD-K76810206 nicergoline Adrenergic receptor antagonist

1725 3.84 cp BRD-K99451608 lopinavir HIV protease inhibitor

1726 3.84 cp BRD-K72783841 tyrphostin-AG-555 EGFR inhibitor

1727 3.84 cp BRD-K96119599 leucodin Melanin inhibitor

1729 3.84 cp BRD-A37776212 ICI-204448 Opioid receptor agonist

1723 3.85 cp BRD-K17743697 KB-R7943 Sodium/calcium exchange inhibitor

1718 3.91 cp BRD-K71731651 PNU-120596 Acetylcholine receptor agonist

1719 3.91 cp BRD-K82484965 carmoxirole Dopamine receptor agonist

1715 3.95 cp BRD-K07403598 CAY-10470 NFkB pathway inhibitor

1714 3.96 cp BRD-K41564320 purvalanol-b Tyrosine kinase inhibitor

1712 3.97 cp BRD-K54314721 zolmitriptan Serotonin receptor agonist

1710 3.99 cp BRD-K28428262 brivanib FGFR inhibitor

1708 4.01 cp BRD-K68488863 ENMD-2076 FLT3 inhibitor

1705 4.02 cp BRD-K01649396 indatraline Norepinephrine transporter inhibitor

1707 4.02 cp BRD-K97056771 HY-11007 BCR-ABL kinase inhibitor

1700 4.05 cp BRD-K89626439 sirolimus MTOR inhibitor

1693 4.09 cp BRD-A73368467 fexofenadine Histamine receptor antagonist

1689 4.11 cp BRD-K32398298 alprazolam Benzodiazepine receptor agonist

1682 4.16 cp BRD-K25079130 avrainvillamide-analog-4 nucleophosmin inhibitor

1683 4.16 cp BRD-K29582115 ziprasidone Dopamine receptor antagonist

1684 4.16 cp BRD-K32610195 androstenedione Cytochrome P450 inhibitor

1685 4.16 cp BRD-K72029282 probucol Atherogenesis inhibitor

1676 4.19 cp BRD-K55420858 mirin MRE11A exonuclease inhibitor

1674 4.22 cp BRD-K25504083 cytochalasin-d Actin polymerization inhibitor

1673 4.26 cp BRD-A13122391 triptolide RNA polymerase inhibitor

1671 4.28 cp BRD-A39268308 epibatidine Acetylcholine receptor agonist

1666 4.3 cp BRD-K42573370 avrainvillamide-analog-2 nucleophosmin inhibitor

1667 4.3 cp BRD-K82357231 desloratadine Histamine receptor antagonist

1668 4.3 cp BRD-A65013509 oxybutynin Acetylcholine receptor antagonist

1657 4.37 cp BRD-K62810658 PD-98059 MEK inhibitor

1653 4.4 cp BRD-A19500257 geldanamycin HSP inhibitor

1649 4.43 cp BRD-K56115039 BU-226 Imidazoline receptor ligand

1647 4.44 cp BRD-K92984783 melperone Serotonin receptor antagonist

1644 4.45 cp BRD-K63828191 raloxifene Estrogen receptor antagonist

1634 4.51 cp BRD-K83963101 MLN-8054 Aurora kinase inhibitor

1631 4.54 cp BRD-A04352665 maraviroc CC chemokine receptor antagonist

1632 4.54 cp BRD-K09859624 methantheline Acetylcholine receptor antagonist

1629 4.58 cp BRD-A30435184 metergoline Dopamine receptor agonist

1619 4.64 cp BRD-K94144010 cotinine Nicotine metabolite

1620 4.64 cp BRD-K59962020 CHEMBL-374350 NFkB pathway inhibitor

1618 4.65 cp BRD-K04710043 hexamethylenebisacetamide AKT inhibitor

1617 4.67 cp BRD-K97118047 "4,5,6,7-tetrabromobenzotriazole" Casein kinase inhibitor

1614 4.69 cp BRD-K35458079 edaravone Nootropic agent

1608 4.74 cp BRD-K83322645 L-693403 Sigma receptor agonist

1605 4.76 cp BRD-K13926615 vardenafil Phosphodiesterase inhibitor

1604 4.77 cp BRD-A52660433 tetrindole Monoamine oxidase inhibitor

1598 4.83 cp BRD-K15600710 obatoclax BCL inhibitor

1594 4.84 cp BRD-A04756508 norgestimate Progesterone receptor agonist

1590 4.86 cp BRD-A41555725 chlortetracycline Protein synthesis inhibitor

1587 4.87 cp BRD-A84493640 atovaquone Mitochondrial electron transport inhibitor

1589 4.87 cp BRD-K50140147 NVP-TAE684 ALK inhibitor

1579 4.92 cp BRD-K40782193 QX-222 Sodium channel blocker

1570 5 cp BRD-K86003836 flubendazole Tubulin inhibitor

1571 5 cp BRD-K28137194 loreclezole GABA receptor agonist

1567 5.05 cp BRD-K83010055 VU-0415374-1 Glutamate receptor modulator

1564 5.07 cp BRD-K14791739 fluticasone Glucocorticoid receptor agonist

1563 5.09 cp BRD-K46556543 canrenoic-acid Mineralocorticoid receptor antagonist

1562 5.1 cp BRD-K79425933 benperidol Dopamine receptor antagonist

1558 5.13 cp BRD-K22096725 ALW-II-49-7 Ephrin inhibitor

1552 5.21 cp BRD-K56047318 RHC-80267 Triacylglycerol lipase inhibitor

1546 5.32 cp BRD-K36009368 NNC-63-0532 Opioid receptor agonist

1536 5.48 cp BRD-K57718010 pentylenetetrazol GABA receptor antagonist

1537 5.48 cp BRD-A69917777 aminopentamide Acetylcholine receptor antagonist

1535 5.5 cp BRD-K12219985 glipizide Sulfonylurea

1530 5.57 cp BRD-K91243525 SR-142948 Neurotensin receptor antagonist

1524 5.7 cp BRD-A25143711 hydrocortisone Glucocorticoid receptor agonist

1523 5.71 cp BRD-K45401373 betulinic-acid Apoptosis stimulant

1519 5.78 cp BRD-A96107863 nisoldipine Calcium channel blocker

1510 5.87 cp BRD-K41868777 W-5 Calmodulin antagonist

1509 5.88 cp BRD-K88868628 iodoacetic-acid Cysteine peptidase inhibitor

1508 5.89 cp BRD-A77291778 cyclopentolate Acetylcholine receptor antagonist

1500 5.95 cp BRD-K15086322 JNJ-10191584 Histamine receptor antagonist

1496 6.01 cp BRD-A41450521 tosufloxacin Bacterial DNA gyrase inhibitor

1490 6.05 cp BRD-K67537649 PQ-401 IGF-1 inhibitor

1487 6.1 cp BRD-K11636097 JNJ-7706621 CDK inhibitor

1484 6.13 cp BRD-K39965020 doconexent PPAR receptor agonist

1483 6.15 cp BRD-K61341215 vecuronium Acetylcholine receptor antagonist

1481 6.17 cp BRD-A64479082 quinidine Sodium channel blocker

1474 6.24 cp BRD-K08273968 griseofulvin Tubulin inhibitor

1471 6.31 cp BRD-K19220233 JNK-9L JNK inhibitor

1470 6.32 cp BRD-A44090213 indoprofen Cyclooxygenase inhibitor

1466 6.37 cp BRD-K77175907 calcifediol Vitamin D receptor agonist

1464 6.39 cp BRD-K60690191 MPEP Glutamate receptor antagonist

1461 6.44 cp BRD-K80725821 RS-16566 Serotonin receptor antagonist

1460 6.48 cp BRD-K81272440 dantrolene Calcium channel blocker

1455 6.54 cp BRD-A02759312 betaxolol Adrenergic receptor antagonist

1456 6.54 cp BRD-A97437073 rosiglitazone Insulin sensitizer

1450 6.58 cp BRD-K54704028 BAY-36-7620 Glutamate receptor antagonist

1447 6.61 cp BRD-A54927599 KF-38789 P-selectin inhibitor

1448 6.61 cp BRD-K69328504 L-690488 Inositol monophosphatase inhibitor

1441 6.69 cp BRD-K92093830 doxorubicin Topoisomerase inhibitor

1440 6.7 cp BRD-K51302260 KU-C103871 GSP agonist

1438 6.73 cp BRD-K10995081 perphenazine Dopamine receptor antagonist

1433 6.81 cp BRD-K09132007 D-4476 TGF beta receptor inhibitor

1431 6.87 cp BRD-K62736196 guanabenz -666

1430 6.94 cp BRD-A10070317 propranolol Adrenergic receptor antagonist

1428 7.01 cp BRD-K93480852 KN-93 Calcium-calmodulin dependent protein kinase inhibitor

1426 7.08 cp BRD-K84141129 VU-0400193-3 Glutamate receptor modulator

1422 7.14 cp BRD-K99922388 DPO-1 Potassium channel blocker

1419 7.16 cp BRD-K28470988 L-690330 Inositol monophosphatase inhibitor

1420 7.16 cp BRD-A67373739 AICA-ribonucleotide AMPK activator

1415 7.22 cp BRD-K81847782 scandenin Plant compound with antimicrobial activity

1409 7.27 cp BRD-K76698671 HNHA HDAC inhibitor

1410 7.27 cp BRD-A35519318 benidipine Calcium channel blocker

1411 7.27 cp BRD-A65671304 candesartan Angiotensin receptor antagonist

1404 7.33 cp BRD-K05658747 raltegravir HIV integrase inhibitor

1403 7.34 cp BRD-A25234499 aminoglutethimide Glucocorticoid receptor antagonist

1400 7.35 cp BRD-K45158365 valsartan Angiotensin receptor antagonist

1399 7.4 cp BRD-A91555231 norepinephrine Adrenergic receptor agonist

1398 7.42 cp BRD-A74667430 etodolac Cyclooxygenase inhibitor

1395 7.48 cp BRD-K66175015 afatinib EGFR inhibitor

1390 7.54 cp BRD-A04327189 synephrine Adrenergic receptor agonist

1389 7.56 cp BRD-K06817181 BRD-K06817181 JAK inhibitor

1383 7.64 cp BRD-K59037100 oxybenzone Lipase inhibitor

1380 7.67 cp BRD-K44779798 miglitol Glucosidase inhibitor

1375 7.71 cp BRD-A65449987 flunisolide Cytochrome P450 inhibitor

1376 7.71 cp BRD-K79353516 indolophenanthridine CALY activator

1370 7.79 cp BRD-A75478957 PD-166793 Metalloproteinase inhibitor

1371 7.79 cp BRD-K20742498 RS-39604 Serotonin receptor antagonist

1367 7.82 cp BRD-K54529596 captopril ACE inhibitor

1365 7.84 cp BRD-K78637815 LY-320135 Cannabinoid receptor antagonist

1362 7.89 cp BRD-A80383043 BRD-A80383043 Glutamate receptor agonist

1359 7.93 cp BRD-K43389675 daunorubicin RNA synthesis inhibitor

1358 7.99 cp BRD-K18518344 digitoxigenin ATPase inhibitor

1356 8.11 cp BRD-A83695761 chromanol Potassium channel blocker

1353 8.23 cp BRD-A13133631 fluorometholone Glucocorticoid receptor agonist

1350 8.28 cp BRD-A36707673 hydroxycholesterol LXR agonist

1349 8.31 cp BRD-K64755930 etazolate Phosphodiesterase inhibitor

1346 8.33 cp BRD-K99029477 prometon Photosynthesis inhibitor

1343 8.34 cp BRD-K52850071 JAK3-Inhibitor-II JAK inhibitor

1341 8.35 cp BRD-K40758068 efavirenz HIV protease inhibitor

1337 8.42 cp BRD-M30523314 vinorelbine Tubulin inhibitor

1338 8.42 cp BRD-K37618799 MRS-1220 Adenosine receptor antagonist

1335 8.46 cp BRD-K87909389 alvocidib CDK inhibitor

1328 8.54 cp BRD-K95785537 PP-2 SRC inhibitor

1329 8.54 cp BRD-M64432851 sunitinib FLT3 inhibitor

1326 8.62 cp BRD-K44497846 enalapril ACE inhibitor

1316 8.75 cp BRD-K79684402 RO-10-5824 Dopamine receptor agonist

1317 8.75 cp BRD-K26117720 gingerol Nitric oxide synthase inhibitor

1314 8.8 cp BRD-K74913225 brinzolamide Carbonic anhydrase inhibitor

1313 8.83 cp BRD-A42831637 tetrahydrocannabinol-7-oic-acid Anti-inflammatory

1309 8.92 cp BRD-K15502390 nevirapine Reverse transcriptase inhibitor

1304 8.97 cp BRD-K82846253 repaglinide Insulin secretagogue

1303 9.01 cp BRD-A80793822 pemoline Dopamine receptor agonist

1301 9.02 cp BRD-A96456596 FPL-55712 Leukotriene receptor antagonist

1300 9.04 cp BRD-K17140735 SCH-79797 Proteasome inhibitor

1298 9.06 cp BRD-K53665955 MK-5108 Aurora kinase inhibitor

1295 9.13 cp BRD-A14966924 alaproclate Serotonin receptor antagonist

1293 9.19 cp BRD-K02607075 tubocurarine Acetylcholine receptor antagonist

1291 9.29 cp BRD-K73293050 WZ-3146 EGFR inhibitor

1290 9.3 cp BRD-K36377456 marmesin Angiogenesis inhibitor

1284 9.38 cp BRD-K94830329 ataluren CFTR channel agonist

1280 9.43 cp BRD-K49657628 tyrphostin-AG-18 EGFR inhibitor

1277 9.47 cp BRD-K38323065 phenacetin Cyclooxygenase inhibitor

1272 9.55 cp BRD-K33483813 actarit Interleukin receptor agonist

1270 9.57 cp BRD-K55703048 latrepirdine Glutamate receptor antagonist

1269 9.59 cp BRD-A58955223 sulforaphane Antineoplastic

1263 9.68 cp BRD-K63151507 MNITMT Lymphocyte inhibitor

1262 9.69 cp BRD-K43164539 cholic-acid Bile acid

1261 9.71 cp BRD-A20589515 dihydroxyphenylglycine Glutamate receptor agonist

1258 9.73 cp BRD-K39111395 BCL2-inhibitor BCL inhibitor

1256 9.74 cp BRD-K62374253 rufloxacin Bacterial DNA gyrase inhibitor

1257 9.74 cp BRD-K12002134 megestrol progesterone receptor agonist

1250 9.88 cp BRD-A41995253 brucine Glycine receptor antagonist

1248 9.91 cp BRD-K21565985 xylazine Adrenergic receptor agonist

1249 9.91 cp BRD-K17075857 chloroxine Opioid receptor antagonist

1245 9.95 cp BRD-K03109492 NSC-663284 CDC inhibitor

1244 9.98 cp BRD-K31627533 rimexolone Glucocorticoid receptor agonist

1243 10 cp BRD-A47144777 dihydro-7-desacetyldeoxygedunin HSP inhibitor

1242 10.02 cp BRD-K39670393 amthamine Histamine receptor agonist

1240 10.06 cp BRD-K59753853 MDL-29951 Glutamate receptor antagonist

1241 10.06 cp BRD-K72726508 arcyriaflavin-a CDK inhibitor

1236 10.22 cp BRD-A85860691 chaetocin Histone lysine methyltransferase inhibitor

1234 10.24 cp BRD-K01612348 meropenem Bacterial cell wall synthesis inhibitor

1232 10.26 cp BRD-K87573634 propylpyrazole Estrogen receptor agonist

1231 10.27 cp BRD-K55191674 benzylpenicillin Penicillin binding protein inhibitor

1228 10.31 cp BRD-K57926513 tyrphostin-AG-1295 PDGFR receptor inhibitor

1229 10.31 cp BRD-K81521265 dicyclohexylurea Epoxide hydolase inhibitor

1226 10.32 cp BRD-K63784565 BRD-K63784565 Topoisomerase inhibitor

1218 10.41 cp BRD-K42679050 Y-27152 Potassium channel activator

1219 10.41 cp BRD-A09094913 strychnine Acetylcholine receptor antagonist

1212 10.52 cp BRD-K48300629 zonisamide Sodium channel blocker

1210 10.56 cp BRD-K13169950 NSC-3852 HDAC inhibitor

1209 10.59 cp BRD-A25687296 emetine Protein synthesis inhibitor

1207 10.62 cp BRD-K61097567 SB-218795 Tachykinin antagonist

1200 10.77 cp BRD-K41859756 NVP-AUY922 HSP inhibitor

1199 10.81 cp BRD-K00610438 altanserin Serotonin receptor antagonist

1197 10.82 cp BRD-K10467831 tibolone Androgen receptor agonist

1198 10.82 cp BRD-K33193182 methylnorlichexanthone Aurora kinase inhibitor

1195 10.85 cp BRD-K11630072 carmofur Thymidylate synthase inhibitor

1190 10.89 cp BRD-K95921201 reserpine Vesicular monoamine transporter inhibitor

1189 10.98 cp BRD-K12502280 TG-101348 FLT3 inhibitor

1186 11.05 cp BRD-K17868609 BRL-54443 Serotonin receptor agonist

1187 11.05 cp BRD-A90131694 alclometasone Glucocorticoid receptor agonist

1185 11.06 cp BRD-K81169441 cerivastatin HMGCR inhibitor

1173 11.4 cp BRD-K62609077 scoulerine Adrenergic receptor antagonist

1171 11.41 cp BRD-K83636919 entacapone Catechol O methyltransferase inhibitor

1172 11.41 cp BRD-K74236984 UNC-0321 Histone lysine methyltransferase inhibitor

1165 11.57 cp BRD-K29530284 amlexanox Histamine receptor modulator

1161 11.69 cp BRD-K03981224 ethisterone Progestogen hormone

1160 11.7 cp BRD-A75368507 demeclocycline Bacterial 30S ribosomal subunit inhibitor

1157 11.71 cp BRD-K24675965 LY-288513 CCK receptor antagonist

1154 11.73 cp BRD-K28296557 AKT-inhibitor-IV AKT inhibitor

1155 11.73 cp BRD-K72222507 quinapril ACE inhibitor

1152 11.78 cp BRD-K47192521 icosapent Platelet aggregation inhibitor

1149 11.84 cp BRD-K26818574 BIX-01294 Histone lysine methyltransferase inhibitor

1142 11.93 cp BRD-K51018020 VAMA-37 DNA dependent protein kinase inhibitor

1143 11.93 cp BRD-A83326220 brazilin Nitric oxide production inhibitor

1141 11.95 cp BRD-K55344148 BU-224 Imidazoline receptor ligand

1138 12.02 cp BRD-K91263825 nortriptyline Tricyclic antidepressant

1135 12.12 cp BRD-A95696820 acadesine AMPK activator

1134 12.15 cp BRD-K67847053 guanabenz Adrenergic receptor agonist

1132 12.16 cp BRD-K79092138 nitrofural Bacterial DNA inhibitor

1131 12.17 cp BRD-A70268693 PG-9 Acetylcholine receptor agonist

1130 12.21 cp BRD-A50675702 fipronil GABA gated chloride channel blocker

1129 12.25 cp BRD-K15592317 CP466722 ATM kinase inhibitor

1128 12.26 cp BRD-K14618467 IKK-16 IKK inhibitor

1124 12.33 cp BRD-K50464341 berbamine Calmodulin antagonist

1121 12.4 cp BRD-K15588452 R-96544 Serotonin receptor antagonist

1120 12.41 cp BRD-K26241953 piceatannol SYK inhibitor

1119 12.42 cp BRD-K14880289 GW-501516 PPAR receptor agonist

1118 12.43 cp BRD-K66707493 lawsone Coloring agent

1116 12.44 cp BRD-K64514229 toltrazuril Antiprotozoal

1112 12.51 cp BRD-K06543683 bisindolylmaleimide-ix CDK inhibitor

1110 12.64 cp BRD-K88741031 "methyl-2,5-dihydroxycinnamate" EGFR inhibitor

1107 12.75 cp BRD-K34330170 rotenonic-acid Retinoid receptor antagonist

1106 12.76 cp BRD-A62434282 goserelin Gonadotropin releasing factor hormone receptor agonist

1105 12.77 cp BRD-K52020312 metronidazole DNA inhibitor

1103 12.78 cp BRD-K01638814 rilmenidine Adrenergic receptor agonist

1102 12.8 cp BRD-A43849199 karakoline Phytotoxin

1100 12.85 cp BRD-K08287586 butylparaben DNA synthesis inhibitor

1099 12.91 cp BRD-K70557564 zosuquidar P-glycoprotein inhibitor

1095 12.97 cp BRD-K88701661 dimercaptosuccinic-acid Chelating agent

1096 12.97 cp BRD-A69960130 bromocriptine Dopamine receptor agonist

1093 13.04 cp BRD-A73909368 dactinomycin RNA polymerase inhibitor

1090 13.08 cp BRD-K05104363 PD-184352 MEK inhibitor

1091 13.08 cp BRD-A35033682 eriodictyol Cytochrome P450 inhibitor

1086 13.16 cp BRD-K67844266 MLN-4924 Nedd activating enzyme inhibitor

1084 13.18 cp BRD-K18036262 L-168049 Glucagon receptor antagonist

1082 13.21 cp BRD-K07888196 tyrphostin-AG-538 IGF-1 inhibitor

1076 13.4 cp BRD-K02715688 hydrastine Tyrosine hydroxylase inhibitor

1074 13.44 cp BRD-K24201553 SB-269970 Serotonin receptor antagonist

1063 13.68 cp BRD-K03440695 boldine Acetylcholine receptor antagonist

1060 13.77 cp BRD-K41895714 AS-605240 PI3K inhibitor

1052 14.09 cp BRD-K89152108 liothyronine Thyroid hormone stimulant

1049 14.12 cp BRD-K17705806 JTC-801 Opioid receptor antagonist

1046 14.16 cp BRD-A83237092 fulvestrant Estrogen receptor antagonist

1045 14.17 cp BRD-K96809896 SKF-86002 p38 MAPK inhibitor

1044 14.22 cp BRD-K76587808 fraxetin Antioxidant

1043 14.23 cp BRD-K71512533 SNS-314 Aurora kinase inhibitor

1041 14.25 cp BRD-K51816706 oxindole-I VEGFR inhibitor

1037 14.28 cp BRD-K38003476 clocortolone Glucocorticoid receptor agonist

1038 14.28 cp BRD-K52459643 prostaglandin-e1 Prostanoid receptor agonist

1030 14.46 cp BRD-K77133231 PD-169316 p38 MAPK inhibitor

1027 14.6 cp BRD-A89434049 sarmentogenin ATPase inhibitor

1025 14.74 cp BRD-K12539581 nocodazole Tubulin inhibitor

1023 14.76 cp BRD-K14693417 cinchonine P-glycoprotein inhibitor

1016 14.87 cp BRD-K19894101 MST-312 Telomerase inhibitor

1003 15.24 cp BRD-K36258877 AZ-10417808 Caspase inhibitor

1000 15.29 cp BRD-K08619838 tremorine Acetylcholine receptor agonist

991 15.48 cp BRD-K40892394 AR-C133057XX Nitric oxide synthase inhibitor

992 15.48 cp BRD-A49765801 fludroxycortide Glucocorticoid receptor agonist

989 15.56 cp BRD-K01624546 docosatrienoic-acid LTB4 inhibitor

988 15.62 cp BRD-K78122587 NNC-55-0396 T-type calcium channel blocker

987 15.64 cp BRD-K84566043 fenpiverinium Acetylcholine receptor antagonist

986 15.65 cp BRD-K22010301 JLK-6 Gamma secretase inhibitor

979 15.8 cp BRD-A18579359 wiskostatin Neural Wiskott-Aldrich syndrome protein inhibitor

978 15.83 cp BRD-K73991644 isoquercetin Aldose reductase inhibitor

977 15.84 cp BRD-K62310379 fluticasone Glucocorticoid receptor agonist

975 15.92 cp BRD-K82865713 prostaglandin-b2 cAMP inhibitor

976 15.92 cp BRD-A72703248 SKF-96365 Calcium channel blocker

974 15.97 cp BRD-K32164935 tolazamide ATP channel blocker

971 16.03 cp BRD-K65814004 diphenyleneiodonium Nitric oxide synthase inhibitor

969 16.12 cp BRD-K78485176 olmesartan Angiotensin receptor antagonist

968 16.16 cp BRD-K62289640 lylamine Cannabinoid receptor agonist

966 16.25 cp BRD-K73978287 hydrocortisone Glucocorticoid receptor agonist

964 16.28 cp BRD-K66093087 FGIN-1-43 Benzodiazepine receptor agonist

956 16.8 cp BRD-K11911061 GR-127935 Serotonin receptor antagonist

957 16.8 cp BRD-K60274257 dephostatin Tyrosine phosphatase inhibitor

954 16.85 cp BRD-K41903098 diphenoxylate Opioid receptor agonist

952 16.86 cp BRD-K27665173 D-64406 PDGFR receptor inhibitor

951 16.99 cp BRD-K37691127 hinokitiol Tyrosinase inhibitor

949 17.01 cp BRD-K59753975 vindesine Tubulin inhibitor

950 17.01 cp BRD-K96778649 tyrphostin-47 EGFR inhibitor

948 17.07 cp BRD-K48735772 PD-158780 EGFR inhibitor

947 17.08 cp BRD-K84175871 pseudoephedrine Adrenergic receptor agonist

943 17.23 cp BRD-A49906757 scopolamine Acetylcholine receptor antagonist

942 17.24 cp BRD-A28970875 puromycin Protein synthesis inhibitor

941 17.25 cp BRD-A09467419 mebeverine Acetylcholine receptor antagonist

935 17.4 cp BRD-A41112154 oleanolic-acid G protein-coupled receptor agonist

933 17.41 cp BRD-K95739795 tetrabenazine Vesicular monoamine transporter inhibitor

930 17.44 cp BRD-K59469039 AG-879 Angiogenesis inhibitor

931 17.44 cp BRD-K94353609 fluocinolone Glucocorticoid receptor agonist

929 17.45 cp BRD-K68132782 terbinafine Fungal squalene epoxidase inhibitor

928 17.53 cp BRD-K59058766 chlorprothixene Dopamine receptor antagonist

918 17.82 cp BRD-K28667196 fillalbin Increases arterial blood pressure

917 17.92 cp BRD-K64881305 ispinesib Kinesin-like spindle protein inhibitor

915 18.03 cp BRD-A20968261 WAY-213613 Glutamate inhibitor

909 18.36 cp BRD-K35377380 I-OMe-AG-538 IGF-1 inhibitor

908 18.51 cp BRD-K33572481 taurodeoxycholic-acid Bile acid

905 18.61 cp BRD-U73238814 QL-XI-92 DDR1 inhibitor

903 18.62 cp BRD-K88625236 nonoxynol-9 Membrane integrity inhibitor

902 18.65 cp BRD-K93441486 diphemanil Acetylcholine receptor antagonist

899 18.67 cp BRD-K50325075 UCL-2077 Slow after hyperpolarization channel blocker

900 18.67 cp BRD-K34995470 SU-1498 VEGFR inhibitor

897 18.69 cp BRD-K30570479 VU-0400195-3 Glutamate receptor modulator

895 18.76 cp BRD-K41160163 fenobam Glutamate receptor antagonist

891 18.82 cp BRD-K80431395 triciribine AKT inhibitor

887 18.84 cp BRD-A24429032 HEAT Adrenergic receptor antagonist

882 19.04 cp BRD-K59650319 YM-298198 Glutamate receptor antagonist

881 19.11 cp BRD-K35559145 levomepromazine Dopamine receptor antagonist

874 19.28 cp BRD-K70914287 BIBX-1382 EGFR inhibitor

872 19.44 cp BRD-K14681867 somatostatin Somatostatin receptor agonist

870 19.56 cp BRD-A71459254 cymarin ATPase inhibitor

866 19.74 cp BRD-K11558771 droxinostat HDAC inhibitor

864 19.82 cp BRD-K98521173 desoxycortone Mineralocorticoid receptor agonist

862 19.91 cp BRD-A08003242 rhodomyrtoxin-b sodium fluorescein uptake inhibitor

857 20.11 cp BRD-A68930007 ouabain ATPase inhibitor

854 20.26 cp BRD-A87125127 3-matida Glutamate receptor antagonist

853 20.42 cp BRD-K72676686 fluvoxamine Selective serotonin reuptake inhibitor (SSRI)

850 20.64 cp BRD-A25775766 securinine GABA receptor antagonist

842 20.88 cp BRD-A78360835 cercosporin Photoactivated toxin

840 21.02 cp BRD-K17415526 tyrphostin-AG-835 Protein tyrosine kinase inhibitor

833 21.33 cp BRD-A46186775 hydrocortisone Glucocorticoid receptor agonist

832 21.34 cp BRD-K49448285 bisindolylmaleimide CDK inhibitor

829 21.39 cp BRD-A19633847 perhexiline Carnitine palmitoyltransferase inhibitor

830 21.39 cp BRD-A35623999 CGP-37157 L-type calcium channel blocker

827 21.45 cp BRD-K87510569 RS-504393 CC chemokine receptor antagonist

824 21.6 cp BRD-A94413429 NTNCB Neuropeptide receptor antagonist

823 21.61 cp BRD-K71266197 PSB-06126 NTPDase inhibitor

818 21.79 cp BRD-K37798499 etoposide Topoisomerase inhibitor

817 21.8 cp BRD-K76840893 RS-17053 Adrenergic receptor antagonist

814 22 cp BRD-K32318651 acyclovir DNA polymerase inhibitor

812 22.13 cp BRD-A93477898 PETCM Caspase activator

809 22.17 cp BRD-K97863768 prothionamide Mycobacterium tuberculosis enoyl-[acyl-carrier-protein] reductase [NADH] (inhA) inhibitor

810 22.17 cp BRD-K65285700 BRD-K65285700 Cannabinoid receptor agonist

808 22.18 cp BRD-K19295594 gossypol BCL inhibitor

807 22.26 cp BRD-K16551401 PNU-22394 Serotonin receptor agonist

806 22.3 cp BRD-K30296925 flavokavain-b Antineoplastic

804 22.54 cp BRD-K43978949 PIT Purinergic receptor antagonist

802 22.78 cp BRD-K50720187 flupirtine Glutamate receptor antagonist

800 22.8 cp BRD-A30590053 MR-16728 Acetylcholine release enhancer

793 23.28 cp BRD-K43764301 dexketoprofen Cyclooxygenase inhibitor

792 23.3 cp BRD-K59851896 calycanthine GABA release inhibitor

791 23.4 cp BRD-K96799727 pifithrin-mu HSP inhibitor

790 23.42 cp BRD-K73589401 corticosterone mineralocorticoid receptor agonist

789 23.43 cp BRD-A00758722 noretynodrel Progestogen hormone

787 23.56 cp BRD-A52282606 lacidipine Calcium channel blocker

786 23.58 cp BRD-A03359064 ICI-89406 Adrenergic receptor antagonist

784 23.71 cp BRD-K22947005 dexbrompheniramine Histamine receptor antagonist

782 23.88 cp BRD-K51918615 iodophenpropit Histamine receptor antagonist

779 23.94 cp BRD-A70514680 articaine Local anesthetic

778 23.95 cp BRD-K56614220 clofazimine GK0582 inhibitor

777 23.99 cp BRD-K69763916 LY-341495 Glutamate receptor antagonist

771 24.29 cp BRD-K71926323 marbofloxacin Bacterial DNA gyrase inhibitor

770 24.33 cp BRD-K84595254 strophanthidin ATPase inhibitor

769 24.42 cp BRD-A96255180 ribavirin Antiviral

768 24.51 cp BRD-K45542189 diethylcarbamazine Lipoxygenase inhibitor

762 24.87 cp BRD-K77390737 xanthohumol ATPase inhibitor

763 24.87 cp BRD-A11990600 lorazepam Benzodiazepine receptor agonist

759 25.08 cp BRD-K01292756 pimozide Dopamine receptor antagonist

756 25.33 cp BRD-K32744045 disulfiram Aldehyde dehydrogenase inhibitor

755 25.38 cp BRD-K35573744 erbstatin-analog EGFR inhibitor

753 25.45 cp BRD-A85234536 N6-cyclopentyladenosine Adenosine receptor agonist

752 25.46 cp BRD-K30697463 desoximetasone Glucocorticoid receptor agonist

751 25.5 cp BRD-K36198571 WAY-170523 Metalloproteinase inhibitor

745 25.74 cp BRD-K35240538 methylprednisolone Glucocorticoid receptor agonist

743 25.76 cp BRD-K15715913 fluperlapine Serotonin receptor antagonist

740 25.8 cp BRD-A57457122 VU-0400071-3 Glutamate receptor modulator

739 25.84 cp BRD-K97399794 quercetin Polar auxin transport inhibitor

738 26.02 cp BRD-K05804044 AZ-628 RAF inhibitor

735 26.13 cp BRD-K98548675 parthenolide NFkB pathway inhibitor

734 26.17 cp BRD-K33226500 indinavir HIV protease inhibitor

730 26.24 cp BRD-K12079898 PD-160170 Neuropeptide receptor antagonist

727 26.37 cp BRD-M86331534 pyrvinium-pamoate AKT inhibitor

725 26.62 cp BRD-K46441700 GR-55562 Serotonin receptor antagonist

719 27 cp BRD-K93325701 damnacanthal SRC inhibitor

718 27.01 cp BRD-K62206109 VUF-5681 Histamine receptor antagonist

716 27.15 cp BRD-K34415467 trimethobenzamide Histamine receptor antagonist

714 27.49 cp BRD-K39120595 bithionol Autotaxin inhibitor

713 27.51 cp BRD-A25004090 erastin Ion channel antagonist

705 28.13 cp BRD-A15530910 carpindolol Adrenergic receptor antagonist

702 28.19 cp BRD-K30867024 SB-216641 Serotonin receptor antagonist

699 28.24 cp BRD-A01826957 xanthinol Vasodilator

690 28.75 cp BRD-K05901394 terguride Dopamine receptor agonist

686 28.87 cp BRD-K54233340 dorsomorphin AMPK inhibitor

685 29.11 cp BRD-K92301463 "16,16-dimethylprostaglandin-e2" Prostanoid receptor agonist

684 29.2 cp BRD-K37456065 VU-0365114-2 M5 modulator

682 29.24 cp BRD-K56301217 ABT-737 BCL inhibitor

681 29.28 cp BRD-A55484088 BNTX Opioid receptor antagonist

679 29.36 cp BRD-K20313525 rosmarinic-acid GABA transaminase inhibitor

678 29.61 cp BRD-K27710560 splitomycin SIRT inhibitor

674 29.8 cp BRD-U25771771 WZ-4-145 EGFR inhibitor

673 29.83 cp BRD-K96862998 pirfenidone TGF beta receptor inhibitor

664 30.37 cp BRD-A26095496 clobetasol Glucocorticoid receptor agonist

663 30.38 cp BRD-K06024458 n-arachidonyl-GABA cannabinoid receptor agonist

654 30.88 cp BRD-K32311154 nifekalant Potassium channel blocker

648 31.48 cp BRD-A43331270 niguldipine Calcium channel blocker

647 31.7 cp BRD-A96897502 U-74389F Lipid peroxidase inhibitor

639 32.03 cp BRD-K25875056 SC-9 Protein tyrosine kinase activator

638 32.04 cp BRD-K92428232 GSK-461364 PLK inhibitor

632 32.35 cp BRD-K75478907 GS-39783 GABA receptor modulator

631 32.38 cp BRD-K03642198 AY-9944 Hedgehog pathway modulator

627 32.52 cp BRD-K86930074 cediranib KIT inhibitor

622 32.87 cp BRD-A09495397 bicuculline GABA receptor antagonist

621 32.9 cp BRD-A82371568 clofarabine Ribonucleoside reductase inhibitor

619 33.01 cp BRD-K93258693 GW-9662 PPAR receptor antagonist

618 33.03 cp BRD-A79314293 cephalosporanic-acid Bacterial cell wall synthesis inhibitor

615 33.09 cp BRD-K10098805 rhapontin Apoptosis stimulant

614 33.12 cp BRD-A15079084 phorbol-12-myristate-13-acetate PKC activator

613 33.2 cp BRD-A27887842 prednisolone Glucocorticoid receptor agonist

612 33.34 cp BRD-K84987553 MDM2-inhibitor MDM inhibitor

606 33.64 cp BRD-K51575138 TPCA-1 IKK inhibitor

602 33.92 cp BRD-A95513702 "androsta-1,4-dien-3,17-dione" Aromatase inhibitor

596 34.37 cp BRD-K52620403 STO-609 Calmodulin antagonist

595 34.38 cp BRD-K31912990 CGP-71683 Neuropeptide receptor antagonist

581 34.93 cp BRD-K94070024 depomedrol Glucocorticoid receptor agonist

582 34.93 cp BRD-K17497770 butein EGFR inhibitor

579 34.98 cp BRD-A92439610 triamcinolone Glucocorticoid receptor agonist

578 35.07 cp BRD-A65440446 cimaterol Adrenergic receptor agonist

576 35.12 cp BRD-K33308633 INCA-6 Calcineurin inhibitor

575 35.16 cp BRD-K63150726 JTE-907 Cannabinoid receptor inverse agonist

573 35.41 cp BRD-K89930444 AG-592 Tyrosine kinase inhibitor

572 35.43 cp BRD-U86922168 QL-XII-47 BTK inhibitor

571 35.47 cp BRD-K19605405 ZM-241385 Adenosine receptor antagonist

566 35.74 cp BRD-K31238592 devazepide CCK receptor antagonist

561 36.16 cp BRD-K63089472 farnesylthioacetic-acid Inhibitor of methyl esterification of farnesylated proteins

560 36.21 cp BRD-A01593789 chlormadinone 5-alpha reductase inhibitor

557 36.54 cp BRD-A42346008 metanephrine Epinephrine metabolite

552 36.78 cp BRD-K96037667 norethindrone Progesterone receptor agonist

550 36.87 cp BRD-A39290993 cyproterone Androgen receptor antagonist

548 36.9 cp BRD-K10177585 PSB-11 Adenosine receptor antagonist

543 37.54 cp BRD-K95435023 PHA-665752 c-Met inhibitor

542 37.56 cp BRD-K12357156 AG-490 EGFR inhibitor

541 37.57 cp BRD-K97309399 thiothixene Dopamine receptor antagonist

531 38.15 cp BRD-K63979671 etifenin Compound used in hepatobiliary scans of the liver

527 38.33 cp BRD-A80502530 cinobufagin ATPase inhibitor

523 38.53 cp BRD-K82135108 elesclomol Oxidative stress inducer

516 38.84 cp BRD-K34437622 BRD-K34437622 Thymidylate synthase inhibitor

514 38.94 cp BRD-K72703948 ZM-447439 Aurora kinase inhibitor

512 39.3 cp BRD-A80775386 hyperforin Cyclooxygenase inhibitor

510 39.4 cp BRD-K09549677 mibefradil T-type calcium channel blocker

509 39.42 cp BRD-K35960502 niclosamide DNA replication inhibitor

507 39.7 cp BRD-K81225797 SCH-58261 Adenosine receptor antagonist

504 39.79 cp BRD-A56892734 esomeprazole ATPase inhibitor

505 39.79 cp BRD-K32292990 CGP-53353 EGFR inhibitor

496 40.25 cp BRD-K74402642 NSC-632839 Ubiquitin specific protease inhibitor

495 40.28 cp BRD-K21806131 tegaserod Serotonin receptor partial agonist

492 40.53 cp BRD-K95899059 LY-344864 Serotonin receptor agonist

490 40.57 cp BRD-A20697603 thiostrepton FOXM1 inhibitor

487 40.99 cp BRD-K07303502 arachidonyl-trifluoro-methane Cytosolic phospholipase inhibitor

486 41.06 cp BRD-A01346607 flumetasone Glucocorticoid receptor agonist

485 41.12 cp BRD-A75935363 atracurium Acetylcholine receptor antagonist

480 41.64 cp BRD-K60460488 nelfinavir HIV protease inhibitor

478 41.73 cp BRD-K22385716 LY-303511 Casein kinase inhibitor

474 41.97 cp BRD-A48570745 ivermectin GABA receptor agonist

471 42.15 cp BRD-A59808129 guggulsterone Cholesterol inhibitor

470 42.27 cp BRD-K29113274 ketoconazole Sterol demethylase inhibitor

469 42.52 cp BRD-K78838262 austricine Hypolipidemic

467 42.59 cp BRD-K41143549 BRD-K41143549 Glutamate receptor antagonist

463 43.09 cp BRD-K73982490 BI-78D3 JNK inhibitor

455 43.42 cp BRD-K08806317 timolol Adrenergic receptor antagonist

454 43.5 cp BRD-K61951118 FG-7142 GABA benzodiazepine site receptor inverse agonist

451 43.77 cp BRD-A52650764 ingenol PKC activator

444 44.17 cp BRD-A53131506 epitestosterone Inactive testosterone analog

441 44.46 cp BRD-K53123955 niridazole Phosphofructokinase inhibitor

439 44.82 cp BRD-K83289131 CAY-10618 NAMPT inhibitor

429 45.37 cp BRD-K68143200 SA-792541 CDC inhibitor

426 45.55 cp BRD-A42167015 carteolol Adrenergic receptor antagonist

421 45.96 cp BRD-A84327315 calcitriol Vitamin D receptor agonist

419 46.21 cp BRD-K14821540 FCCP Mitochondrial oxidative phosphorylation uncoupler

414 46.61 cp BRD-K40227168 vinburnine Adrenergic receptor antagonist

411 47.1 cp BRD-A62809825 thapsigargin ATPase inhibitor

407 47.25 cp BRD-K38477985 malonoben Protein tyrosine kinase inhibitor

403 47.52 cp BRD-K06878038 deferiprone Chelating agent

386 49.07 cp BRD-A82656074 naltrindole Opioid receptor antagonist

380 49.78 cp BRD-K75295174 alisertib Aurora kinase inhibitor

376 50.35 cp BRD-A06352418 terfenadine Histamine receptor antagonist

377 50.35 cp BRD-A71203467 l-stepholidine Dopamine receptor antagonist

362 51.8 cp BRD-K91315211 betahistine Histamine receptor agonist

358 52.04 cp BRD-K05528470 L-745870 Dopamine receptor antagonist

357 52.06 cp BRD-A79803969 memantine Glutamate receptor antagonist

354 53.04 cp BRD-K94919853 10H-phenothiazin-10-yl)(p-tolyl)methanone Butyrylcholinesterase inhibitor

350 53.66 cp BRD-K78599730 manumycin-a Farnesyltransferase inhibitor

349 53.77 cp BRD-A07765530 epinephrine carbonic anhydrase activator

342 54.3 cp BRD-K05977823 tenovins SIRT inhibitor

339 54.48 cp BRD-K85606544 neratinib EGFR inhibitor

330 55.33 cp BRD-K61737877 VEGF-receptor-2-kinase-inhibitor-IV VEGFR inhibitor

329 55.36 cp BRD-K74514084 pazopanib KIT inhibitor

328 55.37 cp BRD-K33583600 isoliquiritigenin Guanylate cyclase activator

327 55.43 cp BRD-K82941592 rosuvastatin HMGCR inhibitor

324 55.67 cp BRD-K19284129 salvinorin-a Opioid receptor agonist

323 55.79 cp BRD-A94756469 digoxin ATPase inhibitor

319 56.03 cp BRD-K93618743 ipriflavone Bone resorption inhibitor

302 56.93 cp BRD-A20126139 medrysone Glucocorticoid receptor agonist

301 56.95 cp BRD-K15409150 penfluridol T-type calcium channel blocker

300 57.01 cp BRD-K49294207 BIBU-1361 EGFR inhibitor

298 57.09 cp BRD-K39520573 GW-5074 Leucine rich repeat kinase inhibitor

296 57.26 cp BRD-A42628519 iopanoic-acid Radiopaque medium

292 57.85 cp BRD-A44448661 pentobarbital Barbiturate antiepileptic

285 58.31 cp BRD-A93236127 digitoxin ATPase inhibitor

278 59.41 cp BRD-K22631935 neurodazine Neurogenesis of non-pluripotent C2C12 myoblast inducer

277 59.93 cp BRD-K50168500 canertinib EGFR inhibitor

274 60.3 cp BRD-K50214219 CS-1657 PARP inhibitor

271 60.47 cp BRD-A11007541 BCI-hydrochloride Protein phosphatase inhibitor

262 61.2 cp BRD-K10573841 tunicamycin GLCNAC phosphotransferase inhibitor

253 62.25 cp BRD-K76872913 benzanthrone Aromatic hydrocarbon derivative

252 62.26 cp BRD-K64935403 ebelactone-b Lipase inhibitor

251 62.5 cp BRD-K78659596 MLN-2238 Proteasome inhibitor

248 62.81 cp BRD-K90543092 levonorgestrel Estrogen receptor agonist

247 63.29 cp BRD-K09436313 prostaglandin Prostanoid receptor antagonist

245 63.36 cp BRD-A08709697 heliotrine Pyrrolizidine alkaloid

242 63.63 cp BRD-A01320529 salmeterol Adrenergic receptor agonist

236 63.98 cp BRD-K34092021 arvanil TRPV agonist

229 64.81 cp BRD-A82590476 SDZ-NKT-343 Tachykinin antagonist

227 65.02 cp BRD-K57631554 aminolevulinic-acid Oxidizing agent

226 65.19 cp BRD-K03406345 azacitidine DNA methyltransferase inhibitor

221 65.44 cp BRD-A34806832 proscillaridin ATPase inhibitor

219 65.84 cp BRD-K03557653 sappanone-a Tyrosinase inhibitor

212 66.69 cp BRD-A72711497 lasalocid Bacterial permeability inducer

205 67.41 cp BRD-A29437505 RWJ-21757 TLR agonist

204 67.45 cp BRD-K64517075 heliomycin ATP synthase inhibitor

202 67.5 cp BRD-A43150328 penicillic-acid other antibiotic

199 68.25 cp BRD-A50737080 CGK-733 ATR kinase inhibitor

198 68.33 cp BRD-K40255344 tyrphostin-A9 Protein tyrosine kinase inhibitor

194 68.58 cp BRD-K20755323 SA-792728 Sphingosine kinase inhibitor

190 69.12 cp BRD-K28120222 parthenolide NFkB pathway inhibitor

182 69.88 cp BRD-A37052580 physostigmine Acetylcholinesterase inhibitor

176 71.27 cp BRD-K05151076 ZK-164015 Estrogen receptor antagonist

174 71.62 cp BRD-K76907295 VU-0418947-2 HIF modulator

175 71.62 cp BRD-K74305673 IKK-2-inhibitor-V IKK inhibitor

173 71.73 cp BRD-K56334280 amonafide Topoisomerase inhibitor

172 71.95 cp BRD-K60640630 mometasone Glucocorticoid receptor agonist

169 72.37 cp BRD-K91145395 prostratin PKC activator

164 73.93 cp BRD-K97764662 PD-173074 FGFR inhibitor

163 73.94 cp BRD-K44432556 VU-0418946-1 HIF modulator

160 74.2 cp BRD-A34205397 suloctidil Adrenergic receptor antagonist

155 74.74 cp BRD-K73395020 SA-1478088 -666

154 74.79 cp BRD-K01555864 dibenzoylmethane Antineoplastic

153 74.9 cp BRD-A17065207 brefeldin-a Protein synthesis inhibitor

151 75.38 cp BRD-K61480498 epoxycholesterol LXR agonist

147 76.04 cp BRD-K84895041 BMY-45778 IP1 prostacyclin receptor agonist

142 77.45 cp BRD-K68336408 tyrphostin-AG-1478 EGFR inhibitor

137 77.99 cp BRD-K05653692 DL-PDMP Glucosyltransferase inhibitor

133 78.26 cp BRD-K21672174 RO-28-1675 Glucokinase activator

128 78.8 cp BRD-K82795137 loratadine Histamine receptor antagonist

126 78.91 cp BRD-K36737713 AG-957 Protein tyrosine kinase inhibitor

123 79.52 cp BRD-K82823804 SA-792987 PKC inhibitor

118 80.1 cp BRD-K61829047 7b-cis Exportin antagonist

117 80.14 cp BRD-K64052750 gefitinib EGFR inhibitor

111 81.25 cp BRD-K26669427 WR-216174 PFMRK inhibitor

106 82.13 cp BRD-K31843556 T-0070907 PPAR receptor antagonist

105 82.18 cp BRD-A38030642 cyclosporin-a Calcineurin inhibitor

103 82.21 cp BRD-K51290057 SA-792709 Retinoid receptor agonist

102 82.31 cp BRD-A82238138 budesonide Glucocorticoid receptor agonist

98 82.63 cp BRD-A28105619 cucurbitacin-i JAK inhibitor

93 83.03 cp BRD-A15914070 4-hydroxy-2-nonenal Cytotoxic lipid peroxidation product

84 83.96 cp BRD-A62184259 cycloheximide Protein synthesis inhibitor

81 84.37 cp BRD-K85402309 dovitinib EGFR inhibitor

79 84.58 cp BRD-A34208323 VU-0404997-2 Glutamate receptor modulator

76 84.88 cp BRD-K66792149 quinoclamine Algicide

75 85 cp BRD-K56700933 phenethyl-isothiocyanate Antineoplastic

73 85.06 cp BRD-A73741725 exemestane Aromatase inhibitor

68 85.69 cp BRD-K94325918 kinetin-riboside Apoptosis stimulant

67 85.85 cp BRD-A56020723 CA-074-Me Cathepsin inhibitor

62 86.84 cp BRD-K33551950 radicicol HSP inhibitor

52 89.17 cp BRD-A72596465 GW-6471 PPAR receptor antagonist

50 89.53 cp BRD-K63606607 bufalin ATPase inhibitor

47 89.92 cp BRD-K51730347 diphencyprone Immunostimulant

33 92.16 cp BRD-K39983086 loteprednol Glucocorticoid receptor agonist

18 95.69 cp BRD-K80970344 pyrrolidine-dithiocarbamate NFkB pathway inhibitor

12 96.62 cp BRD-A23637604 oxymetholone Androgen receptor agonist
