# Supplementary material for: Bioinformatics-based screening and validation of ferroptosis-related genes in sepsis and type 2 diabetes mellitus
Source: Exp Biol Med (Maywood). 2025 Oct 21;250:10612. doi: 10.3389/ebm.2025.10612 (PMC12583109; doi:10.3389/ebm.2025.10612)
Supplement: Supplementary file 2 [file Table1.doc]

**Table S1** The information of the GEO cohort

| **ID** | **Platform** | **Sample** | **Control** | **Disease** |
| --- | --- | --- | --- | --- |
| GSE76894 | GEO | 103 | 84 | 19 |
| GSE65682 | GEO | 802 | 42 | 760 |

**Table S2 Sample information for qRT-PCR**

| **Group** | **Control**  **(n=5)** | **Sepsis**  **(n=5)** | **T2DM with Sepsis**  **(n=5)** |
| --- | --- | --- | --- |
| Age (Y) | 60,65,66,70,71 | 61,66,67,70,72 | 63,68,69,75,78 |
| Sex (Male/Female) | M (2), F (3) | M (2), F (3) | M (1), F (4) |

**Table S3 Primer sequences information**

| **Gene** | **Primer sequences（5’-3’）** | |
| --- | --- | --- |
| Hsa-CDC25B | F | ACGCACCTATCCCTGTCTC |
|  | R | CTGGAAGCGTCTGATGGCAA |
| Hsa-DPP7 | F | GGACCACTTCAACTTCGAGC |
|  | R | GCCCTCGTTCCCAGTGTAG |
| Hsa-FBXO31 | F | GCCGTGAGGAGTATGGTGTTT |
|  | R | GTACATCCACCCGATGATGAAC |
| Hsa-PTCD3 | F | TTTATTCTGGTAGTGCAACCCTC |
|  | R | AAGAACGGCTACTTTATCCCAAG |
| Hsa-CNPY2 | F | TCTGGTGGATGAACTAGAATGGG |
|  | R | TGTTTGGGGGAACAGTAACAGT |
| Hsa-GAPDH | F | AAGGTTTCTGGTTCAGGCGTGG |
|  | R | GCGTGGTCAGGACGTTCGTGT |

*Abbreviations: F, forward; R, reverse.*

**Table S4 The versions of** R and Bioconductor package

| **Package** | **Version** | **Package** | **Version** | **Package** | **Version** |
| --- | --- | --- | --- | --- | --- |
| sessioninfo | 1.2.2 | dplyr | 1.1.4 | WGCNA | 1.72-1 |
| ggExtra | 0.10.1 | Hmisc | 5.1-1 | fastcluster | 1.2.3 |
| RcisTarget.hg19.motifDBs.cisbpOnly.500bp | 1.18.0 | e1071 | 1.7-13 | dynamicTreeCut | 1.63-1 |
| cowplot | 1.1.1 | AUCell | 1.20.2 | ggplot2 | 3.5.1 |
| SimDesign | 2.13 | stringr | 1.5.0 | enrichplot | 1.22.0 |
| vioplot | 0.4.0 | reshape2 | 1.4.4 | clusterProfiler | 4.6.2 |
| zoo | 1.8-12 | visNetwork | 2.1.2 | org.Hs.eg.db | 3.16.0 |
| sm | 2.2-5.7.1 | RcisTarget | 1.18.2 | AnnotationDbi | 1.64.1 |
| corrplot | 0.92 | GSEABase | 1.60.0 | IRanges | 2.32.0 |
| DT | 0.33 | graph | 1.76.0 | S4Vectors | 0.36.2 |
| doMC | 1.3.5 | annotate | 1.76.0 | Biobase | 2.58.0 |
| iterators | 1.0.14 | XML | 3.99-0.14 | BiocGenerics | 0.44.0 |
| doRNG | 1.8.6 | GSVA | 1.46.0 | gplots | 3.1.3 |
| rngtools | 1.5.2 | pROC | 1.18.4 | pheatmap | 1.0.12 |
| foreach | 1.5.2 | ggpubr | 0.6.0 | limma | 3.54.2 |
| aplot | 0.2.2 | WGCNA | 1.72-1 |  |  |

**Table S5 The specific AUC and NES thresholds**

| **Logo** | **Geneset** | **Motif** | **AUC** | **NES** | **TF-highConf** | **nEnrGenes** | **EnrichedGenes** |
| --- | --- | --- | --- | --- | --- | --- | --- |
| 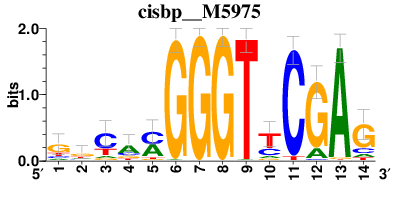 | Key-gene | cisbp__M5975 | 0.196 | 8.73 | ZNF524(directAnnotation) | 2 | CDC25B;  CNPY2 |
| 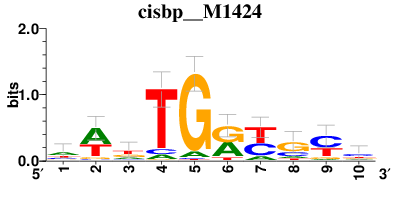 | Key-gene | cisbp__M1424 | 0.192 | 8.56 |  | 1 | CDC25B |
| 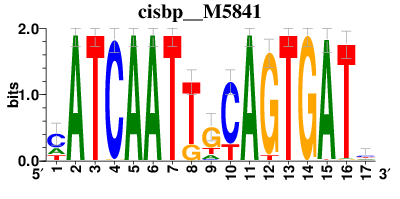 | Key-gene | cisbp__M5841 | 0.19 | 8.5 | SOX8(directAnnotation) | 1 | CDC25B |
| 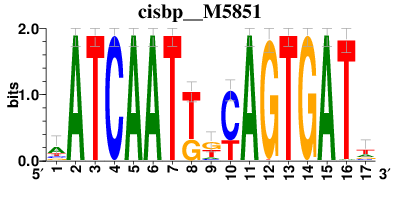 | Key-gene | cisbp__M5851 | 0.19 | 8.46 | SOX9(directAnnotation) | 1 | CDC25B |
| 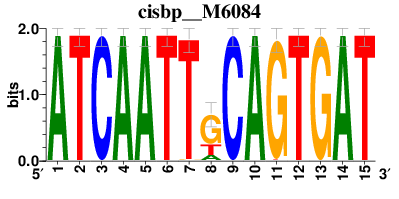 | Key-gene | cisbp__M6084 | 0.189 | 8.45 | SOX10(inferredBy-Orthology) | 1 | CDC25B |
| 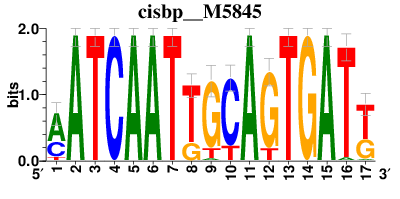 | Key-gene | cisbp__M5845 | 0.189 | 8.41 | SOX8(directAnnotation) | 1 | CDC25B |
| 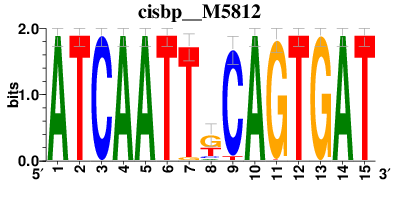 | Key-gene | cisbp__M5812 | 0.187 | 8.35 | SOX10(directAnnotation) | 1 | CDC25B |
| 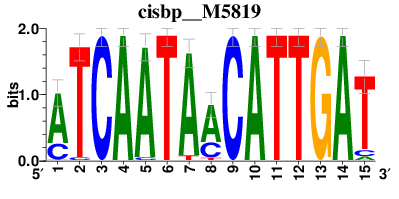 | Key-gene | cisbp__M5819 | 0.183 | 8.16 | SOX15(directAnnotation) | 1 | CDC25B |
| 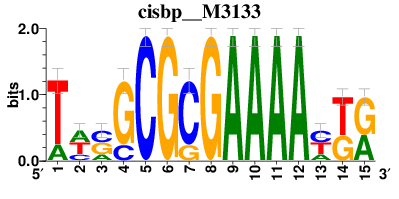 | Key-gene | cisbp__M3133 | 0.181 | 8.08 | E2F1(directAnnotation) | 1 | CDC25B |
| 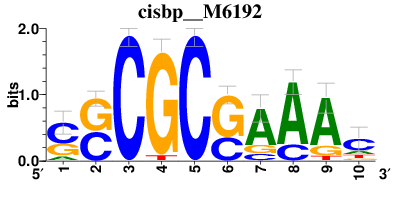 | Key-gene | cisbp__M6192 | 0.181 | 8.07 | E2F3(directAnnotation) | 1 | CDC25B |
