# Supplementary material for: Bioinformatics-based screening and validation of ferroptosis-related genes in sepsis and type 2 diabetes mellitus
Source: Exp Biol Med (Maywood). 2025 Oct 21;250:10612. doi: 10.3389/ebm.2025.10612 (PMC12583109; doi:10.3389/ebm.2025.10612)
Supplement: Supplementary file 3 [file Table2.doc]

Rank Score Type ID Name Description

8553 -98.31 cp BRD-K69840642 ISOX HDAC inhibitor

8544 -91.85 cp BRD-K12867552 THM-I-94 HDAC inhibitor

8543 -91.56 cp BRD-K81418486 vorinostat HDAC inhibitor

8541 -90.51 cp BRD-K74761218 WT-171 HDAC inhibitor

8539 -90.43 cp BRD-K02130563 panobinostat HDAC inhibitor

8535 -87.31 cp BRD-K56957086 dacinostat HDAC inhibitor

8533 -86.24 cp BRD-K11558771 droxinostat HDAC inhibitor

8531 -84.19 cp BRD-K17743125 belinostat HDAC inhibitor

8529 -82.9 cp BRD-K64606589 apicidin HDAC inhibitor

8526 -81.55 cp BRD-K68202742 trichostatin-a HDAC inhibitor

8515 -71.49 cp BRD-K06080977 eicosatetraynoic-acid Cyclooxygenase inhibitor

8514 -70.38 cp BRD-A39646320 HC-toxin HDAC inhibitor

8500 -59.96 cp BRD-K06750613 GSK-1059615 PI3K inhibitor

8493 -57.47 cp BRD-K64890080 BI-2536 PLK inhibitor

8489 -54.84 cp BRD-A08877921 cephalotaxine Protein synthesis inhibitor

8485 -49.51 cp BRD-K01436366 XMD-1150 Leucine rich repeat kinase inhibitor

8479 -47.54 cp BRD-K31238592 devazepide CCK receptor antagonist

8475 -45.44 cp BRD-K22385716 LY-303511 Casein kinase inhibitor

8472 -44.5 cp BRD-K74733595 APHA-compound-8 HDAC inhibitor

8468 -42.41 cp BRD-A17065207 brefeldin-a Protein synthesis inhibitor

8466 -41.64 cp BRD-K59184148 SB-216763 Glycogen synthase kinase inhibitor

8464 -40.38 cp BRD-K84895041 BMY-45778 IP1 prostacyclin receptor agonist

8460 -39.99 cp BRD-K74913225 brinzolamide Carbonic anhydrase inhibitor

8459 -39.89 cp BRD-K94070024 depomedrol Glucocorticoid receptor agonist

8458 -39.3 cp BRD-K57546357 prunetin Breast cancer resistance protein inhibitor

8455 -38.64 cp BRD-K56403959 ZK-756326 CC chemokine receptor ligand

8450 -37.16 cp BRD-K50387473 XMD-892 MAP kinase inhibitor

8448 -35.96 cp BRD-K68336408 tyrphostin-AG-1478 EGFR inhibitor

8446 -35.14 cp BRD-A56020723 CA-074-Me Cathepsin inhibitor

8442 -33.86 cp BRD-K11663430 pyroxamide HDAC inhibitor

8441 -32.62 cp BRD-A08187463 racecadotril Enkephalinase inhibitor

8439 -31.53 cp BRD-K81709173 halcinonide Glucocorticoid receptor agonist

8435 -29.56 cp BRD-K03642198 AY-9944 Hedgehog pathway modulator

8433 -28.71 cp BRD-K33583600 isoliquiritigenin Guanylate cyclase activator

8432 -28.57 cp BRD-K55424922 anpirtoline Serotonin receptor agonist

8428 -27.92 cp BRD-K94832621 Y-134 Estrogen receptor antagonist

8422 -26.68 cp BRD-K79404599 enzastaurin PKC inhibitor

8416 -25.04 cp BRD-A85234536 N6-cyclopentyladenosine Adenosine receptor agonist

8415 -24.86 cp BRD-K22503835 scriptaid HDAC inhibitor

8414 -24.52 cp BRD-K13810148 givinostat HDAC inhibitor

8409 -23.36 cp BRD-A32349859 methyl-angolensate Apoptosis inhibitor

8406 -22.86 cp BRD-K31491153 1-phenylbiguanide Serotonin receptor agonist

8405 -22.64 cp BRD-U01690642 acetyl-geranyl-cysteine Isoprenylated protein methylation inhibitor

8403 -22.53 cp BRD-K53959060 indirubin CDK inhibitor

8399 -22.26 cp BRD-K47328134 lysylphenylalanyl-tyrosine Heparin activation inhibitor

8396 -21.64 cp BRD-A10969569 ambelline Plant alkaloid

8395 -21.32 cp BRD-K88871508 lisuride Dopamine receptor agonist

8393 -21.19 cp BRD-K99291625 SB-203580 p38 MAPK inhibitor

8385 -19.94 cp BRD-K28578425 cilostamide Phosphodiesterase inhibitor

8383 -19.8 cp BRD-K14696368 "9-methyl-5H-6-thia-4,5-diaza-chrysene-6,6-dioxide" NFkB pathway inhibitor

8378 -18.9 cp BRD-K55344148 BU-224 Imidazoline receptor ligand

8377 -18.89 cp BRD-A65013509 oxybutynin Acetylcholine receptor antagonist

8371 -18.01 cp BRD-U88459701 atorvastatin HMGCR inhibitor

8367 -17.4 cp BRD-K01567962 pyrazolanthrone JNK inhibitor

8362 -15.98 cp BRD-K33226500 indinavir HIV protease inhibitor

8357 -15.84 cp BRD-K48722258 dilazep Adenosine reuptake inhibitor

8356 -15.68 cp BRD-K41445866 alfaxalone Chloride channel agonist

8350 -14.85 cp BRD-K30570479 VU-0400195-3 Glutamate receptor modulator

8347 -14.31 cp BRD-K50464341 berbamine Calmodulin antagonist

8346 -14.28 cp BRD-K52522949 NCH-51 HDAC inhibitor

8345 -14.19 cp BRD-K27710560 splitomycin SIRT inhibitor

8341 -13.68 cp BRD-K36737713 AG-957 Protein tyrosine kinase inhibitor

8339 -13.58 cp BRD-A62434282 goserelin Gonadotropin releasing factor hormone receptor agonist

8337 -13.41 cp BRD-K02404261 caffeine Adenosine receptor antagonist

8333 -12.87 cp BRD-A95696820 acadesine AMPK activator

8326 -12.33 cp BRD-K70883034 nimetazepam GABA receptor agonist

8321 -11.84 cp BRD-K23335153 AMN-082 Glutamate receptor modulator

8318 -11.41 cp BRD-A16700644 isoxsuprine Adrenergic receptor agonist

8313 -10.92 cp BRD-K54330070 SB-202190 p38 MAPK inhibitor

8312 -10.9 cp BRD-K96740444 itopride Dopamine receptor antagonist

8311 -10.86 cp BRD-K36395411 SB-206553 Serotonin receptor antagonist

8308 -10.46 cp BRD-A69960130 bromocriptine Dopamine receptor agonist

8306 -10.35 cp BRD-K63150726 JTE-907 Cannabinoid receptor inverse agonist

8305 -10.31 cp BRD-K61269089 daphnetin Protein kinase inhibitor

8296 -9.56 cp BRD-K15715913 fluperlapine Serotonin receptor antagonist

8295 -9.54 cp BRD-K96134740 kitasamycin Protein synthesis inhibitor

8294 -9.53 cp BRD-K67445247 flurofamide Urease inhibitor

8282 -9.07 cp BRD-A42553870 L-152804 Neuropeptide receptor antagonist

8275 -8.67 cp BRD-A81772229 simvastatin HMGCR inhibitor

8273 -8.53 cp BRD-A47706533 L-BSO Glutathione transferase inhibitor

8271 -8.27 cp BRD-A72483914 spiroxatrine Serotonin receptor antagonist

8267 -8.06 cp BRD-A18043272 phensuximide Succinimide antiepileptic

8266 -7.95 cp BRD-K30296925 flavokavain-b Antineoplastic

8263 -7.66 cp BRD-K50891186 GR-103691 Dopamine receptor antagonist

8262 -7.58 cp BRD-A18917088 estradiol Contraceptive agent

8261 -7.57 cp BRD-K68997413 PF-3845 FAAH inhibitor

8258 -7.42 cp BRD-K40965114 cyanopindolol Adrenergic receptor antagonist

8257 -7.38 cp BRD-K39120595 bithionol Autotaxin inhibitor

8254 -7.13 cp BRD-K32906660 bis-tyrphostin EGFR inhibitor

8252 -7.11 cp BRD-A31227688 kynuramine Aryl hydrocarbon receptor activator

8250 -7.04 cp BRD-K05350981 oligomycin-c ATPase inhibitor

8248 -6.88 cp BRD-K20742498 RS-39604 Serotonin receptor antagonist

8242 -6.8 cp BRD-K14807180 SB-221284 Serotonin receptor antagonist

8236 -6.56 cp BRD-K53913732 SB-408124 Orexin receptor antagonist

8235 -6.48 cp BRD-K43887077 dopamine Dopamine receptor agonist

8230 -6.27 cp BRD-K54416256 methimazole Antithyroid

8229 -6.26 cp BRD-K31843556 T-0070907 PPAR receptor antagonist

8227 -6.25 cp BRD-K08619574 thioproperazine Dopamine receptor antagonist

8226 -6.22 cp BRD-K99029477 prometon Photosynthesis inhibitor

8224 -6.21 cp BRD-A72711497 lasalocid Bacterial permeability inducer

8223 -6.2 cp BRD-K06024458 n-arachidonyl-GABA cannabinoid receptor agonist

8220 -6.06 cp BRD-K08109516 L-701324 Glutamate receptor antagonist

8217 -6 cp BRD-K37447567 hydrocotarnine Opioid receptor antagonist

8214 -5.85 cp BRD-K83010055 VU-0415374-1 Glutamate receptor modulator

8213 -5.84 cp BRD-K94353609 fluocinolone Glucocorticoid receptor agonist

8212 -5.82 cp BRD-K89687904 PKCbeta-inhibitor PKC inhibitor

8207 -5.62 cp BRD-K99922388 DPO-1 Potassium channel blocker

8205 -5.6 cp BRD-K27665173 D-64406 PDGFR receptor inhibitor

8202 -5.57 cp BRD-K94919853 10H-phenothiazin-10-yl)(p-tolyl)methanone Butyrylcholinesterase inhibitor

8203 -5.57 cp BRD-A46179541 doxapram Potassium channel blocker

8201 -5.54 cp BRD-A62021152 WAY-161503 Serotonin receptor agonist

8200 -5.51 cp BRD-K41143549 BRD-K41143549 Glutamate receptor antagonist

8198 -5.47 cp BRD-K49294207 BIBU-1361 EGFR inhibitor

8196 -5.37 cp BRD-A26711594 nicardipine Calcium channel blocker

8187 -5.12 cp BRD-A77050075 heraclenol Vitamin K antagonist

8184 -5.07 cp BRD-K03319035 maprotiline Norepinephrine reuptake inhibitor

8180 -5 cp BRD-K09991945 GSK-3-inhibitor-II PKC inhibitor

8176 -4.97 cp BRD-K33572481 taurodeoxycholic-acid Bile acid

8177 -4.97 cp BRD-A50157456 terbutaline Adrenergic receptor agonist

8175 -4.89 cp BRD-K57080016 selumetinib MEK inhibitor

8172 -4.83 cp BRD-K12002134 megestrol progesterone receptor agonist

8169 -4.72 cp BRD-K72541103 JAK3-inhibitor-I JAK inhibitor

8165 -4.51 cp BRD-K34415467 trimethobenzamide Histamine receptor antagonist

8158 -4.45 cp BRD-K51805276 temefos Cholinesterase inhibitor

8149 -4.26 cp BRD-A18579359 wiskostatin Neural Wiskott-Aldrich syndrome protein inhibitor

8145 -4.16 cp BRD-A54029483 IRL-2500 Endothelin receptor antagonist

8141 -4.12 cp BRD-K56064827 EI-273 PKC inhibitor

8139 -4.09 cp BRD-K59650319 YM-298198 Glutamate receptor antagonist

8128 -3.95 cp BRD-A19248578 latrunculin-b Actin polymerization inhibitor

8125 -3.89 cp BRD-A56675431 altizide Thiazide diuretic

8123 -3.84 cp BRD-A42346008 metanephrine Epinephrine metabolite

8122 -3.82 cp BRD-K73293050 WZ-3146 EGFR inhibitor

8115 -3.7 cp BRD-K12502280 TG-101348 FLT3 inhibitor

8116 -3.7 cp BRD-K16508793 diazepam Benzodiazepine receptor agonist

8110 -3.65 cp BRD-K82941592 rosuvastatin HMGCR inhibitor

8108 -3.59 cp BRD-K37618799 MRS-1220 Adenosine receptor antagonist

8099 -3.49 cp BRD-A10070317 propranolol Adrenergic receptor antagonist

8095 -3.45 cp BRD-A38898897 GW-311616 Leukocyte elastase inhibitor

8092 -3.4 cp BRD-A18763547 BAX-channel-blocker Cytochrome C release inhibitor

8090 -3.38 cp BRD-K04976539 aminogenistein SRC inhibitor

8087 -3.36 cp BRD-K24132293 piperlongumine Glutathione transferase inhibitor

8086 -3.35 cp BRD-K41859756 NVP-AUY922 HSP inhibitor

8072 -3.23 cp BRD-K10098805 rhapontin Apoptosis stimulant

8069 -3.17 cp BRD-K64935403 ebelactone-b Lipase inhibitor

8066 -3.15 cp BRD-A39268308 epibatidine Acetylcholine receptor agonist

8061 -3.07 cp BRD-K53545112 CNQX Glutamate receptor antagonist

8054 -3.05 cp BRD-A09467419 mebeverine Acetylcholine receptor antagonist

8058 -3.05 cp BRD-A31800922 procyclidine Acetylcholine receptor antagonist

8053 -3.03 cp BRD-A65615053 zacopride Serotonin receptor antagonist

8047 -2.97 cp BRD-A50737080 CGK-733 ATR kinase inhibitor

8045 -2.96 cp BRD-K12260308 xanthoxyline Antifungal

8046 -2.96 cp BRD-K56596464 QX-314 Sodium channel blocker

8040 -2.88 cp BRD-K61951118 FG-7142 GABA benzodiazepine site receptor inverse agonist

8037 -2.85 cp BRD-A26002865 verrucarin-a Protein synthesis inhibitor

8034 -2.81 cp BRD-A29289453 PCA-4248 Platelet activating factor receptor antagonist

8030 -2.78 cp BRD-K94689771 pinocembrin CYP1B1 inhibitor

8031 -2.78 cp BRD-K00675675 CL-82198 Metalloproteinase inhibitor

8032 -2.78 cp BRD-A75479906 rimantadine Antiviral

8028 -2.75 cp BRD-K31471398 dihydrexidine Dopamine receptor agonist

8023 -2.68 cp BRD-K09549677 mibefradil T-type calcium channel blocker

8021 -2.66 cp BRD-A62071884 siguazodan Phosphodiesterase inhibitor

8018 -2.64 cp BRD-A34205397 suloctidil Adrenergic receptor antagonist

8019 -2.64 cp BRD-K85871428 SC-68376 p38 MAPK inhibitor

8016 -2.63 cp BRD-K11905747 spectinomycin Bacterial 30S ribosomal subunit inhibitor

8009 -2.57 cp BRD-K76064317 tyrphostin-AG-1296 FLT3 inhibitor

8003 -2.49 cp BRD-K29950728 clomifene Estrogen receptor antagonist

7996 -2.43 cp BRD-K47693913 evoxine Furoquinoline alkaloid

7997 -2.43 cp BRD-A39290993 cyproterone Androgen receptor antagonist

7998 -2.43 cp BRD-K40624912 ZM-39923 JAK inhibitor

7992 -2.4 cp BRD-K41410256 balsalazide Cyclooxygenase inhibitor

7988 -2.37 cp BRD-K24538644 KUC104502N -666

7985 -2.36 cp BRD-A54596827 solifenacin Acetylcholine receptor antagonist

7982 -2.34 cp BRD-K81376179 TCS-359 FLT3 inhibitor

7981 -2.33 cp BRD-K25310650 ormetoprim Bacterial antifolate

7977 -2.3 cp BRD-A71009679 KUC103420N -666

7967 -2.26 cp BRD-K46766488 S-14506 Serotonin receptor agonist

7968 -2.26 cp BRD-K53263234 CITCO CAR agonist

7964 -2.22 cp BRD-K08206212 entecavir Reverse transcriptase inhibitor

7965 -2.22 cp BRD-K27141178 SB-203186 Serotonin receptor antagonist

7962 -2.18 cp BRD-K28183345 proguanil Dihydrofolate reductase inhibitor

7953 -2.15 cp BRD-K34608650 BRD-K34608650 Cannabinoid receptor agonist

7944 -2.11 cp BRD-K64670467 JNJ-16259685 Glutamate receptor antagonist

7945 -2.11 cp BRD-K48735772 PD-158780 EGFR inhibitor

7946 -2.11 cp BRD-K74765201 tomelukast Leukotriene receptor antagonist

7947 -2.11 cp BRD-K19136521 indirubin CDK inhibitor

7948 -2.11 cp BRD-A91452556 estradiol-cypionate Estrogen receptor agonist

7942 -2.08 cp BRD-A54490543 pirlindole Monoamine oxidase inhibitor

7935 -2.04 cp BRD-K92984783 melperone Serotonin receptor antagonist

7936 -2.04 cp BRD-K08502430 angiogenesis-inhibitor Angiogenesis inhibitor

7937 -2.04 cp BRD-K21680192 mitoxantrone Topoisomerase inhibitor

7933 -2.01 cp BRD-K14618467 IKK-16 IKK inhibitor

7924 -1.97 cp BRD-K50324045 avrainvillamide-analog-6 nucleophosmin inhibitor

7925 -1.97 cp BRD-A71262238 nafadotride Dopamine receptor antagonist

7926 -1.97 cp BRD-K15791587 L-733060 Tachykinin antagonist

7922 -1.96 cp BRD-A32164164 methyllycaconitine Acetylcholine receptor antagonist

7918 -1.94 cp BRD-A87387433 cefpodoxime Bacterial cell wall synthesis inhibitor

7919 -1.94 cp BRD-K88677950 PD-198306 MAP kinase inhibitor

7914 -1.9 cp BRD-K00337317 NU-7441 DNA dependent protein kinase inhibitor

7915 -1.9 cp BRD-A66927094 nemonapride Dopamine receptor antagonist

7916 -1.9 cp BRD-K31912990 CGP-71683 Neuropeptide receptor antagonist

7909 -1.87 cp BRD-K34092021 arvanil TRPV agonist

7911 -1.87 cp BRD-K53561341 KIN001-220 Aurora kinase inhibitor

7903 -1.83 cp BRD-A59808129 guggulsterone Cholesterol inhibitor

7897 -1.81 cp BRD-K78637815 LY-320135 Cannabinoid receptor antagonist

7895 -1.78 cp BRD-K01648091 LE-300 Dopamine receptor antagonist

7894 -1.77 cp BRD-K46742498 alosetron Serotonin receptor antagonist

7888 -1.76 cp BRD-K11107424 tiotidine Histamine receptor antagonist

7889 -1.76 cp BRD-A41722204 sulmazole Adenosine receptor antagonist

7890 -1.76 cp BRD-A65440446 cimaterol Adrenergic receptor agonist

7892 -1.76 cp BRD-A97739905 ketoprofen Cyclooxygenase inhibitor

7893 -1.76 cp BRD-K63430059 methoxsalen DNA synthesis inhibitor

7883 -1.73 cp BRD-K18523449 mestanolone Androgenic steroid

7884 -1.73 cp BRD-K64044582 linoleamide ACAT inhibitor

7885 -1.73 cp BRD-K37312348 kenpaullone CDK inhibitor

7882 -1.71 cp BRD-A84389633 "tropanyl-3,5-dimethylbenzoate" Serotonin receptor antagonist

7878 -1.69 cp BRD-K36198571 WAY-170523 Metalloproteinase inhibitor

7879 -1.69 cp BRD-K67013324 luzindole Melatonin receptor antagonist

7880 -1.69 cp BRD-K93480852 KN-93 Calcium-calmodulin dependent protein kinase inhibitor

7881 -1.69 cp BRD-K96119599 leucodin Melanin inhibitor

7876 -1.68 cp BRD-K91904471 SD-169 p38 MAPK inhibitor

7871 -1.66 cp BRD-K10961822 latanoprost Prostanoid receptor agonist

7872 -1.66 cp BRD-K51730347 diphencyprone Immunostimulant

7874 -1.66 cp BRD-K72093121 vidarabine Antiviral

7864 -1.62 cp BRD-K26674531 GR-235 Estrogen receptor agonist

7865 -1.62 cp BRD-K51751936 alfadolone GABA receptor agonist

7866 -1.62 cp BRD-K41564320 purvalanol-b Tyrosine kinase inhibitor

7860 -1.59 cp BRD-K18059238 gamma-linolenic-acid Cyclooxygenase inhibitor

7853 -1.57 cp BRD-A64125466 dehydrocholic-acid choleretic agent

7851 -1.55 cp BRD-K71430621 clobenpropit Histamine receptor antagonist

7852 -1.55 cp BRD-A22143024 estropipate Estrogen receptor agonist

7850 -1.54 cp BRD-A35989968 megestrol Progesterone receptor agonist

7847 -1.53 cp BRD-K66175015 afatinib EGFR inhibitor

7844 -1.52 cp BRD-K13514097 everolimus MTOR inhibitor

7845 -1.52 cp BRD-K92817986 BJM-CSC-19 MEK inhibitor

7846 -1.52 cp BRD-K17896185 FIT Opioid receptor agonist

7840 -1.48 cp BRD-A26845397 isamoltan Adrenergic receptor antagonist

7834 -1.44 cp BRD-K83972459 JWE-035 Aurora kinase inhibitor

7835 -1.44 cp BRD-K39983086 loteprednol Glucocorticoid receptor agonist

7833 -1.41 cp BRD-K62353524 DY-131 Estrogen receptor agonist

7827 -1.37 cp BRD-K53523901 arctigenin MEK inhibitor

7829 -1.37 cp BRD-K77908580 entinostat HDAC inhibitor

7818 -1.34 cp BRD-K35240538 methylprednisolone Glucocorticoid receptor agonist

7819 -1.34 cp BRD-K01638814 rilmenidine Adrenergic receptor agonist

7820 -1.34 cp BRD-K20285085 fostamatinib SYK inhibitor

7821 -1.34 cp BRD-K65285700 BRD-K65285700 Cannabinoid receptor agonist

7826 -1.34 cp BRD-K35559145 levomepromazine Dopamine receptor antagonist

7809 -1.3 cp BRD-K27871032 lysergol Ergoline alkaloid

7810 -1.3 cp BRD-K84709232 caffeic-acid Lipoxygenase inhibitor

7811 -1.3 cp BRD-A44448661 pentobarbital Barbiturate antiepileptic

7813 -1.3 cp BRD-K51575138 TPCA-1 IKK inhibitor

7805 -1.27 cp BRD-K89375097 pirenzepine Acetylcholine receptor antagonist

7806 -1.27 cp BRD-K62289640 lylamine Cannabinoid receptor agonist

7797 -1.23 cp BRD-K29653726 topiramate Carbonic anhydrase inhibitor

7798 -1.23 cp BRD-A51820102 econazole Bacterial cell wall synthesis inhibitor

7796 -1.22 cp BRD-K27737647 H-89 PKA inhibitor

7787 -1.2 cp BRD-K93325701 damnacanthal SRC inhibitor

7788 -1.2 cp BRD-K26669427 WR-216174 PFMRK inhibitor

7789 -1.2 cp BRD-K43290182 Ro-04-6790 Serotonin receptor antagonist

7791 -1.2 cp BRD-A47494775 dipivefrine Adrenergic receptor agonist

7792 -1.2 cp BRD-A55393291 testosterone Androgen receptor agonist

7785 -1.19 cp BRD-K32795028 1-benzylimidazole Thromboxane synthase inhibitor

7781 -1.18 cp BRD-K81847782 scandenin Plant compound with antimicrobial activity

7773 -1.16 cp BRD-A51410489 yohimbine Adrenergic receptor antagonist

7774 -1.16 cp BRD-K32755366 reserpic-acid Norepinephrine transporter inhibitor

7775 -1.16 cp BRD-K31792052 pifithrin Interleukin receptor antagonist

7776 -1.16 cp BRD-K78692225 leflunomide Dihydroorotate dehydrogenase inhibitor

7777 -1.16 cp BRD-K01815685 indole aryl hydrocarbon receptor agonist

7778 -1.16 cp BRD-K42098891 protriptyline Tricyclic antidepressant

7770 -1.13 cp BRD-K60230970 MG-132 Proteasome inhibitor

7771 -1.13 cp BRD-K07220430 cinnarizine Calcium channel blocker

7760 -1.09 cp BRD-K94176593 TWS-119 Glycogen synthase kinase inhibitor

7761 -1.09 cp BRD-K97399794 quercetin Polar auxin transport inhibitor

7762 -1.09 cp BRD-K69763916 LY-341495 Glutamate receptor antagonist

7763 -1.09 cp BRD-K05528470 L-745870 Dopamine receptor antagonist

7764 -1.09 cp BRD-A09056319 alfuzosin Adrenergic receptor antagonist

7765 -1.09 cp BRD-A25004090 erastin Ion channel antagonist

7756 -1.06 cp BRD-A49370193 RO-60-0175 Serotonin receptor agonist

7757 -1.06 cp BRD-A48257147 PHCCC Glutamate receptor agonist

7749 -1.02 cp BRD-A68929948 DAPT-GSI-IX Gamma secretase inhibitor

7750 -1.02 cp BRD-A71157293 fursultiamine Vitamin B

7752 -1.02 cp BRD-K05653692 DL-PDMP Glucosyltransferase inhibitor

7744 -0.99 cp BRD-A96897502 U-74389F Lipid peroxidase inhibitor

7745 -0.99 cp BRD-K82823076 RO-15-4513 GABA benzodiazepine site receptor inverse agonist

7743 -0.98 cp BRD-A96456596 FPL-55712 Leukotriene receptor antagonist

7739 -0.96 cp BRD-A09161221 nomilin HSP inhibitor

7735 -0.95 cp BRD-K11911061 GR-127935 Serotonin receptor antagonist

7736 -0.95 cp BRD-K45861246 azaperone Dopamine receptor antagonist

7738 -0.95 cp BRD-K67261995 adipiodone Contrast agent

7731 -0.92 cp BRD-K90333595 phentolamine Adrenergic receptor antagonist

7732 -0.92 cp BRD-K08132273 tyrphostin EGFR inhibitor

7733 -0.92 cp BRD-K68402494 ML-9 Myosin light chain kinase inhibitor

7734 -0.92 cp BRD-A70083328 secnidazole Acetylcholinesterase inhibitor

7725 -0.88 cp BRD-K63913457 eicosatrienoic-acid Vasodilator

7726 -0.88 cp BRD-A49225603 alimemazine Histamine receptor agonist

7727 -0.88 cp BRD-K59332007 linopirdine Potassium channel blocker

7728 -0.88 cp BRD-K14920963 erythrosine Coloring agent

7729 -0.88 cp BRD-K91263825 nortriptyline Tricyclic antidepressant

7722 -0.85 cp BRD-K35430135 SR-59230A Adrenergic receptor antagonist

7723 -0.85 cp BRD-K26548821 quinpirole Dopamine receptor agonist

7718 -0.84 cp BRD-K44094599 tacrolimus Calcineurin inhibitor

7710 -0.81 cp BRD-K86930074 cediranib KIT inhibitor

7711 -0.81 cp BRD-K08640512 RS-100329 Adrenergic receptor antagonist

7712 -0.81 cp BRD-K84987553 MDM2-inhibitor MDM inhibitor

7713 -0.81 cp BRD-K17743697 KB-R7943 Sodium/calcium exchange inhibitor

7714 -0.81 cp BRD-A65145453 ATPA Glutamate receptor agonist

7715 -0.81 cp BRD-K01663662 diphenidol Acetylcholine receptor agonist

7708 -0.8 cp BRD-K96720755 relcovaptan Vasopressin receptor antagonist

7709 -0.8 cp BRD-A02759312 betaxolol Adrenergic receptor antagonist

7703 -0.78 cp BRD-K19462402 buflomedil Adrenergic receptor antagonist

7704 -0.78 cp BRD-K28453807 nitrocaramiphen Cholinergic receptor antagonist

7705 -0.78 cp BRD-A31575449 CGP-20712 Adrenergic receptor antagonist

7707 -0.78 cp BRD-K74133369 oligomycin-a ATP synthase inhibitor

7700 -0.76 cp BRD-K50938786 ropivacaine Sodium channel blocker

7699 -0.75 cp BRD-K95885906 quercetagetin PIM inhibitor

7693 -0.74 cp BRD-K05104363 PD-184352 MEK inhibitor

7694 -0.74 cp BRD-K00959089 thenoyltrifluoroacetone Chelating agent

7695 -0.74 cp BRD-K54790157 trioxsalen DNA synthesis inhibitor

7696 -0.74 cp BRD-K14441456 tyrphostin-AG-556 EGFR inhibitor

7697 -0.74 cp BRD-K94512704 spiramide Dopamine receptor antagonist

7698 -0.74 cp BRD-K85606544 neratinib EGFR inhibitor

7684 -0.7 cp BRD-K52930707 rescinnamine ACE inhibitor

7685 -0.7 cp BRD-K36009368 NNC-63-0532 Opioid receptor agonist

7686 -0.7 cp BRD-K10705233 GW-405833 Cannabinoid receptor agonist

7687 -0.7 cp BRD-K33211335 dextromethorphan Glutamate receptor antagonist

7688 -0.7 cp BRD-A07000685 hydrocortisone Glucocorticoid receptor agonist

7689 -0.7 cp BRD-A21858158 praziquantel Anthelmintic

7678 -0.67 cp BRD-K85015012 NNC-05-2090 GAT inhibitor

7679 -0.67 cp BRD-K62310379 fluticasone Glucocorticoid receptor agonist

7680 -0.67 cp BRD-K83508485 FK-888 Tachykinin antagonist

7681 -0.67 cp BRD-K64755930 etazolate Phosphodiesterase inhibitor

7682 -0.67 cp BRD-K15563106 phloretin Sodium/glucose cotransporter inhibitor

7675 -0.63 cp BRD-M72442222 vicriviroc CC chemokine receptor antagonist

7676 -0.63 cp BRD-U94846492 quinine Hemozoin biocrystallization inhibitor

7677 -0.63 cp BRD-A48720949 testosterone androgen receptor agonist

7669 -0.6 cp BRD-K06208435 YS-035 Calcium channel blocker

7670 -0.6 cp BRD-A84174393 meloxicam Cyclooxygenase inhibitor

7673 -0.6 cp BRD-K49865102 PD-0325901 MEK inhibitor

7662 -0.56 cp BRD-K55591206 epigallocatechin Nitric oxide synthase inhibitor

7663 -0.56 cp BRD-K22010301 JLK-6 Gamma secretase inhibitor

7664 -0.56 cp BRD-K17497770 butein EGFR inhibitor

7667 -0.56 cp BRD-K47639036 flavoxate Acetylcholine receptor antagonist

7659 -0.54 cp BRD-K05737787 isoeugenol Nitric oxide production inhibitor

7655 -0.53 cp BRD-A39415247 norethisterone Progesterone receptor agonist

7656 -0.53 cp BRD-K70914287 BIBX-1382 EGFR inhibitor

7657 -0.53 cp BRD-K36862742 hydroflumethiazide Sodium/potassium/chloride transporter inhibitor

7658 -0.53 cp BRD-K20755323 SA-792728 Sphingosine kinase inhibitor

7654 -0.52 cp BRD-K32311154 nifekalant Potassium channel blocker

7646 -0.49 cp BRD-K84085265 CG-930 JNK inhibitor

7647 -0.49 cp BRD-K33483813 actarit Interleukin receptor agonist

7648 -0.49 cp BRD-K78599730 manumycin-a Farnesyltransferase inhibitor

7649 -0.49 cp BRD-A90515964 guaifenesin Expectorant

7650 -0.49 cp BRD-K92778217 mefenamic-acid Cyclooxygenase inhibitor

7651 -0.49 cp BRD-K26657438 imiquimod TLR agonist

7639 -0.46 cp BRD-K52172416 anastrozole Aromatase inhibitor

7640 -0.46 cp BRD-K62996583 lidoflazine Calcium channel blocker

7641 -0.46 cp BRD-K43330982 JTE-013 Lysophospholipid receptor antagonist

7642 -0.46 cp BRD-K39339537 epirizole Cyclooxygenase inhibitor

7643 -0.46 cp BRD-A78942461 ICI-118551 Adrenergic receptor antagonist

7644 -0.46 cp BRD-A00993607 alprenolol Adrenergic receptor antagonist

7645 -0.46 cp BRD-K05977355 fluconazole Sterol demethylase inhibitor

7637 -0.45 cp BRD-K66615216 moxifloxacin Bacterial DNA gyrase inhibitor

7636 -0.42 cp BRD-K51677086 erythromycin NFkB pathway inhibitor

7626 -0.39 cp BRD-K20338176 cefaclor Bacterial cell wall synthesis inhibitor

7627 -0.39 cp BRD-K34154330 tracazolate GABA receptor modulator

7628 -0.39 cp BRD-K04170657 psoromic-acid Ras GTPase inhibitor

7629 -0.39 cp BRD-K17008822 BD-1008 Sigma receptor antagonist

7630 -0.39 cp BRD-K40887525 ritanserin Serotonin receptor antagonist

7631 -0.39 cp BRD-K52313696 tacedinaline HDAC inhibitor

7632 -0.39 cp BRD-A42759514 ornidazole Antiprotozoal

7633 -0.39 cp BRD-A44008656 doxylamine Histamine receptor antagonist

7634 -0.39 cp BRD-K07303502 arachidonyl-trifluoro-methane Cytosolic phospholipase inhibitor

7635 -0.39 cp BRD-K16554956 PTB1 AMPK activator

7623 -0.37 cp BRD-K62858456 lomerizine Calcium channel blocker

7616 -0.35 cp BRD-K59597909 phenothiazine Dopamine receptor antagonist

7617 -0.35 cp BRD-A62809825 thapsigargin ATPase inhibitor

7618 -0.35 cp BRD-K16664969 GTP-14564 FLT3 inhibitor

7619 -0.35 cp BRD-K54704028 BAY-36-7620 Glutamate receptor antagonist

7620 -0.35 cp BRD-K19227686 phenolphthalein Indicator dye

7621 -0.35 cp BRD-A71203467 l-stepholidine Dopamine receptor antagonist

7622 -0.35 cp BRD-K30020243 aliskiren Antihypertensive

7607 -0.32 cp BRD-A52282606 lacidipine Calcium channel blocker

7608 -0.32 cp BRD-K80672993 M2-PK-activator -666

7609 -0.32 cp BRD-K19533706 tranilast Angiogenesis inhibitor

7610 -0.32 cp BRD-K40992116 parachlorophenol Anti-infective

7611 -0.32 cp BRD-K33308633 INCA-6 Calcineurin inhibitor

7612 -0.32 cp BRD-A09472452 flecainide Sodium channel blocker

7613 -0.32 cp BRD-A65280694 molindone Dopamine receptor antagonist

7614 -0.32 cp BRD-A30435184 metergoline Dopamine receptor agonist

7603 -0.29 cp BRD-K61737877 VEGF-receptor-2-kinase-inhibitor-IV VEGFR inhibitor

7595 -0.28 cp BRD-A24381660 zeranol Estrogen receptor agonist

7596 -0.28 cp BRD-K31542390 mycophenolic-acid Dehydrogenase inhibitor

7597 -0.28 cp BRD-K59753853 MDL-29951 Glutamate receptor antagonist

7598 -0.28 cp BRD-A90799790 isradipine Calcium channel blocker

7599 -0.28 cp BRD-K48869804 icilin TRPV agonist

7600 -0.28 cp BRD-A04706586 bucladesine Adenosine receptor agonist

7601 -0.28 cp BRD-K06895174 cisapride Serotonin receptor agonist

7602 -0.28 cp BRD-K47323024 methapyrilene Histamine receptor antagonist

7594 -0.27 cp BRD-K72034655 peucedanin Apoptosis stimulant

7593 -0.26 cp BRD-A31521121 methocarbamol Muscle relaxant

7588 -0.25 cp BRD-A19633847 perhexiline Carnitine palmitoyltransferase inhibitor

7589 -0.25 cp BRD-K74195153 irsogladine Phosphodiesterase inhibitor

7590 -0.25 cp BRD-K76534306 enrofloxacin Bacterial DNA gyrase inhibitor

7591 -0.25 cp BRD-K57179821 crotamiton Antipruritic

7592 -0.25 cp BRD-K99595596 salsolinol Monoamine oxidase inhibitor

7583 -0.21 cp BRD-A52660433 tetrindole Monoamine oxidase inhibitor

7584 -0.21 cp BRD-K06980535 promazine Dopamine receptor antagonist

7585 -0.21 cp BRD-K63945320 dihydrosamidin Phospholipase inhibitor

7586 -0.21 cp BRD-K12994359 valdecoxib Cyclooxygenase inhibitor

7587 -0.21 cp BRD-A09925278 etilefrine Adrenergic receptor agonist

7576 -0.18 cp BRD-K44442813 pidotimod Interferon receptor agonist

7577 -0.18 cp BRD-K68437527 EMF-bca1-60 caspase inhibitor

7578 -0.18 cp BRD-K75641298 metoclopramide Dopamine receptor antagonist

7579 -0.18 cp BRD-A67862938 naftidrofuryl Adrenergic receptor antagonist

7580 -0.18 cp BRD-K89997465 chlorpromazine Dopamine receptor antagonist

7581 -0.18 cp BRD-A24514565 warfarin Vitamin K antagonist

7582 -0.18 cp BRD-A50764878 MDL-73005EF Serotonin receptor antagonist

7559 -0.14 cp BRD-A48261811 argatroban Thrombin inhibitor

7560 -0.14 cp BRD-K13926615 vardenafil Phosphodiesterase inhibitor

7561 -0.14 cp BRD-K77771411 moxonidine Imidazoline receptor agonist

7562 -0.14 cp BRD-A97730597 hexylcaine Sodium channel blocker

7563 -0.14 cp BRD-K46137903 prednicarbate Phospholipase activator

7564 -0.14 cp BRD-A40639672 ketorolac Cyclooxygenase inhibitor

7565 -0.14 cp BRD-A04553218 chlorphenamine Histamine receptor antagonist

7566 -0.14 cp BRD-A93424738 dexamethasone Glucocorticoid receptor agonist

7567 -0.14 cp BRD-A01593789 chlormadinone 5-alpha reductase inhibitor

7568 -0.14 cp BRD-K49049886 CGS-15943 Adenosine receptor antagonist

7569 -0.14 cp BRD-A92670106 tocainide Sodium channel blocker

7570 -0.14 cp BRD-K05395900 nicotine Acetylcholine receptor agonist

7571 -0.14 cp BRD-A07440155 labetalol Adrenergic receptor antagonist

7572 -0.14 cp BRD-K68143200 SA-792541 CDC inhibitor

7557 -0.13 cp BRD-K04414442 SB-222200 Tachykinin antagonist

7556 -0.12 cp BRD-K44353683 nateglinide Insulin secretagogue

7542 -0.11 cp BRD-K37516142 idebenone Calcium channel modulator

7543 -0.11 cp BRD-K33193182 methylnorlichexanthone Aurora kinase inhibitor

7544 -0.11 cp BRD-K00610438 altanserin Serotonin receptor antagonist

7545 -0.11 cp BRD-K64614248 salicin Anti-inflammatory

7546 -0.11 cp BRD-K25224017 pirenperone Serotonin receptor antagonist

7547 -0.11 cp BRD-K88741031 "methyl-2,5-dihydroxycinnamate" EGFR inhibitor

7548 -0.11 cp BRD-K49519092 immethridine Histamine receptor agonist

7549 -0.11 cp BRD-K33818169 GW-3965 LXR agonist

7550 -0.11 cp BRD-K77947974 fluspirilene Dopamine receptor antagonist

7551 -0.11 cp BRD-K53737926 amitriptyline Norepinephrine inhibitor

7552 -0.11 cp BRD-K97810537 beclometasone Glucocorticoid receptor agonist

7553 -0.11 cp BRD-K01292756 pimozide Dopamine receptor antagonist

7554 -0.11 cp BRD-A92585442 RU-28318 Cytochrome P450 inhibitor

7555 -0.11 cp BRD-A51714012 venlafaxine Adrenergic inhibitor

7540 -0.1 cp BRD-A41112154 oleanolic-acid G protein-coupled receptor agonist

7537 -0.09 cp BRD-K62959606 sphingosine Ceramidase inhibitor

7538 -0.09 cp BRD-A10303790 talampicillin Bacterial cell wall synthesis inhibitor

7535 -0.08 cp BRD-K11399644 phenformin AMPK activator

7536 -0.08 cp BRD-K73109821 diazoxide Potassium channel activator

7525 -0.07 cp BRD-K04466929 Merck60 HDAC inhibitor

7526 -0.07 cp BRD-A33168282 sotalol Adrenergic receptor antagonist

7527 -0.07 cp BRD-K82036761 sertraline Serotonin receptor antagonist

7528 -0.07 cp BRD-K25311561 KU-55933 ATM kinase inhibitor

7529 -0.07 cp BRD-K06388322 pramipexole Dopamine receptor agonist

7530 -0.07 cp BRD-K79131256 albendazole Anthelmintic

7531 -0.07 cp BRD-K67043667 altretamine DNA synthesis inhibitor

7532 -0.07 cp BRD-K08806317 timolol Adrenergic receptor antagonist

7533 -0.07 cp BRD-A42571354 cetirizine Histamine receptor antagonist

7534 -0.07 cp BRD-K03816923 rottlerin MAP kinase inhibitor

7520 -0.06 cp BRD-K62965247 tipifarnib-P2 farnesyltransferase inhibitor

7521 -0.06 cp BRD-K45435259 SCH-23390 Dopamine receptor antagonist

7522 -0.06 cp BRD-A26334849 propafenone Antiarrhythmic

7515 -0.05 cp BRD-K01493881 apigenin Casein kinase inhibitor

7507 -0.04 cp BRD-K09416995 lovastatin HMGCR inhibitor

7508 -0.04 cp BRD-K96799727 pifithrin-mu HSP inhibitor

7509 -0.04 cp BRD-K91290917 amodiaquine Histamine receptor agonist

7510 -0.04 cp BRD-K85119730 tolbutamide ATP channel blocker

7511 -0.04 cp BRD-K93461745 buspirone Serotonin receptor agonist

7488 -0.03 cp BRD-A92630576 trimebutine Opioid receptor agonist

7489 -0.03 cp BRD-K81169441 cerivastatin HMGCR inhibitor

7490 -0.03 cp BRD-A20697603 thiostrepton FOXM1 inhibitor

7491 -0.03 cp BRD-K11540476 EMF-BCA1-64 Caspase inhibitor

7492 -0.03 cp BRD-K04430056 7-nitroindazole nitric oxide synthase inhibitor

7493 -0.03 cp BRD-A34817987 itraconazole Cytochrome P450 inhibitor

7494 -0.03 cp BRD-K73999723 telmisartan Angiotensin receptor antagonist

7495 -0.03 cp BRD-A70649075 sulconazole Sterol demethylase inhibitor

7496 -0.03 cp BRD-K94441233 mevastatin HMGCR inhibitor

7497 -0.03 cp BRD-K32501161 vanoxerine Dopamine uptake inhibitor

7498 -0.03 cp BRD-K06221026 DUP-697 Cyclooxygenase inhibitor

7499 -0.03 cp BRD-K15025317 BAY-11-7821 NFkB pathway inhibitor

7500 -0.03 cp BRD-A29426959 carbinoxamine Histamine receptor antagonist

7501 -0.03 cp BRD-K39987650 bisacodyl Laxative

7502 -0.03 cp BRD-K72726508 arcyriaflavin-a CDK inhibitor

7503 -0.03 cp BRD-K61250553 loperamide Opioid receptor agonist

7504 -0.03 cp BRD-K80738081 resveratrol Cytochrome P450 inhibitor

7505 -0.03 cp BRD-K88789588 letrozole Aromatase inhibitor

7506 -0.03 cp BRD-K41731458 triclosan Enoyl-[acyl-carrier-protein] reductase [NADH] inhibitor

7467 -0.02 cp BRD-K73397362 purmorphamine Smoothened receptor agonist

7468 -0.02 cp BRD-K18816859 L-694247 Serotonin receptor agonist

7469 -0.02 cp BRD-K01095011 finasteride 5-alpha reductase inhibitor

7470 -0.02 cp BRD-K60770992 pergolide Dopamine receptor agonist

7471 -0.02 cp BRD-K28307902 flutamide Androgen receptor antagonist

7472 -0.02 cp BRD-K41713976 E-4031 Potassium channel blocker

7473 -0.02 cp BRD-A89175223 bisoprolol Adrenergic receptor antagonist

7474 -0.02 cp BRD-A10523515 GSK-429286A Rho associated kinase inhibitor

7475 -0.02 cp BRD-K81272440 dantrolene Calcium channel blocker

7476 -0.02 cp BRD-K02265150 amoxapine Norepinephrine reuptake inhibitor

7477 -0.02 cp BRD-K37289225 clozapine Dopamine receptor antagonist

7478 -0.02 cp BRD-K05396879 15-delta-prostaglandin-j2 PPAR receptor agonist

7479 -0.02 cp BRD-A66435872 HTMT Histamine receptor agonist

7480 -0.02 cp BRD-A01320529 salmeterol Adrenergic receptor agonist

7481 -0.02 cp BRD-K02637541 celecoxib Cyclooxygenase inhibitor

7482 -0.02 cp BRD-K78126613 menadione Mitochondrial DNA polymerase inhibitor

7483 -0.02 cp BRD-K98548675 parthenolide NFkB pathway inhibitor

7484 -0.02 cp BRD-K09778810 FGIN-1-27 Inositol monophosphatase inhibitor

7459 -0.01 cp BRD-A41451487 PK-11195 Benzodiazepine receptor antagonist

7460 -0.01 cp BRD-K90789829 nefazodone Adrenergic inhibitor

7461 -0.01 cp BRD-K28936863 ketotifen Histamine receptor agonist

7462 -0.01 cp BRD-K97158071 droperidol Dopamine receptor antagonist

7463 -0.01 cp BRD-K49111258 prazosin Adrenergic receptor antagonist

7464 -0.01 cp BRD-K32821942 azathioprine Dehydrogenase inhibitor

1060 0 cp BRD-K10466330 AVA Nucleophosmin inhibitor

1061 0 cp BRD-K67174588 toremifene Estrogen receptor antagonist

1062 0 cp BRD-K53665955 MK-5108 Aurora kinase inhibitor

1063 0 cp BRD-K79877282 PF-543 Sphingosine kinase inhibitor

1064 0 cp BRD-K61691971 avrainvillamide-analog-1 nucleophosmin inhibitor

1065 0 cp BRD-A74907996 equol Estrogen receptor agonist

1066 0 cp BRD-A83237092 fulvestrant Estrogen receptor antagonist

1067 0 cp BRD-K39569857 avrainvillamide-analog-3 nucleophosmin inhibitor

2022 0 cp BRD-K59773493 benzohydroxamic-acid Antifungal

2023 0 cp BRD-K69688083 mestinon Cholinesterase inhibitor

2024 0 cp BRD-K97799481 theophylline Adenosine receptor antagonist

2025 0 cp BRD-A15493168 tetracycline Bacterial 30S ribosomal subunit inhibitor

2026 0 cp BRD-K28667793 pyrazinamide Fatty acid synthase inhibitor

2027 0 cp BRD-K86873305 piperacillin Bacterial cell wall synthesis inhibitor

2028 0 cp BRD-K82216340 medroxyprogesterone progesterone receptor agonist

2029 0 cp BRD-A51182606 chloramphenicol Protein synthesis inhibitor

2030 0 cp BRD-K54529596 captopril ACE inhibitor

2031 0 cp BRD-K66766661 17-beta-estradiol Estrogen receptor agonist

2032 0 cp BRD-M41783010 acamprosate Glutamate receptor antagonist

2033 0 cp BRD-K90864987 cobalt(II)-chloride HSP inducer

2034 0 cp BRD-K88701661 dimercaptosuccinic-acid Chelating agent

2035 0 cp BRD-K83636919 entacapone Catechol O methyltransferase inhibitor

2036 0 cp BRD-K67977190 eprosartan Angiotensin receptor antagonist

2037 0 cp BRD-K63784565 BRD-K63784565 Topoisomerase inhibitor

2038 0 cp BRD-K60690191 MPEP Glutamate receptor antagonist

2039 0 cp BRD-K56450366 NSC-94258 Antineoplastic

2040 0 cp BRD-K37814297 acepromazine Dopamine receptor antagonist

2041 0 cp BRD-K20197062 SA-94315 Caspase inhibitor

2042 0 cp BRD-K17953061 staurosporine PKC inhibitor

2043 0 cp BRD-K15519488 CS-110266 Dopamine receptor agonist

2044 0 cp BRD-K06817181 BRD-K06817181 JAK inhibitor

2045 0 cp BRD-A87125127 3-matida Glutamate receptor antagonist

2046 0 cp BRD-A83892713 rifampicin RNA polymerase inhibitor

2047 0 cp BRD-A80383043 BRD-A80383043 Glutamate receptor agonist

2048 0 cp BRD-A79314293 cephalosporanic-acid Bacterial cell wall synthesis inhibitor

2049 0 cp BRD-A29731977 17-hydroxyprogesterone-caproate progesterone receptor agonist

2050 0 cp BRD-A20589515 dihydroxyphenylglycine Glutamate receptor agonist

2051 0 cp BRD-A15914070 4-hydroxy-2-nonenal Cytotoxic lipid peroxidation product

2052 0 cp BRD-A14985772 ascorbyl-palmitate antioxidant

2053 0 cp BRD-A09495397 bicuculline GABA receptor antagonist

2054 0 cp BRD-A98283014 calmidazolium Calcium channel blocker

2055 0 cp BRD-K29582115 ziprasidone Dopamine receptor antagonist

2056 0 cp BRD-K77390737 xanthohumol ATPase inhibitor

2057 0 cp BRD-K36627727 tamibarotene Retinoid receptor agonist

2058 0 cp BRD-U97083655 teicoplanin Bacterial cell wall synthesis inhibitor

2059 0 cp BRD-K15891719 tenofovir Reverse transcriptase inhibitor

2060 0 cp BRD-K66956375 oleoylethanolamide Cannabinoid receptor agonist

2061 0 cp BRD-K37720887 SB-525334 TGF beta receptor inhibitor

2062 0 cp BRD-K07736136 VX-702 p38 MAPK inhibitor

2063 0 cp BRD-K37130656 rivaroxaban Coagulation inhibitor

2064 0 cp BRD-K70511574 sunitinib PLK inhibitor

2065 0 cp BRD-K70557564 zosuquidar P-glycoprotein inhibitor

2066 0 cp BRD-K81783531 VX-222 HCV inhibitor

2067 0 cp BRD-K89014967 AS-703026 MEK inhibitor

2068 0 cp BRD-K84639753 safinamide Dopamine uptake inhibitor

2069 0 cp BRD-K91696562 orantinib FGFR inhibitor

2070 0 cp BRD-K91900765 VX-745 p38 MAPK inhibitor

2071 0 cp BRD-K73838513 cinacalcet Calcium channel activator

2072 0 cp BRD-K07265709 razoxane Chelating agent

2073 0 cp BRD-M16762496 PIK-75 DNA protein kinase inhibitor

2074 0 cp BRD-K17306061 aprepitant Tachykinin antagonist

2075 0 cp BRD-K99498722 NPI-2358 Tubulin inhibitor

2076 0 cp BRD-K83837640 JNJ-26854165 HDAC inhibitor

2077 0 cp BRD-A41692738 TGX-221 PI3K inhibitor

2078 0 cp BRD-K16485616 mocetinostat HDAC inhibitor

2079 0 cp BRD-K67578145 GDC-0879 RAF inhibitor

2080 0 cp BRD-K28428262 brivanib FGFR inhibitor

2081 0 cp BRD-K02965346 SU-11274 Hepatocyte growth factor receptor inhibitor

2082 0 cp BRD-K71035033 masitinib KIT inhibitor

2083 0 cp BRD-K74514084 pazopanib KIT inhibitor

2084 0 cp BRD-K50168500 canertinib EGFR inhibitor

2085 0 cp BRD-K99964838 bosutinib ABL inhibitor

2086 0 cp BRD-K99749624 linifanib PDGFR receptor inhibitor

2087 0 cp BRD-K33551950 radicicol HSP inhibitor

2088 0 cp BRD-K60460488 nelfinavir HIV protease inhibitor

2089 0 cp BRD-K46212057 voriconazole Cytochrome P450 inhibitor

2090 0 cp BRD-K83794624 pirarubicin Topoisomerase inhibitor

2091 0 cp BRD-K36529613 PU-H71 HSP inhibitor

2092 0 cp BRD-K55420858 mirin MRE11A exonuclease inhibitor

2093 0 cp BRD-K99451608 lopinavir HIV protease inhibitor

2094 0 cp BRD-K23192422 lestaurtinib FLT3 inhibitor

2095 0 cp BRD-A67748489 K3644 Kinesin-like spindle protein inhibitor

2096 0 cp BRD-K70401845 erlotinib EGFR inhibitor

2097 0 cp BRD-K78431006 crizotinib ALK inhibitor

2098 0 cp BRD-K82091397 SB-239063 p38 MAPK inhibitor

2099 0 cp BRD-K05804044 AZ-628 RAF inhibitor

2100 0 cp BRD-K64800655 PHA-793887 CDK inhibitor

2101 0 cp BRD-K01253243 SB-590885 RAF inhibitor

2102 0 cp BRD-K13566078 BMS-345541 IKK inhibitor

2103 0 cp BRD-K99545815 PF-562271 Focal adhesion kinase inhibitor

2104 0 cp BRD-K52751261 TAK-715 p38 MAPK inhibitor

2105 0 cp BRD-K81528515 nilotinib ABL inhibitor

2106 0 cp BRD-K86465814 HO-013 PPAR receptor agonist

2107 0 cp BRD-A80775386 hyperforin Cyclooxygenase inhibitor

2108 0 cp BRD-K47539947 tetradecylthioacetic-acid Lipid peroxidase inhibitor

2109 0 cp BRD-K61480498 epoxycholesterol LXR agonist

2110 0 cp BRD-A36707673 hydroxycholesterol LXR agonist

2111 0 cp BRD-A96799240 4-hydroxyretinoic-acid Retinoid receptor binder

2112 0 cp BRD-K62012036 acitretin Retinoid receptor agonist

2113 0 cp BRD-K98684188 GSK-0660 PPAR receptor antagonist

2114 0 cp BRD-K95402279 geranylgeraniol Farnesyltransferase inhibitor

2115 0 cp BRD-K79437791 acetyl-farnesyl-cysteine Inhibitor of methylation of endogenous isoprenylated proteins

2116 0 cp BRD-K50720187 flupirtine Glutamate receptor antagonist

2117 0 cp BRD-U08759356 EI-346-erlotinib-analog EGFR inhibitor

2118 0 cp BRD-K08115555 tyrphostin-AG-1288 TNF production inhibitor

2119 0 cp BRD-A18497530 5-iodotubercidin Adenosine kinase inhibitor

2120 0 cp BRD-K67506692 tyrphostin-AG-126 ERK1 and ERK2 phosphorylation inhibitor

2121 0 cp BRD-K32710582 EI-247 IGF-1 inhibitor

2122 0 cp BRD-K80725632 lavendustin-c EGFR inhibitor

2123 0 cp BRD-A74904029 EI-231 Casein kinase inhibitor

2124 0 cp BRD-K47943470 tyrphostin-51 EGFR inhibitor

2125 0 cp BRD-A55756846 H-7 PKA inhibitor

2126 0 cp BRD-K85985071 ellipticine Topoisomerase inhibitor

2127 0 cp BRD-K77625572 etomoxir Carnitine palmitoyltransferase inhibitor

2128 0 cp BRD-K13087974 "4,5-dianilinophthalimide" EGFR inhibitor

2129 0 cp BRD-K35128472 2-aminopurine Serine/threonine kinase inhibitor

2130 0 cp BRD-K32610195 androstenedione Cytochrome P450 inhibitor

2131 0 cp BRD-K43880410 pregnenolone glutamate receptor modulator

2132 0 cp BRD-U66370498 androstanol CAR antagonist

2133 0 cp BRD-K24656285 farnesol FXR agonist

2134 0 cp BRD-K20986251 lithocholic-acid FXR antagonist

2135 0 cp BRD-K30743633 TCPOBOP CAR agonist

2136 0 cp BRD-K81062487 taurocholic-acid Bile acid

2137 0 cp BRD-K18135438 chenodeoxycholic-acid 11-beta-HSD1 inhibitor

2138 0 cp BRD-K41170226 deoxycholic-acid G protein-coupled receptor agonist

2139 0 cp BRD-K43164539 cholic-acid Bile acid

2140 0 cp BRD-K65331431 retinyl vitamin analog

2141 0 cp BRD-K13927029 retinol Retinoid receptor ligand

2142 0 cp BRD-K73982490 BI-78D3 JNK inhibitor

2143 0 cp BRD-K57926513 tyrphostin-AG-1295 PDGFR receptor inhibitor

2144 0 cp BRD-A01317026 "7,8-dihydro-L-biopterin" Dihydroneopterin aldolase inhibitor

2145 0 cp BRD-A84327315 calcitriol Vitamin D receptor agonist

2146 0 cp BRD-K14880289 GW-501516 PPAR receptor agonist

2147 0 cp BRD-K39944607 ochratoxin-a Phenylalanyl tRNA synthetase inhibitor

2148 0 cp BRD-A80960055 celastrol Anti-inflammatory

2149 0 cp BRD-K04923131 GSK-3-inhibitor-IX Glycogen synthase kinase inhibitor

2150 0 cp BRD-A52193669 withaferin-a IKK inhibitor

2151 0 cp BRD-A11702965 chromomycin-a3 DNA binding agent

2152 0 cp BRD-A80574334 oxalomalic-acid Isocitrate dehydrogenase inhibitor

2153 0 cp BRD-K22631935 neurodazine Neurogenesis of non-pluripotent C2C12 myoblast inducer

2154 0 cp BRD-K88551539 CAY-10585 HIF modulator

2155 0 cp BRD-K63915849 AS-604850 PI3K inhibitor

2156 0 cp BRD-K14681867 somatostatin Somatostatin receptor agonist

2157 0 cp BRD-U73238814 QL-XI-92 DDR1 inhibitor

2158 0 cp BRD-U51951544 ZG-10 JNK inhibitor

2159 0 cp BRD-U44618005 WH-4023 SRC inhibitor

2160 0 cp BRD-U25771771 WZ-4-145 EGFR inhibitor

2161 0 cp BRD-K72420232 WZ-4002 EGFR inhibitor

2162 0 cp BRD-K68174511 torin-2 MTOR inhibitor

2163 0 cp BRD-K64857848 XMD-885 Leucine rich repeat kinase inhibitor

2164 0 cp BRD-K40175214 torin-1 MTOR inhibitor

2165 0 cp BRD-K38615104 A-443644 AKT inhibitor

2166 0 cp BRD-K24859147 KIN001-242 Protein kinase inhibitor

2167 0 cp BRD-K19220233 JNK-9L JNK inhibitor

2168 0 cp BRD-A60245366 AS-601245 JNK inhibitor

2169 0 cp BRD-A68589262 troxipide Glucosamine synthetase stimulant

2170 0 cp BRD-K04196797 oxcarbazepine Sodium channel blocker

2171 0 cp BRD-A61856038 tremulacin Lipoxygenase inhibitor

2172 0 cp BRD-K04412738 tramadol Norepinephrine reuptake inhibitor

2173 0 cp BRD-K54316499 tolterodine Acetylcholine receptor antagonist

2174 0 cp BRD-K89125793 tinidazole Antiprotozoal

2175 0 cp BRD-K10467831 tibolone Androgen receptor agonist

2176 0 cp BRD-K60160658 tiagabine GABA uptake inhibitor

2177 0 cp BRD-K94887716 TFMPP Serotonin receptor agonist

2178 0 cp BRD-K32744045 disulfiram Aldehyde dehydrogenase inhibitor

2179 0 cp BRD-K94649603 taxifolin Opioid receptor antagonist

2180 0 cp BRD-A04327189 synephrine Adrenergic receptor agonist

2181 0 cp BRD-A72441487 stiripentol GABA uptake inhibitor

2182 0 cp BRD-A23359898 sibutramine Serotonin reuptake inhibitor

2183 0 cp BRD-K91733562 secoisolariciresinol Antioxidant

2184 0 cp BRD-A27489425 rolitetracycline Bacterial 30S ribosomal subunit inhibitor

2185 0 cp BRD-K70490179 rimcazole Sigma receptor antagonist

2186 0 cp BRD-K89708791 rifaximin RNA synthesis inhibitor

2187 0 cp BRD-K30563334 rifabutin Protein synthesis inhibitor

2188 0 cp BRD-A56245458 reichstein Androgen receptor antagonist

2189 0 cp BRD-K92870997 pterostilbene Cyclooxygenase inhibitor

2190 0 cp BRD-K43236057 piceid ICAM1 inhibitor

2191 0 cp BRD-A37052580 physostigmine Acetylcholinesterase inhibitor

2192 0 cp BRD-K85503079 perospirone Dopamine receptor antagonist

2193 0 cp BRD-K55034111 pefloxacin Bacterial DNA gyrase inhibitor

2194 0 cp BRD-A84134924 pancuronium Acetylcholine receptor antagonist

2195 0 cp BRD-K08924299 palonosetron Serotonin receptor antagonist

2196 0 cp BRD-A23637604 oxymetholone Androgen receptor agonist

2197 0 cp BRD-K23369905 oxiconazole Bacterial cell wall synthesis inhibitor

2198 0 cp BRD-K83144676 olmesartan Angiotensin antagonist

2199 0 cp BRD-K18895904 olanzapine Dopamine receptor antagonist

2200 0 cp BRD-A24543851 nornicotine Acetylcholine receptor agonist

2201 0 cp BRD-A12560204 nitrendipine Calcium channel blocker

2202 0 cp BRD-K57930253 nitrazepam Benzodiazepine receptor agonist

2203 0 cp BRD-K96471533 nitazoxanide Pyruvate ferredoxin oxidoreductase inhibitor

2204 0 cp BRD-K04710043 hexamethylenebisacetamide AKT inhibitor

2205 0 cp BRD-A39052811 mosapride Serotonin receptor agonist

2206 0 cp BRD-K02227374 milnacipran Serotonin reuptake inhibitor

2207 0 cp BRD-K00532621 midazolam Benzodiazepine receptor agonist

2208 0 cp BRD-K52020312 metronidazole DNA inhibitor

2209 0 cp BRD-K88679075 methandriol Androgenic steroid

2210 0 cp BRD-A11990600 lorazepam Benzodiazepine receptor agonist

2211 0 cp BRD-K82795137 loratadine Histamine receptor antagonist

2212 0 cp BRD-A02990301 lofexidine Adrenergic receptor agonist

2213 0 cp BRD-K82147103 lofepramine Norepinephrine reuptake inhibitor

2214 0 cp BRD-K66206289 lobeline Acetylcholine receptor antagonist

2215 0 cp BRD-K51671335 sulpiride Dopamine receptor antagonist

2216 0 cp BRD-A49172652 lansoprazole ATPase inhibitor

2217 0 cp BRD-K22227508 targinine Nitric oxide synthase inhibitor

2218 0 cp BRD-K30097969 pitavastatin HMGCR inhibitor

2219 0 cp BRD-K73991644 isoquercetin Aldose reductase inhibitor

2220 0 cp BRD-K93618743 ipriflavone Bone resorption inhibitor

2221 0 cp BRD-K01649396 indatraline Norepinephrine transporter inhibitor

2222 0 cp BRD-K69650333 idarubicin Topoisomerase inhibitor

2223 0 cp BRD-K84955386 hyperoside Glucosidase inhibitor

2224 0 cp BRD-K59570838 homoveratrylamine Dopamine analog

2225 0 cp BRD-K33312228 halometasone Glucocorticoid receptor agonist

2226 0 cp BRD-K10860596 granisetron Serotonin receptor antagonist

2227 0 cp BRD-A79903587 tegafur Thymidylate synthase inhibitor

2228 0 cp BRD-K84566043 fenpiverinium Acetylcholine receptor antagonist

2229 0 cp BRD-K00673382 famotidine Histamine receptor antagonist

2230 0 cp BRD-K99447003 enalaprilat ACE inhibitor

2231 0 cp BRD-K28029915 dolasetron Serotonin receptor antagonist

2232 0 cp BRD-K86887724 dofetilide Potassium channel blocker

2233 0 cp BRD-K63265447 docetaxel Tubulin inhibitor

2234 0 cp BRD-K13664374 dichloroacetic-acid Pyruvate dehydrogenase kinase inhibitor

2235 0 cp BRD-K39462424 dexchlorpheniramine Histamine receptor antagonist

2236 0 cp BRD-K22947005 dexbrompheniramine Histamine receptor antagonist

2237 0 cp BRD-K30697463 desoximetasone Glucocorticoid receptor agonist

2238 0 cp BRD-K90976994 dehydrocholic-acid Bile acid

2239 0 cp BRD-K73589401 corticosterone mineralocorticoid receptor agonist

2240 0 cp BRD-K15916496 clotrimazole Cytochrome P450 inhibitor

2241 0 cp BRD-K86595100 chlordiazepoxide Benzodiazepine receptor agonist

2242 0 cp BRD-K48932581 cetraxate Mucus protecting agent

2243 0 cp BRD-K52735702 cefdinir Bacterial cell wall synthesis inhibitor

2244 0 cp BRD-M47937986 cefatrizine Bacterial cell wall synthesis inhibitor

2245 0 cp BRD-K11630072 carmofur Thymidylate synthase inhibitor

2246 0 cp BRD-K16336526 capsaicin TRPV agonist

2247 0 cp BRD-A42423104 benproperine Antitussive

2248 0 cp BRD-A35519318 benidipine Calcium channel blocker

2249 0 cp BRD-K85030058 benactyzine Acetylcholine receptor antagonist

2250 0 cp BRD-A17448384 beclometasone Glucocorticoid receptor agonist

2251 0 cp BRD-A68888262 azelastine Histamine receptor antagonist

2252 0 cp BRD-A75935363 atracurium Acetylcholine receptor antagonist

2253 0 cp BRD-K39621635 artemether Antimalarial

2254 0 cp BRD-A39172021 ampiroxicam Cyclooxygenase inhibitor

2255 0 cp BRD-A64297288 amlodipine Calcium channel blocker

2256 0 cp BRD-K29530284 amlexanox Histamine receptor modulator

2257 0 cp BRD-A16444946 acarbose Glucosidase inhibitor

2258 0 cp BRD-A59985574 topotecan Topoisomerase inhibitor

2259 0 cp BRD-K48935217 epothilone Microtubule inhibitor

2260 0 cp BRD-A25569250 KI-16425 Lysophosphatidic acid receptor antagonist

2261 0 cp BRD-K11636097 JNJ-7706621 CDK inhibitor

2262 0 cp BRD-K16621777 enobosarm Androgen receptor modulator

2263 0 cp BRD-K61192372 capecitabine DNA synthesis inhibitor

2264 0 cp BRD-K63923597 barasertib Aurora kinase inhibitor

2265 0 cp BRD-K82164249 andarine Androgen receptor modulator

2266 0 cp BRD-K04833372 GSK-1904529A IGF-1 inhibitor

2267 0 cp BRD-K99616396 motesanib KIT inhibitor

2268 0 cp BRD-K05926469 lenalidomide Antineoplastic

2269 0 cp BRD-M07438658 lapatinib EGFR inhibitor

2270 0 cp BRD-K29905972 axitinib PDGFR receptor inhibitor

2271 0 cp BRD-K49810818 sorafenib FLT3 inhibitor

2272 0 cp BRD-K28120222 parthenolide NFkB pathway inhibitor

2273 0 cp BRD-K47761761 PD-168077 Dopamine receptor agonist

2274 0 cp BRD-K21971034 OM-137 Aurora kinase inhibitor

2275 0 cp BRD-K13390322 AT-7519 CDK inhibitor

2276 0 cp BRD-K79090631 CGP-60474 CDK inhibitor

2277 0 cp BRD-K76908866 CP-724714 EGFR inhibitor

2278 0 cp BRD-K49328571 dasatinib BCR-ABL kinase inhibitor

2279 0 cp BRD-K15402119 huperzine-a Acetylcholinesterase inhibitor

2280 0 cp BRD-K14791739 fluticasone Glucocorticoid receptor agonist

2281 0 cp BRD-K14550461 doxercalciferol Vitamin D receptor agonist

2282 0 cp BRD-K82357231 desloratadine Histamine receptor antagonist

2283 0 cp BRD-K64310881 MW-STK33-3B Potassium channel activator

2284 0 cp BRD-A74771556 nikkomycin Chitin inhibitor

2285 0 cp BRD-A61858259 CAY-10415 Insulin sensitizer

2286 0 cp BRD-A00520476 otenzepad Acetylcholine receptor antagonist

2287 0 cp BRD-K56745457 azauridine Antiviral

2288 0 cp BRD-K51066026 aminoindazole Ionophore

2289 0 cp BRD-K49372556 mofezolac Cyclooxygenase inhibitor

2290 0 cp BRD-K89626439 sirolimus MTOR inhibitor

2291 0 cp BRD-K30197592 5-methoxytryptamine Serotonin receptor agonist

2292 0 cp BRD-K82143716 flucytosine Antifungal

2293 0 cp BRD-K28849549 mesalazine Cyclooxygenase inhibitor

2294 0 cp BRD-K92413528 thiazolidinecarboxylic-acid Reducing agent

2295 0 cp BRD-K09485525 GANT-61 GLI antagonist

2296 0 cp BRD-K06878038 deferiprone Chelating agent

2297 0 cp BRD-K51941867 LM-1685 Cyclooxygenase inhibitor

2298 0 cp BRD-K99063460 didanosine Nucleoside reverse transcriptase inhibitor

2299 0 cp BRD-K66896231 BRD-K66896231 Acetylcholinesterase inhibitor

2300 0 cp BRD-K40619305 larixinic-acid Compound that interacts with metal centers

2301 0 cp BRD-A67438293 treprostinil Prostacyclin analog

2302 0 cp BRD-K54987996 CAY-10578 Casein kinase inhibitor

2303 0 cp BRD-K87573634 propylpyrazole Estrogen receptor agonist

2304 0 cp BRD-U82589721 HG-5-113-01 Protein kinase inhibitor

2305 0 cp BRD-U68942961 JW-7-24-1 LCK Inhibitor

2306 0 cp BRD-U44700465 HG-5-88-01 Protein kinase inhibitor

2307 0 cp BRD-U37049823 HG-6-64-01 RAF inhibitor

2308 0 cp BRD-K72264770 QW-BI-011 Histone lysine methyltransferase inhibitor

2309 0 cp BRD-K65910366 KUC103904N -666

2310 0 cp BRD-K64835161 BRD-K64835161 -666

2311 0 cp BRD-K61217870 n-(3-acetamidophenyl)-3-chlorobenzamide Glutamate receptor antagonist

2312 0 cp BRD-K48115423 2-(4-methoxybenzylthio)-6-methylpyrimidin-4-ol Matrix metalloprotease inhibitor

2313 0 cp BRD-K47278471 diphenhydramine Histamine receptor antagonist

2314 0 cp BRD-K34508425 KUC103898N -666

2315 0 cp BRD-K29113274 ketoconazole Sterol demethylase inhibitor

2316 0 cp BRD-K22828899 TUL-XXI039 Serine/threonine kinase inhibitor

2317 0 cp BRD-K94144010 cotinine Nicotine metabolite

2318 0 cp BRD-K88304388 dextrorphan Glutamate receptor antagonist

2319 0 cp BRD-A22256192 terazosin Adrenergic receptor antagonist

2320 0 cp BRD-K22878149 SB-205607 Delta 1 opioid receptor agonist

2321 0 cp BRD-K28346421 rifapentine RNA polymerase inhibitor

2322 0 cp BRD-K87024524 phenelzine Monoamine oxidase inhibitor

2323 0 cp BRD-A84702196 penicillin Bacterial cell wall synthesis inhibitor

2324 0 cp BRD-K02992638 lamivudine Nucleoside reverse transcriptase inhibitor

2325 0 cp BRD-A74500471 ethambutol Bacterial cell wall synthesis inhibitor

2326 0 cp BRD-A67981824 cefotaxime Bacterial cell wall synthesis inhibitor

2327 0 cp BRD-K59633790 VU-0420363-1 SARS coronavirus 3C-like protease inhibitor

2328 0 cp BRD-K76907295 VU-0418947-2 HIF modulator

2329 0 cp BRD-A52172093 VU-0413807-2 Calcium channel blocker

2330 0 cp BRD-K84141129 VU-0400193-3 Glutamate receptor modulator

2331 0 cp BRD-A57457122 VU-0400071-3 Glutamate receptor modulator

2332 0 cp BRD-K55703048 latrepirdine Glutamate receptor antagonist

2333 0 cp BRD-K43149758 myricetin Androgen receptor agonist

2334 0 cp BRD-K36153907 KU-C103885 Cystic fibrosis transmembrane conductance regulator inhibitor

2335 0 cp BRD-A81402010 KU-C103443N CDC inhibitor

2336 0 cp BRD-A82096673 KU-C103428N CDC inhibitor

2337 0 cp BRD-K08219523 5-nonyloxytryptamine Serotonin receptor agonist

2338 0 cp BRD-A71657825 2-(biphenyl-4-ylsulfonamido)pentanedioic-acid Matrix metalloprotease inhibitor

2339 0 cp BRD-K64610608 EMF-bca1-57 caspase inhibitor

2340 0 cp BRD-K51302260 KU-C103871 GSP agonist

2341 0 cp BRD-A01145011 zebularine DNA methyltransferase inhibitor

2342 0 cp BRD-A43331270 niguldipine Calcium channel blocker

2343 0 cp BRD-A70449690 forskolin Adenylyl cyclase activator

2344 0 cp BRD-K61314889 IWR-1-ENDO PARP inhibitor

2345 0 cp BRD-A28105619 cucurbitacin-i JAK inhibitor

2346 0 cp BRD-A79465854 auranofin NFkB pathway inhibitor

2347 0 cp BRD-A47829399 artesunate DNA synthesis inhibitor

2348 0 cp BRD-K63195589 tipifarnib Farnesyltransferase inhibitor

2349 0 cp BRD-K60623809 SU-11652 Tyrosine kinase inhibitor

2350 0 cp BRD-K08417745 SID-26681509 Cathepsin inhibitor

2351 0 cp BRD-K02526760 QS-11 ARFGAP inhibitor

2352 0 cp BRD-A73680854 PT-630 Dipeptidyl peptidase inhibitor

2353 0 cp BRD-K49456190 prima-1-met thioredoxin inhibitor

2354 0 cp BRD-K30677119 PP-30 RAF inhibitor

2355 0 cp BRD-K67868012 PI-103 MTOR inhibitor

2356 0 cp BRD-K56700933 phenethyl-isothiocyanate Antineoplastic

2357 0 cp BRD-K36324071 NF-449 Purinergic receptor antagonist

2358 0 cp BRD-K78659596 MLN-2238 Proteasome inhibitor

2359 0 cp BRD-K16406336 methylene-blue Guanylyl cyclase inhibitor

2360 0 cp BRD-A04352665 maraviroc CC chemokine receptor antagonist

2361 0 cp BRD-K19796430 erismodegib Smoothened receptor antagonist

2362 0 cp BRD-A58955223 sulforaphane Antineoplastic

2363 0 cp BRD-A02481876 importazole Importin-beta transport receptor inhibitor

2364 0 cp BRD-K64451768 GANT-58 GLI antagonist

2365 0 cp BRD-K75532464 FTI-276 Farnesyltransferase inhibitor

2366 0 cp BRD-K40892394 AR-C133057XX Nitric oxide synthase inhibitor

2367 0 cp BRD-K74402642 NSC-632839 Ubiquitin specific protease inhibitor

2368 0 cp BRD-K01779529 fluoropyruvate Pyruvate dehydrogenase kinase inhibitor

2369 0 cp BRD-K83289131 CAY-10618 NAMPT inhibitor

2370 0 cp BRD-K48923948 BMS-641988 Androgen receptor antagonist

2371 0 cp BRD-K02950022 BMS-299897 Gamma secretase inhibitor

2372 0 cp BRD-K28296557 AKT-inhibitor-IV AKT inhibitor

2373 0 cp BRD-K02113016 olaparib PARP inhibitor

2374 0 cp BRD-K01192156 tyrphostin-AG-112 Protein tyrosine kinase inhibitor

2375 0 cp BRD-K87142802 veliparib PARP inhibitor

2376 0 cp BRD-K61829047 7b-cis Exportin antagonist

2377 0 cp BRD-K59962020 CHEMBL-374350 NFkB pathway inhibitor

2378 0 cp BRD-A20131130 "2',5'-dideoxyadenosine" Adenylyl cyclase inhibitor

2379 0 cp BRD-K73319509 PF-04217903 c-Met inhibitor

2380 0 cp BRD-K60219430 serdemetan MDM inhibitor

2381 0 cp BRD-K17140735 SCH-79797 Proteasome inhibitor

2382 0 cp BRD-A56359832 zileuton Leukotriene inhibitor

2383 0 cp BRD-A36630025 SN-38 Topoisomerase inhibitor

2384 0 cp BRD-K17705806 JTC-801 Opioid receptor antagonist

2385 0 cp BRD-K41260949 valproic-acid HDAC inhibitor

2386 0 cp BRD-K43797669 genistein Tyrosine kinase inhibitor

2387 0 cp BRD-K54708045 nTZDpa PPAR receptor agonist

2388 0 cp BRD-K36965586 m-chlorophenylbiguanide Serotonin receptor agonist

2389 0 cp BRD-K09635314 M-3M3FBS phospholipase activator

2390 0 cp BRD-A73605923 mocimycin Protein synthesis inhibitor

2391 0 cp BRD-A65550283 ginsenoside Steroid hormone receptor agonist

2392 0 cp BRD-A31801025 formestane Aromatase inhibitor

2393 0 cp BRD-A06726973 dibutyrylcyclic-gmp cGMP analog

2394 0 cp BRD-K97509413 coumestrol Estrogen receptor agonist

2395 0 cp BRD-K70487031 flupentixol Dopamine receptor antagonist

2396 0 cp BRD-K26134695 calpeptin Calpain inhibitor

2397 0 cp BRD-A26097136 bulleyaconitine-a Non-opiod analgesic

2398 0 cp BRD-A41301928 bongkrek-acid "Mitochondrial ADP, ATP translocase inhibitor"

2399 0 cp BRD-K28761384 zuclopenthixol Dopamine receptor antagonist

2400 0 cp BRD-K08996725 zolantidine Histamine receptor antagonist

2401 0 cp BRD-K92446736 zatebradine HCN channel blocker

2402 0 cp BRD-K37561857 zardaverine Phosphodiesterase inhibitor

2403 0 cp BRD-K12516989 zaprinast Phosphodiesterase inhibitor

2404 0 cp BRD-K67831364 ZM-323881 VEGFR inhibitor

2405 0 cp BRD-K19605405 ZM-241385 Adenosine receptor antagonist

2406 0 cp BRD-K68392338 ZK-93426 Benzodiazepine receptor antagonist

2407 0 cp BRD-K33882852 ZK-93423 Benzodiazepine receptor agonist

2408 0 cp BRD-K18678457 ZD-7288 HCN channel blocker

2409 0 cp BRD-K64157027 ZD-2079 Adrenergic receptor agonist

2410 0 cp BRD-K60174629 z-prolyl-prolinal Prolyl endopeptidase inhibitor

2411 0 cp BRD-K15935639 z-leu3-VS Proteasome inhibitor

2412 0 cp BRD-K12932420 YM-976 Phosphodiesterase inhibitor

2413 0 cp BRD-K60476892 YC-1 Guanylyl cyclase activator

2414 0 cp BRD-K88358234 xaliproden Serotonin receptor agonist

2415 0 cp BRD-K21565985 xylazine Adrenergic receptor agonist

2416 0 cp BRD-A20968261 WAY-213613 Glutamate inhibitor

2417 0 cp BRD-K68341547 W-9 Calmodulin antagonist

2418 0 cp BRD-K90259198 W-7 Calmodulin antagonist

2419 0 cp BRD-K45068323 W-13 Calmodulin antagonist

2420 0 cp BRD-K40902647 vincamine Adrenergic receptor antagonist

2421 0 cp BRD-K62206109 VUF-5681 Histamine receptor antagonist

2422 0 cp BRD-K60923938 veratridine Sodium channel activator

2423 0 cp BRD-K45158365 valsartan Angiotensin receptor antagonist

2424 0 cp BRD-K50325075 UCL-2077 Slow after hyperpolarization channel blocker

2425 0 cp BRD-A14574269 UB-165 Acetylcholine receptor agonist

2426 0 cp BRD-K18757346 U-46619 Thromboxane receptor agonist

2427 0 cp BRD-A81795050 U-18666A Oxidosqualene cyclase inhibitor

2428 0 cp BRD-K17415526 tyrphostin-AG-835 Protein tyrosine kinase inhibitor

2429 0 cp BRD-K02607075 tubocurarine Acetylcholine receptor antagonist

2430 0 cp BRD-K08619838 tremorine Acetylcholine receptor agonist

2431 0 cp BRD-K95763993 trapidil PDGFR receptor inhibitor

2432 0 cp BRD-K69837166 trap-101 Nociceptin/orphanin FQ (NOP) receptor antagonist

2433 0 cp BRD-K20141153 atomoxetine Norepinephrine transporter inhibitor

2434 0 cp BRD-A87479750 tenidap Cyclooxygenase inhibitor

2435 0 cp BRD-A98378129 talniflumate Cyclooxygenase inhibitor

2436 0 cp BRD-K11158509 tyrphostin-B44 EGFR inhibitor

2437 0 cp BRD-K96778649 tyrphostin-47 EGFR inhibitor

2438 0 cp BRD-K60184833 tyrphostin-46 Tyrosine kinase inhibitor

2439 0 cp BRD-A02176148 tubaic-acid Mitochondrial complex I inhibitor

2440 0 cp BRD-A01295252 trans-7-hydroxy-pipat Dopamine receptor ligand

2441 0 cp BRD-K45988865 tetramethylsilane Internal standard for NMR spectroscopy

2442 0 cp BRD-A31195449 TCB2 Serotonin receptor agonist

2443 0 cp BRD-K67352070 TC-2559 Acetylcholine receptor agonist

2444 0 cp BRD-K97118047 "4,5,6,7-tetrabromobenzotriazole" Casein kinase inhibitor

2445 0 cp BRD-K25186396 tangeritin Cell cycle inhibitor

2446 0 cp BRD-K62221994 T-98475 Gonadotropin releasing factor hormone receptor antagonist

2447 0 cp BRD-K23383398 T-0901317 LXR agonist

2448 0 cp BRD-K14200658 syrosingopine Vesicular monoamine transporter inhibitor

2449 0 cp BRD-K30189597 SYK-inhibitor SYK inhibitor

2450 0 cp BRD-K68332390 ponalrestat Aldose reductase inhibitor

2451 0 cp BRD-K97330509 SRC-kinase-inhibitor-II SRC inhibitor

2452 0 cp BRD-K70881766 solanine Acetylcholinesterase inhibitor

2453 0 cp BRD-K25741894 skimmianine Acetylcholinesterase inhibitor

2454 0 cp BRD-K38449220 seneciphylline Cytochrome P450 inhibitor

2455 0 cp BRD-A25775766 securinine GABA receptor antagonist

2456 0 cp BRD-A49906757 scopolamine Acetylcholine receptor antagonist

2457 0 cp BRD-K09963420 saquinavir HIV protease inhibitor

2458 0 cp BRD-A24122750 saclofen GABA receptor antagonist

2459 0 cp BRD-A10715913 sulpiride Dopamine receptor antagonist

2460 0 cp BRD-K97354755 SU-6656 SRC inhibitor

2461 0 cp BRD-K20287671 SU-4312 PDGFR receptor inhibitor

2462 0 cp BRD-K18905250 ST-91 Adrenergic receptor agonist

2463 0 cp BRD-K72895815 SSR-69071 Leukocyte elastase inhibitor

2464 0 cp BRD-K19309090 SR-95639A Acetylcholine receptor agonist

2465 0 cp BRD-K09397065 SR-57227A Serotonin receptor agonist

2466 0 cp BRD-K35629949 SR-27897 CCK receptor antagonist

2467 0 cp BRD-K91243525 SR-142948 Neurotensin receptor antagonist

2468 0 cp BRD-A56987319 SQ-22536 Adenylyl cyclase inhibitor

2469 0 cp BRD-K96809896 SKF-86002 p38 MAPK inhibitor

2470 0 cp BRD-A09828896 SKF-81297 Dopamine receptor agonist

2471 0 cp BRD-A64227845 SKF-77434 Dopamine receptor agonist

2472 0 cp BRD-K73824630 skatole Thrombin inhibitor

2473 0 cp BRD-K84996949 sinensetin Cyclooxygenase inhibitor

2474 0 cp BRD-K98157055 SIB-1757 Glutamate receptor antagonist

2475 0 cp BRD-K15601958 SEW-2871 Lysophospholipid receptor agonist

2476 0 cp BRD-A31007383 SDZ-WAG-994 Adenosine receptor agonist

2477 0 cp BRD-A82590476 SDZ-NKT-343 Tachykinin antagonist

2478 0 cp BRD-K15868788 SDZ-205-557 Serotonin receptor antagonist

2479 0 cp BRD-A62035778 scopolamine Acetylcholine receptor antagonist

2480 0 cp BRD-K81225797 SCH-58261 Adenosine receptor antagonist

2481 0 cp BRD-K94270326 ecopipam Dopamine receptor antagonist

2482 0 cp BRD-K26373640 IKK-2-inhibitor IKK inhibitor

2483 0 cp BRD-K14767410 SC-560 Cyclooxygenase inhibitor

2484 0 cp BRD-K67298865 SB-431542 TGF beta receptor inhibitor

2485 0 cp BRD-K76805682 SB-415286 Glycogen synthase kinase inhibitor

2486 0 cp BRD-K59331372 SB-366791 TRPV antagonist

2487 0 cp BRD-K41567364 SB-334867 Orexin receptor antagonist

2488 0 cp BRD-K24201553 SB-269970 Serotonin receptor antagonist

2489 0 cp BRD-K61097567 SB-218795 Tachykinin antagonist

2490 0 cp BRD-A22707317 SB-205384 GABA receptor modulator

2491 0 cp BRD-K83637872 SANT-1 Smoothened receptor antagonist

2492 0 cp BRD-K19284129 salvinorin-a Opioid receptor agonist

2493 0 cp BRD-K14965640 ibuprofen Cyclooxygenase inhibitor

2494 0 cp BRD-K05901394 terguride Dopamine receptor agonist

2495 0 cp BRD-K06426971 ryuvidine Histone lysine methyltransferase inhibitor

2496 0 cp BRD-K80778372 RO-19-4605 GABA benzodiazepine site receptor inverse agonist

2497 0 cp BRD-K79684402 RO-10-5824 Dopamine receptor agonist

2498 0 cp BRD-A63546914 RO-04-5595 Glutamate receptor antagonist

2499 0 cp BRD-K31627533 rimexolone Glucocorticoid receptor agonist

2500 0 cp BRD-K21283037 riluzole Glutamate inhibitor

2501 0 cp BRD-K54094468 remoxipride Dopamine receptor antagonist

2502 0 cp BRD-A43974499 reboxetine Adrenergic receptor antagonist

2503 0 cp BRD-K04111260 raclopride Dopamine receptor antagonist

2504 0 cp BRD-K50018155 RS-67506 Serotonin receptor partial agonist

2505 0 cp BRD-K87510569 RS-504393 CC chemokine receptor antagonist

2506 0 cp BRD-K26160755 RS-45041-190 Imidazoline receptor agonist

2507 0 cp BRD-K76840893 RS-17053 Adrenergic receptor antagonist

2508 0 cp BRD-K80725821 RS-16566 Serotonin receptor antagonist

2509 0 cp BRD-K34330170 rotenonic-acid Retinoid receptor antagonist

2510 0 cp BRD-K56047318 RHC-80267 Triacylglycerol lipase inhibitor

2511 0 cp BRD-K21853356 RG-14620 EGFR inhibitor

2512 0 cp BRD-A68281735 REV-5901 Leukotriene receptor antagonist

2513 0 cp BRD-K95921201 reserpine Vesicular monoamine transporter inhibitor

2514 0 cp BRD-K15588452 R-96544 Serotonin receptor antagonist

2515 0 cp BRD-K32412559 morphothebaine Adrenergic receptor antagonist

2516 0 cp BRD-A19053259 pseudopelletierine Anthelmintic

2517 0 cp BRD-K77171813 proxyfan Histamine receptor modulator

2518 0 cp BRD-K26521938 dinoprostone Prostanoid receptor agonist

2519 0 cp BRD-K18250272 propoxycaine Local anesthetic

2520 0 cp BRD-A22684332 procaterol Adrenergic receptor agonist

2521 0 cp BRD-K60511616 pravastatin HMGCR inhibitor

2522 0 cp BRD-K96862998 pirfenidone TGF beta receptor inhibitor

2523 0 cp BRD-K87990216 piretanide Glucocorticoid receptor agonist

2524 0 cp BRD-K66874953 pifithrin-alpha TP53 inhibitor

2525 0 cp BRD-A37817666 picrotoxin GABA receptor antagonist

2526 0 cp BRD-K26241953 piceatannol SYK inhibitor

2527 0 cp BRD-K68873215 phosphodiesterase-V-inhibitor-II Phosphodiesterase inhibitor

2528 0 cp BRD-K11163873 phenanthridone PARP inhibitor

2529 0 cp BRD-K88429204 pyrimethamine Dihydrofolate reductase inhibitor

2530 0 cp BRD-K97564742 mepyramine Histamine receptor antagonist

2531 0 cp BRD-A28970875 puromycin Protein synthesis inhibitor

2532 0 cp BRD-K10177585 PSB-11 Adenosine receptor antagonist

2533 0 cp BRD-K97863768 prothionamide Mycobacterium tuberculosis enoyl-[acyl-carrier-protein] reductase [NADH] (inhA) inhibitor

2534 0 cp BRD-K82865713 prostaglandin-b2 cAMP inhibitor

2535 0 cp BRD-K04010869 prostaglandin-a1 HSP inducer

2536 0 cp BRD-K85242180 beta-CCP "Indoleamine 2,3-dioxygenase inhibitor"

2537 0 cp BRD-K82255054 propofol GABA receptor agonist

2538 0 cp BRD-K72029282 probucol Atherogenesis inhibitor

2539 0 cp BRD-K67537649 PQ-401 IGF-1 inhibitor

2540 0 cp BRD-K00312224 PPT Estrogen receptor agonist

2541 0 cp BRD-K47598052 PP-1 SRC inhibitor

2542 0 cp BRD-A95096829 PNU-96415E Dopamine receptor antagonist

2543 0 cp BRD-K28863208 PNU-282987 Cholinergic receptor agonist

2544 0 cp BRD-K16551401 PNU-22394 Serotonin receptor agonist

2545 0 cp BRD-K71731651 PNU-120596 Acetylcholine receptor agonist

2546 0 cp BRD-K43978949 PIT Purinergic receptor antagonist

2547 0 cp BRD-K97365803 PI-828 PI3K inhibitor

2548 0 cp BRD-K76568384 PHTPP Estrogen receptor antagonist

2549 0 cp BRD-K10843433 phenylbutazone Cyclooxygenase inhibitor

2550 0 cp BRD-K95435023 PHA-665752 c-Met inhibitor

2551 0 cp BRD-A70268693 PG-9 Acetylcholine receptor agonist

2552 0 cp BRD-K57718010 pentylenetetrazol GABA receptor antagonist

2553 0 cp BRD-K57569181 pentoxifylline Phosphodiesterase inhibitor

2554 0 cp BRD-K82823804 SA-792987 PKC inhibitor

2555 0 cp BRD-K77133231 PD-169316 p38 MAPK inhibitor

2556 0 cp BRD-A75478957 PD-166793 Metalloproteinase inhibitor

2557 0 cp BRD-A89337244 PD-102807 Acetylcholine receptor antagonist

2558 0 cp BRD-K52721684 PCO-400 Potassium channel activator

2559 0 cp BRD-K15567136 papaverine Phosphodiesterase inhibitor

2560 0 cp BRD-A22380646 pantoprazole ATPase inhibitor

2561 0 cp BRD-K19525698 ozagrel Thromboxane synthase inhibitor

2562 0 cp BRD-K16195444 oxymetazoline Adrenergic receptor agonist

2563 0 cp BRD-K51816706 oxindole-I VEGFR inhibitor

2564 0 cp BRD-K82731415 olomoucine CDK inhibitor

2565 0 cp BRD-K59037100 oxybenzone Lipase inhibitor

2566 0 cp BRD-A53576514 orphenadrine Acetylcholine receptor antagonist

2567 0 cp BRD-A39522003 OMDM-2 FAAH inhibitor

2568 0 cp BRD-K48029790 OBAA Phospholipase inhibitor

2569 0 cp BRD-A51393488 noscapine Bradykinin receptor antagonist

2570 0 cp BRD-A10355991 norketamine Glutamate receptor antagonist

2571 0 cp BRD-K63165456 norcyclobenzaprine Adrenergic receptor agonist

2572 0 cp BRD-A95696066 nisoxetine Norepinephrine reuptake inhibitor

2573 0 cp BRD-K76775527 nimesulide Cyclooxygenase inhibitor

2574 0 cp BRD-A00100033 nifurtimox DNA inhibitor

2575 0 cp BRD-A87719232 naproxen Cyclooxygenase inhibitor

2576 0 cp BRD-A82656074 naltrindole Opioid receptor antagonist

2577 0 cp BRD-A21723284 naltriben Opioid receptor antagonist

2578 0 cp BRD-A41833852 naloxone Opioid receptor antagonist

2579 0 cp BRD-K18574842 nafcillin Bacterial cell wall synthesis inhibitor

2580 0 cp BRD-K10136726 tosyllysyl-chloromethyl-ketone Chymotrypsin inhibitor

2581 0 cp BRD-A94413429 NTNCB Neuropeptide receptor antagonist

2582 0 cp BRD-K13169950 NSC-3852 HDAC inhibitor

2583 0 cp BRD-A47633927 NPC-15199 ICAM1 antagonist

2584 0 cp BRD-A00758722 noretynodrel Progestogen hormone

2585 0 cp BRD-K32696739 noreleagnine Monoamine oxidase inhibitor

2586 0 cp BRD-K88625236 nonoxynol-9 Membrane integrity inhibitor

2587 0 cp BRD-K06753942 nobiletin MEK inhibitor

2588 0 cp BRD-K78122587 NNC-55-0396 T-type calcium channel blocker

2589 0 cp BRD-K73589491 nizatidine Histamine receptor antagonist

2590 0 cp BRD-K23566484 nilutamide Androgen receptor antagonist

2591 0 cp BRD-K05181084 NGB-2904 Dopamine receptor antagonist

2592 0 cp BRD-A78877355 nefopam Cyclooxygenase inhibitor

2593 0 cp BRD-K61177364 NBI-27914 CRF receptor antagonist

2594 0 cp BRD-A23683907 NAS-181 Serotonin receptor antagonist

2595 0 cp BRD-K69195780 NAN-190 Serotonin receptor agonist

2596 0 cp BRD-K60060639 methyllidocaine antiarrhythmic medication

2597 0 cp BRD-K10042277 desmethylclozapine Acetylcholine receptor agonist

2598 0 cp BRD-K40990712 hexamethyleneamiloride Sodium/hydrogen antiport inhibitor

2599 0 cp BRD-A78377521 monastrol Kinesin-like spindle protein inhibitor

2600 0 cp BRD-K60640630 mometasone Glucocorticoid receptor agonist

2601 0 cp BRD-A44780397 mifepristone Glucocorticoid receptor antagonist

2602 0 cp BRD-A19661776 mianserin Serotonin receptor antagonist

2603 0 cp BRD-A63667919 methylergometrine Dopamine receptor antagonist

2604 0 cp BRD-K09859624 methantheline Acetylcholine receptor antagonist

2605 0 cp BRD-K38477985 malonoben Protein tyrosine kinase inhibitor

2606 0 cp BRD-K92428153 mycophenolate-mofetil Dehydrogenase inhibitor

2607 0 cp BRD-K90524085 MY-5445 Phosphodiesterase inhibitor

2608 0 cp BRD-A99449986 MT-21 Caspase activator

2609 0 cp BRD-A32949107 MRS-1845 Calcium channel blocker

2610 0 cp BRD-A30590053 MR-16728 Acetylcholine release enhancer

2611 0 cp BRD-A16332958 modafinil Adrenergic receptor agonist

2612 0 cp BRD-K63151507 MNITMT Lymphocyte inhibitor

2613 0 cp BRD-K53878242 MMPX Phosphodiesterase inhibitor

2614 0 cp BRD-K93201660 ML-7 Myosin light chain kinase inhibitor

2615 0 cp BRD-K95202259 ML-3163 p38 MAPK inhibitor

2616 0 cp BRD-A03623303 metoprolol Adrenergic receptor antagonist

2617 0 cp BRD-A41145729 methoprene-acid Retinoid receptor agonist

2618 0 cp BRD-A94709349 metaxalone Muscle relaxant

2619 0 cp BRD-A14798026 mestranol Estrogen receptor agonist

2620 0 cp BRD-A14395271 mesoridazine Dopamine receptor antagonist

2621 0 cp BRD-K12244279 MEK1-2-inhibitor MEK inhibitor

2622 0 cp BRD-K09764130 mead-ethanolamide Cannabinoid receptor agonist

2623 0 cp BRD-K96144918 mead-acid KPL-1 tumor suppressor

2624 0 cp BRD-K43245338 MDL-28170 Calpain inhibitor

2625 0 cp BRD-A17453586 MDL-72832 Serotonin receptor agonist

2626 0 cp BRD-A85587465 bemesetron Serotonin receptor antagonist

2627 0 cp BRD-K09635134 l-erythro-MAPP negative control for D-erythro-MAPP

2628 0 cp BRD-K76274772 MAPP-D-erythro Ceramidase inhibitor

2629 0 cp BRD-A59215453 lobelanidine Acetylcholine receptor antagonist

2630 0 cp BRD-K03384561 roquinimex Angiogenesis inhibitor

2631 0 cp BRD-K24526313 levcromakalim Potassium channel activator

2632 0 cp BRD-K92492521 LY-255283 Leukotriene receptor antagonist

2633 0 cp BRD-A12016240 LY-278584 Serotonin receptor antagonist

2634 0 cp BRD-K14282469 LY-165163 Serotonin receptor antagonist

2635 0 cp BRD-K13261168 LY-16350 Dopamine receptor agonist

2636 0 cp BRD-K06234293 LY-364947 TGF beta receptor inhibitor

2637 0 cp BRD-K24675965 LY-288513 CCK receptor antagonist

2638 0 cp BRD-K33864865 LY-225910 CCK receptor antagonist

2639 0 cp BRD-K33396764 alpha-linolenic-acid Omega 3 fatty acid stimulant

2640 0 cp BRD-K52662033 lidocaine Histamine receptor agonist

2641 0 cp BRD-K66707493 lawsone Coloring agent

2642 0 cp BRD-K23583188 lavendustin-a EGFR inhibitor

2643 0 cp BRD-K28806945 L-750667 Dopamine receptor antagonist

2644 0 cp BRD-K05181463 L-741626 Dopamine receptor antagonist

2645 0 cp BRD-K10176267 L-701252 Glutamate receptor antagonist

2646 0 cp BRD-K70241288 L-692585 Growth hormone releasing peptide ligand agonist

2647 0 cp BRD-K40656405 L-165041 PPAR receptor agonist

2648 0 cp BRD-K14765469 vesamicol Acetylcholinesterase inhibitor

2649 0 cp BRD-A43930669 L-368899 Oxytocin receptor antagonist

2650 0 cp BRD-K49671696 ketanserin Serotonin receptor antagonist

2651 0 cp BRD-A38747044 KU-14R Imidazoline receptor ligand

2652 0 cp BRD-A81177136 KN-62 Calcium-calmodulin dependent protein kinase inhibitor

2653 0 cp BRD-A75455249 kavain Calcium channel modulator

2654 0 cp BRD-K05464208 JX-401 p38 MAPK inhibitor

2655 0 cp BRD-K17796732 JWH-015 Cannabinoid receptor agonist

2656 0 cp BRD-K15086322 JNJ-10191584 Histamine receptor antagonist

2657 0 cp BRD-K04546108 JAK3-inhibitor-VI JAK inhibitor

2658 0 cp BRD-K95676198 JAK3-inhibitor-V JAK inhibitor

2659 0 cp BRD-K52850071 JAK3-Inhibitor-II JAK inhibitor

2660 0 cp BRD-K26429091 J-104129 Acetylcholine receptor antagonist

2661 0 cp BRD-K64402243 ivachtin Caspase inhibitor

2662 0 cp BRD-K29173907 isoflupredone Glucocorticoid receptor agonist

2663 0 cp BRD-K79124250 ioxaglic-acid Radiopaque medium

2664 0 cp BRD-K51918615 iodophenpropit Histamine receptor antagonist

2665 0 cp BRD-K43860855 iobenguane Antineoplastic

2666 0 cp BRD-K25906698 immepip Histamine receptor agonist

2667 0 cp BRD-A48570745 ivermectin GABA receptor agonist

2668 0 cp BRD-K60298136 ITE Aryl hydrocarbon receptor agonist

2669 0 cp BRD-K06198550 isorotenone Mitochondrial complex I inhibitor

2670 0 cp BRD-K37080523 isoreserpine Vesicular monoamine transporter inhibitor

2671 0 cp BRD-K92678294 irilin-a Isoflavone

2672 0 cp BRD-K07117950 imperatorin CDK inhibitor

2673 0 cp BRD-A03359064 ICI-89406 Adrenergic receptor antagonist

2674 0 cp BRD-K73290745 ICI-199441 Opioid receptor agonist

2675 0 cp BRD-K66782112 BRD-K66782112 Histamine receptor antagonist

2676 0 cp BRD-K09638361 SA-63133 -666

2677 0 cp BRD-K15196155 IBC-293 Hydroxycarboxylic acid receptor agonist

2678 0 cp BRD-K85383046 IAA-94 Chloride channel blocker

2679 0 cp BRD-K40530731 hyoscyamine Acetylcholine receptor antagonist

2680 0 cp BRD-K92726801 hydrastinine Haemostatic agent

2681 0 cp BRD-A77722753 hydralazine Vasodilator

2682 0 cp BRD-K08554278 bisbenzimide DNA binding agent

2683 0 cp BRD-K07325606 hispidin PKC inhibitor

2684 0 cp BRD-K71003802 hippeastrine Plant alkaloid

2685 0 cp BRD-K37691127 hinokitiol Tyrosinase inhibitor

2686 0 cp BRD-K38903228 hesperidin Flavanone glycoside

2687 0 cp BRD-K07996107 harpagoside Acetylcholinesterase inhibitor

2688 0 cp BRD-A06390036 hydroquinidine Antiarrhythmic

2689 0 cp BRD-A25143711 hydrocortisone Glucocorticoid receptor agonist

2690 0 cp BRD-K02715688 hydrastine Tyrosine hydroxylase inhibitor

2691 0 cp BRD-A34751532 homosalate HSP inducer

2692 0 cp BRD-K81209159 herniarin Acetylcholinesterase inhibitor

2693 0 cp BRD-A24429032 HEAT Adrenergic receptor antagonist

2694 0 cp BRD-K05434375 HA-1004 Calcium channel blocker

2695 0 cp BRD-K70577657 H-9 PKA inhibitor

2696 0 cp BRD-K18742343 H-8 PKA inhibitor

2697 0 cp BRD-K92758126 gibberellic-acid NFkB pathway inhibitor

2698 0 cp BRD-K49890030 gavestinel Glutamate receptor antagonist

2699 0 cp BRD-K59256312 gabexate Serine protease inhibitor

2700 0 cp BRD-K24240364 GYKI-52466 Glutamate receptor antagonist

2701 0 cp BRD-K93258693 GW-9662 PPAR receptor antagonist

2702 0 cp BRD-K71534238 GW-9508 Free fatty acid receptor agonist

2703 0 cp BRD-K90382497 GW-843682X PLK inhibitor

2704 0 cp BRD-A72596465 GW-6471 PPAR receptor antagonist

2705 0 cp BRD-K79930101 GW-583340 EGFR inhibitor

2706 0 cp BRD-K04146668 GW-441756 Growth factor receptor inhibitor

2707 0 cp BRD-K82983861 GW-0742 PPAR receptor agonist

2708 0 cp BRD-K67860401 AR-A014418 Glycogen synthase kinase inhibitor

2709 0 cp BRD-K75478907 GS-39783 GABA receptor modulator

2710 0 cp BRD-A93659613 GR-89696 Opioid receptor agonist

2711 0 cp BRD-K40578143 GR-79236 Adenosine receptor agonist

2712 0 cp BRD-K46441700 GR-55562 Serotonin receptor antagonist

2713 0 cp BRD-K02283807 GR-32191 Thromboxane receptor antagonist

2714 0 cp BRD-K52394958 GR-159897 Tachykinin antagonist

2715 0 cp BRD-K11634954 GBR-13069 Dopamine uptake inhibitor

2716 0 cp BRD-K50135270 GBR-12935 Dopamine uptake inhibitor

2717 0 cp BRD-K77998258 ganglioside SRC activator

2718 0 cp BRD-K87049188 fusaric-acid Dopamine beta hydroxylase inhibitor

2719 0 cp BRD-M00539986 formoterol Adrenergic receptor agonist

2720 0 cp BRD-A49734948 foliosidine Plant alkaloid

2721 0 cp BRD-K72676686 fluvoxamine Selective serotonin reuptake inhibitor (SSRI)

2722 0 cp BRD-A13650332 flucloxacillin Bacterial cell wall synthesis inhibitor

2723 0 cp BRD-K34170797 fexaramine FXR agonist

2724 0 cp BRD-K28168037 fenretinide Apoptosis stimulant

2725 0 cp BRD-K41160163 fenobam Glutamate receptor antagonist

2726 0 cp BRD-A30815329 felodipine Calcium channel blocker

2727 0 cp BRD-K76617868 fasudil Rho associated kinase inhibitor

2728 0 cp BRD-K08998509 fananserin Dopamine receptor antagonist

2729 0 cp BRD-K96402602 farnesylthiotriazole PPMTase inhibitor

2730 0 cp BRD-K66944906 fraxidin Carbonic anhydrase inhibitor

2731 0 cp BRD-A49765801 fludroxycortide Glucocorticoid receptor agonist

2732 0 cp BRD-A65449987 flunisolide Cytochrome P450 inhibitor

2733 0 cp BRD-A01346607 flumetasone Glucocorticoid receptor agonist

2734 0 cp BRD-K44067360 flufenamic-acid Chloride channel blocker

2735 0 cp BRD-K48367671 febuxostat Xanthine oxidase inhibitor

2736 0 cp BRD-K14821540 FCCP Mitochondrial oxidative phosphorylation uncoupler

2737 0 cp BRD-K63089472 farnesylthioacetic-acid Inhibitor of methyl esterification of farnesylated proteins

2738 0 cp BRD-K81839095 estrone Estrogen receptor agonist

2739 0 cp BRD-A99411506 esculin Antioxidant

2740 0 cp BRD-K04046242 equilin Estrogen receptor agonist

2741 0 cp BRD-A39969961 eplerenone Cytochrome P450 inhibitor

2742 0 cp BRD-K46068882 eugenitol Bacterial quorum sensing inhibitor

2743 0 cp BRD-K15842202 eudesmic-acid -666

2744 0 cp BRD-A39747742 estradiol-valerate Estrogen receptor agonist

2745 0 cp BRD-A35033682 eriodictyol Cytochrome P450 inhibitor

2746 0 cp BRD-A73581086 ergometrine Adrenergic receptor agonist

2747 0 cp BRD-K19360254 ergocornine Dopamine receptor agonist

2748 0 cp BRD-K11927976 ER-27319 Mediator release inhibitor

2749 0 cp BRD-A53131506 epitestosterone Inactive testosterone analog

2750 0 cp BRD-K42452249 EO-1428 p38 MAPK inhibitor

2751 0 cp BRD-K88759641 EMD-66684 Angiotensin receptor antagonist

2752 0 cp BRD-K16233984 eriochrome-black-t Azo dye

2753 0 cp BRD-A96485169 EBPC Aldose reductase inhibitor

2754 0 cp BRD-K92093830 doxorubicin Topoisomerase inhibitor

2755 0 cp BRD-K54759182 dosulepin Norepinephrine reuptake inhibitor

2756 0 cp BRD-K73391359 quinisocaine Local anesthetic

2757 0 cp BRD-K17674993 diflorasone Corticosteroid agonist

2758 0 cp BRD-K98521173 desoxycortone Mineralocorticoid receptor agonist

2759 0 cp BRD-K37792168 denbufylline Phosphodiesterase inhibitor

2760 0 cp BRD-A99177642 deltaline Acetylcholine receptor antagonist

2761 0 cp BRD-K96527333 dehydroisoandosterone GABA receptor modulator

2762 0 cp BRD-K94920105 DR-2313 PARP inhibitor

2763 0 cp BRD-K80315159 DPPE Histamine receptor antagonist

2764 0 cp BRD-A27143604 DPN Estrogen receptor agonist

2765 0 cp BRD-K01624546 docosatrienoic-acid LTB4 inhibitor

2766 0 cp BRD-K39965020 doconexent PPAR receptor agonist

2767 0 cp BRD-K85266041 DNQX Glutamate receptor antagonist

2768 0 cp BRD-K31699485 DMEOB glutamate receptor modulator

2769 0 cp BRD-K89274813 DMP-543 Acetylcholine release stimulant

2770 0 cp BRD-K32645441 dipropyl-5ct Serotonin receptor agonist

2771 0 cp BRD-K94080537 diethyltoluamide DEET activator of fly antenna ionotropic receptor IR40a

2772 0 cp BRD-K81521265 dicyclohexylurea Epoxide hydolase inhibitor

2773 0 cp BRD-K29178788 dictamnine Furoquinoline alkaloid

2774 0 cp BRD-K76133116 benzydamine Membrane integrity inhibitor

2775 0 cp BRD-K06014311 DH-97 Melatonin receptor antagonist

2776 0 cp BRD-A75402480 desoxycorticosterone Mineralocorticoid receptor agonist

2777 0 cp BRD-K61401890 deguelin NADH-ubiquinone oxidoreductase (Complex I) inhibitor

2778 0 cp BRD-K32526544 DCEBIO Potassium channel activator

2779 0 cp BRD-K74430258 "1,2-dichlorobenzene" Hepatotoxicant

2780 0 cp BRD-K31633810 DAU-5884 Acetylcholine receptor antagonist

2781 0 cp BRD-K10065684 dantron Laxative

2782 0 cp BRD-A73909368 dactinomycin RNA polymerase inhibitor

2783 0 cp BRD-K09132007 D-4476 TGF beta receptor inhibitor

2784 0 cp BRD-A10420615 cyclopiazonic-acid ATPase inhibitor

2785 0 cp BRD-K95992530 Cyclo-[Arg-Gly-Asp-D-Phe-Val] integrin antagonist

2786 0 cp BRD-K89046952 ciclacillin Bacterial cell wall synthesis inhibitor

2787 0 cp BRD-A76093993 cromakalim Potassium channel activator

2788 0 cp BRD-K08890269 CO-102862 Sodium channel blocker

2789 0 cp BRD-K38003476 clocortolone Glucocorticoid receptor agonist

2790 0 cp BRD-A49358627 ciprofibrate PPAR receptor agonist

2791 0 cp BRD-K72816382 cinalukast Leukotriene receptor antagonist

2792 0 cp BRD-K18618618 cimetidine Histamine receptor antagonist

2793 0 cp BRD-A07875874 cilnidipine Calcium channel blocker

2794 0 cp BRD-A90311807 cilastatin Dehydropeptidase inhibitor

2795 0 cp BRD-K02275692 cefotiam Bacterial cell wall synthesis inhibitor

2796 0 cp BRD-K37848908 ceforanide Penicillin binding protein inhibitor

2797 0 cp BRD-K71860425 CDK2-5-inhibitor CDK inhibitor

2798 0 cp BRD-K82484965 carmoxirole Dopamine receptor agonist

2799 0 cp BRD-K44849676 capsazepine TRPV agonist

2800 0 cp BRD-K46556543 canrenoic-acid Mineralocorticoid receptor antagonist

2801 0 cp BRD-A30437061 camptothecin Topoisomerase inhibitor

2802 0 cp BRD-K59851896 calycanthine GABA release inhibitor

2803 0 cp BRD-A63043573 cabergoline Dopamine receptor agonist

2804 0 cp BRD-K79353516 indolophenanthridine CALY activator

2805 0 cp BRD-A18202423 CPCCOEt Glutamate receptor antagonist

2806 0 cp BRD-K33860217 CP-94253 Serotonin receptor agonist

2807 0 cp BRD-K81876028 CP-93129 Serotonin receptor agonist

2808 0 cp BRD-A03816571 CP-55940 Cannabinoid receptor agonist

2809 0 cp BRD-K96271548 coumaric-acid Antioxidant

2810 0 cp BRD-K13642330 cosmosiin Cytochrome P450 inhibitor

2811 0 cp BRD-K00662280 CL-218872 GABA receptor agonist

2812 0 cp BRD-K67680372 CI-966 GAT inhibitor

2813 0 cp BRD-A81866333 CGS-21680 Adenosine receptor agonist

2814 0 cp BRD-K68103045 CGS-20625 Benzodiazepine receptor agonist

2815 0 cp BRD-K65786282 CGP-7930 GABA receptor modulator

2816 0 cp BRD-A89672324 CGP-55845 GABA receptor antagonist

2817 0 cp BRD-A04668240 CGP-52432 GABA receptor antagonist

2818 0 cp BRD-A35623999 CGP-37157 L-type calcium channel blocker

2819 0 cp BRD-A34706053 CGP-12177 Adrenergic receptor agonist

2820 0 cp BRD-K06159959 CCMQ Inhibitor of the binding of homoquinolinic acid to non-NMDA sensitive sites

2821 0 cp BRD-K15616905 CCCP Mitochondrial oxidative phosphorylation uncoupler

2822 0 cp BRD-K53570330 carbofuran Cholinesterase inhibitor

2823 0 cp BRD-A98702003 carbenoxolone 11-beta-HSD1 inhibitor

2824 0 cp BRD-K27499107 carbacyclin IP receptor activator

2825 0 cp BRD-K03440695 boldine Acetylcholine receptor antagonist

2826 0 cp BRD-A36471396 biperiden Acetylcholine receptor antagonist

2827 0 cp BRD-K89210380 biotin Vitamin B

2828 0 cp BRD-K18779551 bifemelane Acetylcholine release stimulant

2829 0 cp BRD-K04185004 oxybuprocaine Local anesthetic

2830 0 cp BRD-K39111395 BCL2-inhibitor BCL inhibitor

2831 0 cp BRD-A17428743 BW-723C86 Serotonin receptor agonist

2832 0 cp BRD-A16665823 butoconazole Bacterial cell wall synthesis inhibitor

2833 0 cp BRD-K25905511 buddleoflavonoloside Acetylcholinesterase inhibitor

2834 0 cp BRD-K11696279 BU-239 Imidazoline receptor agonist

2835 0 cp BRD-K17868609 BRL-54443 Serotonin receptor agonist

2836 0 cp BRD-K07507905 BRL-37344 Adrenergic receptor agonist

2837 0 cp BRD-A51829654 BRL-15572 Serotonin receptor antagonist

2838 0 cp BRD-K45479396 BP-554 Serotonin receptor agonist

2839 0 cp BRD-A15435692 BMY-14802 Sigma receptor antagonist

2840 0 cp BRD-K32584078 BML-257 AKT inhibitor

2841 0 cp BRD-K94379058 BML-190 Cannabinoid receptor inverse agonist

2842 0 cp BRD-K91315211 betahistine Histamine receptor agonist

2843 0 cp BRD-K09668667 benzo(a)pyrene Carcinogen

2844 0 cp BRD-K29668683 BD-1063 Sigma receptor antagonist

2845 0 cp BRD-K36864847 BD-1047 Adrenergic receptor antagonist

2846 0 cp BRD-K40919711 BAPTA-AM Potassium channel blocker

2847 0 cp BRD-K40742111 baeomycesic-acid Lipoxygenase inhibitor

2848 0 cp BRD-A84189516 baccatin-III Paclitaxel precursor

2849 0 cp BRD-K23922020 arecaidine Acetylcholine receptor agonist

2850 0 cp BRD-K63792901 arecaidine Acetylcholine receptor agonist

2851 0 cp BRD-K15164005 apoptosis-activator-II Carboxylesterase inhibitor

2852 0 cp BRD-K88611939 aniracetam Glutamate receptor agonist

2853 0 cp BRD-K36638830 anabasine Acetylcholine receptor agonist

2854 0 cp BRD-K39670393 amthamine Histamine receptor agonist

2855 0 cp BRD-K07762753 aminopurvalanol-a Tyrosine kinase inhibitor

2856 0 cp BRD-K52459643 prostaglandin-e1 Prostanoid receptor agonist

2857 0 cp BRD-K36258877 AZ-10417808 Caspase inhibitor

2858 0 cp BRD-K78838262 austricine Hypolipidemic

2859 0 cp BRD-K85013741 auraptene Nitric oxide production inhibitor

2860 0 cp BRD-K29555132 arachidonamide Cannabinoid receptor agonist

2861 0 cp BRD-K81729199 AQ-RA741 Acetylcholine receptor antagonist

2862 0 cp BRD-A95513702 "androsta-1,4-dien-3,17-dione" Aromatase inhibitor

2863 0 cp BRD-K78280988 anandamide Cannabinoid receptor agonist

2864 0 cp BRD-A28318179 aminomethyltransferase Nitric oxide synthase inhibitor

2865 0 cp BRD-A11813248 AM-92016 Potassium channel blocker

2866 0 cp BRD-K63533170 AM-630 Cannabinoid receptor antagonist

2867 0 cp BRD-K21667562 AM-404 Cyclooxygenase inhibitor

2868 0 cp BRD-K89055274 alverine Muscle relaxant

2869 0 cp BRD-K59469039 AG-879 Angiogenesis inhibitor

2870 0 cp BRD-K33204703 AG-370 PDGFR receptor inhibitor

2871 0 cp BRD-K41996876 tyrphostin-1 EGFR inhibitor

2872 0 cp BRD-K87919739 tyrphostin-AG-825 Receptor tyrosine protein kinase inhibitor

2873 0 cp BRD-K07888196 tyrphostin-AG-538 IGF-1 inhibitor

2874 0 cp BRD-K12357156 AG-490 EGFR inhibitor

2875 0 cp BRD-K49657628 tyrphostin-AG-18 EGFR inhibitor

2876 0 cp BRD-K94841585 emodic-acid Laxative

2877 0 cp BRD-K87991767 umbelliferone Cyclooxygenase inhibitor

2878 0 cp BRD-K80348542 cephaeline Protein synthesis inhibitor

2879 0 cp BRD-K05906022 limonin HIV protease inhibitor

2880 0 cp BRD-A95445494 maackiain Sodium/glucose cotransporter inhibitor

2881 0 cp BRD-K46384212 o-3M3FBS phospholipase activator

2882 0 cp BRD-A11135865 nor-binaltorphimine Opioid receptor antagonist

2883 0 cp BRD-K17110974 aristolochic-acid Phospholipase inhibitor

2884 0 cp BRD-K80451230 zamifenacin Acetylcholine receptor antagonist

2885 0 cp BRD-K66353228 zoxazolamine Myorelaxant

2886 0 cp BRD-K45296539 ZD-7114 Adrenergic receptor agonist

2887 0 cp BRD-K06712146 YM-90709 IL5 inhibitor

2888 0 cp BRD-K44084986 Y-27632 Rho associated kinase inhibitor

2889 0 cp BRD-K42748308 XE-991 Potassium channel blocker

2890 0 cp BRD-K55430733 WAY-629 Serotonin receptor agonist

2891 0 cp BRD-K41868777 W-5 Calmodulin antagonist

2892 0 cp BRD-M45964048 verteporfin Photosensitizing agent

2893 0 cp BRD-K95655893 MAZ-51 VEGFR inhibitor

2894 0 cp BRD-K51018020 VAMA-37 DNA dependent protein kinase inhibitor

2895 0 cp BRD-K20995441 U-54494A Opioid receptor agonist

2896 0 cp BRD-A60294240 tribenoside Anti-inflammatory

2897 0 cp BRD-K84663978 trequinsin Phosphodiesterase inhibitor

2898 0 cp BRD-K30480208 torasemide Electrolyte reabsorption inhibitor

2899 0 cp BRD-A72988804 tiaprofenic-acid Cyclooxygenase inhibitor

2900 0 cp BRD-A53077924 tianeptine Selective serotonin reuptake enhancer (SSRE)

2901 0 cp BRD-K69600043 thiethylperazine Dopamine receptor antagonist

2902 0 cp BRD-K95739795 tetrabenazine Vesicular monoamine transporter inhibitor

2903 0 cp BRD-K53220666 trimetozine Sedative

2904 0 cp BRD-A92439610 triamcinolone Glucocorticoid receptor agonist

2905 0 cp BRD-K70778732 trazodone Adrenergic receptor antagonist

2906 0 cp BRD-K64514229 toltrazuril Antiprotozoal

2907 0 cp BRD-K09631521 thiotepa Cytochrome P450 inhibitor

2908 0 cp BRD-A42831637 tetrahydrocannabinol-7-oic-acid Anti-inflammatory

2909 0 cp BRD-A06352418 terfenadine Histamine receptor antagonist

2910 0 cp BRD-A22844106 tenoxicam Cyclooxygenase inhibitor

2911 0 cp BRD-K34820100 tebuthiuron Photosynthesis inhibitor

2912 0 cp BRD-K55454768 TAS-301 Calcium-calmodulin dependent protein kinase inhibitor

2913 0 cp BRD-K93645900 tadalafil Phosphodiesterase inhibitor

2914 0 cp BRD-K50495309 SRC-kinase-inhibitor-I SRC inhibitor

2915 0 cp BRD-A77299732 salubrinal Eukaryotic translation initiation factor inhibitor

2916 0 cp BRD-K51476772 ST-638 Tyrosine kinase inhibitor

2917 0 cp BRD-A72066420 mifobate PPAR receptor antagonist

2918 0 cp BRD-K55468218 spiperone Dopamine receptor antagonist

2919 0 cp BRD-A92161634 scopoline Acetylcholine receptor antagonist

2920 0 cp BRD-K52512893 SC-19220 Prostanoid receptor antagonist

2921 0 cp BRD-K80639402 SB-258585 Serotonin receptor antagonist

2922 0 cp BRD-K30867024 SB-216641 Serotonin receptor antagonist

2923 0 cp BRD-K58299615 RO-90-7501 Beta amyloid inhibitor

2924 0 cp BRD-K06543683 bisindolylmaleimide-ix CDK inhibitor

2925 0 cp BRD-K82561139 ricinine Casein kinase inhibitor

2926 0 cp BRD-K46678324 RHO-kinase-inhibitor-II Rho associated kinase inhibitor

2927 0 cp BRD-A83431637 resmethrin Cytochrome P450 inhibitor

2928 0 cp BRD-A71765365 mepireserpate Catecholamine depleting sympatholytic

2929 0 cp BRD-K82846253 repaglinide Insulin secretagogue

2930 0 cp BRD-K83063356 RS-102895 CCR antagonist

2931 0 cp BRD-A34255068 rolipram Phosphodiesterase inhibitor

2932 0 cp BRD-K21733600 rofecoxib Cyclooxygenase inhibitor

2933 0 cp BRD-K54411430 robustic-acid cAMP inhibitor

2934 0 cp BRD-K43796186 benzyl-quinazolin-4-yl-amine EGFR inhibitor

2935 0 cp BRD-K75699339 rizatriptan Serotonin receptor agonist

2936 0 cp BRD-A08003242 rhodomyrtoxin-b sodium fluorescein uptake inhibitor

2937 0 cp BRD-K13725475 rhodomyrtoxin Cytotoxic agent

2938 0 cp BRD-K37206356 rhamnetin HDAC inhibitor

2939 0 cp BRD-K85603128 resorcinol Phosphodiesterase inhibitor

2940 0 cp BRD-A54236247 racephedrine Adrenergic receptor agonist

2941 0 cp BRD-K13544237 r(-)-propylnorapomorphine Dopamine receptor agonist

2942 0 cp BRD-K54665485 R-59022 Diacylglycerol kinase inhibitor

2943 0 cp BRD-K40782193 QX-222 Sodium channel blocker

2944 0 cp BRD-K68867920 quetiapine Dopamine receptor antagonist

2945 0 cp BRD-K50836978 purvalanol-a CDK inhibitor

2946 0 cp BRD-K02581333 protein-tyrosine-phosphatase-inhibitor-IV Tyrosine phosphatase inhibitor

2947 0 cp BRD-K38251852 paxilline Potassium channel blocker

2948 0 cp BRD-K49027941 PSB-1115 Adenosine receptor antagonist

2949 0 cp BRD-K79366068 PSB-069 NTPDase inhibitor

2950 0 cp BRD-K71266197 PSB-06126 NTPDase inhibitor

2951 0 cp BRD-K19352500 prochlorperazine Dopamine receptor antagonist

2952 0 cp BRD-K46317332 proadifen Nitric oxide synthase inhibitor

2953 0 cp BRD-A55913614 primaquine Antimalarial

2954 0 cp BRD-A62525898 prednisone Glucocorticoid receptor agonist

2955 0 cp BRD-A01643550 prednisolone Glucocorticoid receptor agonist

2956 0 cp BRD-A27887842 prednisolone Glucocorticoid receptor agonist

2957 0 cp BRD-A57382968 piroxicam Cyclooxygenase inhibitor

2958 0 cp BRD-K59522102 piperine Monoamine oxidase inhibitor

2959 0 cp BRD-A97479839 piperidolate Acetylcholine receptor antagonist

2960 0 cp BRD-K26801045 pipamperone Dopamine receptor antagonist

2961 0 cp BRD-K96319534 phentermine Dopamine uptake inhibitor

2962 0 cp BRD-K21350491 phenamil TRPV antagonist

2963 0 cp BRD-K38323065 phenacetin Cyclooxygenase inhibitor

2964 0 cp BRD-K03842655 penitrem-a Potassium channel blocker

2965 0 cp BRD-K55191674 benzylpenicillin Penicillin binding protein inhibitor

2966 0 cp BRD-A43150328 penicillic-acid other antibiotic

2967 0 cp BRD-A28746609 paclitaxel Tubulin inhibitor

2968 0 cp BRD-A43671941 oxprenolol Adrenergic receptor antagonist

2969 0 cp BRD-K52075715 oxibendazole Tubulin inhibitor

2970 0 cp BRD-K25394294 oxaprozin Cyclooxygenase inhibitor

2971 0 cp BRD-K78485176 olmesartan Angiotensin receptor antagonist

2972 0 cp BRD-K53123955 niridazole Phosphofructokinase inhibitor

2973 0 cp BRD-K02953697 naringin Cytochrome P450 inhibitor

2974 0 cp BRD-A94669766 naringenin Aromatase inhibitor

2975 0 cp BRD-A83855350 naltrexone Opioid receptor antagonist

2976 0 cp BRD-K09537769 NU-7026 DNA dependent protein kinase inhibitor

2977 0 cp BRD-K59637651 NSC-119889 Protein synthesis inhibitor

2978 0 cp BRD-K54210043 NS-1619 Calcium channel activator

2979 0 cp BRD-K79092138 nitrofural Bacterial DNA inhibitor

2980 0 cp BRD-K76810206 nicergoline Adrenergic receptor antagonist

2981 0 cp BRD-A85025557 NCS-382 GABA receptor antagonist

2982 0 cp BRD-K34014345 naproxol Anti-inflammatory

2983 0 cp BRD-K65146499 nabumetone Cyclooxygenase inhibitor

2984 0 cp BRD-A61470182 n-formylmethionylalanine macrophage activator

2985 0 cp BRD-K46862739 metyrapone Cytochrome P450 inhibitor

2986 0 cp BRD-A66563878 medetomidine Adrenergic receptor agonist

2987 0 cp BRD-A06784547 MRS-1334 Adenosine receptor antagonist

2988 0 cp BRD-K19554809 MK-212 Serotonin receptor agonist

2989 0 cp BRD-A31204924 mitotane Antineoplastic

2990 0 cp BRD-A48237631 mitomycin-c DNA alkylating agent

2991 0 cp BRD-A79981887 midodrine Adrenergic receptor agonist

2992 0 cp BRD-A61793559 metolazone Carbonic anhydrase inhibitor

2993 0 cp BRD-K35941380 methysergide Serotonin receptor antagonist

2994 0 cp BRD-A02189320 met-leu-phe -666

2995 0 cp BRD-K91336023 mesulergine Dopamine receptor agonist

2996 0 cp BRD-K18194590 mephentermine Adrenergic receptor agonist

2997 0 cp BRD-A62057054 MDL-11939 Serotonin receptor antagonist

2998 0 cp BRD-K64746805 MBCQ Phosphodiesterase inhibitor

2999 0 cp BRD-K36377456 marmesin Angiogenesis inhibitor

3000 0 cp BRD-A24817035 laudanosine Central nervous system agent

3001 0 cp BRD-K62792802 LY-83583 Guanylyl cyclase inhibitor

3002 0 cp BRD-K27305650 LY-294002 MTOR inhibitor

3003 0 cp BRD-K95899059 LY-344864 Serotonin receptor agonist

3004 0 cp BRD-A92826379 lupanine Sodium channel blocker

3005 0 cp BRD-K39915878 loxapine Dopamine receptor antagonist

3006 0 cp BRD-K15834839 lobendazole Anthelmintic

3007 0 cp BRD-K88849294 lobaric-acid Tyrosine phosphatase inhibitor

3008 0 cp BRD-K08973992 linoleic-acid Oxidative stress inducer

3009 0 cp BRD-A32836748 leu-enkephalin Opioid receptor agonist

3010 0 cp BRD-A84389091 L-655708 GABA receptor inverse agonist

3011 0 cp BRD-K18036262 L-168049 Glucagon receptor antagonist

3012 0 cp BRD-A93206962 L-755507 Adrenergic receptor agonist

3013 0 cp BRD-A85472596 L-670596 Prostanoid receptor antagonist

3014 0 cp BRD-K09497549 kawain Calcium channel modulator

3015 0 cp BRD-A43849199 karakoline Phytotoxin

3016 0 cp BRD-K90574421 ipsapirone Serotonin receptor agonist

3017 0 cp BRD-K60038276 irbesartan Angiotensin receptor antagonist

3018 0 cp BRD-K52219182 BRD-K52219182 Phosphodiesterase inhibitor

3019 0 cp BRD-A48809242 IB-MECA Adenosine receptor agonist

3020 0 cp BRD-K35377380 I-OMe-AG-538 IGF-1 inhibitor

3021 0 cp BRD-A78295502 hydroquinine Antiarrhythmic

3022 0 cp BRD-A22769835 homochlorcyclizine Antihistamine

3023 0 cp BRD-A08709697 heliotrine Pyrrolizidine alkaloid

3024 0 cp BRD-A46186775 hydrocortisone Glucocorticoid receptor agonist

3025 0 cp BRD-A65767837 hydrocortisone Glucocorticoid receptor agonist

3026 0 cp BRD-K99946902 hexylresorcinol Local anesthetic

3027 0 cp BRD-K54771420 glycocholic-acid Cholesterol inhibitor

3028 0 cp BRD-A17535965 gelsemine Acetylcholine receptor antagonist

3029 0 cp BRD-K83023055 GR-135531 Melatonin receptor agonist

3030 0 cp BRD-K49945136 GR-113808 Serotonin receptor antagonist

3031 0 cp BRD-K51662849 ilomastat Matrix metalloprotease inhibitor

3032 0 cp BRD-K76587808 fraxetin Antioxidant

3033 0 cp BRD-K66296774 fluvastatin HMGCR inhibitor

3034 0 cp BRD-A15297126 fluocinonide Glucocorticoid receptor agonist

3035 0 cp BRD-K29582677 flunarizine Calcium channel blocker

3036 0 cp BRD-A38749782 fludrocortisone Glucocorticoid receptor agonist

3037 0 cp BRD-K66093087 FGIN-1-43 Benzodiazepine receptor agonist

3038 0 cp BRD-A97104540 fenoterol Adrenergic receptor agonist

3039 0 cp BRD-K45033733 famciclovir DNA polymerase inhibitor

3040 0 cp BRD-K40227168 vinburnine Adrenergic receptor antagonist

3041 0 cp BRD-A41519720 ezetimibe Niemann-Pick C1-like 1 protein antagonist

3042 0 cp BRD-A73741725 exemestane Aromatase inhibitor

3043 0 cp BRD-K32977963 eugenol Androgen receptor antagonist

3044 0 cp BRD-A74667430 etodolac Cyclooxygenase inhibitor

3045 0 cp BRD-K08556791 ethoprop Acetylcholinesterase inhibitor

3046 0 cp BRD-A36066264 estradiol-benzoate Estrogen receptor agonist

3047 0 cp BRD-K70301876 escitalopram Selective serotonin reuptake inhibitor (SSRI)

3048 0 cp BRD-K39746403 erythromycin NFkB pathway inhibitor

3049 0 cp BRD-K04548931 pidorubicine Topoisomerase inhibitor

3050 0 cp BRD-K50660797 epicatechin Bacterial DNA gyrase inhibitor

3051 0 cp BRD-K47659338 EMD-386088 Serotonin receptor agonist

3052 0 cp BRD-K47192521 icosapent Platelet aggregation inhibitor

3053 0 cp BRD-K80325895 eicosadienoic-acid -666

3054 0 cp BRD-A99571536 dubinidine Anti-epileptic

3055 0 cp BRD-K54233340 dorsomorphin AMPK inhibitor

3056 0 cp BRD-K93441486 diphemanil Acetylcholine receptor antagonist

3057 0 cp BRD-K18518344 digitoxigenin ATPase inhibitor

3058 0 cp BRD-K60274257 dephostatin Tyrosine phosphatase inhibitor

3059 0 cp BRD-A49160188 donepezil Acetylcholinesterase inhibitor

3060 0 cp BRD-K38305202 domperidone Dopamine receptor antagonist

3061 0 cp BRD-A69636825 diltiazem Calcium channel blocker

3062 0 cp BRD-A65076780 dihydroergocristine Adrenergic receptor antagonist

3063 0 cp BRD-A02713983 dihydrodeoxygedunin Growth factor receptor activator

3064 0 cp BRD-A47144777 dihydro-7-desacetyldeoxygedunin HSP inhibitor

3065 0 cp BRD-K20152659 gamma-homolinolenic-acid Cholesterol inhibitor

3066 0 cp BRD-K45330754 diethylstilbestrol Estrogen receptor agonist

3067 0 cp BRD-K45542189 diethylcarbamazine Lipoxygenase inhibitor

3068 0 cp BRD-K95309561 dienestrol Estrogen receptor agonist

3069 0 cp BRD-K08252256 diclofenac Cyclooxygenase inhibitor

3070 0 cp BRD-K01555864 dibenzoylmethane Antineoplastic

3071 0 cp BRD-K13819402 desoxypeganine Acetylcholinesterase inhibitor

3072 0 cp BRD-K68558722 deracoxib Cyclooxygenase inhibitor

3073 0 cp BRD-A17819071 gedunin HSP inhibitor

3074 0 cp BRD-A39255369 DCPIB Chloride channel blocker

3075 0 cp BRD-K43389675 daunorubicin RNA synthesis inhibitor

3076 0 cp BRD-A92537424 danazol Estrogen receptor antagonist

3077 0 cp BRD-K06467078 corynanthine Adrenergic receptor antagonist

3078 0 cp BRD-K43736954 cortisone Glucocorticoid receptor agonist

3079 0 cp BRD-K55677650 CO-101244 Ionotropic glutamate receptor antagonist

3080 0 cp BRD-K17294426 clebopride Dopamine receptor antagonist

3081 0 cp BRD-A83695761 chromanol Potassium channel blocker

3082 0 cp BRD-K32828673 chelidonine Tubulin inhibitor

3083 0 cp BRD-A64933752 CV-1808 Adenosine receptor agonist

3084 0 cp BRD-K23913458 coumarin Vitamin K antagonist

3085 0 cp BRD-A54487287 cortisone Glucocorticoid receptor agonist

3086 0 cp BRD-A55416093 colforsin Adenylyl cyclase activator

3087 0 cp BRD-K45252063 clofibrate PPAR receptor agonist

3088 0 cp BRD-A26095496 clobetasol Glucocorticoid receptor agonist

3089 0 cp BRD-K30240666 clemastine Histamine receptor antagonist

3090 0 cp BRD-K14693417 cinchonine P-glycoprotein inhibitor

3091 0 cp BRD-K40901640 cinanserin Serotonin receptor antagonist

3092 0 cp BRD-K67017579 cilostazol Phosphodiesterase inhibitor

3093 0 cp BRD-K17075857 chloroxine Opioid receptor antagonist

3094 0 cp BRD-A91699651 chloroquine Antimalarial

3095 0 cp BRD-K42500029 CGP-57380 MAP kinase inhibitor

3096 0 cp BRD-A10977446 carvedilol Adrenergic receptor antagonist

3097 0 cp BRD-K41707108 ceramide Phosphoenolpyruvate carboxylase activator

3098 0 cp BRD-A82238138 budesonide Glucocorticoid receptor agonist

3099 0 cp BRD-K49448285 bisindolylmaleimide CDK inhibitor

3100 0 cp BRD-K14221570 benzopurpurin-4b HIV entry inhibitor

3101 0 cp BRD-K43468059 byssochlamic-acid Mycotoxin

3102 0 cp BRD-A22713669 BVT-948 Tyrosine phosphatase inhibitor

3103 0 cp BRD-K08287586 butylparaben DNA synthesis inhibitor

3104 0 cp BRD-A05186015 bupropion Dopamine uptake inhibitor

3105 0 cp BRD-K56115039 BU-226 Imidazoline receptor ligand

3106 0 cp BRD-K47631482 bromhexine Mucolytic agent

3107 0 cp BRD-A37347161 BRL-52537 Opioid receptor agonist

3108 0 cp BRD-K56509348 BMS-182874 Endothelin receptor antagonist

3109 0 cp BRD-A92177080 betamethasone Glucocorticoid receptor agonist

3110 0 cp BRD-A02180903 betamethasone Glucocorticoid receptor agonist

3111 0 cp BRD-K70327191 benzoxiquine Anti-infective

3112 0 cp BRD-A80017228 bendroflumethiazide Sodium/potassium/chloride transporter inhibitor

3113 0 cp BRD-K04877770 FTase-inhibitor-B581 Farnesyltransferase inhibitor

3114 0 cp BRD-A84493640 atovaquone Mitochondrial electron transport inhibitor

3115 0 cp BRD-A66199457 asiaticoside Antineoplastic

3116 0 cp BRD-A09062839 amylocaine Local anesthetic

3117 0 cp BRD-K52397688 amperozide Dopamine receptor antagonist

3118 0 cp BRD-K50214219 CS-1657 PARP inhibitor

3119 0 cp BRD-K56558538 ambroxol Sodium channel blocker

3120 0 cp BRD-K93080877 Ala-Ala-Phe-CMK Tripeptidyl peptidase inhibitor

3121 0 cp BRD-A72758037 asiatic-acid Apoptosis stimulant

3122 0 cp BRD-K93188295 ARC-239 Adrenergic receptor antagonist

3123 0 cp BRD-K46937689 phenazone Cyclooxygenase inhibitor

3124 0 cp BRD-K98490050 amsacrine Topoisomerase inhibitor

3125 0 cp BRD-K17561142 amiodarone Potassium channel blocker

3126 0 cp BRD-K43068349 AMG-9810 TRPV antagonist

3127 0 cp BRD-K59419204 AM-281 Cannabinoid receptor antagonist

3128 0 cp BRD-A27554692 altrenogest Progestogen hormone

3129 0 cp BRD-K35498378 alrestatin Aldose reductase inhibitor

3130 0 cp BRD-A90131694 alclometasone Glucocorticoid receptor agonist

3131 0 cp BRD-A14966924 alaproclate Serotonin receptor antagonist

3132 0 cp BRD-K03670461 tyrphostin-AG-82 EGFR inhibitor

3133 0 cp BRD-K43405658 tyrphostin-AG-527 Protein tyrosine kinase inhibitor

3134 0 cp BRD-K62929068 6-benzylaminopurine Purinergic receptor activator

3135 0 cp BRD-K20482099 rutin Antioxidant

3136 0 cp BRD-A07824748 flavanone 11-beta-HSD1 inhibitor

3137 0 cp BRD-A85280935 quinpirole Dopamine receptor agonist

7152 0 cp BRD-K64052750 gefitinib EGFR inhibitor

7153 0 cp BRD-A45498368 WYE-125132 MTOR inhibitor

7154 0 cp BRD-K51313569 palbociclib CDK inhibitor

7155 0 cp BRD-K83963101 MLN-8054 Aurora kinase inhibitor

7156 0 cp BRD-A29901043 KIN001-127 ITK inhibitor

7157 0 cp BRD-K04887706 AKT-inhibitor-1-2 AKT inhibitor

7158 0 cp BRD-K68407802 KIN001-055 EGFR inhibitor

7159 0 cp BRD-K92428232 GSK-461364 PLK inhibitor

7160 0 cp BRD-K36740062 GSK-1070916 Aurora kinase inhibitor

7161 0 cp BRD-K52911425 GDC-0941 PI3K inhibitor

7162 0 cp BRD-K15592317 CP466722 ATM kinase inhibitor

7163 0 cp BRD-K69932463 AZD-8055 MTOR inhibitor

7164 0 cp BRD-K46056750 AZD-7762 CHK inhibitor

7165 0 cp BRD-K68191783 ALW-II-38-3 Ephrin inhibitor

7166 0 cp BRD-K93918653 quizartinib FLT3 inhibitor

7167 0 cp BRD-K59369769 tozasertib Aurora kinase inhibitor

7168 0 cp BRD-K56343971 vemurafenib RAF inhibitor

7169 0 cp BRD-K87909389 alvocidib CDK inhibitor

7170 0 cp BRD-K07691486 roscovitine CDK inhibitor

7171 0 cp BRD-K00627859 tubastatin-a HDAC inhibitor

7172 0 cp BRD-K29313308 HDAC3-selective HDAC inhibitor

7173 0 cp BRD-K88742110 BRD-K88742110 HDAC inhibitor

7174 0 cp BRD-K50417881 eticlopride Dopamine receptor antagonist

7175 0 cp BRD-K73196317 urapidil Adrenergic receptor antagonist

7176 0 cp BRD-A95939040 sertaconazole Sterol demethylase inhibitor

7177 0 cp BRD-A07765530 epinephrine carbonic anhydrase activator

7178 0 cp BRD-K13032584 procarbazine Monoamine oxidase inhibitor

7179 0 cp BRD-A33447119 oxfendazole Anthelmintic

7180 0 cp BRD-A36267905 buphenine Adrenergic receptor agonist

7181 0 cp BRD-K67080878 milrinone Phosphodiesterase inhibitor

7182 0 cp BRD-K65417056 meprylcaine Local anesthetic

7183 0 cp BRD-A83937277 mephenytoin Hydantoin antiepileptic

7184 0 cp BRD-K33453211 levocabastine Histamine receptor antagonist

7185 0 cp BRD-A44090213 indoprofen Cyclooxygenase inhibitor

7186 0 cp BRD-K46424862 hymecromone Monoamine oxidase inhibitor

7187 0 cp BRD-K80396088 gliquidone Sulfonylurea

7188 0 cp BRD-A74980173 gatifloxacin Bacterial DNA gyrase inhibitor

7189 0 cp BRD-A73368467 fexofenadine Histamine receptor antagonist

7190 0 cp BRD-A16311756 profenamine Butyrylcholinesterase inhibitor

7191 0 cp BRD-K22193694 dioxybenzone Topical sunscreen agent

7192 0 cp BRD-A77291778 cyclopentolate Acetylcholine receptor antagonist

7193 0 cp BRD-A29485665 bicalutamide Androgen receptor antagonist

7194 0 cp BRD-K14993104 bemegride Chemoreceptor agonist

7195 0 cp BRD-K63979671 etifenin Compound used in hepatobiliary scans of the liver

7196 0 cp BRD-K38055836 etamivan Respiratory stimulant

7197 0 cp BRD-K19111024 clofibric-acid PPAR receptor agonist

7198 0 cp BRD-K77925998 quipazine Serotonin receptor agonist

7199 0 cp BRD-A46393198 tetramisole Immunostimulant

7200 0 cp BRD-K86204871 terconazole Sterol demethylase inhibitor

7201 0 cp BRD-K15933101 ropinirole Dopamine receptor agonist

7202 0 cp BRD-K76304753 phenazopyridine Local anesthetic

7203 0 cp BRD-K93280214 gabazine GABA receptor antagonist

7204 0 cp BRD-K79145749 dibenzepin Norepinephrine reuptake inhibitor

7205 0 cp BRD-A68723818 brompheniramine Histamine receptor antagonist

7206 0 cp BRD-A60197193 amisulpride Dopamine receptor antagonist

7207 0 cp BRD-K32164935 tolazamide ATP channel blocker

7208 0 cp BRD-K50938287 sumatriptan Serotonin receptor agonist

7209 0 cp BRD-K13571841 pepstatin Aspartic protease inhibitor

7210 0 cp BRD-K28912512 nicotinamide Protein synthesis stimulant

7211 0 cp BRD-K79425933 benperidol Dopamine receptor antagonist

7212 0 cp BRD-K36616567 doxepin Histamine receptor antagonist

7213 0 cp BRD-A23072235 pheniramine Histamine receptor antagonist

7214 0 cp BRD-A75552914 isoxicam Cyclooxygenase inhibitor

7215 0 cp BRD-K50388907 fenofibrate PPAR receptor agonist

7216 0 cp BRD-K12513978 fenbufen Cyclooxygenase inhibitor

7217 0 cp BRD-K63550407 erythromycin NFkB pathway inhibitor

7218 0 cp BRD-K68507560 dicycloverine Acetylcholine receptor antagonist

7219 0 cp BRD-A26384407 chlortalidone Carbonic anhydrase inhibitor

7220 0 cp BRD-A39230911 chlorphensin Muscle relaxant

7221 0 cp BRD-K97061094 azacyclonol Histamine receptor antagonist

7222 0 cp BRD-K55044200 amoxicillin Penicillin binding protein inhibitor

7223 0 cp BRD-K32318651 acyclovir DNA polymerase inhibitor

7224 0 cp BRD-A48430263 pioglitazone Insulin sensitizer

7225 0 cp BRD-A97701745 pindolol Adrenergic receptor antagonist

7226 0 cp BRD-K77641333 naphazoline Adrenergic receptor agonist

7227 0 cp BRD-K20655524 mefexamide Psychoactive drug

7228 0 cp BRD-K06926592 tretinoin Retinoid receptor agonist

7229 0 cp BRD-K55127134 fluphenazine Dopamine receptor antagonist

7230 0 cp BRD-A84481105 thioridazine Dopamine receptor antagonist

7231 0 cp BRD-A53952395 prilocaine Local anesthetic

7232 0 cp BRD-A55815733 phylloquinone Vitamin K

7233 0 cp BRD-K15262564 mupirocin Isoleucyl-tRNA synthetase inhibitor

7234 0 cp BRD-K12219985 glipizide Sulfonylurea

7235 0 cp BRD-K63630713 etacrynic-acid Sodium/potassium/chloride transporter inhibitor

7236 0 cp BRD-A75368507 demeclocycline Bacterial 30S ribosomal subunit inhibitor

7237 0 cp BRD-K29458283 chlorambucil DNA inhibitor

7238 0 cp BRD-K93433262 alfacalcidol Vitamin D receptor agonist

7239 0 cp BRD-A94793051 gestrinone Progesterone receptor antagonist

7240 0 cp BRD-A24228527 ofloxacin Bacterial DNA gyrase inhibitor

7241 0 cp BRD-A80638690 floxuridine DNA synthesis inhibitor

7242 0 cp BRD-K23204545 busulfan DNA inhibitor

7243 0 cp BRD-A16478930 amcinonide Glucocorticoid receptor agonist

7244 0 cp BRD-K02123250 JNJ-38877605 Tyrosine kinase inhibitor

7245 0 cp BRD-K06335600 tizanidine Adrenergic receptor agonist

7246 0 cp BRD-A13946108 sulindac Cyclooxygenase inhibitor

7247 0 cp BRD-A88774919 doxycycline Bacterial 30S ribosomal subunit inhibitor

7248 0 cp BRD-A25067867 benzatropine Acetylcholine receptor antagonist

7249 0 cp BRD-K70976396 cefoxitin Bacterial cell wall synthesis inhibitor

7250 0 cp BRD-A20239487 atenolol Adrenergic receptor antagonist

7251 0 cp BRD-K94830329 ataluren CFTR channel agonist

7252 0 cp BRD-K78373679 RO-3306 CDK inhibitor

7253 0 cp BRD-K74763371 bosentan Endothelin receptor antagonist

7254 0 cp BRD-K01612348 meropenem Bacterial cell wall synthesis inhibitor

7255 0 cp BRD-A51382177 fosinopril ACE inhibitor

7256 0 cp BRD-A04308630 genipin Choleretic agent

7257 0 cp BRD-K72903603 zidovudine Reverse transcriptase inhibitor

7258 0 cp BRD-A99833829 bethanechol Acetylcholine receptor agonist

7259 0 cp BRD-K60762818 desipramine Tricyclic antidepressant

7260 0 cp BRD-K57631554 aminolevulinic-acid Oxidizing agent

7261 0 cp BRD-K67102207 phenylbutyrate HDAC inhibitor

7262 0 cp BRD-K11433652 aspirin Cyclooxygenase inhibitor

7263 0 cp BRD-K89348303 ramipril ACE inhibitor

7264 0 cp BRD-A79803969 memantine Glutamate receptor antagonist

7265 0 cp BRD-K08273968 griseofulvin Tubulin inhibitor

7266 0 cp BRD-K44497846 enalapril ACE inhibitor

7267 0 cp BRD-A13188892 doxazosin Adrenergic receptor antagonist

7268 0 cp BRD-K88868628 iodoacetic-acid Cysteine peptidase inhibitor

7269 0 cp BRD-K71289571 zafirlukast Leukotriene receptor antagonist

7270 0 cp BRD-K49519144 LY-2140023 Glutamate receptor agonist

7271 0 cp BRD-K35458079 edaravone Nootropic agent

7272 0 cp BRD-A87848830 bimatoprost Prostanoid receptor agonist

7273 0 cp BRD-A36217750 sulfinpyrazone Uricosuric blocker

7274 0 cp BRD-K90027355 spironolactone Mineralocorticoid receptor antagonist

7275 0 cp BRD-K75089421 procainamide Sodium channel blocker

7276 0 cp BRD-K90885812 propantheline Acetylcholine receptor antagonist

7277 0 cp BRD-K73978287 hydrocortisone Glucocorticoid receptor agonist

7278 0 cp BRD-A86044036 flurbiprofen Cyclooxygenase inhibitor

7279 0 cp BRD-A65282128 cefazolin Bacterial cell wall synthesis inhibitor

7280 0 cp BRD-K96037667 norethindrone Progesterone receptor agonist

7281 0 cp BRD-K90543092 levonorgestrel Estrogen receptor agonist

7282 0 cp BRD-K88568253 iproniazid Monoamine oxidase inhibitor

7283 0 cp BRD-A79237180 ascorbic-acid Antioxidant

7284 0 cp BRD-A67605442 tetrahydrobiopterin Nitric oxide stimulant

7285 0 cp BRD-A31312900 montelukast Leukotriene receptor antagonist

7286 0 cp BRD-A91555231 norepinephrine Adrenergic receptor agonist

7287 0 cp BRD-K92049597 triamterene Sodium channel blocker

7288 0 cp BRD-K50859149 sulfafurazole Bacterial antifolate

7289 0 cp BRD-K79116891 proxymetacaine Sodium channel blocker

7290 0 cp BRD-K95237249 probenecid Uricosuric blocker

7291 0 cp BRD-K86434416 selegiline Monoamine oxidase inhibitor

7292 0 cp BRD-K53790871 triamcinolone Glucocorticoid receptor agonist

7293 0 cp BRD-K10016611 pyridine-2-aldoxime Acetylcholinesterase inhibitor

7294 0 cp BRD-K40645748 mefloquine Adenosine receptor antagonist

7295 0 cp BRD-K57886322 fluocinonide Glucocorticoid receptor agonist

7296 0 cp BRD-K68132782 terbinafine Fungal squalene epoxidase inhibitor

7297 0 cp BRD-K11801786 trimidox Ribonucleotide reductase inhibitor

7298 0 cp BRD-K21520694 sulfacetamide PABA antagonist

7299 0 cp BRD-K38197229 bumetanide Solute carrier family member inhibitor

7300 0 cp BRD-A23290232 westcort Glucocorticoid receptor agonist

7301 0 cp BRD-A07780951 orciprenaline Adrenergic receptor agonist

7302 0 cp BRD-K91601245 mercaptopurine Immunosuppressant

7303 0 cp BRD-A17655518 ibuprofen Cyclooxygenase inhibitor

7304 0 cp BRD-K22662435 ganciclovir DNA polymerase inhibitor

7305 0 cp BRD-K63828191 raloxifene Estrogen receptor antagonist

7306 0 cp BRD-K81128206 edrophonium Acetylcholinesterase inhibitor

7307 0 cp BRD-K87226815 cycloserine Bacterial cell wall synthesis inhibitor

7308 0 cp BRD-A09722536 cyclophosphamide DNA alkylating agent

7309 0 cp BRD-K20920669 cromoglicic-acid Immunosuppressant

7310 0 cp BRD-K63641886 cefuroxime Bacterial cell wall synthesis inhibitor

7311 0 cp BRD-A16754160 ampicillin Bacterial cell wall synthesis inhibitor

7312 0 cp BRD-A29260609 acebutolol Adrenergic receptor antagonist

7313 0 cp BRD-K98763141 niflumic-acid Cyclooxygenase inhibitor

7314 0 cp BRD-K81473089 tacrine Acetylcholinesterase inhibitor

7315 0 cp BRD-K71926323 marbofloxacin Bacterial DNA gyrase inhibitor

7316 0 cp BRD-K66788707 fludarabine DNA synthesis inhibitor

7317 0 cp BRD-K55301415 abiraterone "17,20 lyase inhibitor"

7318 0 cp BRD-K37194137 III606050 Cytochrome P450 inhibitor

7319 0 cp BRD-K35573744 erbstatin-analog EGFR inhibitor

7320 0 cp BRD-K34098590 tienilic-acid Sodium/potassium/chloride transporter inhibitor

7321 0 cp BRD-K15502390 nevirapine Reverse transcriptase inhibitor

7322 0 cp BRD-A80793822 pemoline Dopamine receptor agonist

7323 0 cp BRD-A64479082 quinidine Sodium channel blocker

7324 0 cp BRD-A29644307 nomifensine Dopamine uptake inhibitor

7325 0 cp BRD-A25576662 streptozotocin DNA alkylating agent

7326 0 cp BRD-K98426715 tubacin HDAC inhibitor

7327 0 cp BRD-A37837077 cyclazosin Adrenergic receptor antagonist

7328 0 cp BRD-K17823458 danoprevir HCV inhibitor

7329 0 cp BRD-K97764662 PD-173074 FGFR inhibitor

7330 0 cp BRD-K72703948 ZM-447439 Aurora kinase inhibitor

7331 0 cp BRD-K40255344 tyrphostin-A9 Protein tyrosine kinase inhibitor

7332 0 cp BRD-K24994810 androstenol GABA receptor modulator

7333 0 cp BRD-K32836707 CAY-10577 Casein kinase inhibitor

7334 0 cp BRD-K71512533 SNS-314 Aurora kinase inhibitor

7335 0 cp BRD-K92723993 imatinib BCR-ABL kinase inhibitor

7336 0 cp BRD-K50140147 NVP-TAE684 ALK inhibitor

7337 0 cp BRD-A32161980 carbetocin Oxytocin receptor agonist

7338 0 cp BRD-A02333338 cyclopamine Smoothened receptor antagonist

7339 0 cp BRD-K55991774 BAS-09104376 HIV integrase inhibitor

7340 0 cp BRD-A45664787 iloprost Platelet aggregation inhibitor

7341 0 cp BRD-K22096725 ALW-II-49-7 Ephrin inhibitor

7342 0 cp BRD-K31283835 tofacitinib JAK inhibitor

7343 0 cp BRD-K19416115 sitagliptin Dipeptidyl peptidase inhibitor

7344 0 cp BRD-K31553034 zibotentan Endothelin receptor antagonist

7345 0 cp BRD-K64881305 ispinesib Kinesin-like spindle protein inhibitor

7346 0 cp BRD-K35687265 ON-01910 PLK inhibitor

7347 0 cp BRD-K71823332 epothilone-a Microtubule stabilizing agent

7348 0 cp BRD-K07881437 danusertib Aurora kinase inhibitor

7349 0 cp BRD-K29733039 deforolimus MTOR inhibitor

7350 0 cp BRD-K97056771 HY-11007 BCR-ABL kinase inhibitor

7351 0 cp BRD-K56001384 antimycin-a ATP synthase inhibitor

7352 0 cp BRD-K00656370 6-aminochrysene Transferase inhibitor

7353 0 cp BRD-K07403598 CAY-10470 NFkB pathway inhibitor

7354 0 cp BRD-K26818574 BIX-01294 Histone lysine methyltransferase inhibitor

7355 0 cp BRD-A01826957 xanthinol Vasodilator

7356 0 cp BRD-A75769826 SDM25N Opioid receptor antagonist

7357 0 cp BRD-K00234327 RU-24969 Serotonin receptor agonist

7358 0 cp BRD-K67637637 olopatadine Histamine receptor antagonist

7359 0 cp BRD-K92000912 AM-251 Cannabinoid receptor antagonist

7360 0 cp BRD-A81233518 glycopyrrolate Acetylcholine receptor antagonist

7361 0 cp BRD-K05673000 dicloxacillin Bacterial cell wall synthesis inhibitor

7362 0 cp BRD-K27721098 clopidogrel Purinergic receptor antagonist

7363 0 cp BRD-A47598013 citalopram Serotonin reuptake inhibitor

7364 0 cp BRD-A69512159 carbidopa Aromatic L-amino acid decarboxylase inhibitor

7365 0 cp BRD-A34208323 VU-0404997-2 Glutamate receptor modulator

7366 0 cp BRD-K39823328 VU-0366037-2 Glutamate receptor modulator

7367 0 cp BRD-K30649484 mafenide Carbonic anhydrase inhibitor

7368 0 cp BRD-K68756823 FR-180204 -666

7369 0 cp BRD-A19500257 geldanamycin HSP inhibitor

7370 0 cp BRD-K44432556 VU-0418946-1 HIF modulator

7371 0 cp BRD-K37456065 VU-0365114-2 M5 modulator

7372 0 cp BRD-K50128260 sildenafil Phosphodiesterase inhibitor

7373 0 cp BRD-K23875128 RHO-kinase-inhibitor-III[rockout] Rho associated kinase inhibitor

7374 0 cp BRD-K76205745 losartan Angiotensin receptor antagonist

7375 0 cp BRD-K69328504 L-690488 Inositol monophosphatase inhibitor

7376 0 cp BRD-K28470988 L-690330 Inositol monophosphatase inhibitor

7377 0 cp BRD-K09499853 KU-0060648 DNA dependent protein kinase inhibitor

7378 0 cp BRD-K78959463 FPL-64176 Calcium channel activator

7379 0 cp BRD-A13122391 triptolide RNA polymerase inhibitor

7380 0 cp BRD-K12762134 XAV-939 Tankyrase inhibitor

7381 0 cp BRD-K09907482 PRL-3-inhibitor-I Tyrosine phosphatase inhibitor

7382 0 cp BRD-K08703257 3-amino-benzamide PARP inhibitor

7383 0 cp BRD-K88560311 rucaparib PARP inhibitor

7384 0 cp BRD-A75517195 thiazolopyrimidine CDC inhibitor

7385 0 cp BRD-K82135108 elesclomol Oxidative stress inducer

7386 0 cp BRD-K35723520 darinaparsin Apoptosis stimulant

7387 0 cp BRD-A38030642 cyclosporin-a Calcineurin inhibitor

7388 0 cp BRD-A85860691 chaetocin Histone lysine methyltransferase inhibitor

7389 0 cp BRD-K71799949 carbamazepine Carboxamide antiepileptic

7390 0 cp BRD-K80527266 triacsin-c Adrenergic receptor antagonist

7391 0 cp BRD-A97437073 rosiglitazone Insulin sensitizer

7392 0 cp BRD-K21672174 RO-28-1675 Glucokinase activator

7393 0 cp BRD-A45889380 mepacrine Cytokine production inhibitor

7394 0 cp BRD-K11853856 PJ-34 PARP inhibitor

7395 0 cp BRD-A68631409 evodiamine ATPase inhibitor

7396 0 cp BRD-A67373739 AICA-ribonucleotide AMPK activator

7397 0 cp BRD-K93176058 AC-55649 Retinoid receptor agonist

7398 0 cp BRD-K54256913 MK-1775 WEE1 kinase inhibitor

7399 0 cp BRD-K53414658 tivozanib VEGFR inhibitor

7400 0 cp BRD-K36927236 glibenclamide Sulfonylurea

7401 0 cp BRD-K32536677 AGK-2 SIRT inhibitor

7402 0 cp BRD-K26664453 cytochalasin-b Microtubule inhibitor

7403 0 cp BRD-K00317371 RITA MDM inhibitor

7404 0 cp BRD-K89732114 trifluoperazine Dopamine receptor antagonist

7405 0 cp BRD-A47513740 calyculin Protein phosphatase inhibitor

7406 0 cp BRD-K47207162 zimelidine Serotonin reuptake inhibitor

7407 0 cp BRD-K48427617 U-0124 MEK inhibitor

7408 0 cp BRD-A79672927 tropicamide Acetylcholine receptor antagonist

7409 0 cp BRD-K11742128 triprolidine Histamine receptor antagonist

7410 0 cp BRD-K46211610 tolazoline Adrenergic receptor antagonist

7411 0 cp BRD-K55748775 SCH-28080 ATPase inhibitor

7412 0 cp BRD-K86600316 RS-79948 Adrenergic receptor antagonist

7413 0 cp BRD-A59303141 quinethazone Thiazide diuretic

7414 0 cp BRD-A19736161 ondansetron Serotonin receptor antagonist

7415 0 cp BRD-K26979635 NS-3694 Glutamate receptor antagonist

7416 0 cp BRD-K62056274 quipazine Serotonin receptor agonist

7417 0 cp BRD-A45543382 metrizamide Radiopaque medium

7418 0 cp BRD-K02867583 minaprine Serotonin reuptake inhibitor

7419 0 cp BRD-A68039575 liquiritigenin Aromatase inhibitor

7420 0 cp BRD-K13211965 L-741742 Dopamine receptor antagonist

7421 0 cp BRD-A52326238 isogedunin HSP inhibitor

7422 0 cp BRD-K86509404 iso-olomoucine CDK inhibitor

7423 0 cp BRD-A95869247 indapamide Thiazide diuretic

7424 0 cp BRD-K38436528 imipramine Norepinephrine reuptake inhibitor

7425 0 cp BRD-A37776212 ICI-204448 Opioid receptor agonist

7426 0 cp BRD-A73859745 glycodeoxycholic-acid Apoptosis stimulant

7427 0 cp BRD-K26117720 gingerol Nitric oxide synthase inhibitor

7428 0 cp BRD-K11129031 gemfibrozil Lipoprotein lipase activator

7429 0 cp BRD-A13133631 fluorometholone Glucocorticoid receptor agonist

7430 0 cp BRD-A02367930 ethinyl-estradiol DNA directed DNA polymerase stimulant

7431 0 cp BRD-K45117373 Y-26763 Potassium channel activator

7432 0 cp BRD-A19195498 trimipramine Norepinephrine reuptake inhibitor

7433 0 cp BRD-A09094913 strychnine Acetylcholine receptor antagonist

969 0.01 cp BRD-A41450521 tosufloxacin Bacterial DNA gyrase inhibitor

970 0.01 cp BRD-K53857191 risperidone Dopamine receptor antagonist

971 0.01 cp BRD-K22031190 diflunisal Prostanoid receptor antagonist

942 0.02 cp BRD-K46435977 valaciclovir DNA polymerase inhibitor

943 0.02 cp BRD-K54314721 zolmitriptan Serotonin receptor agonist

944 0.02 cp BRD-K64994968 progesterone Progesterone receptor agonist

945 0.02 cp BRD-K34776109 glimepiride Insulin secretagogue

946 0.02 cp BRD-K57222227 indometacin Cyclooxygenase inhibitor

947 0.02 cp BRD-K62200014 anagrelide Phosphodiesterase inhibitor

948 0.02 cp BRD-A58048407 nimodipine Calcium channel blocker

949 0.02 cp BRD-K77987382 mebendazole Tubulin inhibitor

950 0.02 cp BRD-A36074203 remacemide Glutamate receptor antagonist

951 0.02 cp BRD-K56301217 ABT-737 BCL inhibitor

932 0.03 cp BRD-K49668410 clarithromycin Bacterial 50S ribosomal subunit inhibitor

933 0.03 cp BRD-K68065987 MK-2206 AKT inhibitor

934 0.03 cp BRD-K06147391 telenzepine Acetylcholine receptor antagonist

935 0.03 cp BRD-K78633253 EXO-1 ARF inhibitor

936 0.03 cp BRD-A16694057 bisphenol-a PPAR receptor antagonist

937 0.03 cp BRD-A31159102 fluoxetine Selective serotonin reuptake inhibitor (SSRI)

938 0.03 cp BRD-K97752965 nicorandil Nitric oxide donor

939 0.03 cp BRD-A67799922 phenoxybenzamine Adrenergic receptor antagonist

940 0.03 cp BRD-K67783091 haloperidol Dopamine receptor antagonist

929 0.04 cp BRD-K18909381 CGS-12066B Serotonin receptor agonist

930 0.04 cp BRD-K65639003 icariin Phosphodiesterase inhibitor

931 0.04 cp BRD-A54880345 etomidate GABA receptor modulator

928 0.05 cp BRD-K68264559 brimonidine Adrenergic receptor agonist

926 0.06 cp BRD-K98530306 clonidine Adrenergic receptor agonist

914 0.07 cp BRD-K49481516 galantamine Acetylcholinesterase inhibitor

915 0.07 cp BRD-K59574735 ubenimex Leukotriene inhibitor

916 0.07 cp BRD-K06854232 AM-580 Retinoid receptor agonist

917 0.07 cp BRD-K34533029 tyrphostin-AG-494 EGFR inhibitor

918 0.07 cp BRD-A64977602 mirtazapine Adrenergic receptor antagonist

919 0.07 cp BRD-A29082194 gitoxigenin ATPase inhibitor

920 0.07 cp BRD-K91699951 benzonatate Local anesthetic

921 0.07 cp BRD-A35108200 dexamethasone Glucocorticoid receptor agonist

922 0.07 cp BRD-K62810658 PD-98059 MEK inhibitor

923 0.07 cp BRD-K06593056 LE-135 Retinoid receptor agonist

924 0.07 cp BRD-K63675182 triflupromazine Dopamine receptor antagonist

910 0.1 cp BRD-K81916719 triclabendazole Microtubule inhibitor

911 0.1 cp BRD-K16444452 ibudilast Leukotriene receptor antagonist

899 0.11 cp BRD-K57033106 tripelennamine Histamine receptor antagonist

900 0.11 cp BRD-K93332168 isocarboxazid Monoamine oxidase inhibitor

901 0.11 cp BRD-K52989797 clomipramine Serotonin transporter inhibitor (SERT)

902 0.11 cp BRD-K87158025 benzamil Sodium channel blocker

903 0.11 cp BRD-A11678676 wortmannin PI3K inhibitor

904 0.11 cp BRD-K00603606 ticlopidine Purinergic receptor antagonist

905 0.11 cp BRD-K56429665 calcipotriol Vitamin D receptor agonist

906 0.11 cp BRD-A65671304 candesartan Angiotensin receptor antagonist

907 0.11 cp BRD-K37991163 paroxetine Selective serotonin reuptake inhibitor (SSRI)

908 0.11 cp BRD-A25234499 aminoglutethimide Glucocorticoid receptor antagonist

909 0.11 cp BRD-A09533288 verapamil Calcium channel blocker

890 0.14 cp BRD-K00615600 AG-14361 PARP inhibitor

891 0.14 cp BRD-K61323504 SB-225002 CC chemokine receptor antagonist

892 0.14 cp BRD-K67100011 pivmecillinam Bacterial cell wall synthesis inhibitor

893 0.14 cp BRD-A04756508 norgestimate Progesterone receptor agonist

894 0.14 cp BRD-K64245000 GW-4064 FXR agonist

895 0.14 cp BRD-K96084870 DMBI PDGFR receptor inhibitor

896 0.14 cp BRD-K10974103 diloxanide Protein synthesis inhibitor

897 0.14 cp BRD-K77695569 tiabendazole Angiogenesis inhibitor

898 0.14 cp BRD-A50311610 meclozine CAR agonist

886 0.18 cp BRD-A24191444 ifenprodil Adrenergic receptor antagonist

887 0.18 cp BRD-K17378184 prestwick-559 Dopamine receptor agonist

888 0.18 cp BRD-K73395020 SA-1478088 -666

889 0.18 cp BRD-K67847053 guanabenz Adrenergic receptor agonist

880 0.21 cp BRD-K70358946 aripiprazole Serotonin receptor agonist

881 0.21 cp BRD-K74305673 IKK-2-inhibitor-V IKK inhibitor

882 0.21 cp BRD-K86301799 dipyridamole Phosphodiesterase inhibitor

883 0.21 cp BRD-K19540840 saracatinib SRC inhibitor

884 0.21 cp BRD-K02407574 parbendazole Tubulin inhibitor

879 0.22 cp BRD-K09186807 KIN001-244 Phosphoinositide dependent kinase inhibitor

873 0.25 cp BRD-K25079130 avrainvillamide-analog-4 nucleophosmin inhibitor

874 0.25 cp BRD-A88254928 salbutamol Adrenergic receptor agonist

875 0.25 cp BRD-K71499074 diclofenamide Carbonic anhydrase inhibitor

876 0.25 cp BRD-K50311478 tosyl-phenylalanyl-chloromethyl-ketone Chymotrypsin inhibitor

877 0.25 cp BRD-K51290057 SA-792709 Retinoid receptor agonist

878 0.25 cp BRD-K82746043 navitoclax BCL inhibitor

866 0.28 cp BRD-A96107863 nisoldipine Calcium channel blocker

867 0.28 cp BRD-K15409150 penfluridol T-type calcium channel blocker

868 0.28 cp BRD-K86958018 olvanil TRPV agonist

869 0.28 cp BRD-K98769987 flumazenil Benzodiazepine receptor antagonist

870 0.28 cp BRD-A50684349 fenoldopam Dopamine receptor agonist

871 0.28 cp BRD-K33459542 ditolylguanidine Sigma receptor agonist

872 0.28 cp BRD-K03557653 sappanone-a Tyrosinase inhibitor

865 0.29 cp BRD-K50422030 clomethiazole GABA receptor antagonist

860 0.32 cp BRD-A44133049 azasetron Serotonin receptor antagonist

861 0.32 cp BRD-A36318220 necrostatin-1 RIPK inhibitor

862 0.32 cp BRD-A26032986 zaldaride Calmodulin antagonist

863 0.32 cp BRD-A33711280 metixene Acetylcholine receptor antagonist

864 0.32 cp BRD-K12102668 nialamide Monoamine oxidase inhibitor

858 0.33 cp BRD-A07395371 esmolol Adrenergic receptor antagonist

848 0.35 cp BRD-K42573370 avrainvillamide-analog-2 nucleophosmin inhibitor

849 0.35 cp BRD-K99411983 lumicolchicine "Colchicine isomer, non-binder of microtubules"

850 0.35 cp BRD-A42167015 carteolol Adrenergic receptor antagonist

851 0.35 cp BRD-K42679050 Y-27152 Potassium channel activator

852 0.35 cp BRD-K29673530 hypericin Tyrosine kinase inhibitor

853 0.35 cp BRD-K74212935 ergocryptine Dopamine agonist

854 0.35 cp BRD-A15131297 benazepril ACE inhibitor

855 0.35 cp BRD-K32247306 primidone GABA receptor antagonist

841 0.39 cp BRD-A43882281 pinacidil ATP channel activator

842 0.39 cp BRD-K31611373 fluprostenol Prostanoid receptor agonist

843 0.39 cp BRD-A70514680 articaine Local anesthetic

844 0.39 cp BRD-K85090592 pilocarpine Acetylcholine receptor agonist

845 0.39 cp BRD-A87606379 nadolol Adrenergic receptor antagonist

846 0.39 cp BRD-K96354014 nifedipine Calcium channel blocker

830 0.42 cp BRD-K34581968 BMS-536924 IGF-1 inhibitor

831 0.42 cp BRD-K51318897 fenbendazole Tubulin inhibitor

832 0.42 cp BRD-K80431395 triciribine AKT inhibitor

833 0.42 cp BRD-K47936004 piribedil Dopamine receptor agonist

834 0.42 cp BRD-A78391468 prednisolone Glucocorticoid receptor agonist

835 0.42 cp BRD-K32830106 guanfacine Adrenergic receptor agonist

836 0.42 cp BRD-A69917777 aminopentamide Acetylcholine receptor antagonist

838 0.42 cp BRD-K47869605 podophyllotoxin Microtubule inhibitor

839 0.42 cp BRD-K50398167 meclofenamic-acid Cyclooxygenase inhibitor

840 0.42 cp BRD-K79254416 decitabine DNA methyltransferase inhibitor

829 0.43 cp BRD-K21936341 oxotremorine Acetylcholine receptor agonist

822 0.46 cp BRD-A01787639 naftopidil Adrenergic receptor antagonist

823 0.46 cp BRD-K32107296 temozolomide DNA alkylating agent

824 0.46 cp BRD-K45446451 JZL-184 Monoacylglucerol lipase inhibitor

825 0.46 cp BRD-K80970344 pyrrolidine-dithiocarbamate NFkB pathway inhibitor

826 0.46 cp BRD-A35511923 L-803087 Somatostatin receptor agonist

827 0.46 cp BRD-A58564983 selamectin Nematocide

828 0.46 cp BRD-K76723084 isotretinoin Retinoid receptor agonist

817 0.49 cp BRD-A56012032 thiorphan Membrane metalloendopeptidase inhibitor

818 0.49 cp BRD-K82562631 tolmetin Cyclooxygenase inhibitor

819 0.49 cp BRD-K35531059 molsidomine Guanylyl cyclase activator

820 0.49 cp BRD-K07237224 moclobemide Monoamine oxidase inhibitor

821 0.49 cp BRD-K10995081 perphenazine Dopamine receptor antagonist

816 0.5 cp BRD-K07212038 selinidin Mast cell stabilizer

811 0.53 cp BRD-K72222507 quinapril ACE inhibitor

812 0.53 cp BRD-A91008255 bepridil Calcium channel blocker

813 0.53 cp BRD-A64092382 mexiletine Sodium channel blocker

810 0.55 cp BRD-K47780086 penciclovir DNA directed DNA polymerase inhibitor

808 0.56 cp BRD-A45499626 UBP-302 Glutamate receptor antagonist

798 0.6 cp BRD-K42221274 NNC-711 GAT inhibitor

799 0.6 cp BRD-A00267231 hemado Adenosine receptor agonist

800 0.6 cp BRD-K46018455 bezafibrate PPAR receptor agonist

801 0.6 cp BRD-K78294846 osthol Calcium channel blocker

802 0.6 cp BRD-K92760278 riboflavin Vitamin B

804 0.6 cp BRD-K85133207 HDAC1-selective HDAC inhibitor

805 0.6 cp BRD-K21450440 benzthiazide Carbonic anhydrase inhibitor

806 0.6 cp BRD-A29734509 disopyramide Sodium channel blocker

797 0.61 cp BRD-K93880783 stavudine DNA directed DNA polymerase inhibitor

790 0.63 cp BRD-K52080565 rilmenidine Imidazoline receptor agonist

791 0.63 cp BRD-K11717138 benzbromarone Chloride channel blocker

792 0.63 cp BRD-K45401373 betulinic-acid Apoptosis stimulant

793 0.63 cp BRD-A72703248 SKF-96365 Calcium channel blocker

794 0.63 cp BRD-K92731339 perindopril ACE inhibitor

789 0.65 cp BRD-A97674275 ranolazine Sodium channel blocker

779 0.67 cp BRD-A15530910 carpindolol Adrenergic receptor antagonist

780 0.67 cp BRD-K26573499 DMAB-anabaseine Adrenergic receptor agonist

781 0.67 cp BRD-A41304429 practolol Adrenergic receptor antagonist

782 0.67 cp BRD-K59273480 propentofylline Adenosine reuptake inhibitor

784 0.67 cp BRD-K66876909 linezolid Bacterial 50S ribosomal subunit inhibitor

785 0.67 cp BRD-A70407468 PSB-36 Adenosine receptor antagonist

776 0.68 cp BRD-A16934955 nalbuphine Opioid receptor agonist

777 0.68 cp BRD-K75958195 pizotifen Serotonin receptor antagonist

771 0.7 cp BRD-K69690935 curcumin Cyclooxygenase inhibitor

772 0.7 cp BRD-A30655177 LFM-A13 BTK inhibitor

773 0.7 cp BRD-K66019333 oxantel Anthelmintic

770 0.71 cp BRD-A35588707 teniposide Topoisomerase inhibitor

765 0.74 cp BRD-K00486786 RO-08-2750 NGF binding inhibitor

766 0.74 cp BRD-K93658967 aloisine CDK inhibitor

767 0.74 cp BRD-K15108141 gemcitabine Ribonucleotide reductase inhibitor

761 0.78 cp BRD-K39391626 ethylestrenol Progesterone receptor agonist

762 0.78 cp BRD-A62025033 temsirolimus MTOR inhibitor

763 0.78 cp BRD-A61392169 eliprodil Glutamate receptor antagonist

755 0.81 cp BRD-A76279427 myriocin Serine palmitoyltransferase inhibitor

756 0.81 cp BRD-K97309399 thiothixene Dopamine receptor antagonist

757 0.81 cp BRD-K47717570 NBQX Glutamate receptor antagonist

758 0.81 cp BRD-K96263742 GW-7647 PPAR receptor agonist

759 0.81 cp BRD-K74236984 UNC-0321 Histone lysine methyltransferase inhibitor

749 0.85 cp BRD-K70281171 U-99194 Dopamine receptor antagonist

750 0.85 cp BRD-K46142322 RS-67333 Serotonin receptor partial agonist

751 0.85 cp BRD-K99107520 felbamate Glutamate receptor antagonist

752 0.85 cp BRD-K72783841 tyrphostin-AG-555 EGFR inhibitor

753 0.85 cp BRD-A77349281 RK-682 Tyrosine phosphatase inhibitor

744 0.88 cp BRD-A70731303 avrainvillamide-analog-5 nucleophosmin inhibitor

745 0.88 cp BRD-K27450477 EHNA Adenosine deaminase inhibitor

746 0.88 cp BRD-K12184916 dactolisib MTOR inhibitor

737 0.92 cp BRD-K05151076 ZK-164015 Estrogen receptor antagonist

738 0.92 cp BRD-K34995470 SU-1498 VEGFR inhibitor

743 0.92 cp BRD-A80213327 NSC-23766 Ras GTPase inhibitor

732 0.95 cp BRD-K85402309 dovitinib EGFR inhibitor

733 0.95 cp BRD-K02590140 O-2050 Cannabinoid receptor antagonist

721 0.99 cp BRD-A43082555 loxoprofen Cyclooxygenase inhibitor

722 0.99 cp BRD-K04853698 LDN-193189 Serine/threonine kinase inhibitor

723 0.99 cp BRD-K16277217 piperacetazine Dopamine receptor antagonist

724 0.99 cp BRD-K67439147 SIB-1893 Glutamate receptor antagonist

725 0.99 cp BRD-A54845972 dihydroergotamine Serotonin receptor agonist

726 0.99 cp BRD-K89839824 raltitrexed Thymidylate synthase inhibitor

727 0.99 cp BRD-K14888893 minoxidil KATP activator

728 0.99 cp BRD-K35960502 niclosamide DNA replication inhibitor

714 1.02 cp BRD-K50866992 tropisetron Serotonin receptor antagonist

715 1.02 cp BRD-K65814004 diphenyleneiodonium Nitric oxide synthase inhibitor

716 1.02 cp BRD-K03600606 catechin Beta secretase inhibitor

717 1.02 cp BRD-A65597028 RX-821002 Adrenergic receptor antagonist

708 1.06 cp BRD-A52588987 SKF-83566 Dopamine receptor antagonist

710 1.06 cp BRD-K41895714 AS-605240 PI3K inhibitor

702 1.09 cp BRD-K93095519 SJ-172550 MDM inhibitor

703 1.09 cp BRD-K16977723 PP-3 EGFR inhibitor

704 1.09 cp BRD-A78322124 dobutamine Adrenergic receptor agonist

697 1.13 cp BRD-K62374253 rufloxacin Bacterial DNA gyrase inhibitor

698 1.13 cp BRD-K44779798 miglitol Glucosidase inhibitor

699 1.13 cp BRD-K31054881 BMY-7378 Adrenergic receptor antagonist

700 1.13 cp BRD-K63068307 ZSTK-474 PI3K inhibitor

695 1.14 cp BRD-K08547377 irinotecan Topoisomerase inhibitor

689 1.16 cp BRD-K46469693 SCH-442416 Adenosine receptor antagonist

690 1.16 cp BRD-K23623876 decafluorobutane Contrast agent

691 1.16 cp BRD-A41250306 cyclopenthiazide Thiazide diuretic

692 1.16 cp BRD-K54142781 cirazoline Adrenergic receptor agonist

693 1.16 cp BRD-K95851186 CGP-13501 GABA receptor modulator

694 1.16 cp BRD-K64341947 CFM-1571 Guanylate cyclase activator

687 1.17 cp BRD-K13646352 midostaurin FLT3 inhibitor

685 1.18 cp BRD-K44876623 zolpidem Benzodiazepine receptor agonist

682 1.2 cp BRD-A28422058 L-689560 Glutamate receptor antagonist

683 1.2 cp BRD-K65503129 HSP90-inhibitor HSP inhibitor

684 1.2 cp BRD-K67844266 MLN-4924 Nedd activating enzyme inhibitor

677 1.23 cp BRD-K56614220 clofazimine GK0582 inhibitor

678 1.23 cp BRD-A41941932 vitexin Antioxidant

679 1.23 cp BRD-A58157837 butabindide Tripeptidyl peptidase inhibitor

676 1.24 cp BRD-K75295174 alisertib Aurora kinase inhibitor

671 1.27 cp BRD-K93460210 lamotrigine Serotonin receptor antagonist

672 1.27 cp BRD-K99174507 cardiogenol-c Cardiomyogenesis inducer

661 1.3 cp BRD-K13800121 parecoxib Cyclooxygenase inhibitor

662 1.3 cp BRD-K32398298 alprazolam Benzodiazepine receptor agonist

663 1.3 cp BRD-K68190965 GR-46611 Serotonin receptor agonist

664 1.3 cp BRD-A88282067 delcorine Antiarrhythmic

665 1.3 cp BRD-K10870738 CDC Lipoxygenase inhibitor

668 1.3 cp BRD-K89152108 liothyronine Thyroid hormone stimulant

669 1.3 cp BRD-K93034159 cladribine Adenosine deaminase inhibitor

655 1.34 cp BRD-K12906962 dichlorobenzamil Sodium/calcium exchange inhibitor

656 1.34 cp BRD-K51541829 RO-25-6981 Ionotropic glutamate receptor antagonist

649 1.37 cp BRD-K72024482 MRS-1754 Adenosine receptor antagonist

650 1.37 cp BRD-A96882008 L-732138 Tachykinin antagonist

651 1.37 cp BRD-K30990140 FR-122047 Cyclooxygenase inhibitor

652 1.37 cp BRD-K32292990 CGP-53353 EGFR inhibitor

654 1.37 cp BRD-K94294671 OSI-027 MTOR inhibitor

648 1.39 cp BRD-K57011718 UK-356618 Metalloproteinase inhibitor

647 1.4 cp BRD-K87696786 LY-456236 Glutamate receptor antagonist

645 1.41 cp BRD-K97181089 amiloride Sodium channel blocker

646 1.41 cp BRD-K04210847 tamoxifen Estrogen receptor antagonist

642 1.44 cp BRD-K68620903 dydrogesterone Progesterone receptor agonist

640 1.46 cp BRD-A20126139 medrysone Glucocorticoid receptor agonist

637 1.48 cp BRD-A50928468 norgestrel Progesterone receptor agonist

638 1.48 cp BRD-K07888107 depudecin HDAC inhibitor

634 1.52 cp BRD-A64228451 terreic-acid BTK inhibitor

635 1.52 cp BRD-K99696746 fatostatin SREBP inhibitor

626 1.55 cp BRD-K41337261 ZM-306416 ABL inhibitor

627 1.55 cp BRD-K00206590 P-1075 ATP channel activator

628 1.55 cp BRD-K44899736 RO-16-6941 Monoamine oxidase inhibitor

629 1.55 cp BRD-K96670504 lonidamine Glucokinase inhibitor

631 1.55 cp BRD-K91623615 ABT-751 Tubulin inhibitor

617 1.59 cp BRD-K28115081 apafant Platelet activating factor receptor antagonist

618 1.59 cp BRD-A93000692 ciglitazone PPAR receptor agonist

620 1.59 cp BRD-A93255169 thalidomide TNF production inhibitor

610 1.62 cp BRD-K34437622 BRD-K34437622 Thymidylate synthase inhibitor

611 1.62 cp BRD-A49046702 SKF-89976A GABA uptake inhibitor

612 1.62 cp BRD-K20714604 RS-56812 Serotonin receptor partial agonist

613 1.62 cp BRD-K18316707 O-1918 Cannabinoid receptor antagonist

606 1.64 cp BRD-K03109492 NSC-663284 CDC inhibitor

601 1.66 cp BRD-A13807286 HA-14-1 BCL inhibitor

602 1.66 cp BRD-K25875056 SC-9 Protein tyrosine kinase activator

599 1.67 cp BRD-A60070924 alpha-estradiol Estrogen receptor agonist

593 1.69 cp BRD-K00184207 GR-206 Aryl hydrocarbon receptor ligand

594 1.69 cp BRD-K53972329 ruxolitinib JAK inhibitor

595 1.69 cp BRD-K59456551 methotrexate Dihydrofolate reductase inhibitor

588 1.72 cp BRD-A91866971 SQ-29548 Thromboxane receptor antagonist

586 1.73 cp BRD-K82688027 RG-13022 PDGFR receptor inhibitor

587 1.73 cp BRD-K48168960 propylthiouracil Thyroid peroxidase inhibitor

579 1.76 cp BRD-K04993501 cefixime Bacterial cell wall synthesis inhibitor

580 1.76 cp BRD-K28907958 CD-437 Retinoid receptor agonist

581 1.76 cp BRD-K70330367 amantadine Glutamate receptor antagonist

574 1.8 cp BRD-K37798499 etoposide Topoisomerase inhibitor

575 1.8 cp BRD-A13084692 troglitazone Insulin sensitizer

576 1.8 cp BRD-K53318339 vinpocetine Phosphodiesterase inhibitor

577 1.8 cp BRD-A18620900 estriol Estrogen receptor agonist

570 1.83 cp BRD-K17349619 HLI-373 MDM inhibitor

571 1.83 cp BRD-K27351809 nomegestrol Progesterone receptor agonist

567 1.86 cp BRD-A30205217 ethotoin Hydantoin antiepileptic

566 1.87 cp BRD-K67566344 KU-0063794 MTOR inhibitor

563 1.88 cp BRD-K57304726 PRE-084 Sigma receptor agonist

560 1.9 cp BRD-K49404994 levetiracetam Calcium channel blocker

561 1.9 cp BRD-K03981224 ethisterone Progestogen hormone

555 1.93 cp BRD-K14536225 piceid Glucosidase inhibitor

553 1.94 cp BRD-K28143534 cyproheptadine Histamine receptor antagonist

547 1.97 cp BRD-K81855038 roxatidine Histamine receptor antagonist

548 1.97 cp BRD-A63836183 PD-123319 Angiotensin receptor antagonist

549 1.97 cp BRD-K39733634 L-161982 Prostanoid receptor antagonist

550 1.97 cp BRD-K77793136 hydroxyfasudil Rho associated kinase inhibitor

542 2.04 cp BRD-K84175871 pseudoephedrine Adrenergic receptor agonist

543 2.04 cp BRD-A05352148 ipratropium Acetylcholine receptor antagonist

536 2.08 cp BRD-K26015241 ODQ Guanylyl cyclase inhibitor

537 2.08 cp BRD-K12120659 GR-144053 Integrin antagonist

535 2.11 cp BRD-K68246049 TTNPB Retinoid receptor agonist

533 2.15 cp BRD-K29359156 ebselen H+/K+-ATPase inhibitor

527 2.18 cp BRD-K82381502 acetylcholine Acetylcholine receptor agonist

528 2.18 cp BRD-K94325918 kinetin-riboside Apoptosis stimulant

523 2.22 cp BRD-A10903566 imiloxan Adrenergic receptor antagonist

520 2.26 cp BRD-K93331255 lypressin Vasopressin receptor agonist

521 2.26 cp BRD-K77286328 reversine Aurora kinase inhibitor

513 2.29 cp BRD-K48722833 iloperidone Dopamine receptor antagonist

514 2.29 cp BRD-A98431941 ephedrine Adrenergic receptor agonist

509 2.32 cp BRD-K76872913 benzanthrone Aromatic hydrocarbon derivative

507 2.33 cp BRD-K52620403 STO-609 Calmodulin antagonist

503 2.36 cp BRD-K13356952 methazolamide Carbonic anhydrase inhibitor

504 2.36 cp BRD-K69556541 ryanodine Calcium channel blocker

505 2.36 cp BRD-A45140972 meclocycline Bacterial 30S ribosomal subunit inhibitor

497 2.43 cp BRD-K83597974 pargyline Monoamine oxidase inhibitor

492 2.5 cp BRD-K84595254 strophanthidin ATPase inhibitor

491 2.54 cp BRD-K28137194 loreclezole GABA receptor agonist

488 2.57 cp BRD-K70505054 ranitidine Histamine receptor antagonist

483 2.61 cp BRD-K20313525 rosmarinic-acid GABA transaminase inhibitor

479 2.64 cp BRD-K63516691 T-0156 Phosphodiesterase inhibitor

482 2.64 cp BRD-K77008974 WYE-354 MTOR inhibitor

478 2.65 cp BRD-K18619710 digoxigenin Steroid

470 2.71 cp BRD-K85925969 zalcitabine Nucleoside reverse transcriptase inhibitor

467 2.73 cp BRD-K05977823 tenovins SIRT inhibitor

468 2.73 cp BRD-K82109576 vincristine Tubulin inhibitor

464 2.74 cp BRD-A38913120 BH3I-1 BCL inhibitor

461 2.78 cp BRD-A96255180 ribavirin Antiviral

451 2.85 cp BRD-A77118605 BML-ST330 Phospholipase inhibitor

452 2.85 cp BRD-K66412701 pazufloxacin Topoisomerase inhibitor

446 2.92 cp BRD-K01902415 pirinixic-acid PPAR receptor agonist

447 2.92 cp BRD-K63874012 thioperamide Histamine receptor antagonist

445 2.94 cp BRD-K10670311 sulfasalazine Antirheumatic

441 2.96 cp BRD-K11373525 ZD-7155 Angiotensin receptor antagonist

442 2.96 cp BRD-K89402695 L-655240 Thromboxane receptor antagonist

437 3 cp BRD-K83322645 L-693403 Sigma receptor agonist

433 3.05 cp BRD-A14208071 oxyphenonium Cholinergic receptor antagonist

431 3.07 cp BRD-K27184429 levocetirizine Histamine receptor antagonist

428 3.1 cp BRD-K64785675 TG100-115 -666

429 3.1 cp BRD-A43974575 tranylcypromine Monoamine oxidase inhibitor

422 3.14 cp BRD-A59174698 ritodrine Adrenergic receptor agonist

423 3.14 cp BRD-K84266862 BRL-50481 Phosphodiesterase inhibitor

419 3.17 cp BRD-K09471561 levofloxacin Bacterial DNA gyrase inhibitor

413 3.28 cp BRD-K62982419 cilomilast Phosphodiesterase inhibitor

410 3.31 cp BRD-K06792661 narciclasine Coflilin signaling pathway activator

408 3.33 cp BRD-A11605036 thiocolchicoside GABA receptor antagonist

407 3.35 cp BRD-K59058766 chlorprothixene Dopamine receptor antagonist

406 3.41 cp BRD-A28856712 tetryzoline Adrenergic receptor agonist

400 3.43 cp BRD-K09436313 prostaglandin Prostanoid receptor antagonist

401 3.43 cp BRD-A93477898 PETCM Caspase activator

403 3.43 cp BRD-K21548250 moracizine Sodium channel blocker

396 3.45 cp BRD-K61993165 niacin NAD precursor with lipid lowering effects

397 3.45 cp BRD-A29437505 RWJ-21757 TLR agonist

395 3.46 cp BRD-K37846922 "3,3'-diindolylmethane" CHK inhibitor

394 3.49 cp BRD-K74501079 azithromycin Bacterial 50S ribosomal subunit inhibitor

391 3.5 cp BRD-A71459254 cymarin ATPase inhibitor

388 3.56 cp BRD-A17411484 carprofen Cyclooxygenase inhibitor

389 3.56 cp BRD-A35912562 pregnenolone Glutamate receptor modulator

384 3.66 cp BRD-K47679368 bromfenac Cyclooxygenase inhibitor

382 3.68 cp BRD-K26863634 BIX-01338 Histone lysine methyltransferase inhibitor

380 3.7 cp BRD-K54472332 elvitegravir HIV integrase inhibitor

377 3.73 cp BRD-A15010982 HU-211 Glutamate receptor antagonist

376 3.78 cp BRD-K16478699 PLX-4720 RAF inhibitor

375 3.81 cp BRD-K37865504 LY-2183240 FAAH inhibitor

369 3.94 cp BRD-K43764301 dexketoprofen Cyclooxygenase inhibitor

366 4.05 cp BRD-K28075147 biochanin-a Estrogen receptor agonist

363 4.08 cp BRD-K59753975 vindesine Tubulin inhibitor

360 4.12 cp BRD-M30523314 vinorelbine Tubulin inhibitor

359 4.16 cp BRD-K10573841 tunicamycin GLCNAC phosphotransferase inhibitor

356 4.17 cp BRD-A55946879 BW-B70C Lipoxygenase inhibitor

354 4.23 cp BRD-K90733503 cefalexin Bacterial cell wall synthesis inhibitor

344 4.44 cp BRD-K14329163 BAY-K8644 Calcium channel activator

342 4.47 cp BRD-K86003836 flubendazole Tubulin inhibitor

337 4.55 cp BRD-K28667196 fillalbin Increases arterial blood pressure

339 4.55 cp BRD-M40783228 mesna Antioxidant

335 4.58 cp BRD-K75181824 acetyl-geranygeranyl-cysteine Inhibitor of methyl esterification of geranylgeranylated proteins

329 4.75 cp BRD-K42142750 retrorsine Antimitotic

322 4.97 cp BRD-K14643723 4-(2-Amino-ethyl)-benzenesulfonamide carbonic anhydrase inhibitor

323 4.97 cp BRD-K92301463 "16,16-dimethylprostaglandin-e2" Prostanoid receptor agonist

320 5.07 cp BRD-A83326220 brazilin Nitric oxide production inhibitor

319 5.13 cp BRD-K24681473 YM-155 Survivin inhibitor

312 5.4 cp BRD-K35483542 alitretinoin Retinoid receptor agonist

311 5.41 cp BRD-A43940795 tetrahydropalmatine Serotonin release inhibitor

310 5.47 cp BRD-K40213712 SAL-1 Adenosine receptor antagonist

309 5.52 cp BRD-A03216249 mepivacaine Potassium channel blocker

307 5.66 cp BRD-K75615183 talipexole Adrenergic receptor agonist

305 5.69 cp BRD-A33084410 5'-guanidinonaltrindole Opioid receptor antagonist

304 5.76 cp BRD-K58772419 AZD-6482 PI3K inhibitor

303 5.8 cp BRD-K79602928 metformin Insulin sensitizer

298 5.9 cp BRD-A37492983 iocetamic-acid Radiopaque medium

296 5.95 cp BRD-A89434049 sarmentogenin ATPase inhibitor

288 6.52 cp BRD-A55369275 CGP-54626 GABA receptor antagonist

281 6.83 cp BRD-A54927599 KF-38789 P-selectin inhibitor

280 6.99 cp BRD-A62890442 3-methyl-GABA GABA aminotransferase activator

279 7 cp BRD-K21806131 tegaserod Serotonin receptor partial agonist

278 7.02 cp BRD-K62736196 guanabenz -666

277 7.04 cp BRD-K26997899 SA-792574 Microtubule inhibitor

276 7.1 cp BRD-K41051431 mecillinam Bacterial cell wall synthesis inhibitor

273 7.2 cp BRD-K53979406 ALX-5407 Glycine transporter inhibitor

272 7.3 cp BRD-A42628519 iopanoic-acid Radiopaque medium

270 7.4 cp BRD-K74112339 acetohydroxamic-acid Urease inhibitor

267 7.63 cp BRD-K63343048 orlistat Lipase inhibitor

266 7.65 cp BRD-K76674262 homoharringtonine Protein synthesis inhibitor

265 7.79 cp BRD-K24576554 AT-9283 JAK inhibitor

263 7.85 cp BRD-A11007541 BCI-hydrochloride Protein phosphatase inhibitor

262 7.94 cp BRD-A80928489 1-monopalmitin P-glycoprotein inhibitor

259 8.08 cp BRD-K40758068 efavirenz HIV protease inhibitor

257 8.17 cp BRD-K81209512 AG-494 EGFR inhibitor

254 8.73 cp BRD-K83302049 protopine Histamine receptor antagonist

250 8.84 cp BRD-K89930444 AG-592 Tyrosine kinase inhibitor

244 9.62 cp BRD-K42095107 daidzein Estrogen receptor agonist

237 10.19 cp BRD-K87932577 CDK1-5-inhibitor CDK inhibitor

235 10.38 cp BRD-K74141488 naftifine Fungal squalene epoxidase inhibitor

234 10.39 cp BRD-A41995253 brucine Glycine receptor antagonist

232 10.43 cp BRD-K48692744 NU-1025 PARP inhibitor

231 10.63 cp BRD-K51485625 ritonavir HIV protease inhibitor

230 10.72 cp BRD-K92138166 mammea-a other antibiotic

229 10.73 cp BRD-K99818283 PIK-90 PI3K inhibitor

228 10.89 cp BRD-A98299281 velnacrine cholinesterase inhibitor

227 10.93 cp BRD-K62363391 dapsone Bacterial antifolate

224 11.16 cp BRD-K91442916 CAM-9-026 Membrane metalloendopeptidase inhibitor

223 11.22 cp BRD-K89162000 tandutinib FLT3 inhibitor

219 11.56 cp BRD-U86922168 QL-XII-47 BTK inhibitor

214 11.86 cp BRD-K51967704 BIIB021 HSP inhibitor

215 11.86 cp BRD-A33833419 TER-14687 Inhibitor of translocation of PKCq in T cells

213 11.91 cp BRD-K55930204 phenytoin Hydantoin antiepileptic

212 12.03 cp BRD-K50384076 "7,4'-dihydroxyflavone" Opioid receptor antagonist

209 12.31 cp BRD-K86191271 cytosporone-b NUR77 receptor agonist

203 13.21 cp BRD-K10852020 tolcapone Catechol O methyltransferase inhibitor

202 13.44 cp BRD-K18855837 varenicline Acetylcholine receptor agonist

199 13.72 cp BRD-K95785537 PP-2 SRC inhibitor

194 14.42 cp BRD-K28360340 TW-37 BCL inhibitor

192 14.88 cp BRD-K09255212 clioquinol Chelating agent

191 14.95 cp BRD-A15415227 GW-1929 PPAR receptor agonist

190 15.13 cp BRD-K63504947 semaxanib VEGFR inhibitor

189 15.32 cp BRD-K03406345 azacitidine DNA methyltransferase inhibitor

188 15.4 cp BRD-K68095457 palmitoylethanolamide Cannabinoid receptor agonist

187 15.69 cp BRD-K19295594 gossypol BCL inhibitor

186 16.02 cp BRD-K13049116 BMS-754807 IGF-1 inhibitor

184 16.87 cp BRD-K92015269 GBR-12783 Dopamine uptake inhibitor

183 17.22 cp BRD-A15079084 phorbol-12-myristate-13-acetate PKC activator

182 17.31 cp BRD-A82371568 clofarabine Ribonucleoside reductase inhibitor

181 17.41 cp BRD-K67277431 picotamide Thromboxane receptor antagonist

179 17.96 cp BRD-K25504083 cytochalasin-d Actin polymerization inhibitor

178 18.12 cp BRD-K34441861 moexipril ACE inhibitor

177 18.19 cp BRD-K08589866 linsitinib IGF-1 inhibitor

176 18.51 cp BRD-A15034104 bergenin Interleukin inhibitor

174 19.85 cp BRD-M64432851 sunitinib FLT3 inhibitor

172 21.14 cp BRD-A50675702 fipronil GABA gated chloride channel blocker

168 22.12 cp BRD-K18787491 U-0126 MEK inhibitor

166 22.21 cp BRD-K64517075 heliomycin ATP synthase inhibitor

161 23.06 cp BRD-A35338386 NECA Adenosine receptor agonist

162 23.06 cp BRD-K47150025 KI-8751 VEGFR inhibitor

160 23.08 cp BRD-K39520573 GW-5074 Leucine rich repeat kinase inhibitor

156 23.7 cp BRD-K22009844 phenprobamate Muscle relaxant

154 24.01 cp BRD-A56892734 esomeprazole ATPase inhibitor

153 24.11 cp BRD-A94543220 bifonazole Sterol demethylase inhibitor

152 24.42 cp BRD-K78084463 W-12 Calmodulin antagonist

145 27.17 cp BRD-A25687296 emetine Protein synthesis inhibitor

144 27.47 cp BRD-K68488863 ENMD-2076 FLT3 inhibitor

143 27.54 cp BRD-K87048468 RS-102221 Serotonin receptor antagonist

140 28.86 cp BRD-K41903098 diphenoxylate Opioid receptor agonist

136 30.31 cp BRD-A62182663 YK-4279 Apoptosis stimulant

134 30.33 cp BRD-K77677632 SB-200646 Serotonin receptor antagonist

133 30.51 cp BRD-A02710418 meptazinol Opioid receptor agonist

130 31.02 cp BRD-K95901403 XL-147 PI3K inhibitor

129 31.18 cp BRD-K18799075 BAY-59-3074 Cannabinoid receptor partial agonist

127 31.55 cp BRD-K98493452 honokiol AKT inhibitor

125 31.73 cp BRD-K31987754 oleylethanolamide Cannabinoid receptor agonist

124 31.94 cp BRD-A06352508 SB-218078 CHK inhibitor

123 32.01 cp BRD-K32330832 VER-155008 HSP inhibitor

120 33.28 cp BRD-K77175907 calcifediol Vitamin D receptor agonist

119 33.37 cp BRD-A09539288 homatropine Acetylcholine receptor antagonist

118 33.98 cp BRD-K49669041 BX-912 Pyruvate dehydrogenase kinase inhibitor

111 36.65 cp BRD-K82577285 dipropyl-dopamine Dopamine receptor agonist

110 37.26 cp BRD-M86331534 pyrvinium-pamoate AKT inhibitor

106 38.21 cp BRD-A41555725 chlortetracycline Protein synthesis inhibitor

102 40.54 cp BRD-U33728988 QL-X-138 MTOR inhibitor

101 41.01 cp BRD-K51223576 AG-99 Tyrosine kinase inhibitor

100 42.56 cp BRD-K78278890 NM-PP1 Mutant kinase inhibitor

99 43.58 cp BRD-K86727142 embelin HCV inhibitor

95 44.81 cp BRD-A55594068 vinblastine Microtubule inhibitor

88 46.79 cp BRD-K61341215 vecuronium Acetylcholine receptor antagonist

87 46.94 cp BRD-K12539581 nocodazole Tubulin inhibitor

81 49.56 cp BRD-K66792149 quinoclamine Algicide

77 50.17 cp BRD-A78360835 cercosporin Photoactivated toxin

76 50.7 cp BRD-K69032158 diprotin-a Dipeptidyl peptidase inhibitor

75 51.2 cp BRD-K19894101 MST-312 Telomerase inhibitor

74 51.81 cp BRD-A68930007 ouabain ATPase inhibitor

69 54.06 cp BRD-K63606607 bufalin ATPase inhibitor

67 54.28 cp BRD-K83988098 alvespimycin HSP inhibitor

61 57.31 cp BRD-K56334280 amonafide Topoisomerase inhibitor

58 59.4 cp BRD-K54095730 CMPD-1 p38 MAPK inhibitor

51 64.08 cp BRD-K62609077 scoulerine Adrenergic receptor antagonist

50 64.83 cp BRD-A80641450 FR-139317 Endothelin receptor antagonist

48 65.25 cp BRD-A55484088 BNTX Opioid receptor antagonist

44 67.05 cp BRD-K92991072 PAC-1 Caspase activator

42 68.08 cp BRD-A44551378 LFM-A12 EGFR inhibitor

40 69.3 cp BRD-A94756469 digoxin ATPase inhibitor

38 69.86 cp BRD-A93236127 digitoxin ATPase inhibitor

37 71.29 cp BRD-K91370081 anisomycin DNA synthesis inhibitor

32 72.6 cp BRD-K05658747 raltegravir HIV integrase inhibitor

26 75.78 cp BRD-A45333398 periplocymarin Apoptosis stimulant

25 76.42 cp BRD-K47983010 BX-795 IKK inhibitor

22 79.87 cp BRD-K91145395 prostratin PKC activator

20 82.82 cp BRD-A62184259 cycloheximide Protein synthesis inhibitor

18 83.1 cp BRD-K15600710 obatoclax BCL inhibitor

17 83.48 cp BRD-K76698671 HNHA HDAC inhibitor

16 83.69 cp BRD-A34806832 proscillaridin ATPase inhibitor

13 85.65 cp BRD-A80502530 cinobufagin ATPase inhibitor

6 90.77 cp BRD-A63998256 helveticoside ATPase inhibitor

5 90.84 cp BRD-A52650764 ingenol PKC activator
